# ﻿A revision of *Lycianthes* (Solanaceae) in Australia, New Guinea, and the Pacific

**DOI:** 10.3897/phytokeys.209.87681

**Published:** 2022-09-23

**Authors:** Sandra Knapp

**Affiliations:** 1 Natural History Museum, Cromwell Road, London SW7 5BD, UK The Natural History Museum London United Kingdom

**Keywords:** Australia, conservation, endemism, *Lycianthes*, New Guinea, Oceania, species diversity

## Abstract

The genus *Lycianthes* (Dunal) Hassl. (Solanaceae) has in the past been treated as a section of the large genus *Solanum* L., but is more closely related to *Capsicum* L. The eighteen species of *Lycianthes* occurring in Australia, New Guinea (defined as the island of New Guinea, comprising Papua New Guinea [incl. Bougainville] and the Indonesian provinces of Papua Barat and Papua, plus the surrounding islands connected during the last glacial maximum) and the Pacific Islands are here treated in full, with complete descriptions, including synonymy, typifications and synonyms, distribution maps and illustrations. The history of taxonomic treatment of the genus in the region is also discussed. These taxa occupy a diverse range of forested habitats, and are in diverse in habit, from small shrubs to large canopy lianas to epiphytic shrubs. They are for the most part rarely collected, and many are endemic (14 of the 18 species treated here). Australia has a single endemic *Lycianthes* species (*L.shanesii* (F.Muell.) A.R.Bean). Nine species are found in both Indonesia and Papua New Guinea, one in Indonesia only, four in Papua New Guinea only, and *L.vitiensis* (Seem). A.R.Bean is known from Bougainville (Papua New Guinea) and the south Pacific as far east as Samoa. *Lyciantheslucens* S.Knapp **sp. nov.** is described from the islands of Lihir, New Ireland and the Louisiade Archipelago of Papua New Guinea. The cultivated *L.rantonnetii* (Carrière) Bitter is also treated in full, in this region known currently only from Australia; it is native to southern South America. Preliminary conservation assessments are presented for all species except the cultivated *L.rantonnetii*.

## ﻿Introduction

*Lycianthes* (Dunal) Hassl. is the third largest genus in the Solanaceae, after *Solanum* L. and *Cestrum* L. (Dean et al. 2020). The genus comprises approximately 150 to 200 species, most of these distributed in the Americas, with the centre of species diversity in Mesoamerica. Significant species diversity is present outside of the Americas however, with New Guinea, the world’s most species-rich tropical island (Cámara-Leret et al. 2020), standing out as an area of high endemism, as has been seen for many other taxa. Previously considered a section of *Solanum*, *Lycianthes* has been shown to be more closely related to the peppers (*Capsicum* L.) despite sharing the striking character of poricidal anthers with *Solanum* (Särkinen et al. 2013).

*Lycianthes* can be recognised by the combination of axillary inflorescences, poricidal anthers and a calyx without distinct lobes, but rather with a truncate rim (sometimes called a sleeve) with or without tooth-like appendages protruding from near or below the calyx rim (D’Arcy 1986). Species of *Lycianthes* can be confused with other genera with similar unlobed calyces like *Capsicum* L., *Brachistus* Miers, *Cuatresia* Hunz., and *Witheringia* L’Hér. (D’Arcy 1986; Dean et al. 2020), especially when flowers are lacking.

**Figure 1.**
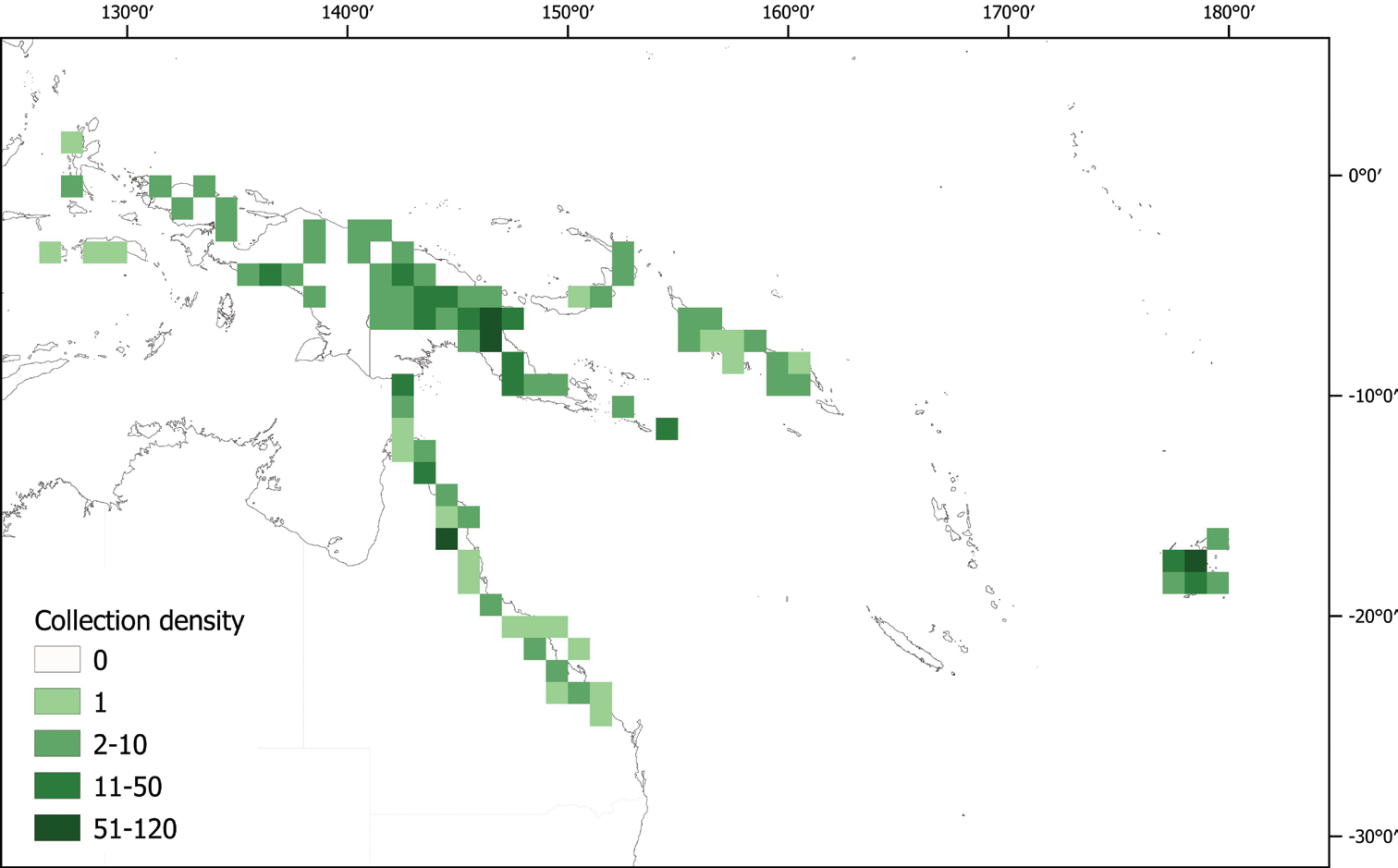
Map of all collections examined for *Lycianthes* in the area treated in this monograph (Australia, New Guinea and the Pacific). Darker squares indicate those with more collections.

This revision is the first in a series of treatments of the species of *Lycianthes* outside the Americas, and part of collaborative work to treat the species of this genus worldwide (e.g., Dean et al. 2020). In this revision, I treat *Lycianthes* in the broad area encompassing Australia, New Guinea and the islands of the south Pacific as far east as Samoa (see Fig. 1 for a map of the region treated in this monograph and Table 1 for a list of the species and their distributions). I am treating New Guinea as the broad geologically coherent area comprising the Indonesia provinces of Papua and Papua Barat, the country Papua New Guinea and the surrounding small islands that were connected during the last glacial maximum (as defined by Cámara-Leret et al. 2020, see figure 1 and discussion in that reference), but also including Bougainville (as of today politically part of Papua New Guinea, but due to become independent in 2027) and the islands of the Pacific that were not connected at that time.

**Table 1.** Comparison of species recognised in Bitter (1919), Symon (1981a, 1985; Symon and Clarkson 1985) and this treatment. Bold face type indicates accepted species by the relevant authors. Species treated as synonyms are in square brackets.

Bitter (1919)	Symon (1981a, 1985); Symon and Clarkson (1985)	This treatment
[not included]	[*Solanumacuminatissimum* Merr. & L.M.Perry; as syn. of *S.moszkowskii*]	[syn. of *L.moszkowskii*]
Lycianthesbiflora(Lour.)Bittersubsp.biflora	*Solanumbiflorum* Lour.	*Lycianthesbiflora* (Lour.) Bitter
[not included]	*Solanumbitterianum* Symon	*Lycianthesbitteriana* (Symon) A.R.Bean
*Lycianthesbalanidium* (Bitter) Bitter	[syn. of *S.memecylonoides*]	[syn. of *L.oliveriana*]
*Lycianthesbambusarum* (Bitter) Bitter	*Solanumbambusarum* Bitter	*Lycianthesbambusarum* (Bitter) Bitter
[not included]	*Solanumbelense* Merr. & L.M.Perry	*Lycianthesbelensis* (Merr. & L.M.Perry) A.R.Bean
*Lycianthescladotrichota* (Bitter)Bitter	*Solanumcladotrichotum* Bitter	*Lycianthescladotrichota* (Bitter)Bitter
[not included]	*Solanumdendropilosum* Symon	*Lycianthesdendropilosa* (Symon) A.R.Bean
*Lycianthesimpar* (Warb.) Bitter	*Solanumimpar* Warb.	*Lycianthesimpar* (Warb.) Bitter
*Lyciantheskaernbachii* (Lauterb. & K.Schum.) Bitter	*Solanumkaernbachii* Lauterb. & K. Schum.	*Lyciantheskaernbachii* (Lauterb. & K. Schum.) Bitter
*Lycianthesledermannii* (Bitter) Bitter	[syn. of *S.oliverianum*]	[syn. of *L.oliveriana*]
[not included]	[not included]	*Lyciantheslucens* S.Knapp
*Lycianthesmemecylonoides* (Bitter & Schltr.) Bitter	*Solanummemecylonoides* Bitter & Schltr.	[syn. of *L.oliveriana*]
*Lycianthesmoszkowskii* (Bitter) Bitter	*Solanummoszkowskii* Bitter	*Lycianthesmoszkowskii* (Bitter) Bitter
*Lycianthesoliveriana* (Lauterb. & K. Schum.) Bitter	*Solanumoliverianum* Lauterb. & K. Schum.	*Lycianthesoliveriana* (Lauterb. & K. Schum.) Bitter
*Lycianthespatellicalyx* (Bitter) Bitter	[syn. of *S.cladotrichotum*]	[syn. of *L.cladotrichota*]
[not included]	*Solanumperanomalum* Wernham ex Ridl.	*Lycianthesperanomala* (Wernham ex Ridl.) A.R.Bean
[not included]	*Solanumpustulatum* Symon	[syn of *L.rostellata*]
[not included]	[*Solanumridleyanum* Wernham; as syn. of *S.moszkowskii*]	[syn. of *S.impar*]
*Lycianthesrechingeri* (Witasek) Bitter	[syn. of *S.vitiense* Seem.)	[syn. of *L.vitiensis*]
[not included]	*Solanumrantonnei* (Carrière) Bitter	*Lycianthesrantonnetii* (Carrière) Bitter
[not included]	*Solanumrostellatum* Merr. & L.M.Perry	*Lycianthesrostellata* (Merr. & L.M.Perry) A.R.Bean
*Lycianthesschlechteriana* (Bitter) Bitter	[syn. of *S.kaernbachii*]	[syn. of *L.kaernbachii*]
[not included]	*Solanumshanesii* F.Muell.	*Lycianthesshanesii* (F.Muell.) A.R.Bean
[not included]	*Solanumvitiense* Seem.	*Lycianthesvitiensis* (Seem.) A.R.Bean
[not included]	*Solanumwollastonii* Wernham	*Lyciantheswollastonii* (Wernham) A.R.Bean

## ﻿Taxonomic and phylogenetic history

***Taxonomy***. Taxa now recognised as *Lycianthes* were first included in the large and diverse genus *Solanum*, due to the shared poricidal anthers. Dunal (1813, 1816, 1852) recognised them as his group “Polymeris” based on the calyx appendages and axillary flowers. This group was maintained by subsequent authors (see summary in Dean et al. 2020), eventually being recognised at the subgeneric level by the German botanist Georg Bitter (Bitter 1917) in a treatment of *Solanum* in the Papuan region.

In the same year the group was distinguished as a distinct genus by Émile (or Emil) Hassler (Hassler 1917), who included only a few of the species in Dunal’s larger group in *Solanum* (Dunal 1852) that contained stone cells in their fruits, all from the Americas. Bitter (1919) later recognised the distinctness of *Lycianthes* at the generic level and his worldwide monograph of the genus contained 189 taxa (species and infraspecies), most transferred from *Solanum*. He suggested the genus was closely related to *Capsicum* based on their similar calyx morphology; this suggestion, however, was not widely accepted until the advent of molecular phylogenetic studies in the Solanaceae (see below). Throughout the rest of the 20^th^ century various authors continued to treat the species of *Lycianthes* as members of *Solanum* (e.g., Morton 1976; Hunziker 1979; Symon 1985), or as a distinct genus related to *Solanum* (e.g., Hunziker 2001).

Phylogenetic analyses of Solanaceae using DNA sequence data showed that the few species of *Lycianthes* included were indeed most closely related to *Capsicum* and not to *Solanum* (e.g., Särkinen et al. 2013); few species from outside the Americas were included in this analysis. These and other more focused studies on the two genera appeared to suggest that *Lycianthes* was paraphyletic with respect to *Capsicum* (Spalink et al. 2018), but considerable discordance between different genomic regions complicates resolution of the monophyly of *Lycianthes*. Its close relationship to *Capsicum*, however, is now undisputed. Recent studies using whole plastome sequences and more numerous nuclear sequences (A. Orejuela, pers. comm.) support the reciprocal monophyly of *Capsicum* and *Lycianthes*; these more recent studies have included more species of *Lycianthes* from outside the Americas (see species treatments for specifics).

Dean et al. (2020) summarised the taxonomic history of *Lycianthes* in the Americas; taxa outside the Americas have been less intensively studied. Approximately 30–40 species of *Lycianthes* occur across Asia from northern India to the Pacific islands of Fiji and Samoa. Several of these are widespread and taxonomically very difficult (e.g., *L.biflora* (Lour.) Bitter) and are the subject of ongoing investigation. Most species of *Lycianthes* are not commonly collected, in the Americas many of them have flowers that are only open for a short time each day (see Dean et al. 2020) and they often occur in small, scattered populations. The species of *Lycianthes* from New Guinea are unusual in being largely endemic, making them a discrete group floristically, although their monophyly has not been demonstrated. *Lycianthesshanesii* (F.Muell.) A.R.Bean is endemic to Australia; it was not treated by Bitter (1919) or Symon (1985). Symon (1981a, b) excluded it from *Solanum* in his treatment of Australian solanums, and suggested it was a species of *Capsicum*.

The first of the species treated here to be described was *Lycianthesbiflora* (Lour.) Bitter (as *S.biflorum* Lour.) in the Portuguese missionary João de Loureiro’s flora for tropical Asia (Loureiro 1790). Loureiro’s work was based on his long residence in the southern third of today’s Vietnam, then known as ‘Cochinchina’ (Merrill 1935). *Lycianthesbiflora* is widespread across tropical Asia, Louriero’s plants were from Indochina; the island of New Britain is the easternmost part of the range of *L.biflora*, which is common throughout Indochina into India.

In the islands of the Pacific, Seemann (1863) described *Solanumvitiense* Seem. from Fiji, based on his own collections made for his *Flora Vitiensis*. Reinecke (1898) placed similar plants from Samoa into the genus *Brachistus* (as *B.feddei* Reinecke), suggesting it was identical or very closely related to Seeman’s *S.vitiense*; in his view *S.vitiense* was not a *Solanum* based on the tree-like habit, longitudinal dehiscence of the free anthers and anthers inserted at the mouth of the corolla. His best guess as to generic placement (Reinecke 1898) was *Brachistus*, although he realised his plants did not fit well there either (see description of *L.vitiensis* (Seem.) A.R.Bean for details).

The first of the endemic New Guinea *Lycianthes* was described by Otto Warburg (1891), using his own collections made on a short visit to what is now the Indonesian province of Papua Barat (*Solanumimpar* Warb.). Schumann and Lauterbach (1900) published a flora of the German “protectorates” on the island (now the northern part of Papua New Guinea) and in it included *S.vitiense* from the island of Bougainville and described two new taxa (*S.oliverianum* Lauterb. & K.Schum., *S.kaernbachii* Lauterb. & K.Schum.), both based on collections at the Berlin Botanic Garden (B) that were destroyed during the Second World War.

In his treatment of Papuan *Solanum*, Bitter (1917) included two subsections in subgenus Lycianthes ; section Polymeris (Dunal) Bitter, with a single member, *L.biflora*, and section Cypellocalyx Bitter, with twelve taxa, all but three of them new to science, mostly based on single collections (*S.bambusarum* Bitter, *S.memecylonoides* Bitter, S.memecylonoidessubsp.finisterrae Bitter, *S.balanidium* Bitter, *S.cladotrichotum* Bitter, *S.patellicalyx* Bitter, *S.moszkowskii* Bitter, *S.oliverianum*, *S.ledermannii* Bitter, *S.impar*, *S.kaernbachii*, *S.schlechterianum* Bitter).

Two years later in his worldwide monograph of *Lycianthes* that he now recognised at the generic level, Bitter (1919) transferred all of his Papuan taxa described earlier (Bitter 1917) to *Lycianthes* without comment and without citing new material. He recognised the Asian species related to *L.biflora* as section Asiomelanesia Bitter and retained his section Cypellocalyx. He did not include in either treatment the species described by Wernham (1916) from the collections made on Puncak Jaya (Mount Jaya or Mount Carstenz) by Cecil Boden Kloss on the “Wollaston Expedition to Dutch New Guinea” (Ridley 1916): *S.peranomalum* Wernham ex Ridl., *S.ridleyanum* Wernham and *S.wollastonii* Wernham. This undoubtably reflects the difficulty in scientific communication, particularly for German botanists, in the years following the First World War.

Merrill and Perry (1949) described several more species now recognised as *Lycianthes* (again as SolanumsectionLycianthes) in their treatment of plants collected on the various collecting trips funded by the wealthy philanthropist Richard Archbold to New Guinea just prior to the Second World War. These taxa were described from single collections made by the botanist Leonard Brass (*S.acuminatissimum* Merr. & L.M.Perry, *S.belense* Merr. & L.M.Perry, *S.multifolium* Merr. & L.M.Perry, *S.rostellatum* Merr. & L.M.Perry). They also documented *S.rechingeri* Witasek and *S.impar* from Brass’s collections. They compared their new taxa to those previously described, suggesting that *S.rostellatum* was closely related to *S.cladotrichotum*.

Symon (1985) revised the genus *Solanum* for New Guinea following intensive collecting efforts undertaken by Australian and Papua New Guinean botanists in Papua New Guinea and Dutch botanists in what was then known as the Indonesian province of Irian Jaya. He treated the species of *Lycianthes* at the subgeneric level in *Solanum*, stating “I am not yet satisfied that these species justify generic status while the fundamental cleavage between the *Pachystemonum* and *Leptostemonum* [non-spiny and spiny solanums] is accepted within a single genus.” Earlier, however, he had maintained the two genera as distinct (Symon 1984a, b). In the New Guinea monograph (Symon 1985) he maintained Bitter’s (1917) sections *Polymeris* and *Cypellocalyx*, describing one new species in the former (*S.bitterianum* Symon) and three new species in the latter (*S.dendropilosum* Symon, *S.pustulatum* Symon, *S.umbonatum* Symon). Symon (1985) recognised two species in *Polymeris* and 16 in *Cypellocalyx* (see Table 1). He included *S.vitiense* due to its occurrence on Bougainville, and synonymised *S.rechingeri* Witasek (Witasek 1908) and *Brachistusfeddei* with it. Symon and Clarkson (1985) recognised *S.shanesii* F.Muell. as a member of “section Lycianthes”; correcting Symon’s (1981a, b) previous assignment of the species to *Capsicum*. Further work on *Lycianthes* in the region used these names in *Solanum* until Bean (2003) transferred all taxa still with only names in *Solanum* to *Lycianthes*; he did not provide descriptions or critical analysis of species limits, and largely repeated type citations from Symon (1985) and Symon and Clarkson (1985).

Smith (1991) in his treatment of Solanaceae for his revision of Seemann’s (1865–1873) flora of Fiji, treated the only species of *Lycianthes* in Fiji as a member of *Solanum* (as *Solanumvitiense* Seem.), with *B.feddei* in synonymy, following Symon (1985); he did not include the Samoan *S.rechingeri* in synonymy.

***Phylogeny***. Few of these species have been included in molecular phylogenies. Särkinen et al. (2013) found *Lycianthesshanesii* to be sister to a small clade comprising *L.biflora* and *L.lysimachioides* (Wall.) Bitter (another Asian species occurring from India to Japan); together these were sister to a large heterogenous clade of American species. Spalink et al. (2018), examining the role of genomic discordance in the ambiguity of the backbone of the *Lycianthes*+*Capsicum* clade, sampled mostly American taxa, they only included *L.biflora* from Asia in their analyses. They found *L.biflora* to be sister to the rest (n=7) of the *Lycianthes* and *Capsicum* species in their data set, suggesting *Lycianthes* is paraphyletic relative to *Capsicum* as currently defined.

The preliminary analyses of full plastome sequences (A. Orejuela, pers. comm.) suggest that the only two New Guinea species included (*Lycianthesoliveriana* and *L.cladotrichota*) are sister taxa and are nested within a larger group containing American and Asian species (incl. *L.lysimachioides*), but more taxa need to be included in analyses to ascertain if the diversity of endemic species on New Guinea represents a unique radiation, as is the case for other endemic lineages on New Guinea (Cámara-Leret et al. 2020). These full plastome results suggest *Capsicum* and *Lycianthes* are reciprocally monophyletic, with very high support (A. Orejuela, pers. comm.). It is clear that the Australasian species of *Lycianthes* are important for understanding the evolution and biogeography of the genus, and of the origins of *Capsicum*. Ongoing work (E. Gardner, L. Bohs and S. Knapp, pers. comm.) with broader sampling across the region treated here and southeast Asia more generally will address these questions.

## ﻿Morphology

***Habit and stems***. All species treated here are perennial, woody plants. Habit is extremely variable, ranging from small shrubby forms that are sometimes somewhat herbaceous (e.g., *Lycianthesbiflora*) to large woody vines (e.g., *L.kaernbachii*, *L .oliveriana*) or trees more than 10 m tall (e.g., *L.vitiensis*). As with many species of Solanaceae that form large woody vines (e.g., Knapp and Vorontsova 2016; Dean et al. 2020), small plants growing at the edges of clearings can flower and are therefore described as shrubs on herbarium labels. The occurrence of many of these taxa in mossy forests (e.g., *L.wollastonii*) means that it can be difficult to ascertain life form from herbarium labels, so caution must be taken with assuming any given species is one life form or another.

Stems of all the native species are terete, and variously pubescent (see Pubescence below). The stems of the cultivated *Lycianthesrantonnetii* are strongly ridged and angled, especially on older stems. Bark of taxa that are trees or large vines is usually greyish brown and is often peeling or blistered on older stems.

**Figure 2.**
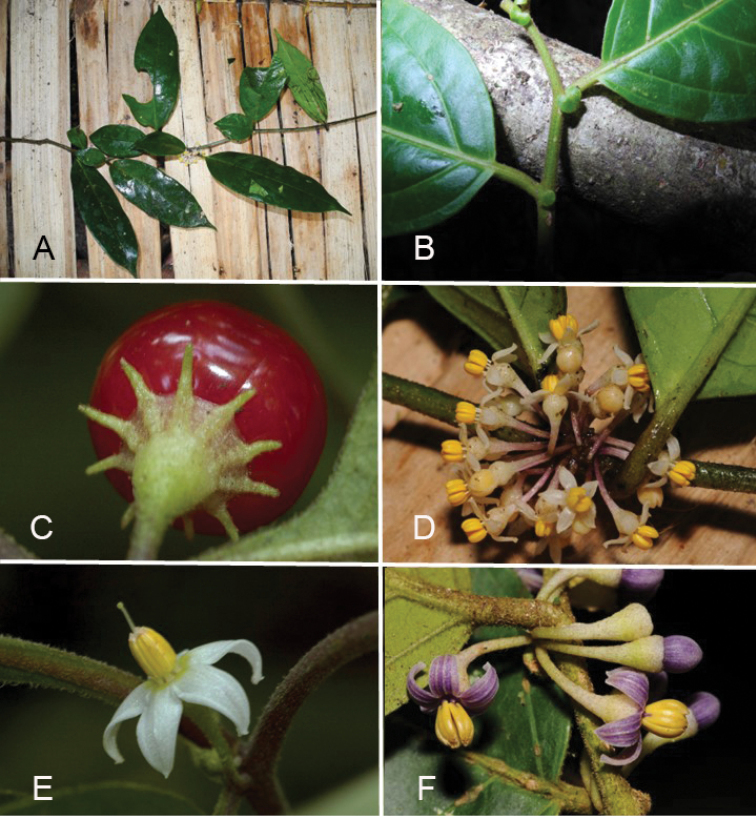
Representative morphology of *Lycianthes***A***Lycianthesoliveriana* with major and minor leaves differing only in size (*Damas et al. SAJ-1050*, Papua New Guinea) **B***Lycianthescladotrichota* with major and minor leaves differing markedly in size and shape (*James et al. SAJ-1385*, Papua New Guinea) **C***Lycianthesbiflora* in fruit with linear, awn-like calyx appendages (*Knapp 10106*, Yunnan, China) **D***Lycianthesoliveriana* with urceolate calyces with no appendages (*Damas et al. SAJ-1050*, Papua New Guinea) **E***Lycianthesbiflora* with stellate corollas and membranous lobes (*Knapp 10106*, Yunnan, China) **F***Lycianthescladotrichota* with stellate corollas and thick, fleshy lobes (short-styled plant, *James et al. SAJ-1385*, Papua New Guinea). Photograph credits: **A, B, D, F** Shelley James; **C, E** Sandra Knapp.

Growth in the Solanaceae is initially monopodial, but with the onset of flowering, becomes sympodial. Inflorescences terminate each branch, and stem growth continues from two axillary buds subtending the inflorescence (seen in species of *Capsicum* and some American *Lycianthes*, see Dean et al. 2020), or growth can continue from a single axillary bud, forming a monochasial branching pattern (as seen in most species of *Solanum* and in the taxa treated here). Each lateral shoot with alternate leaves arranged in a 1/3 phyllotaxic spiral and a terminal inflorescence (segment of stem between each inflorescence) is termed the sympodial unit in the descriptions here. Danert (1958) showed that when growth of the axes is suppressed, the leaves appear paired (geminate) at a node, thus the arrangement of leaves along the stems of Solanaceae is due to concaulescence of sympodial units, such that leaves can appear either singly or in groups. The number and arrangement of leaves in each sympodial unit can be diagnostic for both clades and species (e.g., Knapp 2002; Peralta et al. 2008). All of the taxa treated here have difoliate sympodial units with leaves usually strongly paired (geminate) at the nodes (Fig. 2A, B); these paired leaves are either similar in both size and shape, similar in shape but differing in size (e.g., *L.oliveriana*, *L.shanesii*), or very different in both size and shape (e.g., *L.impar*, *L.rostellata*).

***Leaves***. Leaves of *Lycianthes* are simple, lobed or sinuate margins have not been observed in these species in the region treated here. Field labels of *L.shanesii* mention the margins as “undulate” but this is not apparent on dried specimens. Most species have slightly discolorous leaves, with the abaxial surfaces paler than the adaxial surfaces, which are often described as dark green. Leaf texture varies from membranous (e.g., *L.multifolia*) to thick and somewhat coriaceous (e.g., *L.impar*, *L.oliveriana*) to somewhat chartaceous (e.g., *L.cladotrichota*).

Many of the species treated here have anisophyllous sympodial units (see above), with the major and minor leaves of markedly different size and/or shape (see Fig. 2A, B and illustrations of individual species). In species descriptions I have described the major (larger) leaves first, followed by the minor leaves. The minor leaves in many species are deciduous (e.g., *Lycianthesrostellata*) as branches age, or in drying.

**Figure 3.**
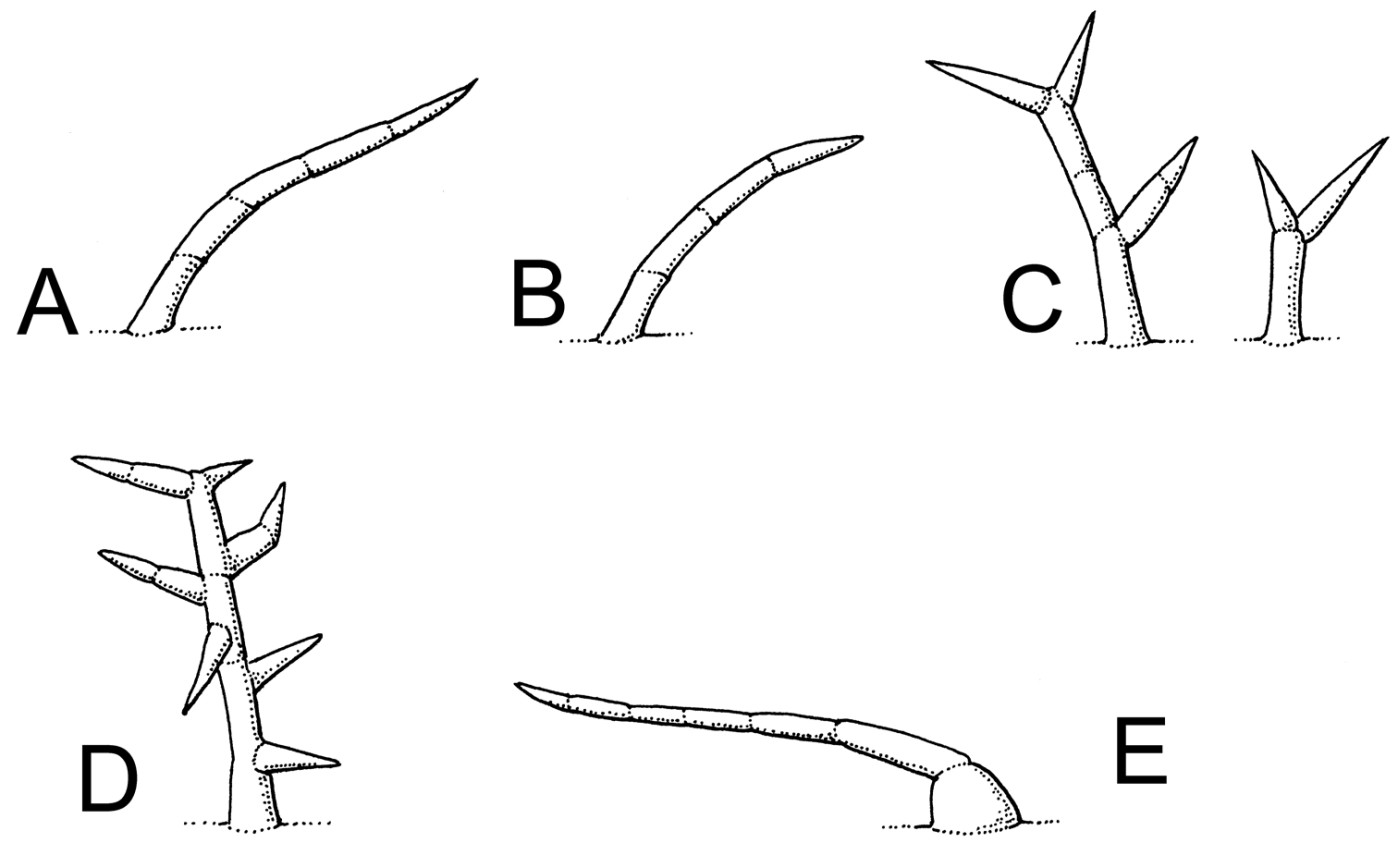
Trichome diversity in New Guinea *Lycianthes***A** simple (unbranched) stiff, antrorse trichomes (*Streimann & Katik NGF-34091*) **B** simple (unbranched) “crisped”trichomes (*Kalkmann BW-3479*) **C** dendritic (antler-like) trichomes (*Streimann & Katik NGF-34091*) **D** dendritic (Christmas-tree like) trichomes (*Wells NGF-7565*) **E** simple (unbranched) stiff, antrorse trichomes with enlarged bases (*Millar NGF-40737*). Drawing by Lucy Smith.

***Pubescence***. Trichome morphology can be very useful in Solanaceae taxonomy (see Seithe 1962, 1979; Knapp 2001) with type, density, and colour often diagnostic for species identification. Trichome type varies between, but not within species in the region. All of the species of *Lycianthes* treated here have tiny papillate trichomes on the new growth, these are common on most Solanaceae and are not those usually used for species differentiation. Trichomes in the taxa here, where present, are all uniseriate and of a single rank of cells (see Fig. 3); they are simple (unbranched, consisting of a single row of cells; e.g., *L.kaernbachii*, *L.moszkowskii*), branched like deer-antlers (with long branches that are spread apart; e.g., *L.biflora*, *L.cladotrichota*) or tightly branched (with the branches closely spaced and congested; e.g., *L.bitteriana*, *L.dendropilosa*). These tightly dendritic trichomes were termed “tannenbaumartig” (Christmas-tree type) by Seithe (1962), to differentiate them from echinoid trichomes (see Vorontsova and Knapp 2016; Aubriot and Knapp 2022) that are derived from stellate trichomes and usually have a much shorter central axis. *Lyciantheskaernbachii* occasionally has a few forked (furcate, with two branches only and “Y”-shaped) trichomes mixed in with more numerous simple trichomes on the stems. The stem trichomes of *L.rostellata* often have somewhat pustulate, multicellular bases; the extreme of this character was the basis for Symon’s *S.pustulatum*, here treated as a synonym of *L.rostellata*. On older stems these remain behind as small warty protuberances.

Many of the taxa here have antrorse trichomes all directed to the distal portion of axes, these can be stiff (e.g., *Lyicanthescladotrichota*, *L.peranomala*) or softer (e.g., *L.multifolia*, *L.wollastonii*). These latter trichomes have often been described as “crisped” (meaning slightly curled, e.g., Symon 1985) but it is unclear if the curling nature of the hairs is due to drying or is a feature present on live plants as well. These softer trichomes are often weak-walled and collapsing in a moniliform way (with cells compressing in alternate directions).

Multicellular glandular trichomes only occur in the cultivated *Lycianthesrantonnetii*, and then only mixed with eglandular trichomes, the native species in this region have only eglandular trichomes.

***Inflorescences***. Inflorescences of all species of *Lycianthes* appear to arise from the leaf axils (see Sympodia above), often as small fascicles of only a few flowers (see Dean et al. 2020). The apparently axillary inflorescence is one unequivocal character distinguishing *Lycianthes* from *Solanum* (although see Knapp and Helgason 1997, the Pteroidea clade of *Solanum* has axillary inflorescences, but these are never fasciculate). Some species in the region develop a short scorpioid axis, along which flowers are borne in pairs (e.g., *L.impar*, *L.vitiensis*); this rarely exceeds a centimetre or so in length (see Figs 22, 52). The inflorescence in *L.kaernbachii* is unusual in being concaulescent with the stem such that the flowers are borne in two rows along the internodes, often the entire length between leaves (see Figs 25, 26). The development of this unusual structure, and of the inflorescence in *Lycianthes* in general, has not been investigated.

The number of flowers in fascicles is variable between species (a single or only two in *Lycianthesbelensis* versus up to 20 or even more in *L.oliveriana*), but also within species, depending on the age of the inflorescence (see individual species illustrations). In some species (e.g., *L.oliveriana*, *L.vitiensis*) the number of flowers in each inflorescence can vary depending upon the age of the inflorescence, early flowering inflorescences have few flowers while older ones have many. The number of flowers is in these taxa not reliable for species level identification.

***Calyces***. In all species of *Lycianthes* the calyx is synsepalous and unlobed (D’Arcy 1986; Dean et al. 2020). The cup-like calyx tube has an entire truncate rim, although in *L.vitiensis* the hyaline rim appears somewhat irregularly lobed through tearing (see Fig. 51). Calyx appendages, if present, emerge from below the truncate rim or from very near it; there is often a small margin of tissue between the appendage and the edge of the rim. The calyx appendages can vary in number and length even within a single plant, but their presence or absence is generally consistent within a species. Appendages can be linear and subulate (e.g., *L.biflora*, see Fig. 2D), small, broad triangles perpendicular to the calyx tube (e.g., *L.lucens*, *L.wollastonii*) or mere bumps that are much more apparent in bud than in mature flowers (e.g., *L.bambusarum*). The cultivated *L.rantonnetii* often has calyx appendages of two different lengths emerging at slightly different levels on the calyx tube. Most of the species here have no calyx appendages, and the calyx is a truncate cup (e.g., *L.kaernbachii*, *L.oliveriana*, *L.rostellata*) and is occasionally somewhat urceolate (see Fig. 2E).

In most of the species here the calyx is described as fleshy and is often a contrasting colour to the corolla or the fruit, usually white or cream or purple (Fig. 2D, F). In dry specimens the fruiting calyx appears woody and sometimes (e.g., *Lycianthesoliveriana*) is tightly invested around the lower part of the berry and fruits on herbarium specimens are reminiscent of acorns (see Fig. 38). The calyx of dried specimens is often warty (somewhat tuberculate); this uneven texture is also apparent in live plants (see Fig. 2E). Label descriptions of fruits in the field suggest the calyx cup subtending the berry is an integral part of the display to frugivores (see description of *L.impar*).

***Corollas***. The *Lycianthes* corolla is sympetalous, with 5 (occasionally 4) lobes. Corolla shape varies from deeply stellate to rotate; deeply stellate corollas are divided nearly to the base and rarely have thin interpetalar tissue connecting the lobes (e.g., *L.rostellata*), while rotate corollas have abundant interpetalar tissue (e.g., *L.rantonnetii*). The only species occurring in this region with rotate corollas is the cultivated *L.rantonnetii* (Figs 43, 44) but in the Americas many species of *Lycianthes* have this corolla morphology (see Dean et al. 2020). Corollas on herbarium specimens are generally open, and appear not to exhibit diurnal movements as observed by Dean (2001).

Interpetalar tissue is thinner than that of the lobes proper, is usually folded within the bud before anthesis, and usually lacks the pubescence found on the rest of the abaxial corolla surface ; lobes of several of the native species treated here have thin but obvious margins of interpetalar tissue, such as *Lycianthesbitteriana*, *L.belensis*, *L.multifolia* and *L.shanesii*. Other taxa lack any interpetalar tissue and the lobes are distinctly valvate in bud and usually densely papillate along the entire lobe margin (e.g., *L.cladotrichota*, *L.impar*, *L.oliveriana*, *L.peranomala*, see. Fig. 2 and illustrations of individual species).

Corolla colour in these species varies from white to dark purple, with many species having individuals with either colour or with patterns of colour such as the midveins of corolla lobes darker than the rest of the tissue (e.g., *Lycianthesshanesii*). Reliance of corolla colour for identification is not recommended. Symon (1985) identified some regional differentiation in corolla colour (white versus violet) in *L.vitiensis* (as. *S.vitiense*) but suggested this might have been due to colour change through the life of the flower as is seen in some species of *Solanum* (e.g., *S.wendlandii* Hook., Knapp et al. 2017) or *Brunfelsia* L. (e.g., *B.australis* Benth., Plowman 1998). *Lycianthesbiflora* flowers often have a greenish yellow or yellow eye at the centre (actually at the base of each lobe) like that seen in many species of *Solanum* (see Knapp 2013; Särkinen et al. 2018). The function of these markings, sometimes referred to as “pseudonectaries”, is not known.

***Androecium***. Stamens of *Lycianthes* are ‘Solanum-type’ (sensu Endress 1996) and specialised for buzz-pollination by bees. The anthers are usually longer than the filaments and dehisce by terminal pores. Bees grasp the anther cone and vibrate their indirect flight muscles to setup a vibration that causes pollen to be ejected from the anther theca (Buchmann et al. 1977; Vallejo-Marín et al. 2010). Endress (1996) has suggested that the poricidal nature of anthers is a secondarily acquired feature in plants with dry pollen, since vibratile pollination occurs in many plants without poricidal anthers. Most species of *Lycianthes* treated here have ellipsoid anthers (e.g., *L.kaernbachii*, *L.oliveriana*) but in some taxa the apical portion of the anther is tapered, giving them a pointed appearance (e.g., *L.rostellata*). The base of the anthers is always to some degree sagittate. The anthers are in general not tightly coherent or fused together, but some species (e.g., *L.kaernbachii*, *L.oliveriana*, *L.vitiensis*) only form a loose structure in the centre of the flower.

Terminal anther pores in the taxa treated here are always distally directed but are of two distinct shapes. Pores are either circular and distinctly bounded, or somewhat tear-drop shaped, with a line of dehiscence extending between the anther locules for varying lengths. In taxa where the pore is tear-drop shaped (e.g., *Lycianthesbiflora*, *L.bitteriana*, *L.wollastonii*) the terminal anther pores elongate as the flower ages, and the anthers appear to “unzip” (D’Arcy et al. 1996; Knapp 2002) and become somewhat longitudinally dehiscent. In *L.vitiensis*, the anthers are sometimes completely longitudinally dehiscent, with no pore-like structure present. In species with circular pores (e.g., *L.cladotrichota*, *L.oliveriana*), the anther pore does not change shape with flower age and remains a distinct round opening at the distal tip. At anther dehiscence in all species the pore or other shaped opening is edged with white crystals which have been described as the “oxalate package” (D’Arcy et al. 1996). These crystals have been implicated in anther dehiscence but are not differently placed in species with poridical anthers (see D’Arcy et al. 1996).

Unlike the heterantherous androecia of many American *Lycianthes* species (see Dean et al. 2020), all native species occurring in the region have stamens, anthers, and filaments of equal length. The bright orange anthers of the cultivated *L.rantonnetii*, however, are borne on filaments of different lengths, two short and three long (see description and Fig. 43).

Differences in filament versus anther length can be useful in species identification. For example, the anthers and filaments of *Lycianthesoliveriana* are of similar lengths (see Fig. 2D) while the anthers of the similarly glabrous *L.bambusarum* are much longer than the filaments. It seems from specimens that the filaments of short-styled flowers are sometimes longer than those of long-styled flowers (e.g., *L.kaernbachii*); these species are likely dioecious, and this polymorphism may be similar to that seen in some dioecious species of *Solanum* (see Anderson et al. 2015).

Filaments in most of the species treated here are glabrous, but *Lycianthescladotrichota* and the cultivated *L.rantonnetii* have tangled weak-walled simple trichomes on the adaxial surfaces of the filaments (inside the stamen tube).

***Gynoecium***. The gynoecium in *Lycianthes* is bicarpellate with axile placentation. The ovary is glabrous, and usually conical to globose. The flowers appear to lack nectaries, as do all species of *Lycianthes*. In species with heterostylous flowers the ovary in short-styled flowers is vestigial. The style is usually straight and glabrous, but in the cultivated *L.rantonnetii* is it strongly curved. In long-styled flowers the style is exserted from the anther cone, but in short-styled flowers it is contained well within (e.g., Fig. 2F). The stigma is either very minutely capitate (e.g., *L.biflora*) or larger and more obviously capitate (e.g., *L.moszkowskii*) clavate (e.g., *L.rostellata*) or markedly bilobed with strongly diverging lobes (e.g., *L.cladotrichota*). The ovules are anatropous and non-arillate.

***Fruits***. In *Lycianthes*, the fruit is a globose to subglobose to ellipsoid berry. In the species treated here, berries are globose to subglobose in most taxa. *Lycianthesimpar* appears to have ellipsoid berries but I have seen only a single specimen with mature fruits (*Kloss s.n.*). In cultivation *L.rantonnetii* rarely sets fruit, but in its native range the berries are large and ellipsoid (see Barboza 2013 and Fig. 43). Berry colour in *Lycianthes* can be green, yellow, orange, red, blue or dark purplish black (see Dean et al. 2020) at maturity, and all are green when immature. While most of the species here have soft, fleshy berries, those of *L.kaernbachii* and *L.oliveriana* appear to remain hard through maturity (they never flatten on pressing and drying). Some species (e.g., *L.lucens*) have translucent shiny pericarp (epicarp) at maturity, while others are opaque and matte (e.g., *L.belensis*, *L.oliveriana*). The majority of species treated here have red or orange red berries at maturity (e.g., *L.biflora*, *L.moszkowskii*), although mature berries of several are purple or purplish black (e.g., *L.bitteriana*, *L.impar*, *L.oliveriana*, *L.rostellata*). *Lycianthesvitiensis* has berries that begin green, pass through orange, eventually become bright red when mature (although *Kajewski 1863* records them as blue, see description). Mature fruits are not known from *L.bambusarum*, *L.dendropilosa*, and *L.wollastonii*.

Across the Solanaceae small inclusions known as sclerids, brachyclerids or stone cells are found in the fruit, often mixed amongst the seeds (Bitter 1911, 1914). In the large genus *Solanum*, this character is found in particular clades (e.g., Archaesolanum, Morelloid; see Symon 1994; Särkinen et al. 2018). These concretions are composed of modified sclerenchyma cells with massively enlarged cell walls; the stone cells of pears and quinces (Rosaceae) are classic examples of this cell type. Neither their function nor their origin in Solancaeae is known. Bitter (1914) suggested that they existed in an evolutionary series in the family, with more “advanced” taxa lacking them altogether. In the species treated here stone cells are found only in *Lycianthesrantonnetii* (see Fig. 43), which belongs to the clade that includes the type of *Lycianthes* (Särkinen et al. 2013). None of the species of *Lycianthes* native to Australia, New Guinea and the Pacific Islands have stone cells.

***Seeds***. Seeds of *Lycianthes* are usually flattened or compressed vary from round to ovoid across the genus (Dean et al. 2020). In the species treated here, seeds are flattened and vary from pale straw-colored to rusty red. *Lycianthesbambusarum* and *L.rostellata* have a distinctive deep hilar notch. *Lycianthesmoszkowskii* has unusual large seeds (ca. 6 mm in diameter) with a broad lateral wing somewhat reminiscent of the winged seeds of *Solanumcapsicioides* All. (Acanthophora clade of Leptostemonum; see Vorontsova and Knapp 2016). Winged seeds are unusual in berry-type fruits. In *L.impar* seeds are thin, elongate and kidney-shaped (Fig. 22). Seed number per berry varies from 2 to more than 100 seeds per berry, most species have in the range of 20–50 seeds per berry.

Seed coat morphology has been suggested as a useful character in the taxonomy of Solanaceae (Souèges 1907; Lester and Durrands 1984). The lateral walls of the outer seed epidermal layer can develop lignified radial thickenings that appear as hair-like structures (Souèges 1907; Lester and Durrands 1984; Peralta et al. 2008). When the outer wall of the epidermis is removed, either naturally or by enzymatic digestion (Lester and Durrands 1984; Knapp 2002) such seeds appear pubescent; seed measurements here include these projections. In the species treated here the outline of seed testal cells varies from rectangular or pentagonal to deeply sinuate (also termed cerebelloid); this is usually constant within a species and can be useful confirming identifications.

## ﻿Ecology and natural history

***Distribution and habitats***. Twelve of the 18 species treated here are found only on the island of New Guinea, the world’s most species-rich tropical island (Table 2). *Lyciantheslucens* and *L.vitiensis* are confined to islands to the east of New Guinea, with *L.vitiensis* occurring as far east as Samoa. *Lycianthesbiflora* is widely distributed throughout tropical Asia from India to southern China and Japan, including most of Indonesia, but is not found in Australia. Symon (1986) suggested phytogeographical links to Asia for *L.biflora* and *L.bitteriana*, which he recognised as section Asiomelanesia, but strong links to the Americas for the rest of the species treated here (which he recognised as members of section Cypellocalyx). He suggested these species were related to the Central American *L.synanthera* (Sendtn.) Bitter and *L.nitida* Bitter and represent the remnants of a group once widespread across Gondwana. These biogeographic ideas need to be tested with robust phylogenetic hypotheses, but initial results from whole plastid DNA sequencing (A. Orejuela, pers. comm.) suggest the New Guinea species are indeed closely related to *L.synanthera*, but few species have been included in analyses to date.

**Table 2.** *Lycianthes* species in Australia, New Guinea and the Pacific with their distribution and putative system (see text for discussion of breeding systems). Species endemic to a single country are indicated in bold face type.

Species	Distribution	Putative breeding system
*Lycianthesbambusarum* (Bitter) Bitter	Indonesia (Papua, Papua Barat), Papua New Guinea	Flowers unisexual; plants dioecious
*Lycianthesbelensis* (Merr. & L.M.Perry) A.R.Bean	Indonesia (Papua, Papua Barat), Papua New Guinea	Flowers unisexual; plants dioecious
*Lycianthesbiflora* (Lour.) Bitter	Indonesia (Papua, Papua Barat), Papua New Guinea (widespread in rest of tropical Asia from India to Philippines and north to Japan and China)	Flowers bisexual; plants hermaphroditic
***Lycianthesbitteriana* (Symon) A.R.Bean**	**Papua New Guinea**	Flowers bisexual; plants hermaphroditic
*Lycianthescladotrichota* (Bitter) Bitter	Indonesia (Papua, Papua Barat), Papua New Guinea	Flowers unisexual; plants dioecious
***Lycianthesdendropilosa* (Symon) A.R.Bean**	**Papua New Guinea**	No long-styled flowers seen; plants dioecious?
*Lycianthesimpar* (Warb.) Bitter	Indonesia (Papua, Papua Barat), Papua New Guinea	No long-styled flowers seen, only fruits; plants dioecious?
***Lyciantheskaernbachii* (Lauterb. & K.Schum.) Bitter**	**Papua New Guinea**	Flowers unisexual; plants dioecious
***Lyciantheslucens* S.Knapp**	**Papua New Guinea**	Flowers unisexual; plants dioecious
*Lycianthesmoszkowskii* (Bitter) Bitter	Indonesia (Papua, Papua Barat), Papua New Guinea	Flowers unisexual; plants dioecious
*Lycianthesmultifolia* (Merr. & L.M.Perry) A.R.Bean	Indonesia (Papua, Papua Barat), Papua New Guinea	Flowers unisexual; plants dioecious
*Lycianthesoliveriana* (Lauterb. & K.Schum.) Bitter	Indonesia (Papua, Papua Barat, Maluku), Papua New Guinea	Flowers unisexual; plants dioecious
*Lycianthesperanomala* (Wernham ex Ridl.) Bitter	Indonesia (Papua, Papua Barat), Papua New Guinea	Flowers unisexual; plants dioecious
*Lycianthesrantonnetii* (Carrière) Bitter	Cultivated and perhaps naturalised in Australia (also New Zealand)	Flowers bisexual; plants hermaphroditic
***Lycianthesrostellata* (Merr. & L.M.Perry) A.R.Bean**	**Papua New Guinea**	Flowers bisexual; plants hermaphroditic
***Lycianthesshanesii* (F.Muell.) A.R.Bean**	**Australia**	Flowers unisexual; plants dioecious
*Lycianthesvitiensis* (Seem.) A.R.Bean	Fiji, Papua New Guinea (Bougainville), Samoa, Tonga	Flowers unisexual; plants dioecious
***Lyciantheswollastonii* (Wernham) A.R.Bean**	**Indonesia (Papua)**	Flowers bisexual (only long-styled flowers seen); plants hermaphroditic?

In Australia *Lycianthesshanesii* is relatively well-collected, and in the Pacific, early collections from A.C. Smith are quite representative. Collecting density across the main island of New Guinea is highly uneven (see Fig. 1), with many more specimens coming from Papua New Guinea than from Indonesian Papua (Cámara-Leret et al. 2020). The distribution of *Lycianthes* in New Guinea follows this pattern, and I expect as collecting increases in Papua and Papua Barat (e.g., Mustaqim et al. 2022) both distributional records and new taxa of these fascinating plants will be uncovered for this floristically rich tropical island.

These species of *Lycianthes* are mostly plants of forests, often described as growing in the understory or in primary, undisturbed forests at a wide variety of elevations. Forests in which these taxa occur are often described on labels as “mossy” or “wet”, and individuals appear to be rare when encountered. The Australian endemic *L.shanesii* is unusual in that the forests where it occurs are semideciduous monsoon vine thickets, much drier than the forests on New Guinea where most of these other taxa occur. *Lycianthesbiflora* is unusual in the group as a weedy plant occurring mostly at forest edges, in clearings and along roads and field edges.

***Reproductive systems***. Most species of *Lycianthes* occurring in the Americas are hermaphroditic with both male and female function operational in the same flower (see Dean et al. 2020). While some species have been documented as self-incompatible (SI, e.g., Dean 2004) and others like *L.rantonnetii*, are likely to be SI based on lack of fruit set in cultivation, most species of *Lycianthes* have not been assessed for compatibility.

In contrast, most of the species treated here are clearly heterostylous and based on examination of herbarium specimens they appear to have long-styled and short-styled flowers on different plants, suggesting they are androdioecious or dioecious (see Table 2). Separation of function to different plants in these *Lycianthes* species has only been documented in the Pacific *L.vitiensis*, where both Reinecke (1898) and Smith (1991) suggested the species was androdioecious, with male and hermaphroditic individuals. Symon (1985) suggested that many of the New Guinea species were probably dioecious but was not able to confirm this in the field. In *Solanum*, species exhibiting this set of traits were first described as androdioecious (see Symon 1970, 1979), a breeding system in which individual plants have either only staminate or only hermaphroditic flowers. Further study, however, revealed that the putatively hermaphroditic flowers had inaperturate pollen that functioned not in fertilisation but as a pollinator reward in these buzz-pollinated plants where pollen is the only reward (see Anderson 1979; Anderson and Symon 1989; Knapp et al. 1998; Martine et al. 2009; Anderson et al. 2015). All species of *Solanum* previously described as androdioecious have been shown to be dioecious, sometimes cryptically so (Anderson et al. 2015) with the floral morphology of staminate and pistillate flowers not markedly different. Staminate and pistillate plants of these cryptically dioecious species (e.g., *S.appendiculatum* Dunal, see Anderson 1979) were often described as different species, based on small differences in flower size. Dioecy has evolved in several *Solanum* lineages, and in some cases appears to be associated with islands (Anderson et al. 2015).

The putatively dioecious species of *Lycianthes* from Australia, New Guinea and the Pacific are like the cryptically dioecious species of *Solanum* in having staminate (male) and pistillate (female) flowers of very similar morphology, differing only in style length and occasionally in flower size (e.g., *L.kaernbachii*, *L.vitiensis*). Here too some of these plants have been described as distinct taxa based on small differences in flower size or lack of berries (e.g., *S.ledermannii* Bitter, *S.schlechterianum* Bitter). Field confirmation of the breeding system of these *Lycianthes* species is lacking, however, but from specimen evidence it appears that dioecy is the common state, especially on New Guinea. Many of the dioecious solanums exhibit “leaky” dioecy, where staminate flowers occasionally set fruit, perhaps enhancing their ability as colonists, either on islands (Anderson et al. 2015) or in new habitats (McDonnell et al. 2019). That there is a concentration of dioecious species of *Lycianthes* on the world’s largest tropical island means that these species perhaps represent an island radiation of dioecious taxa, like that seen in the spiny solanums of northern Australia (see Martine et al. 2009; Anderson et al. 2015). Field observations and studies of these largely rare forest plants are a priority for establishing the extent, degree, and evolution of the dioecious sexual system in *Lycianthes*.

***Conservation status***. A few of these species are widespread and relatively well-collected (e.g., *Lycianthesbiflora*, *L.vitiensis*), while most are poorly collected, and their distribution is not well understood. Forests on the island of New Guinea are all threatened, and land under protection is relatively small, though increasing (Parsch et al. 2022). Preliminary conservation assessments for all species except the cultivated *L.rantonnetii* are presented in Table 3 and detailed in individual species treatments. None of these species have been formally assessed for the IUCN Red List.

**Table 3.** Preliminary conservation assessments for the *Lycianthes* species of Australia, New Guinea and the Pacific. Abbreviations for threat status follow IUCN (2020). For discussion see individual species treatments.

Species	EOO (km^2^)	AOO (km^2^)	Threat status (suggested here)
*Lycianthesbambusarum* (Bitter) Bitter	7,346	44	EN
*Lycianthesbelensis* (Merr. & L.M.Perry) A.R.Bean	10,187	16	EN
*Lycianthesbiflora* (Lour.) Bitter	3,560,380	108	LC
*Lycianthesbitteriana* (Symon) A.R.Bean	22	12	CR
*Lycianthescladotrichota* (Bitter) Bitter	254,412	48	VU
*Lycianthesdendropilosa* (Symon) A.R.Bean	0	8	CR
*Lycianthesimpar* (Warb.) Bitter	257,568	48	VU
*Lyciantheskaernbachii* (Lauterb. & K.Schum.) Bitter	8,882	24	EN
*Lyciantheslucens* S.Knapp	70,602	24	VU
*Lycianthesmoszkowskii* (Bitter) Bitter	165,342	80	VU
*Lycianthesmultifolia* (Merr. & L.M.Perry) A.R.Bean	1.954	12	EN
*Lycianthesoliveriana* (Lauterb. & K.Schum.) Bitter	1,006,290	156	LC/NT
*Lycianthesperanomala* (Wernham ex Ridl.) Bitter	189,160	16	VU
*Lycianthesrantonnetii* (Carrière) Bitter	–	–	Not assessed
*Lycianthesrostellata* (Merr. & L.M.Perry) A.R.Bean	94,873	84	LC/NT
*Lycianthesshanesii* (F.Muell.) A.R.Bean	349,397	196	LC
*Lycianthesvitiensis* (Seem.) A.R.Bean	1,758,710	288	LC
*Lyciantheswollastonii* (Wernham) A.R.Bean	0	8	CR

## ﻿Species concepts

My goal for this revision has been to provide circumscriptions for the members of this rarely collected and sometimes morphologically variable group of species. Delimitation of species here follows what is known as the “morphological cluster” species concept (Mallet 1995; Knapp 2008): i.e., “assemblages of individuals with morphological features in common and separate from other such assemblages by correlated morphological discontinuities in a number of features” (Davis and Heywood 1963). Biological (Mayr 1982), phylogenetic (Cracraft 1989) and the host of other finely defined species concepts (see Mallet 1995) are almost impossible to apply in practice and are therefore of little utility in a practical sense (see Knapp 2008). It is important, however, to clearly state the criteria for the delimitation of species, rather than dogmatically follow particular ideological lines (see Luckow 1995; Davis 1997). My decisions relied on clear morphological discontinuities to define the easily distinguished species. The probable dioecious nature of many of these species has complicated species recognition; male and female (or hermaphroditic) plants can have flowers of slightly different sizes, and the lack of fruit in many collections makes them difficult to place. Specific characters used for recognition are detailed with each species description and in the key. I have tried to emphasise similarities between populations instead of differences, which so often reflect incomplete collecting or local variation, especially in a complex landscape like New Guinea. The approach has been relatively conservative, defining as distinct entities those population systems (sets of specimens) that differ in several morphological characteristics. In some species (e.g., *L.oliveriana*) variation exists in certain characters, but inspection of large number of specimens reveals no apparent natural breaks in variation but rather a mixing between highly morphologically variable populations. Here the pattern of variation is such that no reliable units can be consistently extracted, nor is geography a completely reliable predictor of character states. Here variability within and between populations seems more important than the variations of the extremes other taxonomists have recognised as distinct. This variation is described, while understanding that others may wish to interpret it differently.

## ﻿Materials and methods

This revision is based on examination of herbarium material from 1,241 collections (2,365 specimens, of which 1,058 are from Australia, New Guinea and the Pacific) in the following 64 herbaria (herbarium acronyms follow Index Herbariorum, found on-line at http://sweetgum.nybg.org/science/ih/): A, AD, AHUC, AK, ALCB, BISH, BM, BO, BR, BRI, BSHC, C, CAL, CANB, CAS, CHR, CNS, CORD, CTES, DAV, DD, DNA, E, FLAS, FUEL, G, G-DC, GH, GZU, HBG, IAC, K, KAG, KUN, L. LAE, LE, MBA, MBK, MBM, MEL, MO, MPU, NDG, NSW, NY, NZFRI, P, PBL, PERTH, PH, QRS, SI, SING, SP, TI, UBC, UC, US, USM, W, WAG, WELT, WIS. The complete data set cited includes records for the widely distributed *Lycianthesbiflora* from across its entire range. Digital images of collections were consulted to add duplicates and new records; I have only included citations of digital images of which I am certain of the identification. The on-line resources at the Naturalis Biodiversity Research Center (https://bioportal.naturalis.nl/; U, L and WAG) and the Papua New Guinea Forest Research Institute (LAE, available at https://www.pngplants.org/) have been used extensively. Specimen records for *L.rantonnetii* and *L.shanesii* occurring in Australia were downloaded from the Atlas of Living Australia (Atlas of Living Australia occurrence download at https://biocache.ala.org.au/occurrence/search?q=lsid%3Ahttps%3A%2F%2Fid.biodiversity.org.au%2Fnode%2Fapni%2F2913725&qualityProfile=ALA&disableQualityFilter=spatially-suspect&disableQualityFilter=duplicates accessed on 17 February 2022 and https://biocache.ala.org.au/occurrence/search?q=lsid%3Ahttps%3A%2F%2Fid.biodiversity.org.au%2Ftaxon%2Fapni%2F51303940&qualityProfile=ALA&disableQualityFilter=duplicates accessed on 18 February 2022) , cleaned, and all georeferences were checked.

An index to all numbered collections seen for this revision is presented in the Suppl. material 1 in pdf format. Searchable csv files of specimens examined are included as Suppl. material 2 (specimens for Australia, New Guinea and the Pacific only); Suppl. material 3 (all specimens examined). All of these files are also provided on the NHM Data Portal (https://doi.org/10.5519/j1y41fl4).

Measurements were made from dried herbarium material, with supplemental information on colour and texture taken from specimen labels. Specimens with coordinates on the labels were mapped directly, while the rest were georeferenced using the available locality data, sometimes supplemented by available collecting itineraries (e.g., Ridley 1916; van Steenis-Kruseman 1985). Maps were constructed with points in the centres of degree squares in a 1° square grid. Conservation threat status was assessed following the IUCN Red List categories and criteria (IUCN 2020) using the on-line assessment tools in GeoCat (http://geocat.kew.org; see Bachmann et al. 2011). The Extent of Occurrence (EOO) measures the range of the species, while the Area of Occurrence (AOO) represents the number of collecting points within the range based on a default grid of 2 km^2^; the AOO is highly sensitive to collecting bias, a known issue with plants of New Guinea.

Many of the names for New Guinea *Lycianthes* coined by Georg Bitter are based on type specimens that were destroyed in the bombing of the Berlin herbarium (B) in the 1940s (see Vorontsova and Knapp 2010). Duplicates of some of these have been found and lectotypes selected from amongst these, and where I have not found duplicates I have neotypified these names with widely distributed collections that are congruent with the original descriptions and are from as near to the same area as the destroyed type specimen as possible. I have given preference to specimens held in herbaria in Indonesia or Papua New Guinea. Given the paucity of collections of these plants, this has not always been possible. Names based on collections made by Carl Ledermann (see Ledermann 1919) are particularly challenging, as many of the localities he visited have never or rarely been accessed again (Veldkamp et al. 1988; Takeuchi and Golman 2002). All names coined by Merrill and Perry (1949) have holotypes at A, although no herbaria are cited in the descriptions. In the first paper in the series of “PlantaePapuanaeArchiboldianae” (Merrill and Perry 1939), they clearly state “The types of new species herein described, unless otherwise stated, are deposited in the herbarium of the Arnold Arboretum”.

I have cited barcodes or accessions numbers where available for all type specimens. Barcodes are cited as they are read with a barcode reader, either with or without the herbarium acronym (e.g., K000224089, L.4156113 where the acronym is part of the barcode, or 00050681 for US where it is not). Accession numbers are cited as “acc. #” (e.g., LAE acc. # 254856) and if a specimen has both a barcode and an accession number both are cited, with the barcode first.

For several of the species treated here Symon (1985) stated “type” or “holotype” and cited a single herbarium, inadvertently lectotypifying these names (Prado et al. 2015). I have indicated Symon’s original citation in square brackets.

Citation of literature follows BPH-2 (Bridson 2004) with alterations implemented in IPNI (International Plant Names Index, http://www.ipni.org) and Harvard University Index of Botanical Publications (http://kiki.huh.harvard.edu/databases/publication_index.html). Following Knapp (2013) we have used the square bracket convention for publications in which a species is described by one author in a publication edited or compiled by another, the traditional “in” attributions such as Dunal in DC. for those taxa described by Dunal in Candolle’s *Prodromus Systematis Naturalis Regni Vegetabilis*. This work is cited here as Prodr. [A.P. de Candolle] and the names are thus attributed only to Dunal. Standard forms of author names are according to IPNI (International Plant Names Index, http://www.ipni.org).

## ﻿Taxonomic treatment

### ﻿ Lycianthes

Taxon classificationPlantaeSolanalesSolanaceae

(Dunal) Hassl., Annuaire Conserv. Jard. Bot. Geneve 20: 180. 1917 nom. cons.

E204EC33-AB2B-5B50-9E6B-D7D6C017EE5F

*
Otilix* Raf., Medical Fl. 2: 87. 1830, nom. utique rej. Type species. Lyciantheslycioides (L.) Hassl. (as Solanumlycioides L.)
Solanum subsect. Lycianthes Dunal, Prodr. [A.P. de Candolle] 31(1): 29. 1852. Type species (designated by D’Arcy 1972, pg. 211). Lyciantheslycioides (L.) Hassl. (as Solanumlycioides L.)
Parascopolia Baill., Hist. Pl. 9: 338. 1888, nom utique rej. Type species. Lycianthesacapulcensis (Baill.) D’Arcy (as Parascopoliaacapulcensis Baill.)

#### Description.

Perennial herbs, shrubs, vines, lianas or trees, sometimes epiphytic. Stems terete or angled, glabrous or pubescent with simple (unbranched), forked, dendritic or stellate trichomes, these usually eglandular, but sometimes glandular. New growth usually with papillae, these sometimes glandular. Sympodial units unifoliate or difoliate, if difoliate the leaves geminate and often differing in both size and shape (anisophyllous). Leaves simple, entire, glabrous or pubescent with simple (unbranched), forked, dendritic or stellate trichomes, these usually eglandular, but sometimes glandular; petioles well-developed or not. Inflorescences axillary or adnate to the stems and caulescent (*L.kaernbachii* only), fasciculate or with a short rhachis; pedicels articulated at the base. Flowers 4–6-merous, usually 5-merous, but some species (e.g., *Lycianthesbanahaensis* (Elmer) Bitter of the Philippines) consistently 4-merous, perfect or heterostylous, long- and short-styled flowers borne on the same or different plants (in Australia, New Guinea and the Pacific probably dioecious). Calyx with a truncate rim, with various numbers (usually multiples of five, but sometimes fewer) of appendages protruding from the calyx tube below or just at the rim, or without appendages; appendages small bumps to linear subulate in shape. Corolla rotate to deeply stellate, white to deep purple (yellow in *L.banahaensis*), often with the midvein of the lobes darker and the centre paler or yellow-green, interpetalar tissue present or absent, the lobes minute (rotate corollas) or long-triangular, spreading, cupped or reflexed at anthesis. Stamens equal or unequal, if unequal due to anther and/or filament differences; anthers plumply ellipsoid and obovate to tapering at the tips, usually dehiscing by apical pores, these sometimes opening to longitudinal slits with age. Ovary conical or globose, glabrous; style straight or curved, the stigma minutely capitate, clavate or strongly bifid with diverging lobes. Fruit a berry, globose to ellipsoid to ovoid, green, orange, red or purple, sometimes with stone cells in the mesocarp. Seeds few to many, usually flattened, sometimes winged (e.g., *L.moszkowskii*). Chromosome number: n=1224 (few species have chromosome counts).

#### Distribution and ecology.

Species of *Lycianthes* are found in the Americas, Asia, Australia, New Guinea and the islands of the Pacific. Species richness is concentrated in Mexico and Central America. No *Lycianthes* species are native to Africa, Europe or North America north of Mexico.

#### Discussion.

By far the greatest species diversity in *Lycianthes* occurs in the Americas (see Dean et al. 2020), but significant diversity occurs on the island of New Guinea (treated here). The description above attempts to cover variation across the distribution, both in the Americas and in the eastern Hemisphere. Ongoing work on the species of Asia (Japan, Philippines and Indonesia to India) may reveal new character states not included above.

As discussed above, the species treated here are mostly (excepting the widespread and weedy *L.biflora*) not found elsewhere in Asia, and although they may not be a phylogenetically distinct group, they are geographically logical to treat as a unit. The species treated here are, with a few exceptions, very rarely collected and there are many gaps in our knowledge of both their distribution and morphology. Field observations indicate this is not just due to under-collecting, but that these plants are rare where they do occur; this is often the case for large woody lianas and epiphytes of primary forests (e.g., Orejuela 2021).

### ﻿Artificial key to the species of *Lycianthes* in Australia, New Guinea and the Pacific

**Table:** 

1	Young stems and/or upper leaf surfaces with branched trichomes	**2**
–	Young stems and/or upper leaf surfaces glabrous or with only simple (unbranched) trichomes	**7**
2	Trichomes with short, congested branches (Christmas-tree like or “tannenbaumartig”)	**3**
–	Trichomes forked (with a single branch) or dendritic (antler-like)	**4**
3	Flowers 10–15 per axillary fascicle; calyx with awl-shaped appendages to 1 mm long	***Lycianthesbitteriana***
–	Flowers 1–3 per axillary fascicle; calyx without appendages	***Lycianthesdendropilosa***
4	Calyx truncate, with no appendages	**5**
–	Calyx with 5–10 linear, awl shaped-shaped appendages	**6**
5	Flowers and fruits in axillary fascicles; corolla 1–1.3 cm in diameter; anthers ca. 3 mm long	***Lycianthescladotrichota***
–	Flowers and fruits borne along the stem between the nodes; corolla 0.8–0.9 cm in diameter; anthers 1.5–2 mm long	***Lyciantheskaernbachii***
6	Corolla deeply stellate, with little or no interpetalar tissue; berry bright red when ripe, globose; stone cells absent; stems terete; wild plants, New Guinea	***Lycianthesbiflora***
–	Corolla rotate, with abundant interpetalar tissue; berry yellowish orange when ripe, ellipsoid; stone cells present; stems angled and often somewhat striped; cultivated plants, Australia	***Lycianthesrantonnetii***
7	Calyx with appendages of various shapes and sizes (can be very small and only visible in bud, *L.bambusarum*)	**8**
–	Calyx truncate, with no appendages	**14**
8	Calyx appendages linear and awl-shaped, usually 10	**9**
–	Calyx appendages smaller, not linear, often only nubs on the calyx tube below the rim	**10**
9	Corolla deeply stellate, with little or no interpetalar tissue; berry bright red when ripe, globose; stone cells absent; stems terete; wild plants, New Guinea	***Lycianthesbiflora***
–	Corolla rotate, with abundant interpetalar tissue; berry yellowish orange when ripe, ellipsoid; stone cells present; stems angled and often somewhat striped; cultivated plants, Australia	***Lycianthesrantonnetii***
10	Young stems moderately to densely pubescent with soft curled (“crisped”) trichomes, these often somewhat antrorse	**11**
–	Young stems glabrous or with only a few trichomes or papillae	**12**
11	Corolla ca. 2.4 cm in diameter; calyx rim hyaline and markedly undulate (“ruffly”), the appendages, if present, arising well below; anthers 2.5–3 mm long	***Lycianthesbelensis***
–	Corolla 1.4–1.6 cm in diameter; calyx rim not markedly hyaline, the appendages arising from the rim; anthers ca. 1 mm long	***Lycianthesmultifolia***
12	Calyx appendages less than 1 mm long, often visible only in bud, parallel to the calyx tube; major leaves narrowly elliptic or lanceolate; corolla lacking interpetalar tissue; seeds with a deep notch	***Lycianthesbambusarum***
–	Calyx appendages greater than 1 mm long, triangular, perpendicular to the calyx tube; major leaves elliptic; corolla with some interpetalar tissue; seeds lacking a deep notch	**13**
13	Leaves with the midrib not keeled; flowering pedicels 2.5–4 cm long; anthers 2.5–3 mm long. New Ireland, Lihir Island, Louisiade Archipelago	***Lyciantheslucens***
–	Leaves with the midrib keeled; flowering pedicels 1.8–2.2 cm long; anthers 4.5–5 mm long. Mount Jaya, Papua, Indonesia	***Lyciantheswollastonii***
14	Small trees; calyx irregular and splitting into apparent lobes	**15**
–	Shrubs, vines or epiphytes; calyx truncate, not splitting into apparent lobes	**16**
15	Leaves elliptic-ovate, widest in the upper half; pubescence of new growth and stems near the inflorescences pale golden; pedicels and calyces glabrous or with a few scattered trichomes; anthers 4–4.5 mm long, tapered at the tips. Queensland, Australia	***Lycianthesshanesii***
–	Leaves elliptic, widest at the middle; pubescence of new growth and stems near the inflorescences reddish gold or rusty brown; pedicels and calyces uniformly and minutely pubescent; anthers 2.5–4 mm long, if tapered only slightly so, Pacific, Bougainville to Samoa	***Lycianthesvitiensis***
16	Flowers and fruit borne along the stem between the nodes in parallel lines	***Lyciantheskaernbachii***
–	Flowers and fruit borne in axillary fascicles or small rachises	**17**
17	Trichomes of young stems and leaves stiff and strongly antrorse; leaves of a geminate pair always differing in size and shape	**18**
–	Trichomes of young stems and leaves soft and curling (“crisped”) or absent, not stiff; leaves of a geminate pair, if present, not always differing in shape	**20**
18	Leaves obovate, widest in the upper half; corolla 2–2.5 cm in diameter; berry 1.2–1.5 cm in diameter; seeds markedly winged	***Lycianthesmoszkowskii***
–	Leaves elliptic or narrowly elliptic, widest at the middle; corolla less than or equal to 2 cm in diameter; berry less than 1.5 cm in diameter; seeds not winged	**19**
19	Flowers with the corolla 1.4–2 cm in diameter; anthers 5–6 mm long; stem trichomes often with multicellular bases; seeds with a deep notch	***Lycianthesrostellata***
–	Flowers with the corolla 0.6–0.8 cm in diameter; anthers 1.5–2 mm long; stem trichomes without multicellular bases; seeds without a deep notch	***Lycianthesperanomala***
20	Flowers and fruits borne on a short rachis, not strictly fasciculate; minor leaves markedly different in size and shape from the major leaves; berry elongate	***Lycianthesimpar***
–	Flowers and fruits strictly fasciculate; minor leaves, if present, different in size but not markedly in shape from major leaves; berry globose	**21**
21	Fascicles with more than 4 flowers (often many flowered); anthers 2–2.5 mm long, slightly obovoid; fruiting calyx cupping the berry, strongly tuberculate	***Lycianthesoliveriana***
–	Fascicles few-flowered; anthers more than 2.5 mm long, somewhat tapered at the tips; fruiting calyx spreading beneath the berry, not cupping nor strongly tuberculate (warty)	**22**
22	Shrubs; leaves with the veins puberulent abaxially; leaf venation not markedly anastomosing before the margins; calyx rim hyaline and undulate (“ruffly”); corolla ca. 2.4 cm in diameter; anthers 2.5–3 mm long	***Lycianthesbelensis***
–	Lianas or epiphytes (occasionally shrubs on labels); leaves completely glabrous; leaf venation markedly anastomosing before the margins; calyx rim truncate, not hyaline and “ruffly”; corolla 0.8–1.2 cm in diameter; anthers 4–4.5 mm long	***Lycianthesbambusarum***

### ﻿Synoptic character list for *Lycianthes* in Australia, New Guinea and the Pacific

This synoptical character list can be used as a multi-entry key for identification. I have only listed diagnostic characters here, for example, inflorescences with more than 10 flowers, but not the more general case of few-flowered. For detailed distributional information please see Table 2. The list is intended to be used as a tool via a process of elimination; any character can be selected and in combination with other characters, a smaller selection of species can be obtained, for which the descriptions will be useful for coming to a final identification.

Plants of Australia: rantonnetii, shanesii

Plants of the island of New Guinea: bambusarum, belensis, biflora, bitteriana, cladotrichota, dendropilosa, impar, kaernbachii, moszkowskii, multifolia, oliveriana, peranomala, rostellata, wollastonii

Plants of islands East of New Guinea: biflora, lucens, vitiensis

Plants found in cultivation: rantonnetii

Trees with a single trunk: shanesii, vitiensis

Woody lianas: bambusarum, cladotrichota, kaernbachii, oliveriana

Shrubs: belensis, biflora, bitteriana, lucens, moszkowskii (?), multifolia

Stem trichomes stiff and markedly antrorse: moszkowskii (sometimes), peranomala, rostellata

Leaves >10 cm long: kaernbachii, oliveriana

Leaves of a geminate pair markedly different in both size and shape: cladotrichota, dendropilosa, impar, kaernbachii, moszkowskii, multifolia, peranomala, rostellatum, wollastonii

Mature leaves with branched Christmas-tree like trichomes: bitteriana, dendropilosa

Mature leaves with branched antler-like trichomes: biflora, cladotrichota, kaernbachii (rarely), rantonnetii (rarely)

Inflorescence adnate to the stem and caulescent, in two rows along stem between the nodes: kaernbachii

Inflorescence with a distinct rhachis: bitteriana, impar, vitiensis

Inflorescence with more than 10 flowers: cladotrichota, impar, kaernbachii, oliveriana, vitiensis

Calyx lacking appendages: bambusarum (rarely), belensis, impar, kaernbachii, moszkowskii, oliveriana, peranomala, rostellata, shanesii, vitiensis

Calyx appendages linear and awl-shaped: biflora, rantonnetii

Calyx appendages triangular and perpendicular to the calyx tube: lucens, wollastonii

Corolla rotate, with abundant interpetalar tissue: rantonnetii

Corolla stellate, interpetalar tissue completely absent: cladotrichota, impar, kaernbachii, oliveriana, peranomala

Anthers plumply ellipsoid or obovate: kaernbachii, oliveriana, peranomala

Anthers opening by longitudinal slits: vitiensis

Anthers bright orange: rantonnetii

Berry yellowish orange when ripe: rantonnetii

Berry purple or black when ripe: bitteriana, impar

Fruiting calyx cupping the base of the fruit (acorn-like): olivieriana

Seeds winged: moszkowskii

Seeds with a prominent hilar notch: bambusarum, rostellatum, vitiensis

### ﻿Species descriptions

### ﻿1. Lycianthes bambusarum

Taxon classificationPlantaeSolanalesSolanaceae

(Bitter) Bitter, Abh. Naturwiss. Vereins Bremen 24 [preprint]: 503. 1919.

4A9415A7-7AA4-5747-8D4B-FE948A1F1404

Figs 4 , 5

Solanum bambusarum Bitter, Bot. Jahrb. Syst. 55: 91. 1917. Type. Papua New Guinea. Madang: “Schraderberg” [Schrader Mountain], 1,900–2,000 m, May-Jun 1913, *C.L. Ledermann 12129* (holotype: B [destroyed], no duplicates found). Papua New Guinea. Chimbu: Crater Mountain Wildlife Management Area, vicinity of Haia, along the Wara oo streamcourse (first river E of Mt.Widau), 640 m, 6 Mar 1997, *W.N. Takeuchi 11704* (neotype, designated here: LAE [acc. # 279948]; isoneotypes: K [K000224089, K000449027], L [L.4156113]).
Solanum umbonatum Symon, J. Adelaide Bot. Gard. 8: 63. 1985. Type. Papua New Guinea. Morobe: Edie Creek, about 4 miles (6.4 km) SW of Wau, 1,829 m, 26 Apr 1963, *T.G. Hartley 11756* (holotype: CANB [CANB151116]; isotypes: A, BRI [BRI-AQ0080263], K [K001153711], L [L0003674, L.2874714], LAE [acc. # 64346]).
Lycianthes umbonata (Symon) A.R.Bean, Austrobaileya 6(3): 568. 2003. Type. Based on Solanumumbonatum Symon.

#### Type.

Based on *Solanumbambusarum* Bitter.

**Figure 4.**
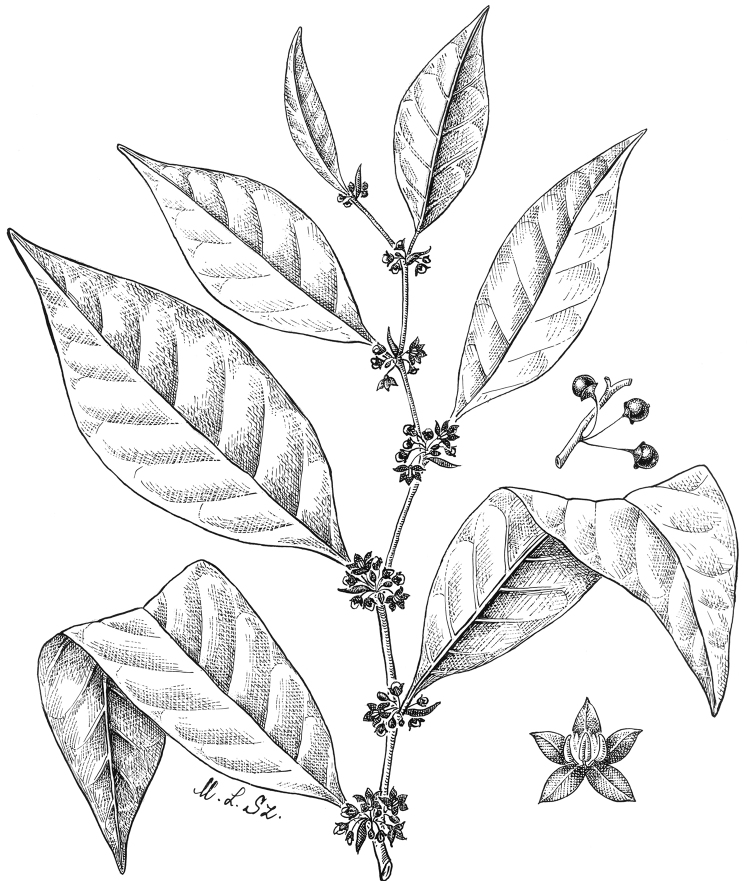
*Lycianthesbambusarum* (Bitter) Bitter. Drawing by M.L. Szent-Ivany, first published in Symon (1985: fig. 23, as *S.umbonatum* Symon). Courtesy of the Board of the Botanic Gardens and State Herbarium (Adelaide, South Australia), reproduced with permission.

#### Description.

Shrubs, scrambling shrubs, lianas or epiphytes, to 3.5 m tall (long); stems terete, glabrous; new growth minutely puberulent with tiny usually single-celled papillate trichomes less than 0.1 mm long, soon glabrous; bark of older stems pale beige, somewhat corky and peeling. Sympodial units unifoliate, the leaves not geminate. Leaves simple; blades 4–11 cm long, 2–4.5 cm wide, narrowly elliptic to lanceolate, less commonly elliptic (i.e., *Craven & Schodde 1258*), slightly discolorous, membranous to chartaceous; adaxial and abaxial surfaces glabrous, the midrib somewhat keeled adaxially; principal veins 4–5 pairs, prominently anastomosing in arches before the margins; base acute to more commonly attenuate; margins entire; apex acuminate; petiole 0.6–1.2 cm long, glabrous. Inflorescences axillary fascicles of 1–4 (rarely to 8–10, e.g., *Hartley 11434*) flowers, only 1–2 open at a time, completely glabrous; pedicels (0.6 in bud) 1–1.2 cm long at anthesis, ca. 1 mm in diameter at the base, ca. 1.5 mm in diameter at the apex, glabrous or minutely papillate near the base, articulated at the base; pedicel scars tightly packed in leaf axils. Buds globose, becoming ellipsoid, the corolla exserted halfway from the calyx tube just before anthesis. Flowers 4–5-merous, unisexual and heterostylous, the flowers on individual plants apparently all either short-styled or long-styled and the plants dioecious (needs field testing). Calyx with the tube 2.5–3 mm long, 4–5 mm in diameter, cup-shaped, glabrous or minutely papillate, apparently fleshy, purple, with 4–5 small umbonate appendages to ca. 0.5 mm long or the appendages absent, the rim entire and extending for 0.25–0.5 mm beyond the appendages, the appendages more prominent in buds. Corolla 0.8–1.2 cm in diameter, purple, stellate, lobed 3/4 of the way to the base, interpetalar tissue absent, the lobes 4–5 mm long, 1.5–2 mm wide, erect to spreading, fleshy (stiff and woody in dried specimens), glabrous abaxially and adaxially but densely papillate on tips and margins, the tips cucullate. Stamens equal; filament tube minute; free portion of the filaments ca. 0.1 mm long, glabrous; anthers 4–4.5 mm long, 1.5–2 mm wide, ellipsoid, somewhat tapering at the tips, bright yellow, poricidal at the tips, the pores directed distally, not elongating to slits with age. Ovary conical, vestigial in short-styled flowers, glabrous; style in short-styled flowers less than 1 mm long, in long-styled flowers 4–6 mm long, straight, purple, glabrous; stigma bilobed, the surfaces minutely papillate. Fruit a globose berry, 0.6–0.7 cm in diameter, green (immature? – in *Streimann 9635* remnants of the style still at apex), the pericarp glabrous, thin, matte, opaque; fruiting pedicels 1–1.3 cm long, ca. 1 mm in diameter at the base, ca. 2 mm in diameter at the apex, erect or spreading, somewhat woody, purple or green; fruiting calyx a cup-like spreading plate beneath the berry, somewhat thickened and warty (fleshy in live plants?). Seeds 50–70 per berry, ca. 2.5 mm long, ca. 2.5 mm wide, round with a deep notch at the hilum, yellowish tan, the surfaces deeply pitted, the testal cells pentagonal in outline. Stone cells absent. Chromosome number not known.

**Figure 5.**
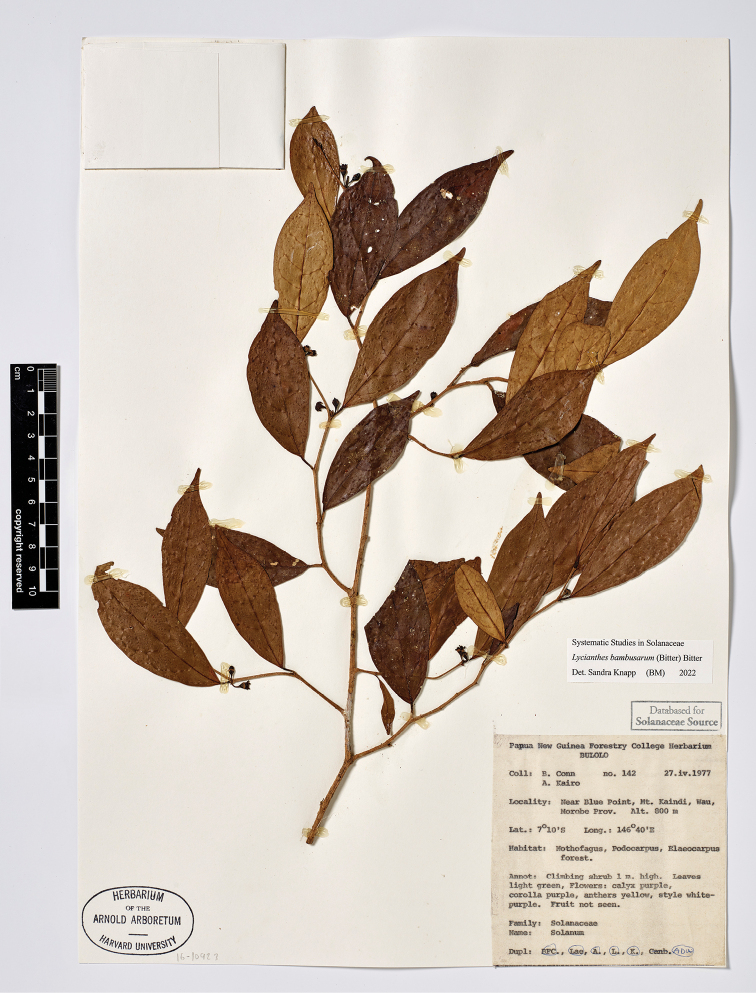
*Lycianthesbambusarum* herbarium specimen. Papua New Guinea. Morobe: *Conn & Kairo 142* (A). Courtesy of the Herbarium of the Arnold Arboretum of Harvard University, reproduced with permission.

#### Distribution

**Figure 6.**
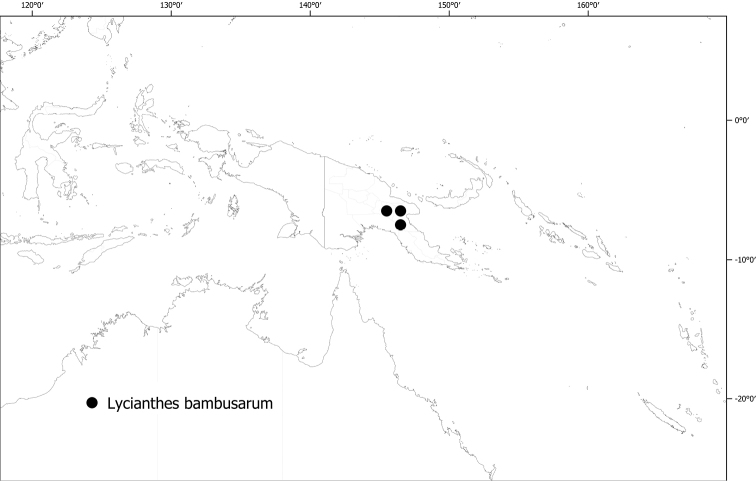
Distribution of *Lycianthesbambusarum*.

**(Fig. 6).***Lycianthesbambusarum* is endemic to the island of New Guinea; collected only from Papua New Guinea (Chimbu, Morobe, Madang [destroyed type only]).

#### Ecology and habitat.

*Lycianthesbambusarum* is a plant of montane forests with bamboo, *Pandanus* and/or *Nothofagus*, between 450 and 2,400 m elevation.

#### Common names.

None recorded.

#### Preliminary conservation assessment

(IUCN 2020). EOO (7,346 km^2^ - VU); AOO (44 km^2^ - EN). *Lycianthesbambusarum* is known from three localities; in light of this and the threats to forest habitats on New Guinea more generally, I propose a preliminary threat status of Endangered (EN [B1,2ab (iii, iv)]) for *L.bambusarum*.

#### Discussion.

*Lycianthesbambusarum* was distinguished by Bitter (1917) on the basis of its unifoliate sympodia, narrow leaves and 4-merous flowers; Symon (1985) recognised no other collections as this species, and described *L.umbonata* (as *S.umbonatum*) on the basis of its 5-merous flowers with distinct “umbos” on the calyx. The calyx appendages in *L.bambusarum* are very evident in bud (as in the type of *S.umbonatum*) but in flower are very small to non-existent (as shown in Bitter 1917: 92). Based on the otherwise widely overlapping descriptions I am here treating *L.umbonata* as a synonym of *L.bambusarum*.

*Lycianthesbambusarum* is similar to *L.rostellata* but differs from that species in being almost completely glabrous on stems and leaves, having unifoliate sympodia, and having heterostylous flowers. *Lycianthesrostellata* is distinctly pubescent, especially on the stems, with stiff, antrorse simple trichomes that often have multicellular bases, has small trowel-shaped minor leaves, and appears to have all bisexual flowers. The two taxa share narrow leaves and berries with numerous deeply notched seeds.

The type of *Lycianthesbambusarum* (as *S.bambusarum*) was collected by C.L. Ledermann in his explorations of the central mountain ranges of what is now Papua New Guinea (Ledermann 1919; Veldkamp et al. 1988; Takeuchi and Golman 2002). I have found no duplicates of this collection (*Ledermann 12129*), the original of which was cited as being in the herbarium in Berlin (Bitter 1917). These areas have rarely been accessed since Ledermann’s collecting, and I have seen no specimens from Madang that correspond to *L.bambusarum*. The collection (*Takeuchi et al. 11704*) selected here as a neotype matches the protologue, is from a similar elevation in the same mountain range and has a number of duplicates that are widely distributed, including in Papua New Guinea (where the neotype sheet is held in LAE).

#### Specimens examined.

Papua New Guinea. **Morobe**: near Blue Point, Mt. Kaindi, Wau, 800 m, 24 Apr 1977, *Conn & Kairo 142* (A, K); near Wengomanga, via Oiwa, Aseki Patrol Area, 11 Apr 1966, *Craven & Schodde 1258* (A, K, L, LAE, US); Wau, Mount Kaindi, contour trail in forest, 2,390 m, 10 Jul 1977, *Fallen 374* (L, LAE, MO); Tymne-Wago track, 457 m, 18 Mar 1963, *Hartley 11434* (A, K, L, LAE); Aseki-Spreader Div., Menyamya subdistrict, 1,800 m, 8 Jan 1972, *Stevens LAE-54766* (A, K, L, LAE, US); Ekuti Divide, Bulolo-Aseki road, 33 km WSW of Bulolo, 2,250 m, 17 Oct 1982, *Streimann 9635* (A, E, K, L, LAE); Angabena Ridge, ca. 3 km from Aseki-Menyamya Rd., Menyamya subdistrict, 1,675 m, 7 Jan 1972, *Streimann & Stevens LAE-53892* (A, K, L, LAE); Aseki road from Bulolo, subdistrict Wau, 2,300 m, 29 Jul 1977, *Symon 10632* (K, L, LAE, MO); Mount Kaindi, upper slopes of Mt. Kaindi, 2,000 m, 30 May 1984, *Symon & Katik 13822* (K, L, LAE, MO); Aseki road below the crest, 31 May 1984, *Symon & Katik 13829* (K, L, LAE, MO).

### ﻿2. Lycianthes belensis

Taxon classificationPlantaeSolanalesSolanaceae

(Merr. & L.M.Perry) A.R.Bean, Austrobaileya 6(3): 567. 2003.

4EA913D0-D23F-59F0-A3C5-8C5D128AB2B9

Figs 7 , 8

Solanum belense Merr. & L.M.Perry, J. Arnold Arb. 30: 50. 1949. Type. Indonesia: Papua: 18 km. NE of Lake Habbema, Bele River, 2,300 m, Nov 1938, *L.J. Brass 11223* (holotype: A [n.v.]; isotypes: BM [BM000778128], L [L0003624], LAE [acc. # 6543, acc. # 229595]).

#### Type.

Based on *Solanumbelense* Merr. & L.M.Perry.

**Figure 7.**
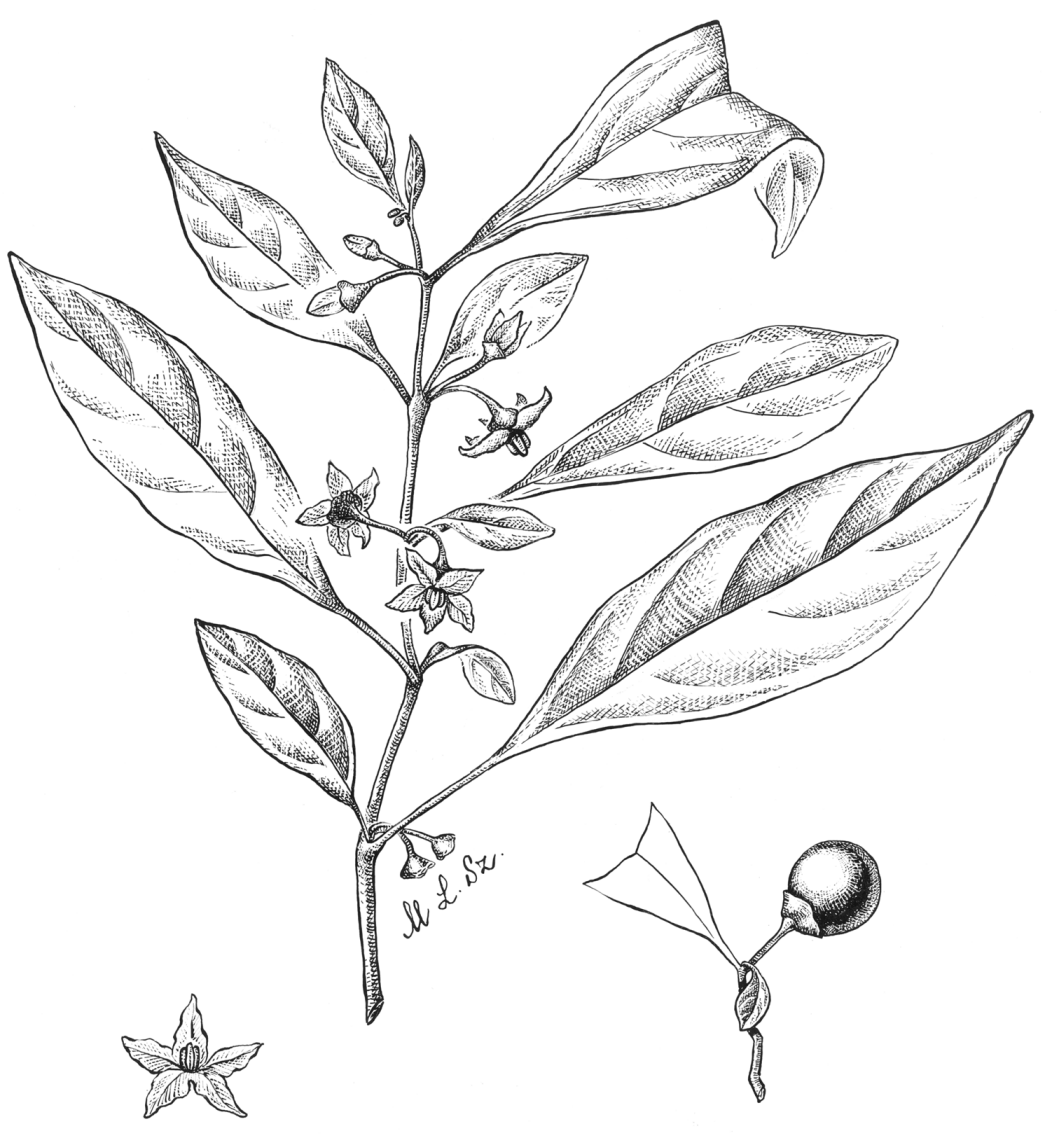
*Lycianthesbelensis* (Merr. & L.M.Perry) A.R.Bean. Drawing by M.L. Szent-Ivany, first published in Symon (1985: fig. 9, as *S.belense* Merr. & L.M.Perry). Courtesy of the Board of the Botanic Gardens and State Herbarium (Adelaide, South Australia), reproduced with permission.

#### Description.

Small shrubs to 1 m tall; stems terete, moderately pubescent with tiny glandular papillae and simple uniseriate 1–5-celled trichomes ca. 0.5 mm long, the trichomes transparent, weak-walled and collapsing, somewhat tangled; new growth densely pubescent with simple uniseriate trichomes like those of the stems, glabrescent; bark of older stems brown, glabrescent. Sympodial units difoliate, the leaves geminate, the leaves of a pair differing only in size, not in shape, the minor leaves often deciduous. Leaves simple; blades of major leaves 7–14 cm long, 3–6.5 cm wide, elliptic, widest in the middle, concolorous, membranous to chartaceous; adaxial surfaces glabrous; abaxial surfaces often purplish green, with the lamina glabrous and scattered simple uniseriate trichomes ca. 0.5 mm long on the veins and midrib; principal veins 4–6 pairs, puberulent beneath; base narrowly acute, margins entire; apex acute to abruptly acuminate; petiole 0.6–1(1.5) cm long, glabrous or with a few scattered trichomes like those of the stems; blades of minor leaves 2.5–4 cm long, 1.3–1.7 cm wide, in shape and pubescence like the major leaves; petioles 0.5–0.7 cm long. Inflorescences axillary fascicles of ca. 4 flowers, 1–2 open at a time, moderately pubescent like the stems; pedicels 0.7–2 cm long at anthesis, lengthening considerably at anthesis, ca. 0.5 mm in diameter at the base, ca. 1.5 mm in diameter at the apex, glabrous, articulated at the base; pedicel scars closely spaced in leaf axils. Buds ellipsoid, the corolla included in the calyx tube and exserted halfway just before anthesis. Flowers 5-merous, apparently unisexual and heterostylous, individual specimens with either short-styled or long-styled flowers, the plants possibly dioecious. Calyx tube 2.5–3 mm long, 2.5–3 mm in diameter, cup-shaped, glabrous with the rim minutely papillate, occasionally with up to 3 very small appendages, the rim transparent and ruffly, extending ca. 0.75 mm beyond the appendages (if present). Corolla ca. 2.4 cm in diameter, white or purple, stellate, lobed ca. 2/3 of the way to the base, a thin rim of interpetalar tissue present, the lobes ca. 3 mm long, ca. 0.9 mm wide, spreading, membranous, glabrous except for the densely papillate tips, margins and adaxial midvein. Stamens equal; filament tube minute; free portion of the filaments ca. (0.5) 3 mm long, glabrous; anthers 2.5–3 mm long, ca. 1.5 mm wide, ellipsoid and tapering, yellow, poricidal at the tips, the pores directed distally and not elongating with age. Ovary conical, glabrous; style in short-styled flowers less than 1 mm long, in long-styled flowers ca. 4 mm long (only seen in buds), straight, glabrous; stigma strongly bifid with diverging lobes ca. 0.25 mm long, the surfaces minutely papillate. Fruit a globose berry, ca. 1 cm in diameter, green (immature), the pericarp glabrous, relatively thin, matte, opaque; fruiting pedicels ca. 1.5 cm long, ca. 1 mm in diameter at the base, ca. 1.5 mm in diameter at the apex, green (?), erect or spreading, not markedly woody; fruiting calyx a spreading plate beneath the berry, somewhat fleshy except for the thinner rim. Seeds and stone cells not seen. Chromosome number not known.

**Figure 8.**
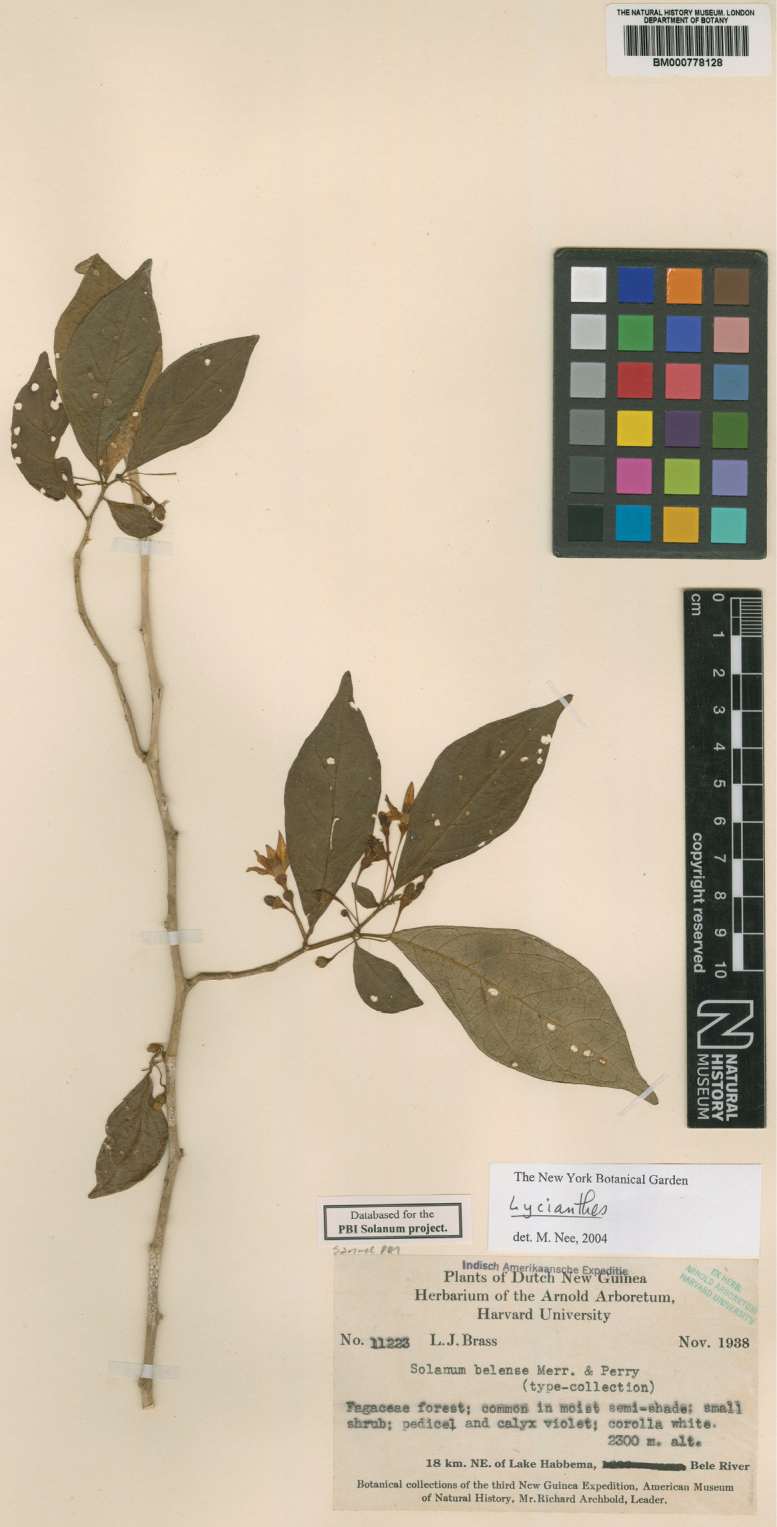
*Lycianthesbelensis* herbarium specimen. Indonesia. Papua: *Brass 11223* (isotype of *S.belense*, BM000778128). Courtesy of the Trustees of the Natural History Museum, London, reproduced with permission.

#### Distribution

**Figure 9.**
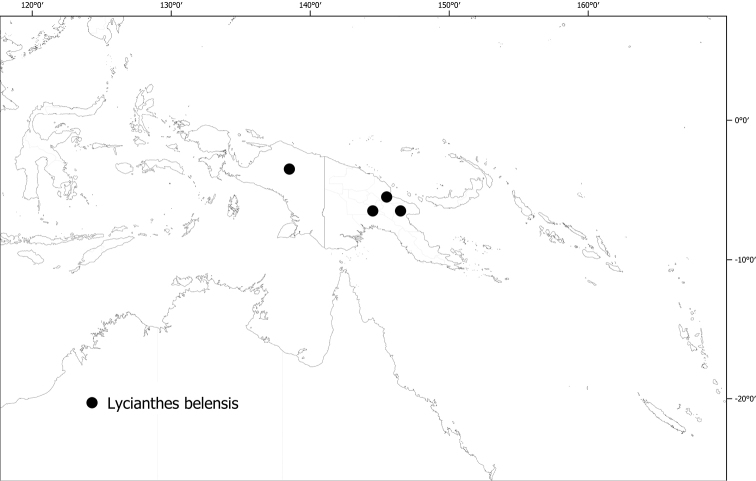
Distribution of *Lycianthesbelensis*.

**(Fig. 9).***Lycianthesbelensis* is endemic to New Guinea; found in Papua New Guinea (Chimbu, Eastern Highlands) and Indonesia (Papua).

#### Ecology and habitat.

*Lycianthesbelensis* is rarely collected but appears to be a plant of Fagaceae and *Castanopsis* forests, from sea level to around 2,300 m elevation.

#### Common names.

None recorded.

#### Preliminary conservation assessment

**(IUCN 2020).**EOO (10,187 km^2^ - VU); AOO (16 km^2^ - EN). *Lycianthesbelensis* is known from only three widely spaced localities, all in forested areas; it is very rarely collected and has not been collected recently. I propose a preliminary threat status of Endangered (EN [B2 a(iii, iv)]) for *L.belensis*.

#### Discussion.

There are very few collections of *Lycianthesbelensis* and the range of variation needs further examination with additional material. The type collection (*Brass 11223*) has somewhat larger flowers on longer pedicels than other material, but leaf shape, pubescence and seed morphology suggest these all correspond to the same taxon. Specimens here included in *L.lucens* from the islands in the Louisiade Archipelago have been identified as *L.belensis*. *Lycianthesbelensis* differs from *L.lucens* in its pubescent (versus glabrous) stems and its calyx with a thin, translucent ‘ruffly’ rim and 0–3 tiny appendages (versus a less obviously translucent rim with 3–5 triangular appendages emerging at right angles to the calyx tube).

*Lycianthesbelensis* is a plant of high elevations while *L lucens*, also a plant of montane forests, is found at lower elevations. Their phylogenetic relationship has not been tested.

#### Specimens examined.

Indonesia. [type only]

Papua New Guinea. **Chimbu**: Chimbu valley, Gembogl, Yei nigl, 2,700 m, 31 Jan 1981, *Sterly 80-469* (L); **Eastern Highlands**: Kassam, [Kassam Pass], 1,370 m, 3 Nov 1959, *Brass 32400* (LAE, US); Mount Gahavisuka, 2,250 m, 16 Mar 1984, *Cruttwell 2602* (L).

### ﻿3. Lycianthes biflora

Taxon classificationPlantaeSolanalesSolanaceae

(Lour.) Bitter, Abh. Naturwiss. Vereins Bremen 24 [preprint]: 461. 1919.

7F327725-F17E-53FD-98DD-A48704E95EC5

**Figure 10.**
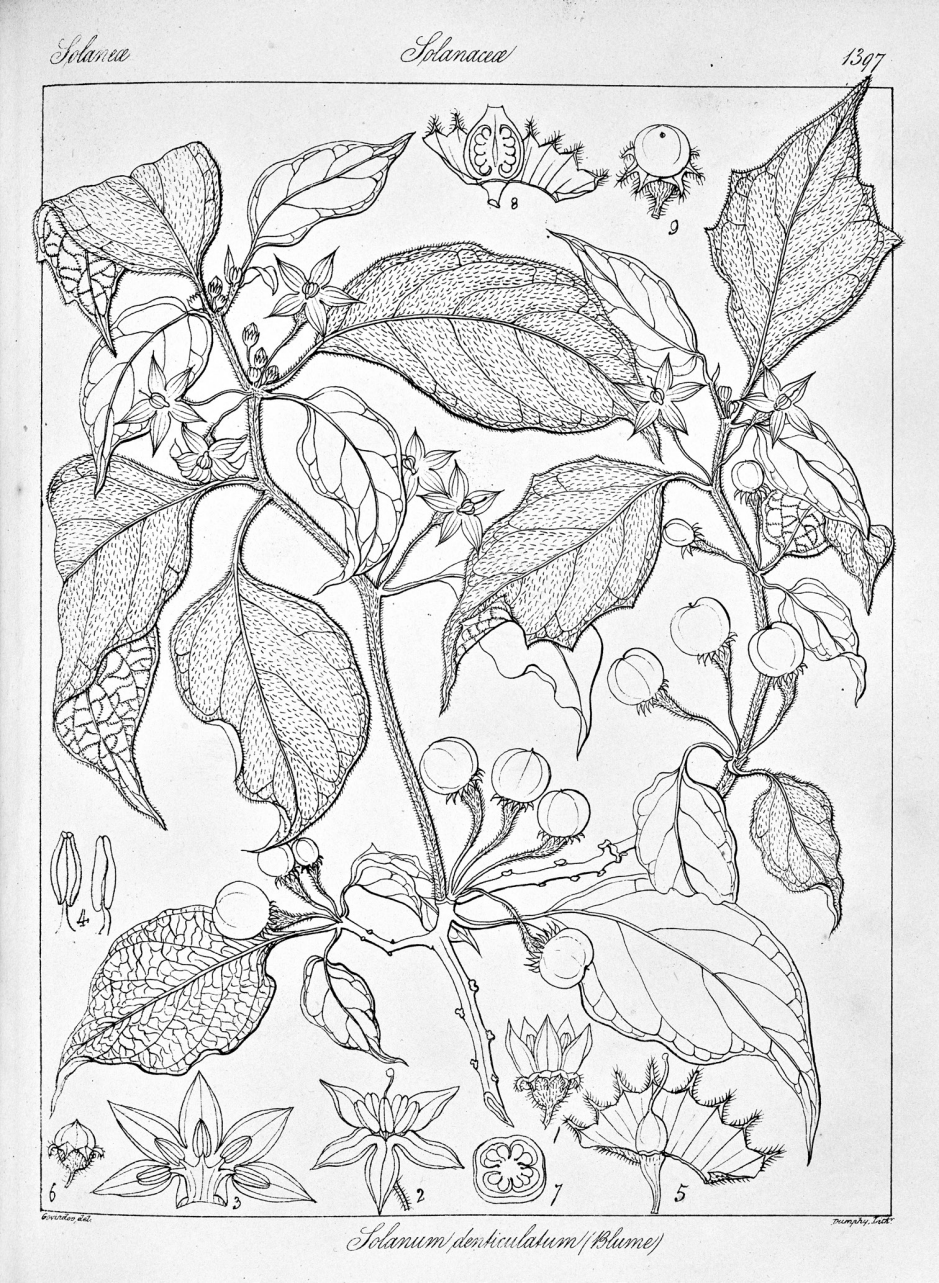
*Lycianthesbiflora* (Lour.) Bitter. Reproduced from Wight (1850: tab. 1397, as *S.denticulatum* Blume). Courtesy of the NHM Library and Archives reproduced with permission.

**Figure 11.**
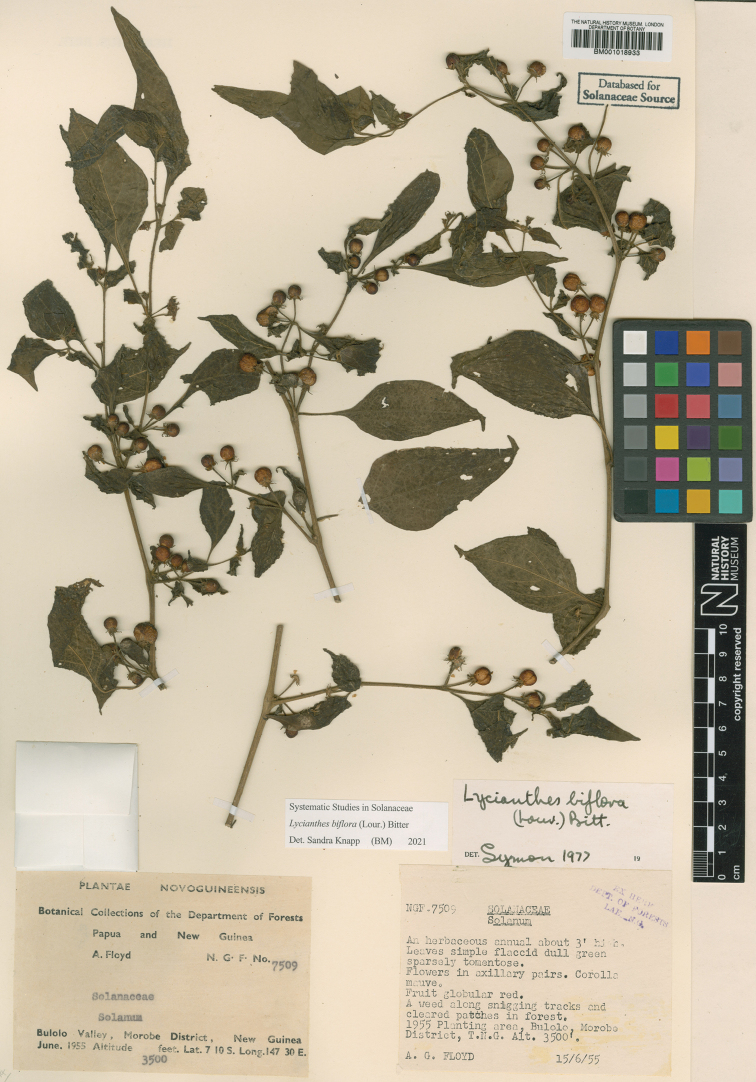
*Lycianthesbiflora* herbarium specimen. Papua New Guinea. Morobe: *Floyd NGF-7509* (BM001018933). Courtesy of the Trustees of the Natural History Museum, London, reproduced with permission.

Figs 3C , 3E , 10 , 11

Solanum biflorum Lour., Fl. Cochinch. 129. 1790. Type. China. Guangzhou: “Pakwan supra Cantonem”, Jul 1869, *H.F. Hance 2128* (neotype, designated by Hul and Dy Phon 2014, pg. 57: P [P00058796]; isoneotypes: P [P00058797]).

#### Type.

Based on *Solanumbiflorum* Lour.

#### Description.

Small shrubs or herbs, 0.5–1.5 m tall; stems terete, sparsely to densely pubescent with a mixture of transparent simple and/or forked or dendritic 3–10-celled uniseriate trichomes to 1 mm long, the dendritic trichomes antler-like or merely forked; new growth sparsely to densely pubescent with simple and dendritic trichomes like those of the stems, in plants with sparse pubescence the trichomes mostly confined to the leaf veins; bark of older stems pale brown, somewhat glabrescent. Sympodial units difoliate, the leaves geminate, the leaves of pair usually differing in size but not in shape. Leaves simple; blades of major leaves 5–16 cm long, 2.5–6.5 cm wide, ovate to elliptic, usually widest in the lower half but occasionally near the middle, somewhat discolorous, membranous; adaxial surfaces almost glabrous to evenly and moderately pubescent with transparent mixed simple and dendritic trichomes like those of the stems, these much denser along the veins; abaxial surfaces sparsely to moderately pubescent with the same trichomes as those of the adaxial surfaces, but the pubescence denser; principal veins 4–6 pairs, sparsely to densely pubescent, often drying yellow on both surfaces; base attenuate, markedly decurrent onto the petiole; margins entire, markedly ciliate with transparent and mixed simple and/or dendritic trichomes like those of the leaf surfaces; apex abruptly acuminate or acuminate; petiole 0.5–2.5 cm long, winged from the decurrent leaf bases, sparsely to densely pubescent like the stems and leaves; blades of minor leaves 2.5–5 cm long, 1.5–3 cm wide, shape, texture and pubescence like that of the majors; base attenuate onto the petiole; margins entire, ciliate; apex abruptly acuminate or acuminate; petiole 0.4–1(2.5) cm long, pubescent like the stems and leaves. Inflorescences axillary fascicles of (1)2–6 flowers, usually only one open at a time, sparsely to densely pubescent with mixed simple and dendritic trichomes like the stems; pedicels at anthesis 0.9–1 cm long, ca. 0.75 mm in diameter at the base, ca. 1.5 mm in diameter at the apex, nodding and the flowers borne below the leaves, sparsely to densely pubescent with transparent mixed simple and/or dendritic uniseriate like those of the stems and leaves, articulated at the base; pedicel scars tightly packed in the leaf axils. Buds elliptic, the corolla strongly exserted from the calyx tube before anthesis, the calyx appendages clasping the buds. Flowers 5-merous, apparently all perfect. Calyx tube 2–3 mm long, 2.5–3.5 mm in diameter, conical to openly cup-shaped, sparsely to densely pubescent like the stems and pedicels, with 10(12) linear awl-like appendages 1–5 mm long at anthesis, these variable in length even in a single flower, the appendages emerging at the rim or to 0.5 mm below, pubescent like the rest of the calyx. Corolla 1.4–1.8 cm in diameter, white or lavender with a green central area, often as two green dots at the base of each lobe, stellate, lobed nearly to the base, interpetalar tissue absent, the lobes 4–6 mm long, ca. 3 mm wide, spreading or slightly reflexed, membranous, adaxially glabrous, densely puberulent/papillate in the distal half abaxially, the tips and margins densely papillate. Stamens equal; filament tube minute; free portion of the filaments 0.5–1 mm long, glabrous; anthers 3–3.5 mm long, 1–1.5 mm wide, ellipsoid, the tips slightly pointed, yellow, poricidal at the tips, the pores tear-drop shaped, distally directed, lengthening to slits with age. Ovary conical, glabrous; style 4.5–6 mm long, straight, glabrous; stigma capitate, the surfaces minutely papillate. Fruit a globose berry, 1–1.5 cm in diameter, bright red when ripe, changing from green to orange to red through development, the pericarp glabrous, thin, shiny and transparent; fruiting pedicels 1–1.8 cm long, ca. 1 mm in diameter at the base, ca. 1.5 mm in diameter at the apex, green, not markedly woody, erect with the fruits borne above the leaves; fruiting calyx a flat plate beneath the fruit, the calyx appendages elongating to ca. 2 times their length in flower, spreading and forming a star under the berry (see Fig. 2C). Seeds 100+ per berry, 1.5–2 mm long, 1–1.5 mm wide, flattened and prismatically irregularly tear-drop shaped, straw-yellow, the surfaces deeply pitted, the testal cells sinuate in outline. Stone cells absent. Chromosome number: n=24 (Symon 1985; based on *Kairo & Symon 10652*).

#### Distribution

**Figure 12.**
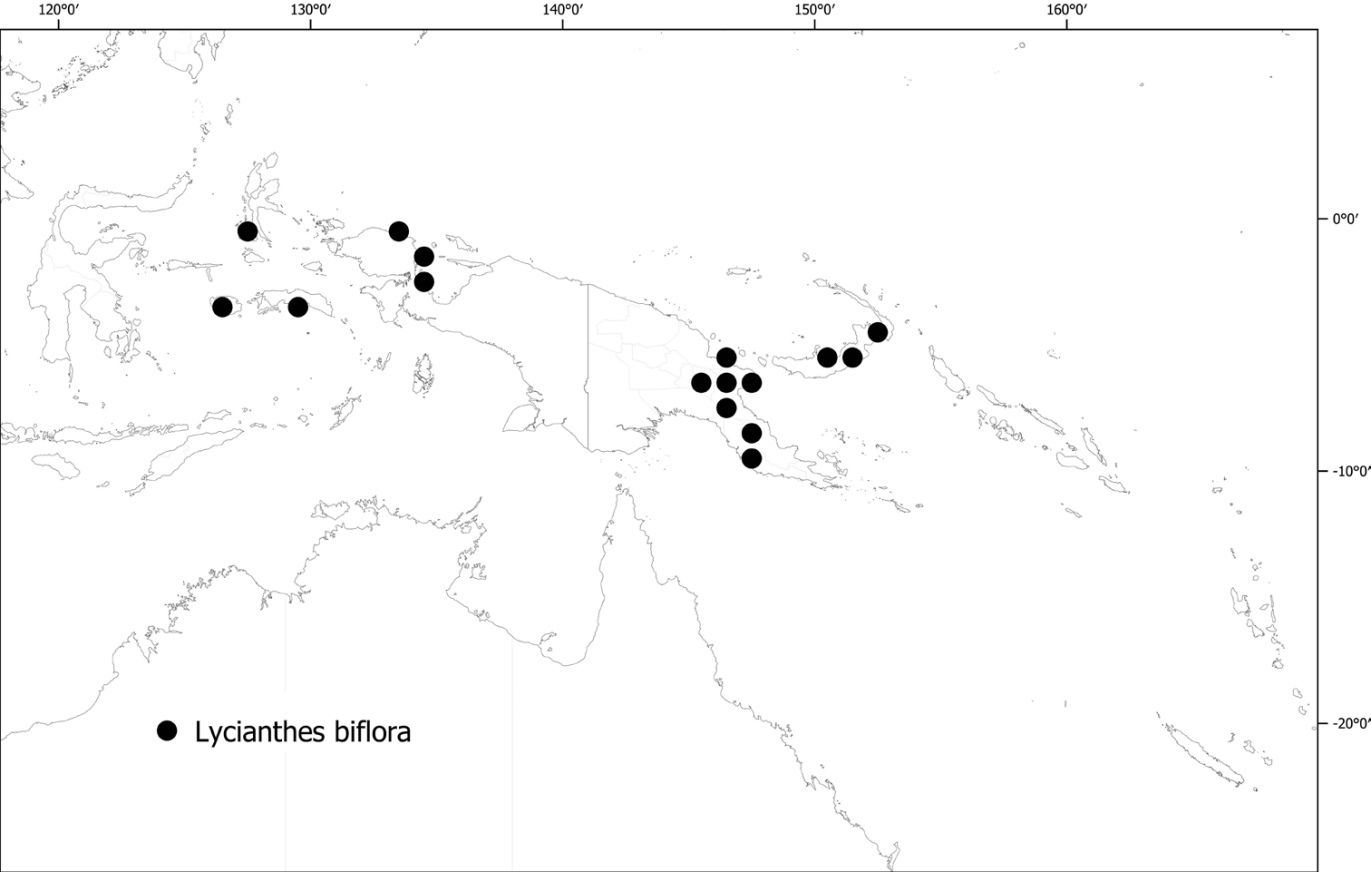
Distribution of *Lycianthesbiflora* (only for region treated in this monograph).

**(Fig. 12).***Lycianthesbiflora* is a widely distributed species in southeast Asia, ranging from India and China through much of Indonesia; the island of New Britain in the Bismarck Archipelago represents the easternmost edge of its range. On the island of New Guinea it is widely distributed in both Papua New Guinea and Indonesia.

#### Ecology and habitat.

*Lycianthesbiflora* is a plant of disturbed habitats. Often described as a weed, it grows in secondary forests, along stream beds and roads, in burn regrowth and in the vicinity of agricultural fields, from 100 to 1,400 m elevation.

#### Common names.

None recorded from this region (many vernacular names are known from elsewhere in the species range).

#### Preliminary conservation assessment

**(IUCN 2020).**EOO (region treated here only 3,560,380 km^2^ - LC); AOO (region treated here only 108 km^2^ - EN). *Lycianthesbiflora* is widely distributed within the region treated here and more broadly; this coupled with its weedy nature suggests a preliminary threat status of Least Concern (LC).

#### Discussion.

*Lycianthesbiflora* is a widely distributed species throughout tropical Asia, occurring north to Japan and east to New Britain. Bitter (1919) treated it as a species complex (“Gesamtart” = inclusive species) but nevertheless described many infraspecific variations from across its range based on small differences in pubescence, leaf shape and distribution. Material from New Guinea and the Solomon Islands matches the neotype specimen from China selected by Hul and Dy Phon (2014) and no infraspecific taxa or synonyms have been described based on material from Australia, New Guinea or the Pacific Islands. Therefore, full synonymy for *L.biflora* has been left to a future monograph treating *Lycianthes* in Asia (S. Knapp, in prep.). Specimens identified as *L.biflora* in the file Suppl. material 3 are preliminary and may be changed.

*Lycianthesbiflora* is a small, weak-stemmed shrub, usually growing in open places. Even in New Guinea it is very variable in degree and type of pubescence, with individuals ranging from densely pubescent with mixed branched and simple trichomes to individuals that are almost glabrous. Length of calyx appendages vary between individuals and also within a flower; calyx appendages can be the same or slightly different lengths within the same flower and vary in length between individual plants. The length of pedicels in fruit also varies across the species range, as does number of flowers in a fascicle; despite its name, plants of *L.biflora* do not always have two flowers per fascicle but can have up to six. In the field, the plants are distinctive, with the flowers hanging below the leaves and the pedicels becoming erect through fruit maturation with ripe fruits held above the leaves, where they are exposed to their dispersers (probably birds, although this has not been verified in the field).

On New Guinea, *Lycianthesbiflora* could be most easily confused with *L.bitteriana*, a similarly shrubby species of open areas. *Lycianthesbiflora* can be distinguished from *L.bitteriana* in its red versus blackish purple berries, its branched trichomes that are loose and shaped like deer anthers versus congested branched trichomes that look like Christmas trees. Plants of *L.biflora* are generally smaller and less robust than those of *L.bitteriana*.

Like many other species of Solanaceae that are widespread, small differences in morphology can look very different when looked at in isolation but become difficult to disentangle when morphology is examined across a wide geographical range (e.g., see Knapp 2013; Särkinen et al. 2018; Knapp et al. 2019). Future studies on the genetics and sequence variability of the considerable morphological variation of *L.biflora* across its range are needed.

#### Specimens examined.

Australia. **Christmas Island**: middle of Island, Lombok utan, 1898, *Andrews s.n.* (BM, K); Aug 1908, *Andrews 181* (BM); Christmas Island, (So of Java), 20 Nov 1888, *Lister s.n.* (K); Aug 1980, *Powell 164* (K); Phosphate Hill, Oct 1904, *Ridley 34* (K).

Indonesia. **Maluku**: Buru, NW Buru, S. of Bara, Waeduna River, 350–400 m, 16 Nov 1984, *van Balgooy 4916* (A); Seram, Manusela National Park, along a trail between Hatumete (sea level) and Hoale Pass (1,770 m) southern slope of Murkele Ridge, Kecamatan District, Tehoru; C. Seram, 550–1,200 m, 20 Feb 1985, *Kato et al. C-7273* (A). **Maluku Utara**: Bacan, Babang, Kec. Labuha, 100 m, 26 Aug 1986, *Ramlanto 869* (K, L); North Maluku, Gunung Sibela, N Moluccas, Bacan Island, Gunung Sibela near Waiaua, 1,000 m, 23 Oct 1974, *de Vogel 3565* (L, LAE, MO); Gunung Sibela, N Moluccas, Bacan Island, Gunung Sibela near Waiaua, 250 m, 28 Oct 1974, *de Vogel 3718* (L, MO). **Papua**: NE Kepal Burung, Kabupaten Manokwari, Kecamantan Manokwari, mountains S of the Arfak Plains, steep ridges between Arfak Plains and Gunung Itsiwei, 550 m, 29 Apr 1994, *Sands et al. 6431* (K). **Papua Barat (West Papua)**: Andjai [Andaj], Kebar Valley, 600 m, 7 Sep 1959, *Moll BW-9529* (L); North East Kepala Burung, Kabupatem Manokwari, Arfak Mountains, Mupi Dessa, trail from Mupi village to G[unung] Humibou, near Sungai Mupi between confluence of Kali Ngwes and Sungai Mupi and site of Kamnpong Mubri Lama, 875 m, 14 Apr 1995, *Sands & Maturbongs 6791* (A, K); Wondiwoi Mountains, Wandammen Peninsula, 300 m, 24 Feb 1962, *Schram BW-10645* (L); Wondiwoi Mountains, Wandammen Peninsula, 350 m, 28 Feb 1962, *Schram BW-10744* (L, WAG).

Papua New Guinea. Bismarck Archipel., 1889, *Warburg 21250* (BM). **Central**: Boridi, 914 m, 16 Nov 1935, *Carr 14991* (BM, K, L, NY); [Merska Hills] Sogeri Region., 762 m, 10 Apr 1886, *Forbes*, *H.O. 882* (BM, CAL, MEL, P); subdistrict Port Moresby, on ridge below Boridi village, 920 m, 1 Oct 1973, *Foreman & Vinas LAE-60242* (A, LAE); NE of Manumu Village, subdistrict Port Moresby, 450 m, 16 Sep 1973, *Isles & Vinas NGF-33899* (L). **East New Britain**: near Mapping site at edge of Mengen Massif, subdistrict Pomio, 885 m, 9 Jun 1973, *Stevens & Lelean LAE-58668* (E, K, LAE); Gazelle Peninsula, Baining Mountains, bulldozer track into the Wild Dog Prospect at Mt Sinvit, 950 m, 10 Feb 2004, *Takeuchi et al. 16902* (A, K, L). **Eastern Highlands**: Kassam Pass, Kainantu subdistrict, 1,280 m, 9 Jan 1988, *Henty et al. NGF-29203* (K, LAE); Crater M[ountain] Wildlife Management Area, Kusare, near the El Niño burn area, 1400 m, 28 Jul 1998, *Takeuchi 12688* (A, K, LAE). **Madang**: “Kaiser Wilhelmsland, waldranderam oberen Djamu” [northern New Guinea], 700 m, 9 Feb 1908, *Schlechter 17305* (P). **Milne Bay**: Biniguni Camp, Gwariu River, 200 m, 12 Aug 1953, *Brass 23978* (A, LAE). **Morobe**: 1955 Planting area, Bulolo. Morobe District, T.N.G., 1,067 m, 9 Jun 1955, *Floyd 7459* (BM, K, LAE, US), 15 Jun 1955, *Floyd 7509* (BM, K, LAE); Mount Missan, C.N.G.T. Logging areas, Stoney Creek, on foot slopes of Mount Missan (near Bulolo), Wau subdist., 914–1,219 m, 1 Jun 1977, *Kairo & Symon 10652* (K, LAE); Bulolo, Middle Logging Area, subdistrict Wau, 853 m, 10 Aug 1966, *Kairo & Streimann NGF-27869* (K, LAE); Oomsis Creek, Markham valley, 500 m, 3 Feb 1960, *Millar NGF-11795* (K, LAE); Oomsis Creek, Markham Valley, 152 m, 3 Feb 1960, *Millar NGF-11794* (A); Finschaffen, 300 m, 5 Jul 1978, *Rau 380* (A, L); Hump L.A. 5 mi SE Bulolo, Wau subdistrict, 1,067 m, 15 Mar 1971, *Streimann & Kairo NGF-25853* (A, K, LAE); Bulolo, 914 m, Jan 1957, *Wells NGF-7569* (A, K, LAE). **New Britain Island**: New Britain, New Pommeron. Bei Mussawa., Nov 1901, *Schlechter 13748* (BM, K, P); bei Mussawa, Nov 1901, *Schlechter 13749 a*, (K). **Oro**: Isuarava [Isurava], 5 Mar 1936, *Carr 15965* (BM, L).

### ﻿4. Lycianthes bitteriana

Taxon classificationPlantaeSolanalesSolanaceae

(Symon) A.R.Bean, Austrobaileya 6(3): 567. 2003.

701B8A3D-8C70-5DCE-99F1-4E6844DA8B99

Figs 13 , 14

Solanum bitteriana Symon, J. Adelaide Bot. Gard. 8: 34, fig. 5. 1985. Type. Papua New Guinea. Morobe: Stoney Creek, CNGT logging area, on foot slopes of Mount Missan, near Bulolo (subdist. Wau), 1,067 m, 1 May 1977, *D.E Symon & A. Kairo 10651* (holotype: AD [AD98581513]; isotypes: AD [AD98581514], CANB [CANB355342], F, K [K001080539], L [L.4153288], LAE [acc. # 254856], MO [MO-503790, acc. # 3748792], US [00050681, acc. # 3083630]).

#### Type.

Based on *Solanumbitterianum* Symon.

**Figure 13.**
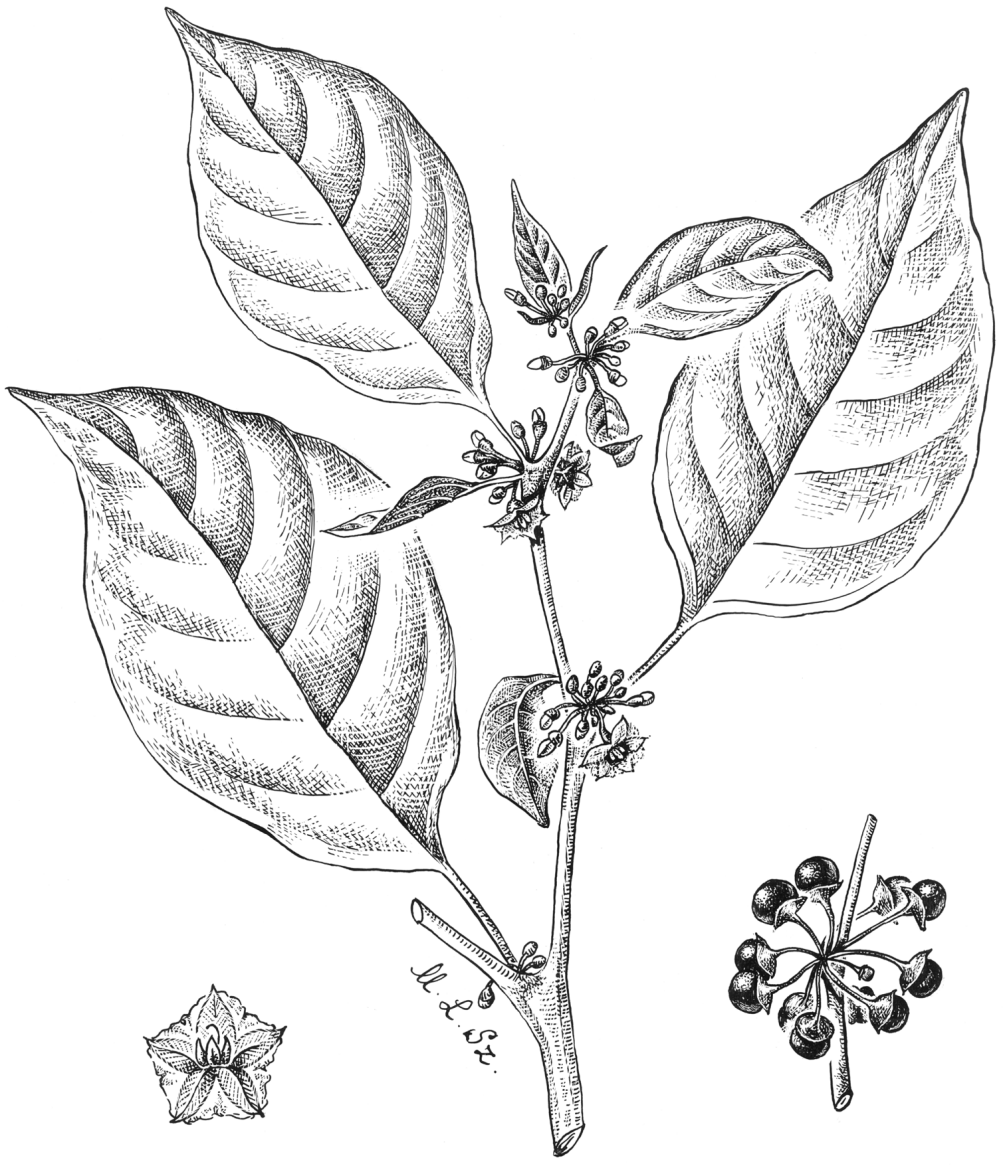
*Lycianthesbitteriana* (Symon) A.R.Bean. Drawing by M.L. Szent-Ivany, first published in Symon (1985: fig. 5, as *S.bitterianum* Symon). Courtesy of the Board of the Botanic Gardens and State Herbarium (Adelaide, South Australia), reproduced with permission.

#### Description.

Large woody herbs to shrubs ca. 2 m tall; stems terete, densely pubescent with uniseriate dendritic 5–10-celled trichomes to 0.5 mm long, the branches short and congested (“tannenbaumartig”), drying yellowish tan; new growth densely pubescent with uniseriate dendritic trichomes like those of the stems, drying yellowish tan, not markedly glabrescent; bark of older stems dark brown, somewhat glabrescent. Sympodial units difoliate, the leaves geminate, the leaves of a pair differing only in size, not in shape. Leaves simple; blades of major leaves 9–14 cm long, 4–6 cm wide, elliptic to broadly elliptic, widest in the middle, discolorous, membranous to chartaceous; adaxial surfaces moderately and evenly pubescent with dendritic trichomes with congested branches like those of the stems, these denser along the veins; abaxial surfaces more densely pubescent with dendritic trichomes, but the lamina still visible; principal veins 8–9 pairs, yellowish tan abaxially; base acute to truncate, oblique; margins entire; apex acuminate; petioles 1.6–3 cm long, densely dendritic-pubescent; blades of minor leaves 2.5–5.5 cm long, 1.3–3 cm wide, similar in shape and pubescence to the major leaves; petioles 0.5–1 cm long. Inflorescences axillary, the flowers borne on a woody axis with 3–4 short branches to 0.8 cm long, with 10–15 flowers, densely dendritic-pubescent with trichomes like those of the stems and leaves; pedicels 0.9–1 cm long at anthesis, ca. 0.5 mm in diameter at the bae, ca. 1 mm in diameter at the apex, densely pubescent with dendritic trichomes with congested branches, articulated at the base; pedicel scars tightly packed along the woody axes. Buds narrowly ellipsoid, the corolla ca. halfway exserted from the calyx tube before anthesis. Flowers 5-merous, apparently perfect. Calyx tube 2–2.5 mm long, 2–2.5 mm wide, cup-shaped, densely dendritic-pubescent, with 4–5 linear, awl-shaped appendages 0.5–1 mm long, these varying in length within individual flowers, the rim extending ca. 0.1 mm beyond the appendages. Corolla 1–1.2 cm in diameter, white or “whitish blue”, stellate, lobed ca. halfway to the base, interpetalar tissue present, the lobes 3–3.5 mm long, ca. 2.5 mm wide, spreading or slightly reflexed, membranous, glabrous with densely papillate tips and a few dendritic trichomes along the midvein adaxially. Stamens equal; filament tube minute; free portion of the filaments ca. 0.5 mm long, glabrous; anthers ca. 2.5 mm long, ca. 1 mm wide, yellow, poricidal at the tips, the pores lengthening to slits with age. Ovary conical, glabrous; style ca. 6.5 mm long, glabrous; stigma minutely capitate and slightly bilobed. Fruit a globose berry, 0.6–0.7 cm in diameter, black or purple-black when ripe, the pericarp glabrous, thin, matte, opaque; fruiting pedicels 1.1–1.3 cm long, ca. 1 mm in diameter at the base, ca. 2 mm in diameter at the apex, green (?), erect to spreading, densely dendritic-pubescent; fruiting calyx a spreading cup beneath the berry, dendritic-pubescent. Seeds ca. 100 + per berry, ca. 1.5 mm long, ca. 1.5 mm wide, flattened with a deep notch at the hilum, yellowish tan, the surfaces minutely pitted, the testal cells sinuate in outline. Stone cells absent. Chromosome number not known.

**Figure 14.**
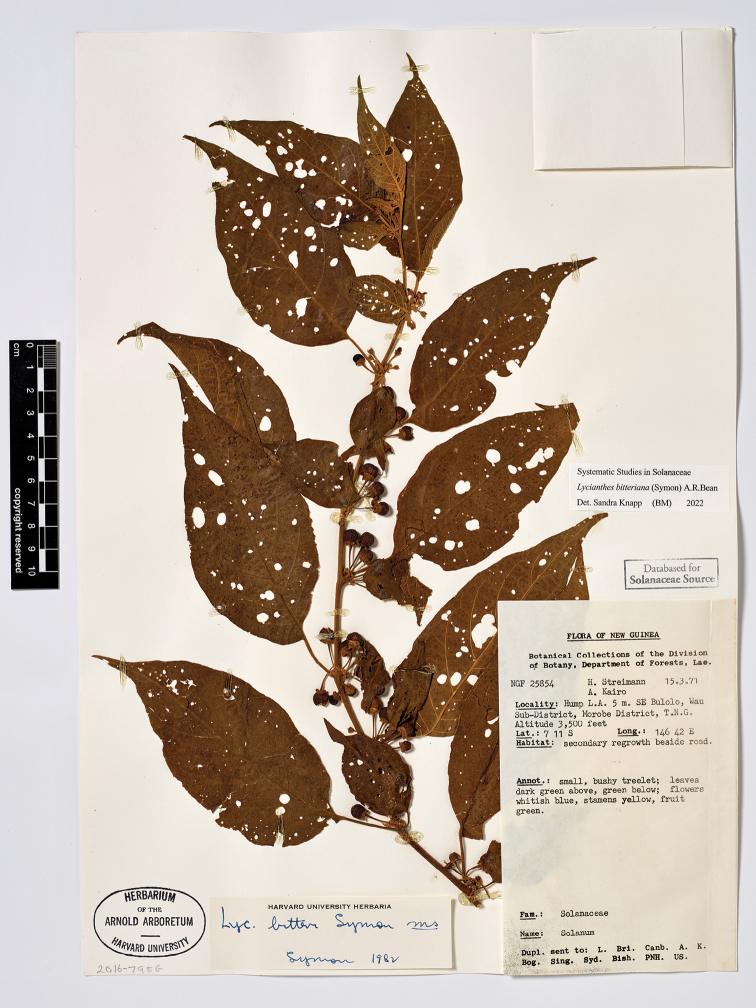
*Lycianthesbitteriana* herbarium specimen. Papua New Guinea. Morobe: *Streimann & Kairo NGF-25854* (A). Courtesy of the Herbarium of the Arnold Arboretum of Harvard University, reproduced with permission.

#### Distribution

**Figure 15.**
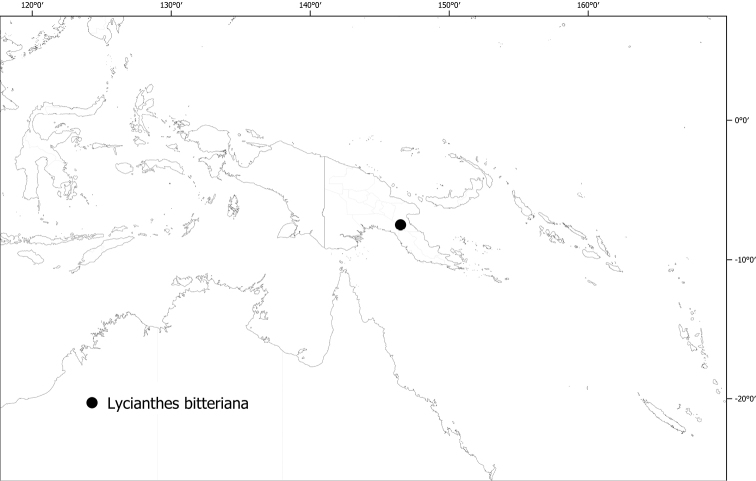
Distribution of *Lycianthesbitteriana*.

**(Fig. 15).***Lycianthesbitteriana* is endemic to the island of New Guinea; it has only been collected in Papua New Guinea (Morobe).

#### Ecology and habitat.

*Lycianthesbitteriana* is a plant of secondary forests at mid-elevations, from ca. 1,000 m.

#### Common names.

None recorded.

#### Preliminary conservation assessment

**(IUCN 2020).**EOO (22 km^2^ - CR); AOO (12 km^2^ - EN). *Lycianthesbitteriana* is known from three localities less than 5 km apart, but could be more widely distributed, given its apparently secondary forest nature. I propose a preliminary threat status of Critically Endangered (CR [B1, 2a(iv)]) for *L.bitteriana*, but field assessment of its distribution is a priority.

#### Discussion.

*Lycianthesbitteriana* is a distinctive species with its congested branched trichomes that look like tiny Christmas trees (“tannbaumartig” of Seithe 1962) and shiny black berries. Unlike many of the other endemic New Guinea Lycianthes, *L.bitteriana* is a coarse herb or small shrub, in habit very similar to the widely distributed *L.biflora*. These two species can be easily distinguished by mature berry colour (black in *L.bitteriana* versus bright red in *L.biflora*), inflorescence morphology (many flowers on a short axis in *L.bitteriana* versus few-flowered fascicles in *L.biflora*), and branched trichomes morphology (*L.bitteriana* with congested branches versus the loosely branched trichomes of *L.biflora*). In addition, the corolla of *L.bitteriana* has abundant interpetalar tissue and is divided ca. halfway to the base, while that of *L.biflora* is deeply stellate with little or no interpetalar tissue.

*Lycianthesdendropilosa* has similar branched trichome with congested branches but differs from *L.bitteriana* in number of flowers per inflorescence (many in *L.bitteriana*, 1–3 in *L.dendropilosa*), calyx appendages (ca. 10 in *L.bitteriana*, absent in *L.dendropilosa*) and adaxial leaf morphology (evenly pubescent in *L.bitteriana*, glabrous and shiny in *L.dendropilosa*).

Given the nature of the secondary habitat in which *Lycianthesbitteriana* occurs, it is likely to be more widely distributed across the island of New Guinea.

#### Specimens examined.

Papua New Guinea. **Morobe**: Hump L.A. 5 mi SE Bulolo, Wau subdistrict, 1,067 m, 15 Mar 1971, *Streimann & Kairo NGF-25854* (A, K, LAE); Mun. Bulolo District, Bulolo, 14 Jan 1957, *Wells NGF-7565* (K, L, LAE).

### ﻿5. Lycianthes cladotrichota

Taxon classificationPlantaeSolanalesSolanaceae

(Bitter) Bitter, Abh. Naturwiss. Vereins Bremen 24 [preprint]: 504. 1919.

6058C1EA-7142-513C-8114-67AE94E15168

**Figure 16.**
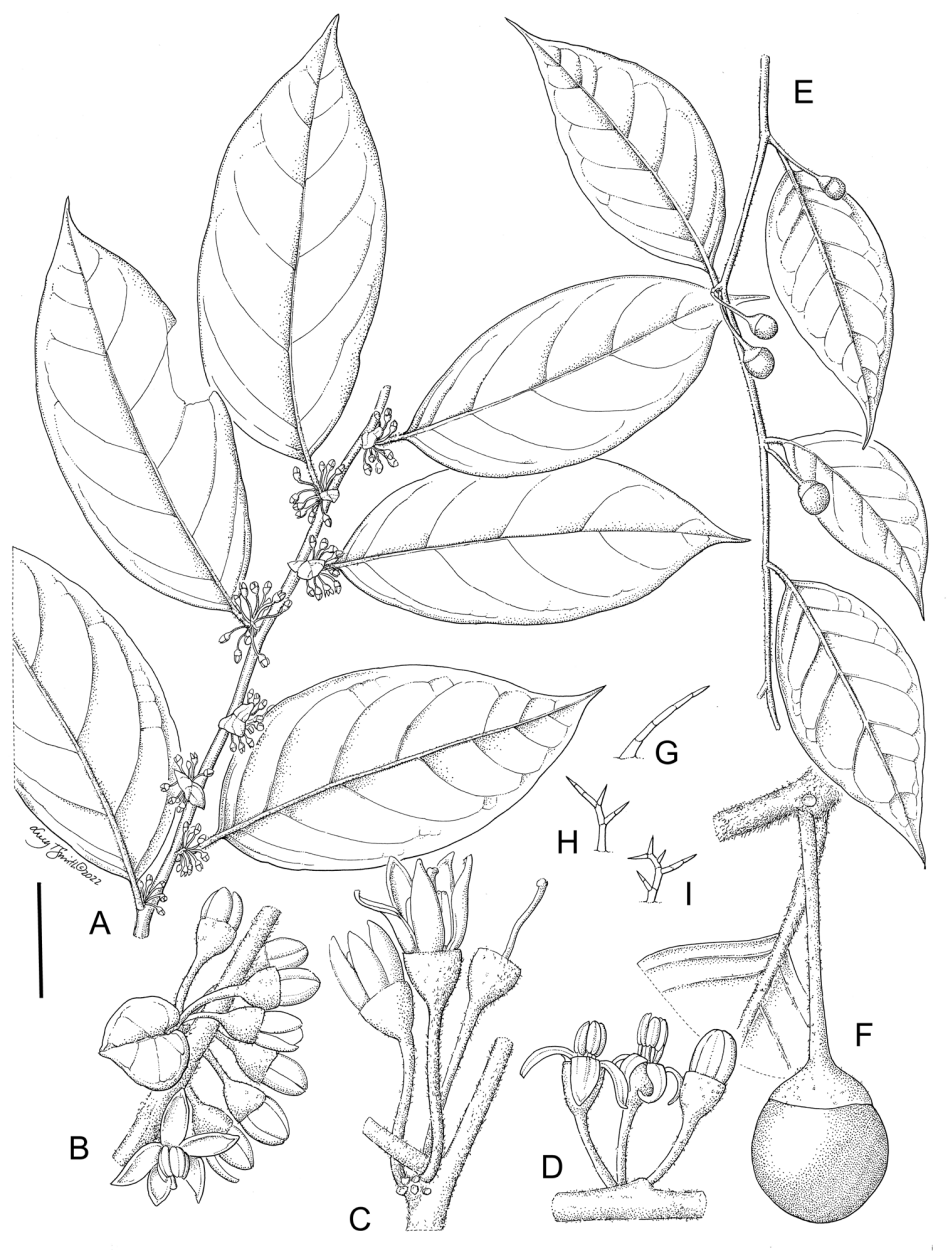
*Lycianthescladotrichota* (Bitter) Bitter **A** flowering branch **B** inflorescence with long-styled flowers **C** long-styled flower **D** short-styled flowers **E** fruiting branch **F** berry **G, H** trichomes from stem **I** trichome from calyx. (**A, G, H***Streimann & Katik NGF-30479***B, I***Takeuchi 22832***C***Crutwell 2310***D** photograph of *James et al. SAJ-1385***E, F***Takeuchi 12379*). Drawing by Lucy Smith. Scale bars: 4 cm (**A, E**); 7 mm (**B, C**); 7.5 mm (**D, F**); 0.9 mm (**G–I**).

**Figure 17.**
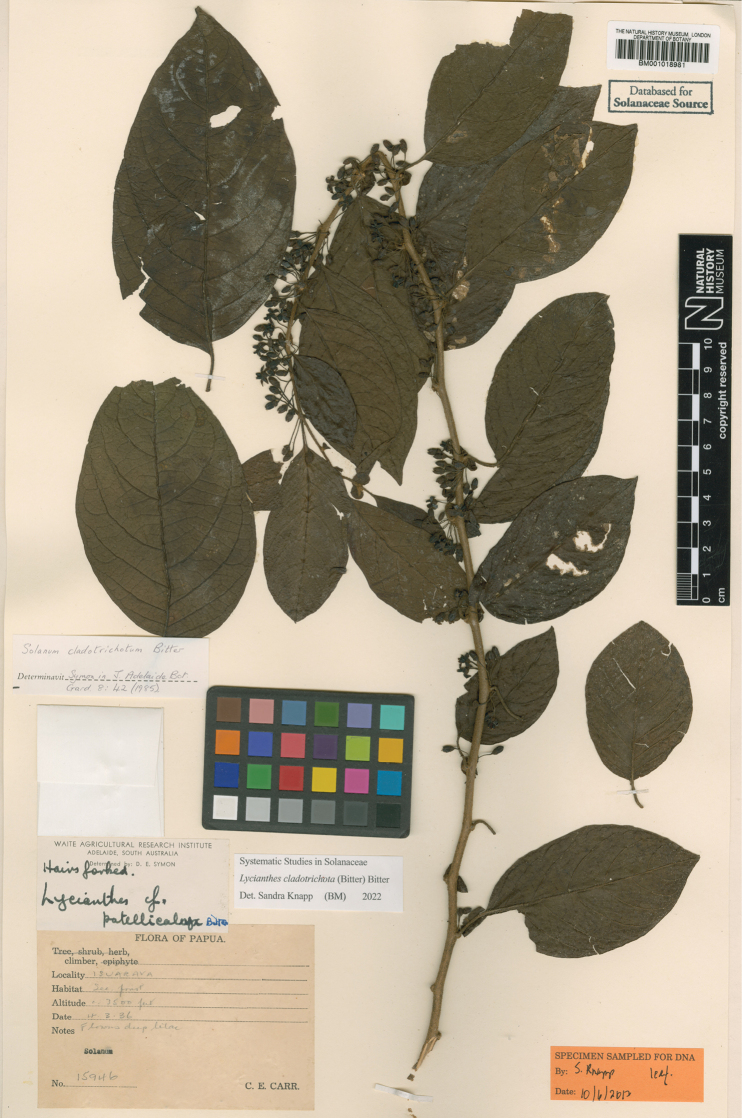
*Lycianthescladotrichota* herbarium specimen. Papua New Guinea. Oro: *Carr 15946* (BM001018981). Courtesy of the Trustees of the Natural History Museum, London, reproduced with permission.

Figs 3B, F , 16 , 17

Solanum cladotrichotum Bitter, Bot. Jahrb. Syst. 55: 96. 1917. Type. Papua New Guinea. Sanduan: “Sepikgebiet” [Felsspitze = Rocky Peak in Ledermann 1919], 1,400 m, Jul-Aug 1913, *C.L. Ledermann 12606* (holotype: B [destroyed]; lectotype, designated by Symon 1985, pg. 40: K [K000759443]; isolectotypes: BM [BM000778204], L [L0003588], LAE).
Solanum patellicalyx Bitter, Bot. Jahrb. Syst. 55: 99. 1917. Type. Papua New Guinea. East Sepik: “Hunsteinspitz” [Mount Hunstein], 1,300 m, Feb-Mar 1913, *C.L. Ledermann 11272*, *11483* (syntypes: B, destroyed; no duplicates found).
Lycianthes patellicalyx (Bitter) Bitter, Abh. Naturwiss. Vereins Bremen 24 [preprint]: 504. 1919. Type. Based on Solanumpatellicalyx Bitter.

#### Type.

Based on *Solanumcladotrichotum* Bitter.

#### Description.

Shrubs or more commonly described as woody climbers, to 10 m tall (long); stems terete, moderately to densely pubescent with stiff uniseriate dendritic trichomes to 1.5 mm long, the branches antler-like and well-spaced, occasionally also mixed with with stiff uniseriate simple trichomes ; new growth densely pubescent with stiff dendritic trichomes like those of the stems; bark of older stems pale brown, not markedly glabrescent, but becoming somewhat corky and peeling. Sympodia units unifoliate (minor leaves deciduous?) or difoliate and the leaves geminate, the leaves of a pair very different in size and shape; minor leaves often only present on new non-reproductive shoots. Leaves simple; blades of major leaves 7–19 cm long, 3.5–10 cm wide, elliptic to broadly elliptic (the type with narrowly elliptic leaves), widest in the middle, somewhat discolorous, chartaceous to coriaceous, thick and possibly rubbery-fleshy in live plants; adaxial surfaces shiny, glabrous but densely to moderately dendritic-pubescent along the veins and keeled midrib; abaxial surfaces sparsely and evenly pubescent with stiff antler-like dendritic trichomes ca. 0.5 mm long on the lamina, these denser along the veins and midrib; principal veins 8–12 pairs, prominent and impressed adaxially; base acute to truncate; margins entire; apex acute to abruptly acuminate with an elongate drip-tip; petiole 1–1.5 cm long, moderately pubescent with stiff dendritic trichomes like those of the stems and leaves; blades of minor leaves (if present) 1–2.2 cm long, 1–1.2 cm wide, orbicular and somewhat clasping the stem, bullate, pubescence like that of the major leaves; base truncate or cordate; margins entire; apex obtuse or rounded; petioles minute, to 0.5 cm long. Inflorescences dense axillary fascicles of 10–20 flowers, many flowers open at once, densely dendritic-pubescent with antler-like trichomes from the adjacent stems; pedicels at anthesis 0.7–1.5 cm long, ca. 0.5 mm in diameter at the base, ca. 1.5 mm in diameter at the apex, spreading to erect, possible held below the leaves, sparsely to moderately pubescent with stiff antler-like dendritic trichomes like those of the stems and leaves, articulated at the base; pedicel scars tightly clustered in the leaf axils. Buds ellipsoid, the corolla halfway exserted from the calyx tube before anthesis. Flowers 5-merous, heterostylous and apparently unisexual on separate plants, individual specimens either all long-styled and with fruit (e.g., *Takeuchi 22892*) or short-styled (e.g., *Carr 15946*). Calyx tube ca. 2 mm long, 3–4 mm in diameter, woody and stiff in dry specimens, fleshy in live plants, cup-shaped or slightly urceolate, sparsely pubescent with stiff dendritic trichomes like those of the stems and pedicels, mixed with some simple uniseriate trichomes of similar stiffness and size, without appendages, the rim slightly constricted. Corolla 1–1.3 cm in diameter, white or pale purple, stellate, lobed nearly to the base, interpetalar tissue absent, the lobes ca. 5 mm long, ca. 2 mm wide, spreading or perhaps reflexed, thick (fleshy in live plants), densely papillate on tips and margins, the tips slightly cucullate. Stamens equal; filament tube minute; free portion of the filaments 0.5–1 mm long, densely pubescent with tangled weak-walled simple uniseriate trichomes; anthers ca. 3 mm long, 1.25–1.5 mm wide, yellow, poricidal at the tips, the pores distally directed, not splitting with age. Ovary conical, glabrous; style in short-styled flowers less than 0.5 mm long or absent, in long-styled flowers 4–4.5 mm long, straight, glabrous; stigma broadly bilobed, the surfaces minutely papillate. Fruit a globose berry, 0.5–0.6 cm in diameter, yellow-green (immature?) or fleshy blue-violet (fide Bitter 1917 in description of *S.patellicalyx*), the pericarp glabrous, thin, matte, opaque; fruiting pedicels 1–1.4 cm long, ca. 1 mm in diameter at the base, 1.5–2 mm in diameter at the apex, spreading or pendent, somewhat woody, with a few dendritic trichomes; fruiting calyx a spreading cup beneath the berry, woody in dry specimens, fleshy in live plants (?), often with a few dendritic trichomes, but these usually absent and the calyx somewhat corky or warty. Seeds 80–100 per berry, 2–2.5 mm long, 1.2–1.5 mm wide, tear-drop shaped with no distinct notch, the hilum apical, tan, the surfaces deeply pitted (less so in centre of seeds), the testal cells pentagonal in outline. Stone cells absent. Chromosome number not known.

#### Distribution

**Figure 18.**
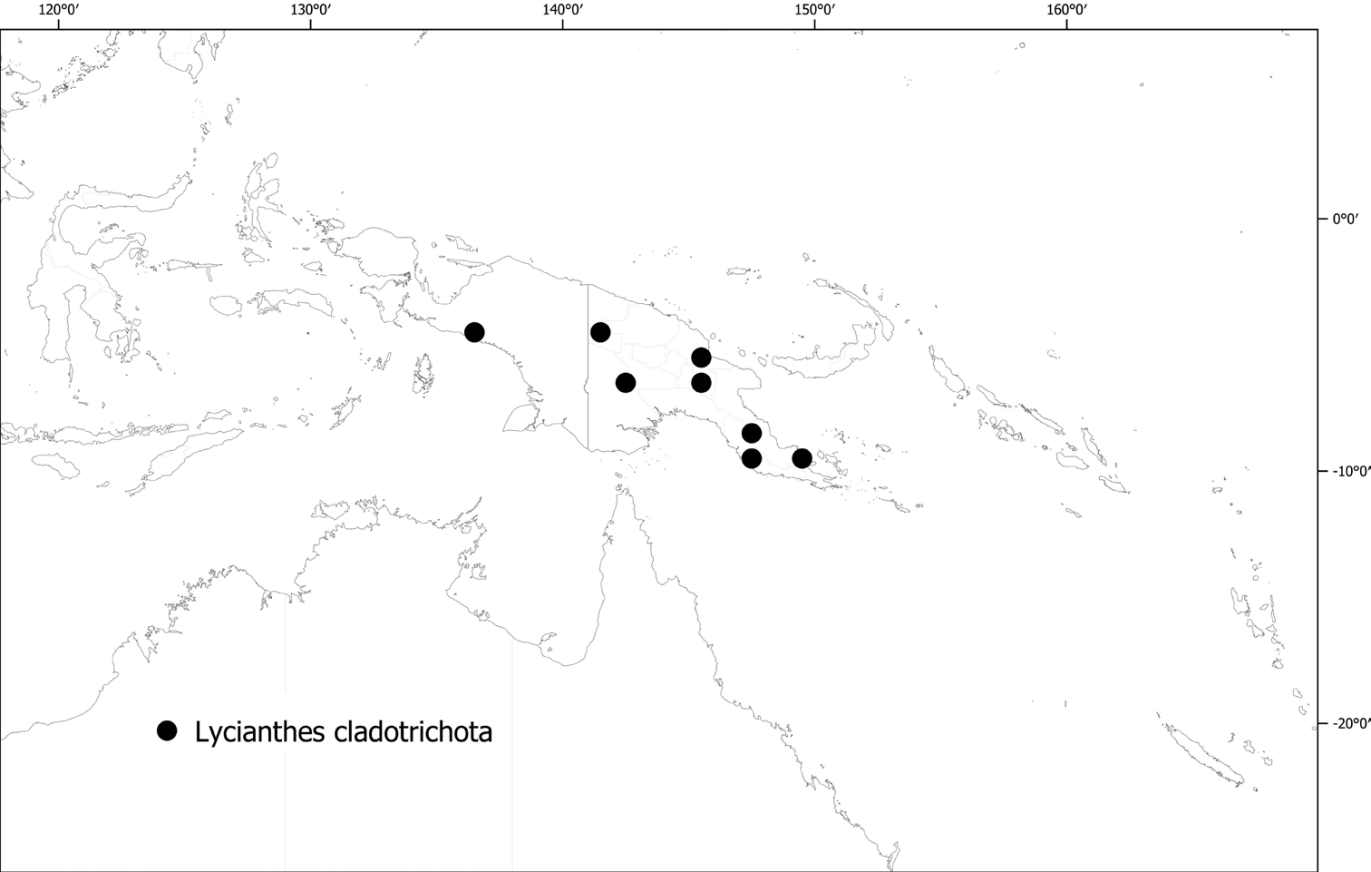
Distribution of *Lycianthescladotrichota*.

**(Fig. 18).***Lycianthescladotrichota* is endemic to the island of New Guinea; it has been collected in Papua New Guinea (Central, Eastern Highlands, Milne Bay, Oro, Sanduan, Western) and Indonesia (Papua).

#### Ecology and habitat.

*Lycianthescladotrichota* is a plant of lowland rainforest, occurring between 50 and 1,400 m elevation.

#### Common names.

Papua New Guinea. Oro: ahara (Orokaira language, *Hoogland 3979*).

#### Preliminary conservation assessment

**(IUCN 2020).**EOO (254,412 km^2^ - LC); AOO (48 km^2^ - EN). *Lycianthescladotrichota* is known from six localities across the island of New Guinea, but in spite of its apparently large distribution it has a relatively small area of occupancy (due to lack of collecting generally). Given the small AOO and its primary forest habitat, I suggest a preliminary threat status of Vulnerable (VU [B2a(iii, iv)]) for *L.cladotrichota*. It does occur in at least one protected area (Crater Mountain Wildlife Area).

#### Discussion.

*Lycianthescladotrichota* a relatively widely distributed species of wet, lowland forests. It is distinctive in its stiff, antrorse pubescence of dendritic trichomes, thick, shiny major leaves, and tiny, orbicular minor leaves. The sympodia often appear unifoliate, but this is due to the abscission of the minor leaves – on young stems they are uniformly present (Fig. 2B). The thick, slightly urceolate calyx tube (Fig. 2F) is somewhat like that of *L.oliveriana*, but that species is glabrous, and the minor leaves are not tiny and deciduous. The fruiting calyx in *L.cladotrichota* is a spreading cup, while that of *L.oliveriana* cups the lower part of the berry, making it look at bit like an acorn. *Lycianthesimpar* has similar small, heart-shaped or orbicular minor leaves, but can easily be distinguished by its simple rather than dendritic pubescence and in its inflorescence with a distinct axis; *L.cladotrichota* flowers are strictly fasciculate in the leaf axils.

The holotype of *Lycianthescladotrichota* (*Ledermann 12606*) was in Berlin and is no longer extant; fortunately, unlike many other Ledermann collections, several duplicates exist. *Lycianthespatellicalyx* was described (Bitter 1917) from collections made a few months earlier than those used to describe *L.cladotrichota*, the principal distinguishing characteristics of the former were its slightly broader and less pubescent leaves. *Lycianthescladotrichota* was described from flowering plants, and *L.patellicalyx* from fruiting material; in these plants that are possibly dioecious these differences can seem striking in the absence of more specimens. I have found duplicates of neither of the collections cited in the protologue of *L.patellicalyx* (*Ledermann 11272*, *11483*), both from Mount Hunstein (Veldkamp et al. 1988). Symon (1985) placed *L.patellicalyx* (as *S.patellicalyx*) in synonymy with *L.cladotrichota* based on the similarity in descriptions, I maintain that here, and await further collections to neotypify the former name.

#### Specimens examined.

Indonesia. **Papua**: Mimika Regency, Mount Jaya, PT-Freeport Indonesia Concession Area, Kuala Kencana, near Ecological Plot 5, 65 m, 25 Jan 1998, *Johns et al. 8900* (A, K, MO).

Papua New Guinea. **Central**: Kairuku-Hiri District, Mt. Gerebu, Trail towards summit ridge, 489 m, 5 Nov 2013, *James et al. SAJ 1385* (BISH, BM, LAE). **Eastern Highlands**: M[oun]t Otto, south slopes,, m, 6 Aug 1959, *Brass 30845* (K, L, LAE, US); Mount Gahavisuka, 2,250 m, Apr 1983, *Cruttwell 2310* (K, L, LAE); Daulo Pass, top of Daulo Pass, 2,320 m, 22 Jun 1977, *Symon*, & *Katik 10676* (K, LAE, MO, US); Crater Mountain Wildlife Area, ridge above Hauneäbäbo, 1,920 m, 21 Jul 1998, *Takeuchi 12379* (K, US). **Milne Bay**: Raba Raba subdistrict, junction Ugat and Mayu Rivers, near Mayu Island (Mt Suckling complex), 400 m, 25 Jul 1972, *Streimann & Katik NGF-34091* (E, K, L, LAE, US); **Oro**: Isuarava [Isurava], 1,067 m, 4 Mar 1936, *Carr 15946* (BM, G, K, L, NY, P), 15 Mar 1936, *Carr 16109* (BM, G, K, L, NY); “Northern Div.” near Pitoki village, ca. 3 km S of Kokoda station, 23 Sep 1953, *Hoogland 3979* (A, LAE); Kokoda, ‘Northern District, Papua’, 400 m, 28 Jul 1964, *Millar NGF-23549* (K, L, LAE). **Western**: Baia River (Expedition Bivouac 3) survey track B, 300 m, 13 Feb 2008, *Takeuchi et al. 22892* (A, K, LAE, MO).

### ﻿6. Lycianthes dendropilosa

Taxon classificationPlantaeSolanalesSolanaceae

(Symon) A.R.Bean, Austrobaileya 6(3): 567. 2003.

A7BB03D5-D7DF-5352-A0A4-C16D7B3A5648

Figs 19 , 20

Solanum dendropilosum Symon, J. Adelaide Bot. Gard. 8: 44. 1985. Type. Papua New Guinea. Western Highlands: Laiagam, Lagaip valley, near Kepilam village, 2,439 m, 2 Aug 1960, *R.D. Hoogland & R. Schodde 7291* (holotype: CANB [CANB83729]; isotypes: A [00395063], BM [BM000886135], BRI [BRI-AQ0080425], G [G00343297], L [L0003631], LAE [acc. # 39126], US [00479494, acc. # 2411884]).

#### Type.

Based on *Solanumdendropilosum* Symon.

**Figure 19.**
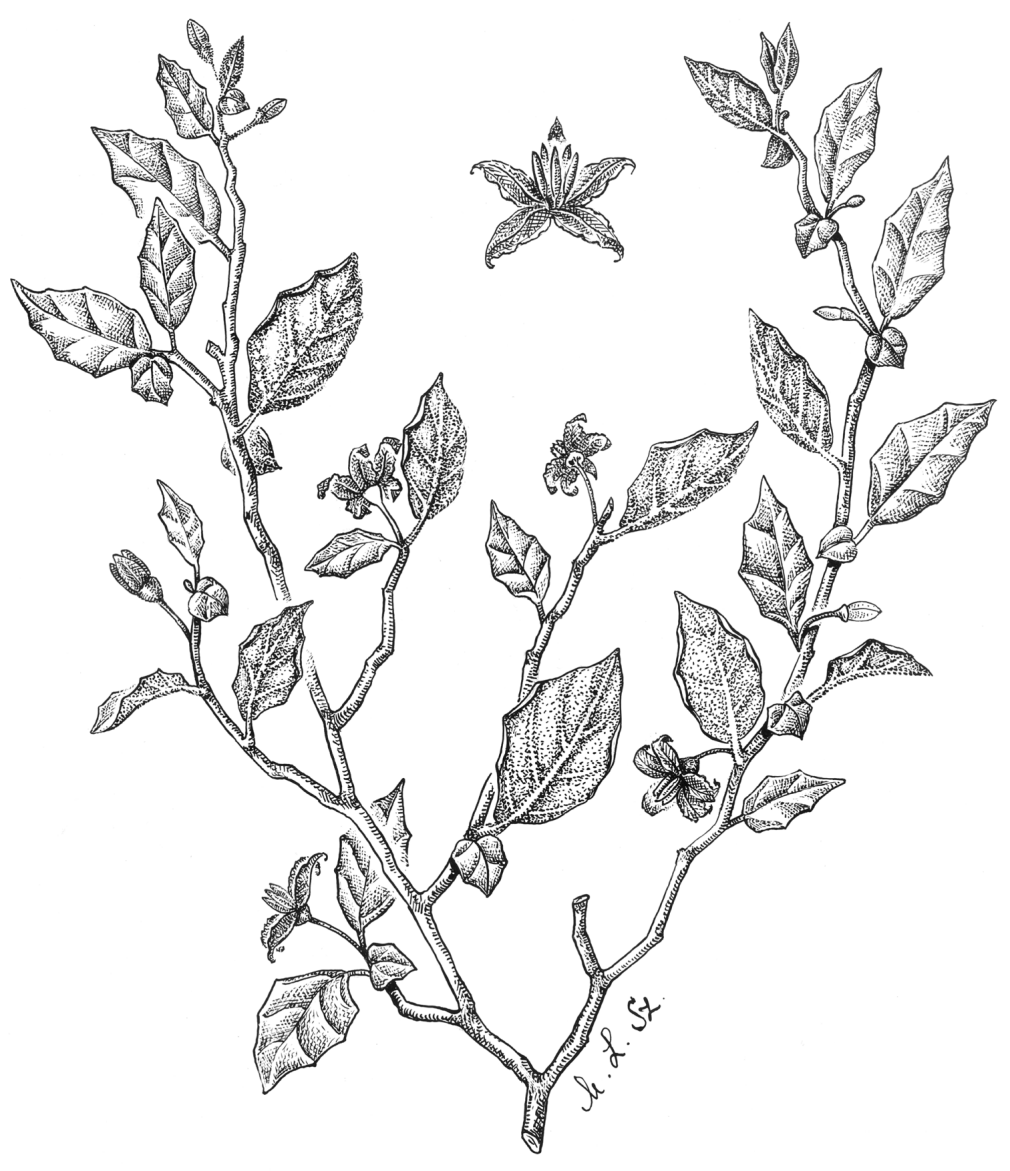
*Lycianthesdendropilosa* (Symon) A.R.Bean. Drawing by M.L. Szent-Ivany, first published in Symon (1985: fig. 11, as *S.dendropilosum* Symon). Courtesy of the Board of the Botanic Gardens and State Herbarium (Adelaide, South Australia), reproduced with permission.

#### Description.

Straggling shrubs or root climbers, 1–1.5 m tall; stems terete, densely glandular-papillate and densely pubescent with uniseriate dendritic trichomes to 0.75 mm long, the branches short and congested (“tannenbaumartig” sensu Seithe 1962), these drying yellowish tan; new growth densely papillate and pubescent like the stems, the trichomes tangled and interwoven; bark of older stems pale brown, becoming somewhat corky and glabrescent. Sympodial units difoliate, the leaves geminate, the leaves of a pair differing in size and shape. Leaves simple; blades of major leaves 2.5–4 (8) cm long, 1–2.5 cm wide, elliptic to narrowly elliptic (lanceolate in *Symon & Katik 10691*), widest in the middle, discolorous, coriaceous or membranous (*Symon & Katik 10691*); adaxial surfaces shiny, sparsely and unevenly pubescent with dendritic trichomes with congested branches like those of the stems, these denser along the midrib and principal veins; abaxial surfaces densely dendritic pubescent (lamina barely visible in *Hoogland & Schoode 7291*), yellowish tan; principal veins 4–6 pairs, densely pubescent, the midrib keeled adaxially; base acute; margins entire, strongly revolute in *Hoogland & Schoode 7291*; apex acute or more often acuminate; petiole 0.4–0.6 cm long, densely dendritic-pubescent like the stems; blades of minor leaves 0.6–0.7 cm long, 0.6–0.7 cm wide, orbicular or heart-shaped, similar in texture and pubescence to the major leaves; base cordate or rounded; margins entire or revolute; apex rounded; petiole absent or less than 0.1 cm long, densely dendritic-pubescent. Inflorescences axillary fascicles of 1–3 flowers, only one open at a time, densely dendritic-pubescent; pedicels at anthesis 1–1.2 cm long, ca. 1 mm in diameter at the base, ca. 1.5 mm in diameter at the apex, erect or spreading, densely pubescent with golden dendritic trichomes with congested branches like those of the stems and leaves, articulated at the base; pedicel scars tightly packed in the leaf axils, Buds long-ellipsoid, the corolla strongly exserted from the calyx tube before anthesis. Flowers 5-merous, probably heterostylous and unisexual, no styles seen in the only flowering specimen seen (*Hoogland & Schoode 7291*), the plants possibly dioecious. Calyx tube ca. 3 mm long, 3–3.5 mm in diameter, cup-shaped, densely golden dendritic-pubescent with trichomes like those of the pedicels, thick and coriaceous (fleshy in live plants?), without appendages, the rim slightly thickened. Corolla 1.5–2 cm in diameter, purple (mauve), deeply stellate, lobed nearly to the base, interpetalar tissue absent, the lobes 6–9 mm long, 2–3 mm wide, spreading, thick and fleshy, adaxially glabrous, abaxially moderately pubescent with simple uniseriate trichomes 0.25–0.5 mm long, if these branched then merely forked, the branches not congested, the tips and margins densely papillate, the tips cucullate. Stamens equal; filament tube minute; free portion of the filaments 1–1.5 mm long, glabrous; anthers 5–7 mm long, 1–1.2 mm wide, long-ellipsoid and tapered at the tips, yellow, poricidal at the tips, the pores distally directed, not elongating with age. Ovary not seen. Fruit and seeds not known. Chromosome number not known.

**Figure 20.**
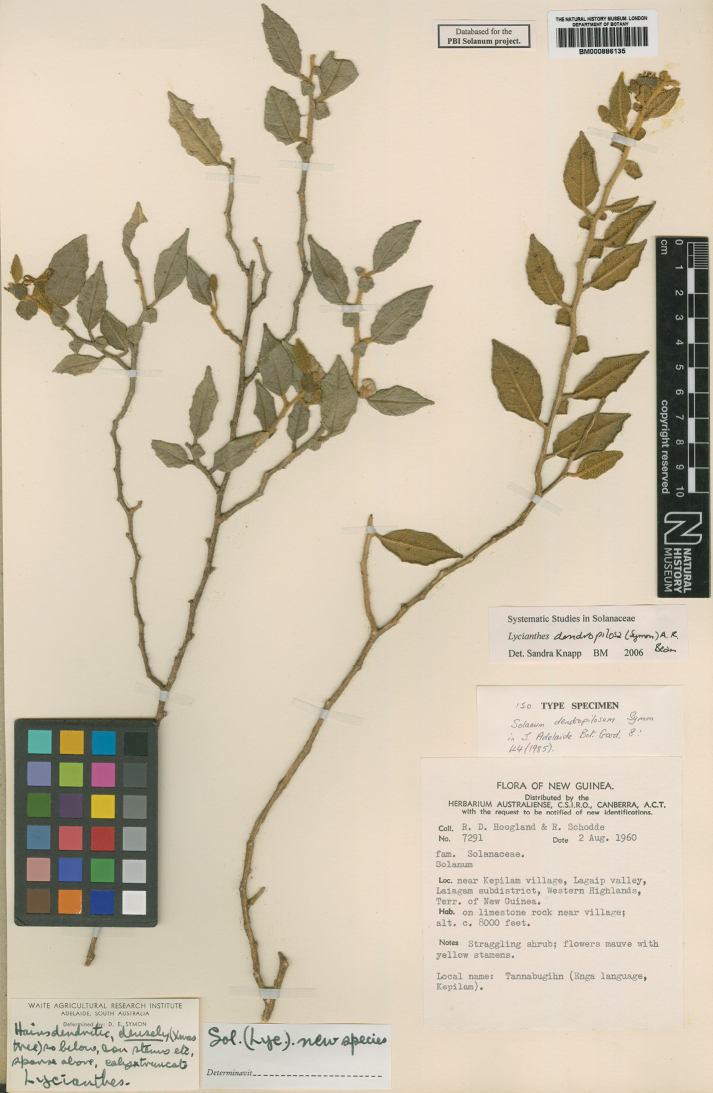
*Lycianthesdendropilosa* herbarium specimen. Papua New Guinea. Western Highlands: *Hoogland & Schodde 7291* (isotype of *S.dendropilosum*, BM000886135). Courtesy of the Trustees of the Natural History Museum, London, reproduced with permission.

#### Distribution

**Figure 21.**
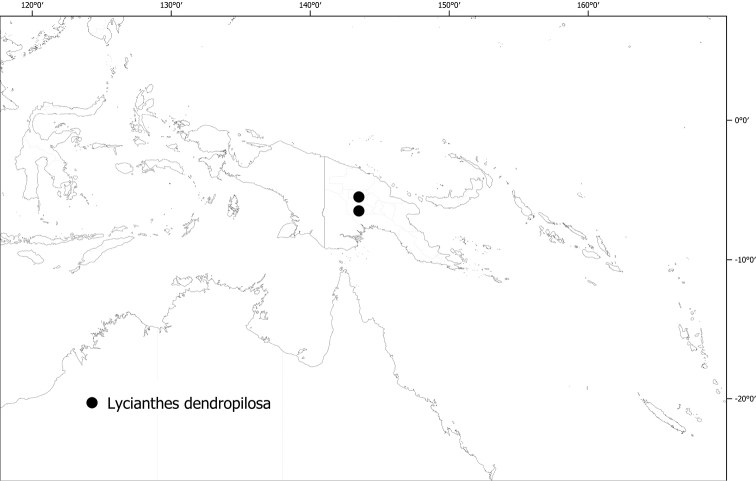
Distribution of *Lycianthesdendropilosa*.

**(Fig. 21).***Lycianthesdendropilosa* is endemic to New Guinea; it has only been collected in Papua New Guinea (Southern Highlands, Western).

#### Ecology and habitat.

*Lycianthesdendropilosa* has been collected in open areas near villages (*Symon & Katik 10691* from “near old garden site”), from 2,400 to 2,700 m elevation.

#### Common names.

None recorded.

#### Preliminary conservation assessment

**(IUCN 2020).**EOO (0 km^2^ - CR); AOO (8 km^2^ - CR). *Lycianthesdendropilosa* is known from only two localities and has been very rarely collected. It could be assessed as Data Deficient (DD), but given the threats to New Guinea forests more broadly I suggest it warrants assessment as Critically Endangered (CR [B1,2a,b(iii,iv)]); more collecting and a better understanding of its distribution are priorities.

#### Discussion.

*Lycianthesdendropilosa* is a distinctive species with small leaves, branched trichomes with densely congested branches, and 1–3 flowers per leaf axil. It shares trichome type with *L.bitteriana*, but that species is a coarse herb rather than a straggling shrub, has many flowers on a short inflorescence axis rather than 1–3 flowers per axil, and smaller flowers with copious interpetalar tissue (1–1.2 cm in diameter with interpetalar tissue in *L.bitteriana*, 1.5–2 cm in diameter without interpetalar tissue in *L.dendropilosa*). Fruits of *L.dendropilosa* are not yet known. Other than the type, that has thick, coriaceous leaves with somewhat revolute margins, the only other specimen I have seen that is attributable to this species (*Symon & Katik 10691*) is sterile; the leaves on this specimen are much thinner in texture and more elongate, indicating this may represent a juvenile pre-flowering individual.

#### Specimens examined.

Papua New Guinea. **Southern Highlands**: between Nol and Mendi, 24 km from Mendi, just after crest [“and o”], 2,000 m, 24 Jun 1977, *Symon & Katik 10691* (L, LAE).

### ﻿7. Lycianthes impar

Taxon classificationPlantaeSolanalesSolanaceae

(Warb.) Bitter, Abh. Naturwiss. Vereins Bremen 24 [preprint]: 504. 1919.

6B54B97B-522B-5AA3-AA69-251DE2F0BC51

Figs 22 , 23

Solanum impar Warb., Bot. Jahrb. Syst. 13: 415. 1891. Type. Indonesia. Papua Barat: Sigar [=Sekar], *O. Warburg 21244* (holotype: B, destroyed, no duplicates found). Indonesia. Papua: “Sg. Aëndosa bij Oeta” [near Oeta], 3 m, 4 Jul 1941, *Aët (exp. Lundquist) 407* (neotype, designated here: BO [acc. # 1579909; isoneotypes: BO [acc. # 1579910], L [L.2881729, L.2881730]).
Solanum ridleyanum Wernham, Trans. Linn. Soc. London 9: 119. 1916. Type. Indonesia. Papua: “Utakwa River to Mt. Carstenz [Puncak Jaya], alt. 1,100–2.500 ft, Camp III, IV” Dec 1912-Feb 1913, *C.B. Kloss s.n.* (lectotype, designated by Symon 1985, pg. 52 [as holotype]: BM [BM000778110]).

#### Type.

Based on *Solanumimpar* Warb.

**Figure 22.**
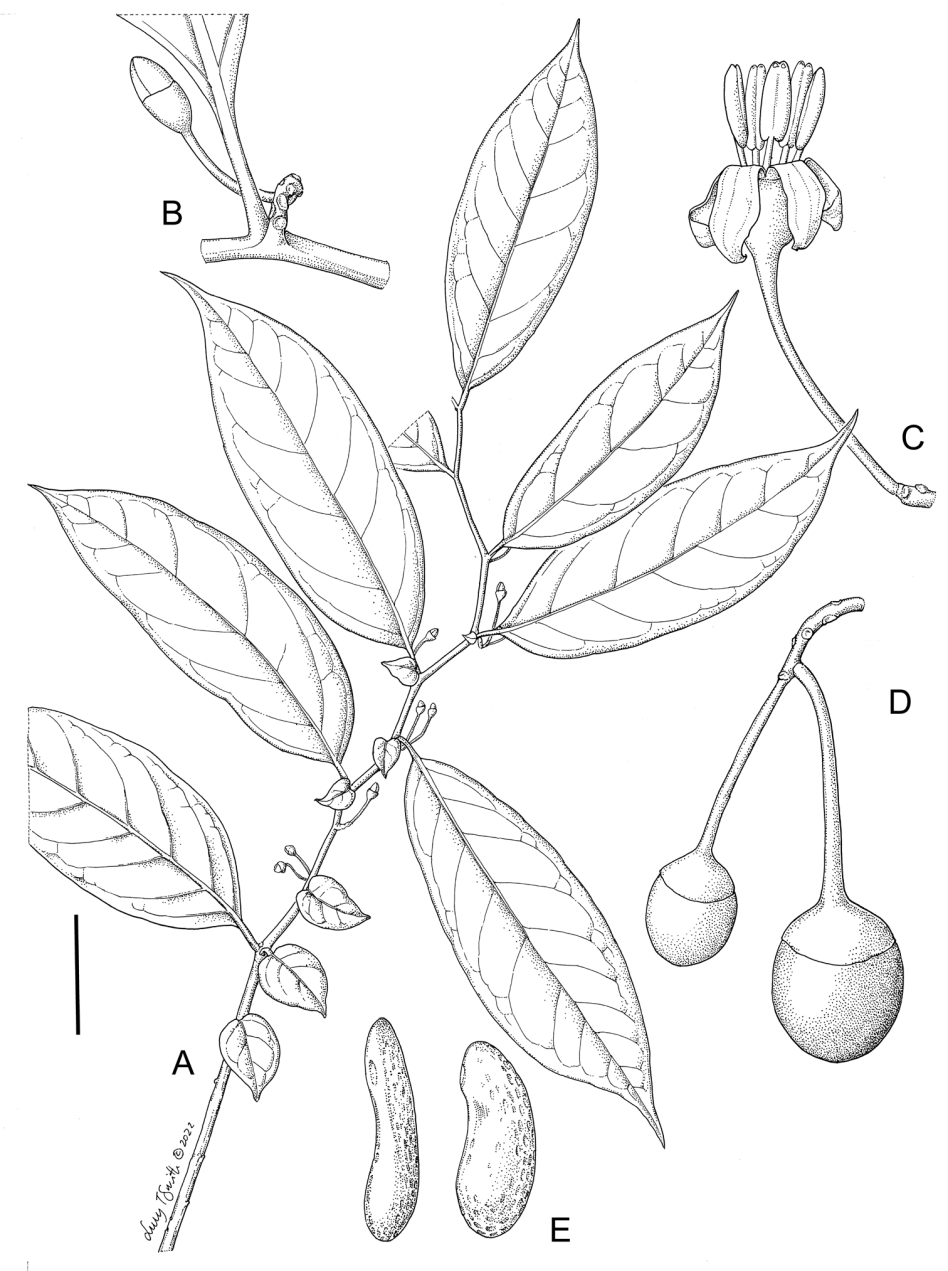
*Lycianthesimpar* (Warb.) Bitter **A** flowering branch **B** inflorescence with bud **C** short-styled flower **D** berries **E** seeds. (**A***Utteridge et al. 119*, **B***van Leeuwen 11108*, **C***Sands 7329*, **D, E***Puradyatmika et al. 10428*). Drawing by Lucy Smith. Scale bars: 4 cm (**A**); 5 mm (**B, C**); 0.75 cm (**D**); 1.4 mm (**E**).

#### Description.

Small trees or climbers, to 3.5 m tall; stems terete, sometimes with adventitious roots from the nodes (*Kloss s.n.*, 22 Nov 1912), moderately pubescent with transparent, antrorsely curled simple uniseriate 2–3-celled trichomes to 0.5 mm long, the basal cells sometimes enlarged; new growth densely papillate and pubescent with antrorse trichomes like those of the stems; bark of older stems pale grey-brown and somewhat corky and peeling. Sympodial units difoliate, the leaves geminate, the leaves of a pair differing in size and shape. Leaves simple; blades of major leaves 8.5–17 cm long, 2.9–6 cm wide, elliptic, discolorous, chartaceous or coriaceous; adaxial surfaces shiny, completely glabrous; abaxial surfaces glabrous; principal veins 8–10 pairs, prominent and pale yellow of reddish tan beneath; base acute; margins entire; apex acuminate; petiole 0.5–1 cm long, with a few simple antrorse trichomes like those of the stems at the very base; blades of minor leaves 0.8–1.5 cm long, 0.8–1.1 cm wide, orbicular to obcordate but quite variable in shape even within a single plant, similar in texture and pubescence to the majors; base cordate or truncate; margins entire; apex acute or rounded; petiole absent to less than 0.5 cm long, glabrous to absent. Inflorescences axillary with a short axis 0.4–1 cm long, 10–12-flowered, hanging under the leaves, only 1–2 flowers open at a time, glabrous and corky; pedicels at anthesis 0.6–0.9 cm long, ca. 0.5 mm in diameter at the base, ca. 1.25 mm in diameter at the apex, spreading or nodding, glabrous, articulated at the base pedicel scars closely packed, from the very base of the axis. Buds ellipsoid, the corolla ca. halfway exserted from the calyx tube before anthesis. Flowers 5-merous, only short-styled flowers seen, the plants possibly dioecious. Calyx tube 2.5–3 mm long, ca. 3 mm wide, elongate cup-shaped, thick and probably somewhat fleshy in live plants, purple, glabrous, without appendages, the rim slightly thinner and sparsely papillate. Corolla ca. 1.2 cm in diameter, purple or violet with the lobes white (fide *Sands et al. 7329*), stellate, lobed nearly to the base, interpetalar tissue absent, the lobes ca. 5 mm long, ca. 1.5 mm wide, spreading, thick and fleshy, both surfaces glabrous, densely papillate on tips and margins. Stamens equal; filament tube minute; free portion of the filaments 0.75–1 mm long, glabrous; anthers ca. 3 mm long, ca. 1 mm wide, ellipsoid, yellow, poricidal at the tips, the pores round, directed distally, not elongating to slits with age. Ovary conical, glabrous, vestigial (only seen in short-styled flowers); style and stigma not seen. Fruit an elongate berry, 0.7–1 cm long, ca. 0.6 cm wide, bright blue or purple-blue when ripe, whitish blue to almost white when immature (fide *Utteridge et al. 119*), the pericarp glabrous, thin, matte, opaque, the fruit flesh purple (fide *Utteridge et al. 119*); fruiting pedicels 1–1.2 cm long, ca. 0.75 mm in diameter at the base, ca. 2 mm in diameter at the apex, purple, pendent and hanging below the leaves; fruiting calyx a “cupule-like structure” (fide *Utteridge et al. 119*) subtending the fruit, bright purple or white (fide *Takeuchi 9181*), thick and probably fleshy in live plants. Seeds 10–20 per berry, ca. 4 mm long, ca. 1.5 mm wide, reniform, not markedly flattened, white (“white, kidney-shaped” in live plants fide *Utteridge et al. 119*) or pale tan, the surface minutely pitted, the testal cells rectangular in outline. Stone cells absent. Chromosome number not known.

**Figure 23.**
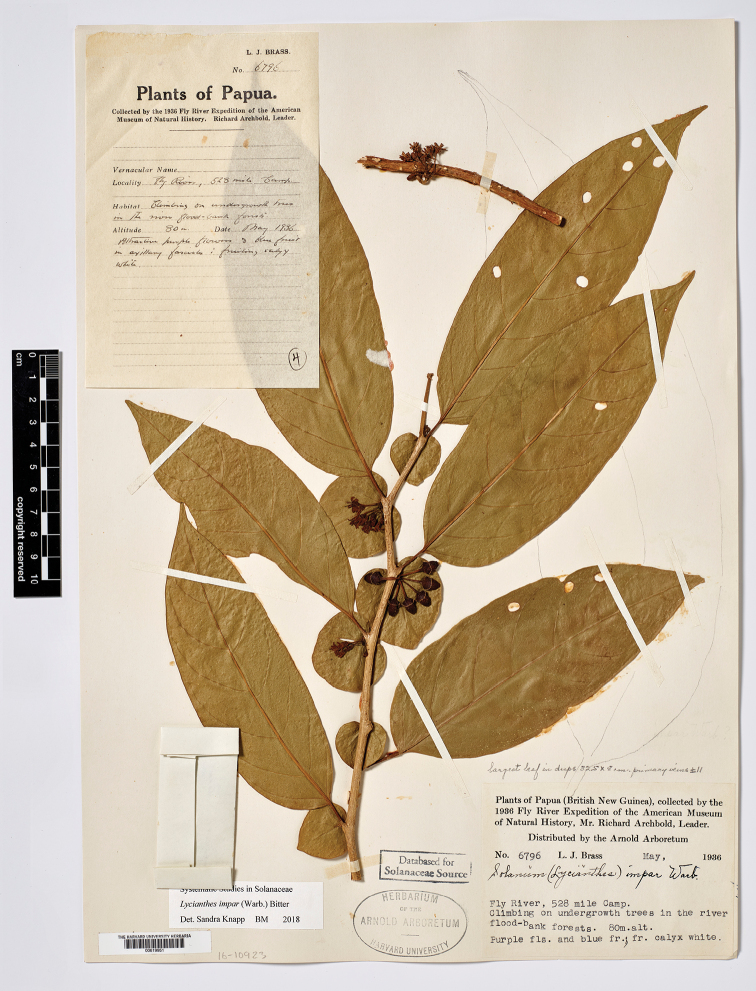
*Lycianthesimpar* herbarium specimen. Indonesia. Papua: *Brass 6796* (A). Courtesy of the Herbarium of the Arnold Arboretum of Harvard University, reproduced with permission.

#### Distribution

**Figure 24.**
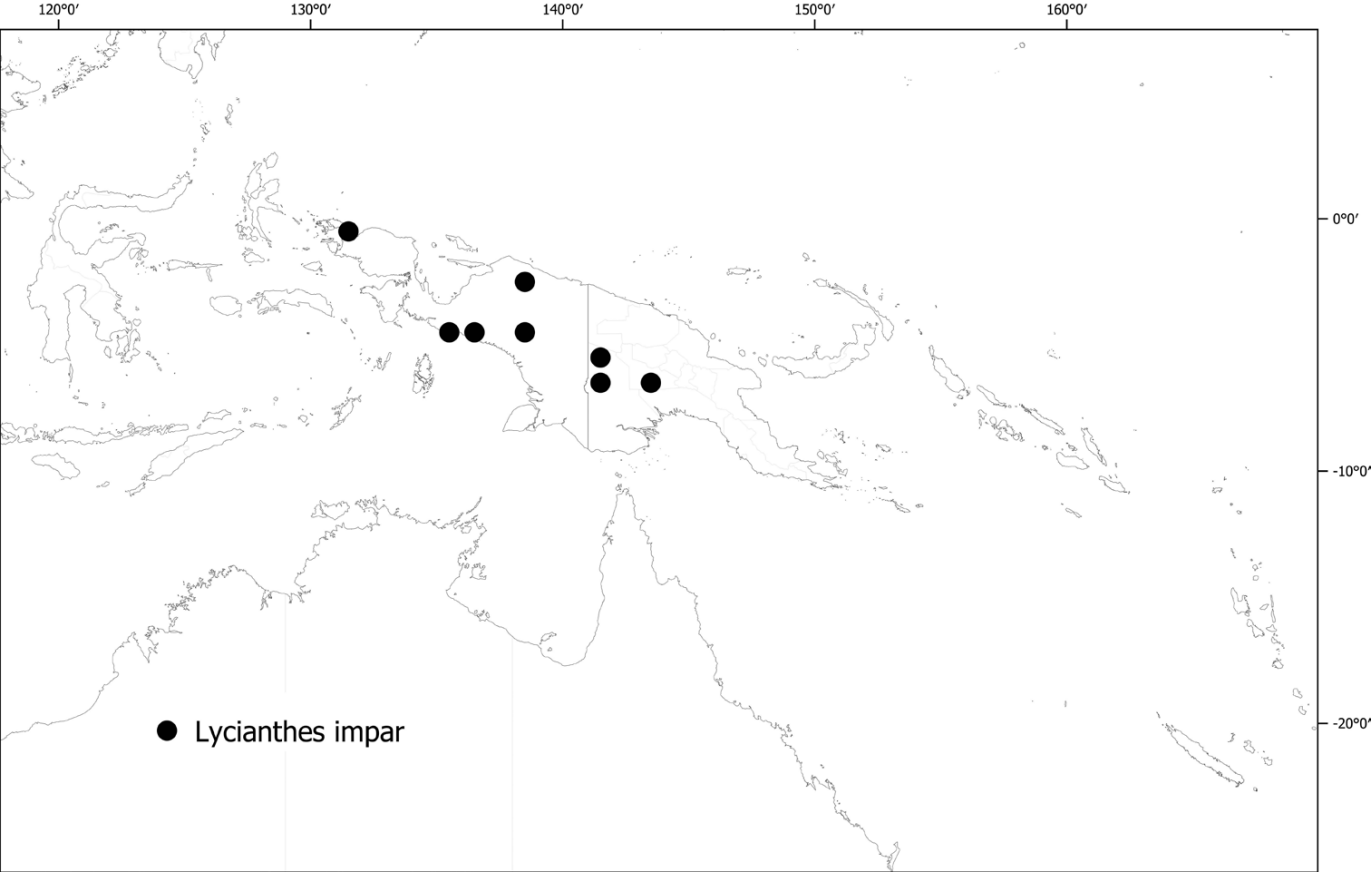
Distribution of *Lycianthesimpar*.

**(Fig. 24).***Lycianthesimpar* is endemic to the island of New Guinea; it has been collected in Papua New Guinea (Southern Highlands, Western) and Indonesia (Papua, Papua Barat).

#### Ecology and habitat.

*Lycianthesimpar* occurs in lowland rainforests on alluvium, between 10 and 210 m elevation.

#### Common names.

None recorded.

#### Preliminary conservation assessment

**(IUCN 2020).**EOO (257,568 km^2^ - LC); AOO (48 km^2^ - EN). *Lycianthesimpar* is known from more than seven localities in both Indonesia and Papua New Guinea, some of which are in protected areas (Parsch et al. 2022). It is a mostly lowland forest plant that is rather rarely collected; I suggest a preliminary threat status of Vulnerable (VU [B2a,b(iii, iv)])) based on the AOO, the number of localities and the threats for lowland forests more generally (Parsch et al. 2022).

#### Discussion.

*Lycianthesimpar* is a small tree or vine with strikingly differently shaped major and minor leaves. The orbicular or heart-shaped minor leaves are like those of *L.cladotrichota* and *L.peranomala*, but *L.cladotrichota* differs in leaves that are pubescent with dendritic trichomes; both *L.impar* and *L.peranomala* have glabrous leaves. Stem trichomes of *L.impar* are soft and somewhat tangled, while those of *L.peranomala* are stiff and markedly antrorse. *Lycianthesimpar* differs from both those species in having a short, sometimes forked, inflorescence axis; both *L.peranomala* and *L.cladotrichota* have strictly fasciculate flowers. *Lycianthesoliveriana* is similarly glabrous, but Warburg’s description of *Solanumimpar* clearly mentions a “peduncle” (inflorescence axis), distinguishing it clearly from *L.oliveriana* that lacks any axis. The soft, ovoid berries of *L.impar* are quite distinct from the woodier globose berries of *L.oliveriana*. The striking contrast in colour (purple/white) between the fleshy calyx cup and berry during fruit ripening mentioned on labels (e.g., *Utteridge et al. 119*, *Takeuchi 9181*) suggests the fruits are bird dispersed.

Symon (1985) mistakenly synonymised *Solanumridleyanum* with *Lycianthesmoszkowskii* (as *S.moszkowskii*) from which it differs in pubescence type (soft and tangled versus stiff and antrorse), inflorescence morphology (with a small sometimes forked axis versus strictly fasciculate), fruit shape and colour (purplish blue and elongate-ovoid versus globose and bright cherry red) and seed morphology (narrowly kidney-shaped versus strikingly winged in *L.moszkowskii*).

The type collection of *Solanumimpar* was made by Otto Warburg (*Warburg 21244*) in the McCluer Gulf area on the N coast of the Bomberai Peninsula in Papua Barat, where he made a short stop in January of 1889 on his world tour (van Steenis-Kruseman 1985). I have found no duplicates of his gathering, nor have I found any specimens collected from the same area. A collection made further southwest of the type locality near the town of Oeta in Papua by Aët (an Indonesian collector who collected widely in conjunction with various Dutch botanists, including on the Lundquist expedition, see van Steenis-Kruseman 1985) is a good match for the protologue, has flowers and fruit and is from similar habitat. I here designate *Aët 407* (BO acc. # 1579909) as the neotype for *L.impar*.

In the herbarium at Naturalis (L) Georg Bitter annotated herbarium specimens of *Lycianthesimpar* with designations he never published “*Lycianthesamblycarpa*” (*Versteeg 1351*) and “*Lycianthesradicans*” (*Lam 706*) (see Names not validly published). Another name not validly published “*Lycianthesfistulosa*” (with no attribution) is written on the duplicate of *Versteeg 1351* at BO (acc. # 1588531).

#### Specimens examined.

Indonesia. **Papua**: Rouffaerriiver, Motorbiv, 100 m, Nov 1926, *Docters van Leeuwen 11108* (K, L); Utakwa Expedition to Mt. Carstensz. Camp I-III, 22 Nov 1912, *Kloss s.n.* (BM); reg. flum. Mamberamo, pr. Pioniersbiv [Sungai Mamberano] [Geelvink Bay], 10 m, 23 Jul 1920, *Lam 706* (BO, K, L); Mimika Regency, Mount Jaya, PT-Freeport Indonesia Concession Area, between Kali Kopi levee (new E Levee) and the Kopi River, along black water stream flowering into the Kopi River (also black water), 210 m, 9 Mar 1999, *Puradyatmika et al. 10428* (A, K, MO); Mimika Regency, Mount Jaya, PT-Freeport Indonesia Concession Area, Kuala Kencana, near PT Freeport Indonesia Office, 50–100 m, 27 Aug 1998, *Sands 7329* (A, K); PT-Freeport Indonesia Concession Area, Rimba Irian Golf Course, on outskirts of Kuala Kencana, 10 m, 15 Mar 1999, *Utteridge et al. 119* (A, K, L, MO); “fluv. Lorentz” [Lorentz River = Sungai Unir] [Snow Mountains fide Symon 1985], 19 May 1907, *Versteeg 1137* (BO, L); fluv. Lorentz, prope ‘Sabang’ [Lorentz River, Sabang van Weel’s camp; Alkmaar bivouac] [Snow Mountains fide Symon 1985], 2 Jul 1907, *Versteeg 1351* (BO, L). **Papua Barat (West Papua)**: Sorong, near Klamono, 20 Aug 1948, *Pleyte 633* (BO, L).

Papua New Guinea. sin. loc. (“Solanum elegans Zp./N Guinea”), *Without Collector s.n.* (L). **Southern Highlands**: Kutubu patrol area, karst limestone NW of Yorokobaiu village, 600 m, 10 Sep 1993, *Takeuchi 9181* (A); **Western**: Fly River, 528 mile camp., May 1936, *Brass 6796* (A, BM, BRI, L); Kiunga subdistr., base camp (Ok Tedi river), 700 m, 2 Nov 1969, *Foreman & Galore NGF-45764* (L); Kiunga subdistr., Ok Tedi headwaters, near Kennecott field camp, 800 m, 29 Oct 1969, *Henty et al. NGF-42805* (L); Kiunga, Kiunga subdistr., 30 m, 9 Aug 1971, *Streimann & Katik LAE-51786* (L).

### ﻿8. Lycianthes kaernbachii

Taxon classificationPlantaeSolanalesSolanaceae

(Lauterb. & K.Schum.) Bitter, Abh. Naturwiss. Vereins Bremen 24 [preprint]: 504. 1919, as “kaernbachii”.

3CC7DA1C-AB25-5F94-AD96-66E1ECAE841F

Figs 25 , 26

Solanum kaernbachii Lauterb. & K.Schum., Fl. Schutzgeb. Südsee [Schumann & Lauterbach] 535. 1900 [“1901”], as “kaernbachii”. Type. Papua New Guinea. Morobe: “Kaiser Wilmhelmsland, Sattelberg, nach Selilo”, 800 m, 10 Dec 1893, *L. Kaernbach 77* (holotype: B [destroyed]). Papua New Guinea. Morobe: Sattelberg, 20 Dec 1935, *M.S. Clemens 1289* (neotype, designated here: L [L.2881629]; isoneotypes: G [G00415794], L [L.2881630]).
Solanum schlechterianum Bitter, Bot. Jahrb. Syst. 55: 111. 1917, as “Schlechterianum”. Type. Papua New Guinea. Madang: “Kaiser Wilhelmsland, Waldern am Djamu”, ca. 700 m, 24 Feb 1908, *F.R.R. Schlechter 17339* (holotype: B [B 10 0278656]; isotype: P [P00379697]).
Lycianthes schlechteriana (Bitter) Bitter, Abh. Naturwiss. Vereins Bremen 24 [preprint]: 504. 1919, as “Schlechteriana”. Type. Based on Solanumschlechterianum Bitter.

#### Type.

Based on *Solanumkaernbachii* Lauterb. & K.Schum.

**Figure 25.**
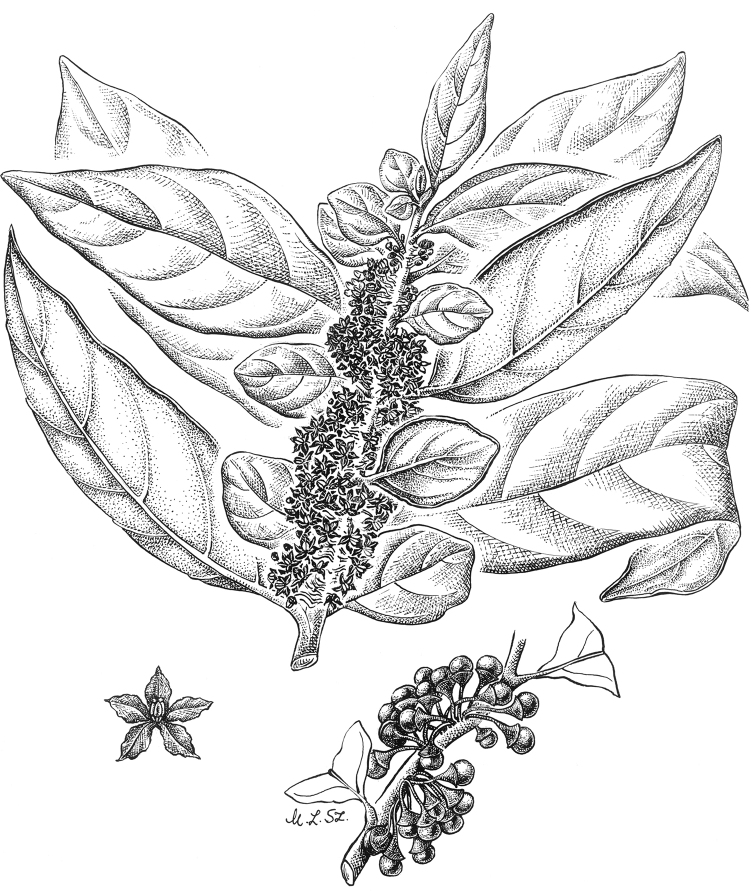
*Lyciantheskaernbachii* (Lauterb. & K.Schum.) Bitter. Drawing by M.L. Szent-Ivany, first published in Symon (1985: fig. 13, as *S.kaernbachii* Lauterb. & K.Schum.). Courtesy of the Board of the Botanic Gardens and State Herbarium (Adelaide, South Australia), reproduced with permission.

#### Description.

Woody climber or large liana, size not recorded; stems terete, densely to moderately pubescent with simple uniseriate 3–7-celled weak-walled, trichomes to 1 mm long, these occasionally forked, drying golden tan; new growth densely pubescent with simple uniseriate trichomes like those of the stems, bright golden tan in dry material; bark of older stems brown, not markedly glabrescent on twigs. Sympodial units difoliate, the leaves geminate, the leaves of a pair different in size and shape. Leaves simple; blades of major leaves 9–21 cm long, 3.5–11 cm wide, elliptic to narrowly elliptic, widest in the middle, discolorous, coriaceous (“greasy” fide *Kairo & Streimann NGF-30943*); adaxial surfaces shiny, glabrous to sparsely pubescent with simple uniseriate trichomes, these denser along the veins; abaxial surfaces moderately to densely and even pubescent with simple uniseriate trichomes to 1 mm long, these 3–7-celled, weak-walled, drying golden tan, denser along the veins; principal veins 7–10 pairs, the midrib somewhat keeled, the veins impressed above; base acute to somewhat cordate-truncate, oblique; margins entire, slightly revolute; apex acute to abruptly acuminate; petioles 0.5–3 cm long, densely pubescent with simple uniseriate trichomes like those of the stems and leaves; blades of minor leaves 2.5–6.5 cm long, 2–6 cm wide, broadly elliptic to orbicular, texture and pubescence like that of the major leaves; base rounded or cordate; margins entire to slightly revolute; apex rounded or obtuse; petioles absent to 0.6 cm long, pubescence like thatof the major leaves. Inflorescences paired rows of cauliflorous pedicels along the stem between the nodes and in leaf axils with groups of more than 10 flowers, many flowers open simultaneously, pubescence like that of the stems; pedicels 0.8–1.1 cm long, ca. 0.5 mm in diameter at the base, ca. 1 mm in diameter at the apex, slender and spreading, purple or green, glabrous to sparsely to moderately pubescent with golden simple uniseriate trichomes to 0.25 mm long, these less dense than stem pubescence, articulated at the base; pedicel scars closely spaced in rows along the stems. Buds ellipsoid, the corolla more or less strongly exserted from the calyx tube before anthesis. Flowers 5-merous, heterostylous and unisexual, individual specimens with either short-styled or long-styled flowers, the plants probably dioecious. Calyx tube 2–2.5 mm long, 2.5–3 mm in diameter, urn-shaped, thick and woody in dry material (fleshy in live plants?), slightly tuberculate, with scattered to denser simple uniseriate trichomes like those of the pedicels, without appendages, the rim constricted and thickened. Corolla 0.8–0.9 cm in diameter, creamy white to reddish cream (fragrant fide *Kairo & Streimann NGF-30943*), stellate, lobed nearly to the base, interpetalar tissue absent, the lobes 3–4 mm long, 1.2–1.5 mm wide, spreading or reflexed, thick and fleshy (live plants), glabrous adaxially, minutely puberulent abaxially, the tips and margins densely papillate, the tips strongly cucullate. Stamens equal; filament tube minute; free portion of the filaments 1–1.5 mm long, glabrous; anthers 1.5–2 mm long, ca. 1 mm wide, plumply ellipsoid, yellow, poricidal at the tips, the pores directed distally, not elongating to slits with age. Ovary globose to conical, glabrous, vestigial in short-styled flowers; style in short-styled flowers apparently absent, in long-styled flowers ca. 4.5 mm long, straight, glabrous; stigma strongly bifid, the lobes ca. 1 mm long, the surfaces minutely papillate. Fruit a globose berry, 0.5–0.6 cm in diameter, green, the pericarp glabrous, hard and somewhat woody in dried material, opaque; fruiting pedicels 0.9–1.1 cm long, ca. 1 mm in diameter at the base, ca. 2 mm in diameter at the apex, spreading, more or less woody, slightly tuberculate; fruiting calyx a cup around the basal third of the berry, woody in dry material (fleshy in live plants?), with a few simple uniseriate trichomes, somewhat tuberculate. Seeds 2–20 per berry, 3–3.5 mm long, 2–2.5 mm wide, flattened reniform, without a deep notch, reddish tan, the surfaces deeply pitted, especially near the margins, the testal cells sinuate in outline. Stone cells absent. Chromosome number not known.

**Figure 26.**
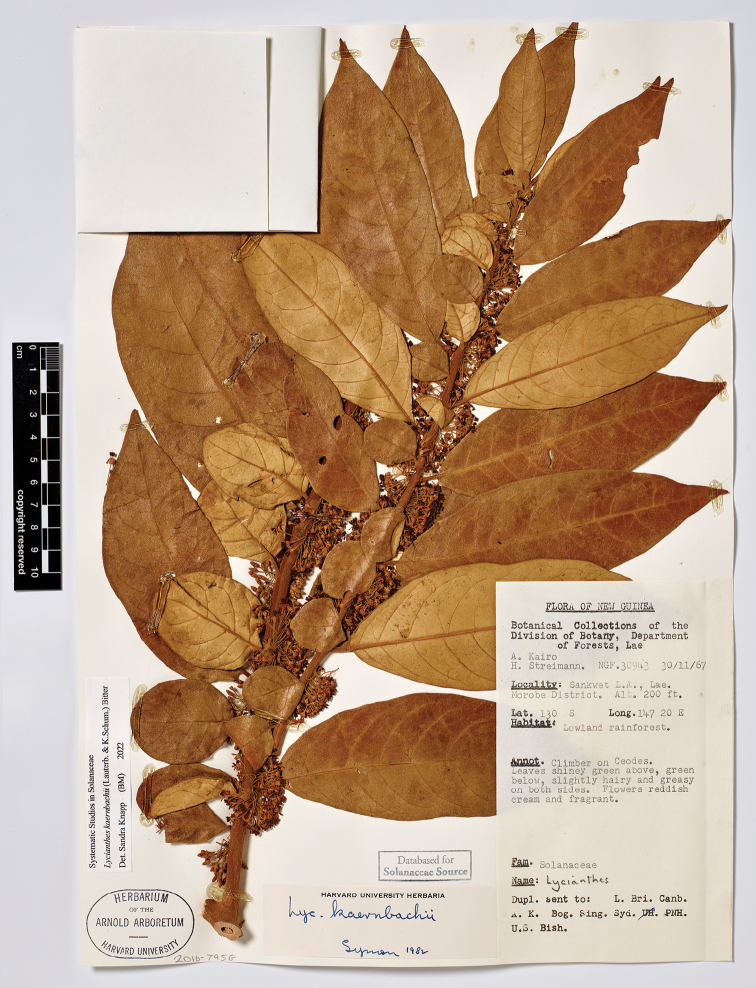
*Lyciantheskaernbachii* herbarium specimen. Papua New Guinea. Morobe: *Kairo & Streimann NGF-30943* (A). Courtesy of the Herbarium of the Arnold Arboretum of Harvard University, reproduced with permission.

#### Distribution

**Figure 27.**
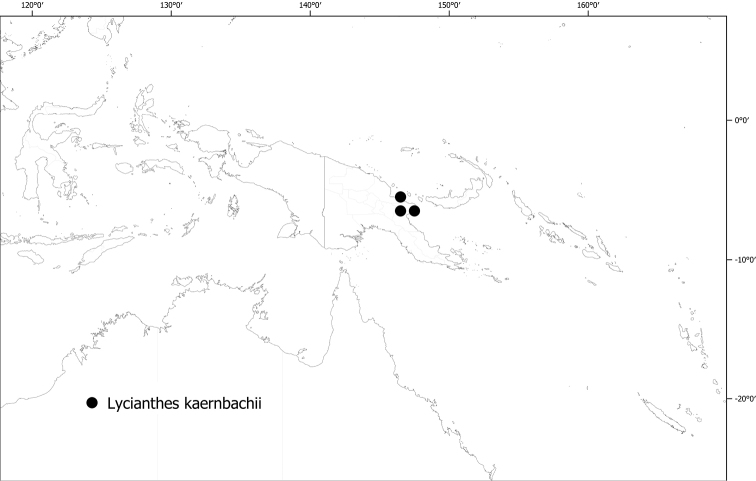
Distribution of *Lyciantheskaernbachii*.

**(Fig. 27).***Lyciantheskaernbachii* is endemic to the island of New Guinea; it has only been collected in Papua New Guinea (Madang, Morobe) in the eastern Finisterre Range.

#### Ecology and habitat.

*Lyciantheskaernbachii* is a plant of primary lowland rainforest, between 30 and 700 m elevation.

#### Common names.

Papua New Guinea. nigukwaa (Schumann and Lauterbach 1901).

#### Preliminary conservation assessment

**(IUCN 2020).**EOO (8,882 km^2^ - VU); AOO (24 km^2^ - EN). *Lyciantheskaernbachii* is known from five localities, all in the Finesterre range. Its small range, coupled with the lack of collections from protected areas suggest a preliminary threat status of Endangered (EN[B1,2ab(iii,iv)]) for *L.kaernbachii*.

#### Discussion.

*Lyciantheskaernbachii* is probably the most distinctive and unusual of the New Guinea species of *Lycianthes*. It is a large canopy liana with softly pubescent leaves and is cauliflorous, a character not seen elsewhere in the genus, and one that I have not seen elsewhere in the family. The inflorescence axis is apparently adnate to the stem, and the buds, flowers and fruits are in two parallel rows of varying length between the nodes. Flowers of *L.kaernbachii*, at less than 1 cm in diameter, are among the smallest (with *L.peranomala*) amongst New Guinea *Lycianthes*. Corolla lobes of *L.kaernbachii* are valvate in bud and thick and fleshy on live plants, becoming somewhat woody on dry specimens and completely lack interpetalar tissue. This type of corolla is shared with *L.oliveriana* and *L.peranomala*, but *L.kaernbachii* is easily distinguished from those taxa by its soft pubescence on abaxial leaf surfaces (*L.oliveriana* is glabrous and *L.peranomala* has sparse pubescence of stiff antrorse trichomes only along the veins) and its cauliflory (the other two species have strictly axillary inflorescences).

*Millar NGF-23365* is less pubescent than most of the other collections of *Lyciantheskaernbachii* I have seen and was tentatively identified as *L.oliveriana* by Symon based on a non-reproductive specimen; he later suggested it might be a mixed collection (Symon 1985). It does, however, have the distinctive linear cauliflory of *L.kaernbachii* on other duplicates and the pubescence of the new growth indicates it is not a mixed collection but rather an unusual sparsely pubescent plant of *L.kaernbachii*.

The type specimen of *Solanumkaernbachii* was held in Berlin and was destroyed in the Second World War, along with the many other types no longer extant (Vorontsova and Knapp 2010); no duplicates have been found. As a neotype (L.2881629) I have selected a collection from the same locality “Sattelberg” in eastern Papua New Guinea (*Clemens 1289*) that corresponds to the description in the protologue and is held in several herbaria.

The holotype of *Solanumschlechterianum* was not destroyed along with other New Guinea collections at Berlin; Bitter (1917) acknowledged the similarity of his new species with *L.kaernbachii* (as *S.kaernbachii*) and his illustration clearly shows the distinctive caulescent inflorescences.

#### Specimens examined.

Papua New Guinea. **Madang**: “Kaiser Wilhelmsland, Waldern am Djamu”, 700 m, 24 Apr 1908, *Schlechter 17339* (P). **Morobe**: Wareo, 610 m, 1 Jan 1935, *Clemens & Clemens 1426* (L); Sankwet L.A., Lae, 60 m, 30 Nov 1967, *Kairo & Streimann. NGF-30943* (A, E, K, L, NSW), 30 m, 5 Jan 1968, *Kairo & Emos NGF-30983* (A, E, K, L, LAE); Bupu village above Wampit, 762 m, 3 Mar 1964, *Millar NGF-23260* (K, L, LAE, NY, US); Bupu village above Wampit, 701 m, 4 Mar 1964, *Millar NGF-23365* (A, K, L, LAE, US); midway of Buso River, SE of Buso Camp, 18 Jun 1984, *Vinas & Kairo 308* (K).

### ﻿9. Lycianthes lucens

Taxon classificationPlantaeSolanalesSolanaceae

S.Knapp sp. nov.

EFFF79D4-C8AB-51E4-A7B8-554623D32452

urn:lsid:ipni.org:names:77305530-1

Figs 28 , 29

#### Diagnosis.

Like *L.vitiensis*, but differing in its shrub rather than tree habit, narrowly elliptic rather than elliptic leaves, its fewer-flowered inflorescences that are strictly axillary rather than many-flowered on an elongate axis, presence of triangular calyx appendages versus lack of appendages, anthers that are strictly poricidal versus anthers dehiscing through elongate slits or the pores lengthening with age, and smaller seeds (3 mm versus 4–5 mm long) that lack a distinct notch.

#### Type.

Papua New Guinea. New Ireland: Lihir Island [Niolam Island], Mount Tementa, above Palie Mission, Namatanai subprovince, 710 m, 7 Nov 1984, *O. Gideon LAE-57196* (holotype: LAE [acc. # 256314]; isotypes: K [K000922490], L [L.2882045]).

**Figure 28.**
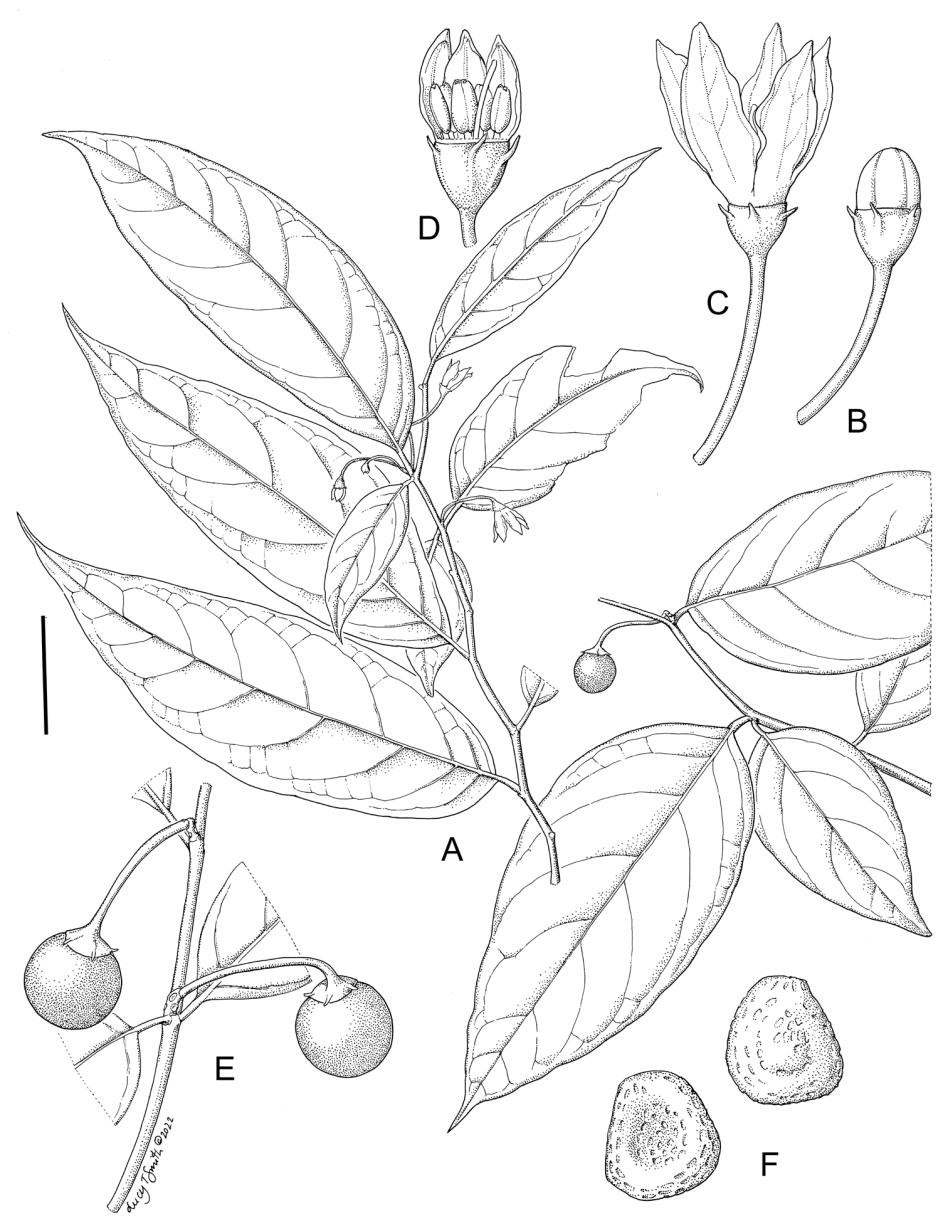
*Lyciantheslucens* S.Knapp **A** flowering branch **B** flower bud **C** flower **D** flower bud with petals removed to show stamens **E** fruiting branch **F** berries **G** seeds. (**A, C, E, G***Sands et al. 2230***B***Sands et al. 2073*). Drawing by Lucy Smith. Scale bars: 4 cm (**A, E**); 7 mm (**B, C**); 6 mm (**D**); 2 cm (**F**); 2.5 mm (**G**).

#### Description.

Shrubs 0.5–1.3 m tall; stems terete, glabrous or with very sparse simple or forked uniseriate papillae or trichomes, these sometimes to 5-celled, less than 0.2 mm long, drying reddish gold, the papillae possible glandular, a single collection with adventitious roots from the stem, these filamentous (*Croft et al. LAE-71421*); new growth glabrous or sparsely papillate, the papillae with darker (glandular?) terminal cells; bark of older stems white to whitish grey, somewhat wrinkled and ridged from drying. Sympodial units difoliate, the leaves geminate, sometimes appearing unifoliate due to the minor leaves abscising, the leaves of a pair similar in shape but not in size. Leaves simple; blades of major leaves 9–15 cm long, 3–7 cm wide, narrowly elliptic, discolorous, membranous; adaxial surfaces very shiny, glabrous; abaxial surfaces glabrous, paler; principal veins 4–7 pairs, yellowish green beneath (in dry material), the midrib not keeled, the veins arching but not forming distinct inframarginal loops; base acute or sometimes somewhat rounded; margins entire; apex acuminate, rarely acute; petioles 0.5–1.5 cm long, completely glabrous; blades of minor leaves 3–9 cm long, 1–4.5 cm wide, shape, texture and pubescence like that of the majors; base acute or sometimes somewhat rounded; margins entire; apex acuminate, rarely acute; petioles 0.3–0.5 cm long, glabrous. Inflorescences axillary fascicles or 1–4 flowers (most often 2–3 flowers), only a single flower open at a time, completely glabrous; pedicels at anthesis 2.5–4 cm long, markedly lengthening just before anthesis, 0.5–0.75 mm in diameter at the base, 1–2 mm in diameter at the apex, pale green or white, spreading or perhaps nodding, completely glabrous and shiny, articulated at the base; pedicel scars in a tight cluster in the leaf axils. Buds ellipsoid, the corolla ca. halfway exserted from the calyx tube before anthesis, completely included in young buds. Flowers 5-merous, heterostylous and unisexual, specimens with either short-styled flowers or long-styled flowers and fruit, the plants perhaps dioecious, the short-styled flowers marginally smaller than long-styled flowers. Calyx tube 2–4 mm long, shorter in short-styled flowers, 2.5–3.5 mm in diameter, openly cup-shaped, white flushed with purple (fide *Sands et al. 1966*), with (3)4–5 triangular appendages 1–1.5 mm long, ca. 0.5 mm wide, oriented perpendicular to the calyx tube, these usually of differing lengths in a single flower, emerging 0.5–1 mm from the calyx rim, in very young buds the appendages sometimes papillate. Corolla 1.1–2.4 cm in diameter, short-styled flowers at the smaller size end, white or pale purple, stellate, lobed ca. 3/4 of the way to the base, interpetalar tissue present, the lobes 4–8 mm long, 3–4 mm wide, spreading or slightly cupped, membranous, glabrous on both surfaces, the tips and distal portions of the margins densely papillate. Stamens equal; filament tube minute; free portion of the filaments ca. 1.5 mm long, glabrous; anthers 2.5–3 mm long, ca. 1.5 mm wide, ellipsoid, yellow, poricidal at the tips, the pores distally directed, round and not elongating to slits with age. Ovary conical, glabrous, vestigial in short-styled flowers; style in short-styled flowers vestigial, in long-styled flowers 6–6.5 mm long, straight, glabrous; stigma clavate, the surfaces minutely papillate. Fruit a globose berry, 1.2–1.5 cm in diameter, deep red when ripe, green to orange-red to deep red through development, the pericarp glabrous, thin, shiny, translucent; fruiting pedicels 3–4 cm long, longer in mature fruit, 1–1.5 mm in diameter at the base, 3–3.5 mm in diameter at the apex, not markedly woody, pendent (?), pale green; fruiting calyx a spreading plate beneath the fruit, stiff and perhaps fleshy or woody in live plants, the veins leading to the calyx appendages enlarged and prominent. Seeds 50–100 per berry, ca. 3 mm long, 2.5–3 mm wide, flattened and somewhat round with one straight edge in outline, reddish tan, the margins darker, the surfaces deeply pitted, the testal cells pentagonal in outline. Stone cells absent. Chromosome number not known.

**Figure 29.**
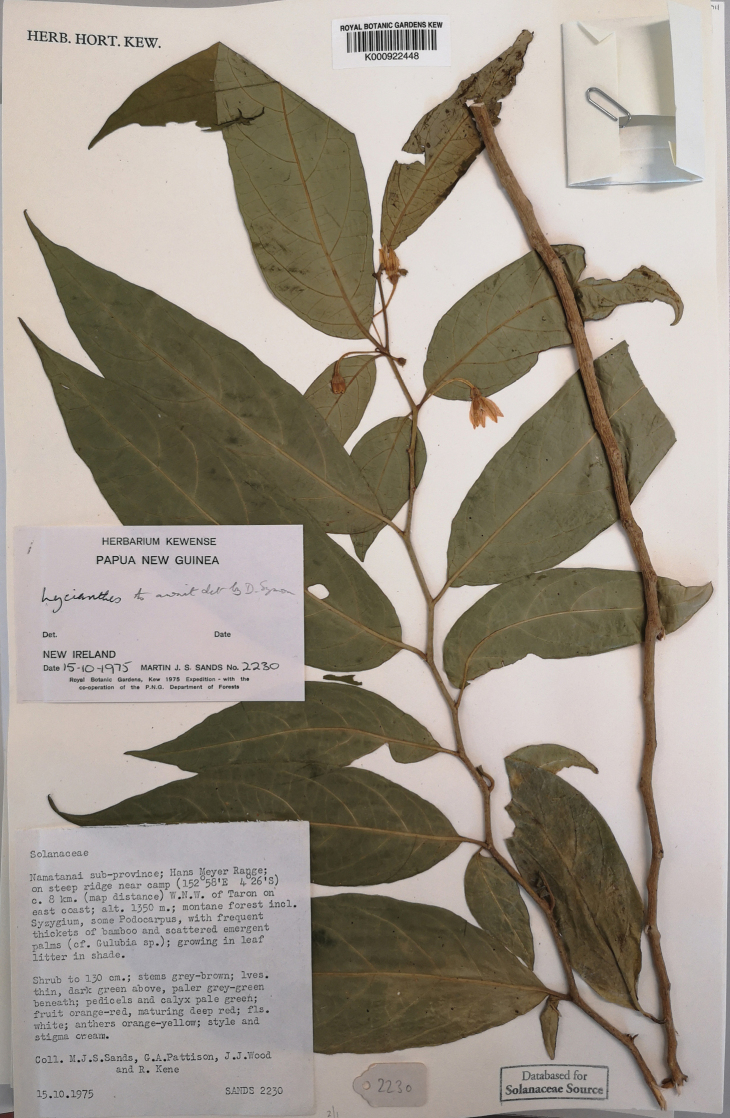
*Lyciantheslucens* herbarium specimen. Papua New Guinea. New Ireland: *Sands et al. 2030* (K000922448). Courtesy of the Trustees of the Royal Botanic Garden, Kew, reproduced with permission.

#### Distribution

**Figure 30.**
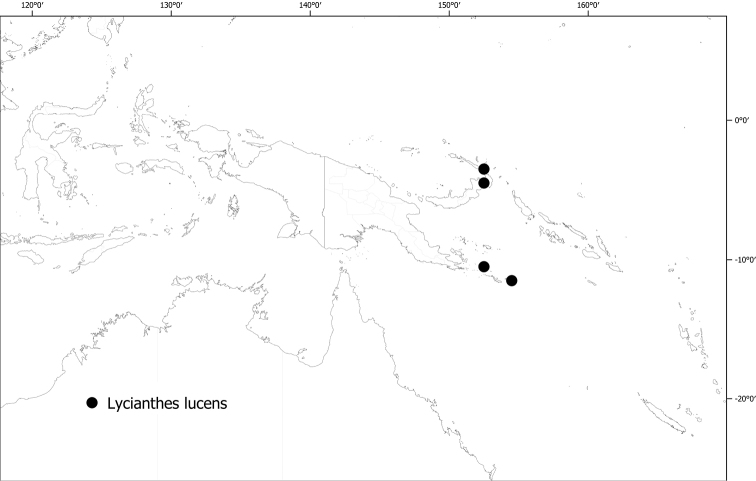
Distribution of *Lyciantheslucens*.

**(Fig. 30).***Lyciantheslucens* is endemic to the islands east of the main island of New Guinea; it has been collected on New Ireland, Lihir Island and the islands of the Louisiade Archipelago (Rossel and West Misima Islands).

#### Ecology and habitat.

*Lyciantheslucens* grows in the shaded understory of montane rainforests, from 700 to 1,350 m elevation.

#### Common names.

None recorded.

#### Preliminary conservation assessment

**(IUCN 2020).**EOO (70,602 km^2^ - LC); AOO (24 km^2^ - EN). *Lyciantheslucens* is known from only four localities on widely separated islands; the large EOO consists mostly of uninhabitable ocean. Gold mining on Lihir island is a significant threat for the forest habitat of this species; I suggest a threat status of Endangered (EN [B1,2a, b(iii,iv)]) for *L.lucens*.

#### Discussion.

*Lyciantheslucens* is a beautiful plant, with large flowers, bright red berries and shiny leaves. The species epithet is derived from the shiny, glabrous leaves. Symon (1985) cited *Katik et al. LAE-70928* from Rossel Island as *L.belensis* (as *S.belense*), and other specimens in herbaria have been annotated as that species by him. *Lyciantheslucens* resembles *L.belensis* in its shiny leaves and mostly axillary inflorescences with few, large flowers but it differs in its glabrous stems and adaxial leaf venation (versus stem and abaxial veins softly pubescent in *L.belensis*) and triangular calyx appendages to 1.5 mm long that are perpendicular to the calyx tube (versus appendages that are either absent or less than 0.75 mm long and not obviously perpendicular to the calyx tube in *L.belensis*). The calyx rim in *L.belensis* is thin, transparent and ruffly (see Fig. 8), while that of *L.lucens* is strictly truncate with distinct teeth and not markedly ruffly (Fig. 28). *Lycianthesbelensis* is a plant of high elevations in the central ranges, while *L.lucens* is a plant of montane forests only found on the islands to the east of New Guinea proper.

*Lycianthesmultifolia* has shiny leaves somewhat similar to those of *L.lucens* but it has smaller flowers (1.4–1.6 cm in diameter versus ca. 2.4 cm in diameter in *L.lucens*) and densely pubescent stems and venation (versus glabrous in *L.lucens*). Sympodial units of *L.lucens* are strictly difoliate, while those of *L.multifolia* often have more than two leaves at a node.

The only other *Lycianthes* species found as far east in the New Guinea and Pacific area as *L.lucens* is *L.vitiensis* from the island of Bougainville to Samoa. *Lyciantheslucens* differs from *L.vitiensis* in habit (shrub versus tree) and inflorescence morphology (axillary fascicles versus with a distinct axis), in addition to the other characters mentioned in the diagnosis.

Like other *Lycianthes* species in the area, *L.lucens* appears to be dioecious, with individual specimens bearing either short-styled (staminate) flowers or long-styled (pistillate or bisexual) flowers and fruit.

#### Specimens examined (paratypes).

Papua New Guinea. **Milne Bay**: Rossel Island, Mt. Rossel, S. slopes, 700 m, 19 Oct 1956, *Brass 28494* (A, L); Mt. Oiatau, West Misima Island, subprov. Misima, 700 m, 23 Mar 1979, *Croft LAE-71421* (A, K, L, LAE); Mount Rossel, Rossel Island, subprov. Misima, 780 m, 18 Mar 1979, *Katik et al. LAE-70928* (A, E, K, L, LAE), 19 Mar 1979, *Katik et al. LAE-70954* (K, L, LAE). **New Ireland**: Lihir Island [Niolam Island], Mount Tementa, above Palie Mission, Namatanai subprovince, 710 m, 7 Nov 1984, *Gideon LAE-57196* (K, LAE, L); Hans Meyer Range, on steep ridge below camp, ca. 8 km (map distance) WNW of Taron on east coast, Namatanai subprov., 1,260 m, 9 Oct 1975, *Sands et al. 1966* (K), 1,075 m, 8 Oct 1975, *Sands et al. 2073* (K), 1,350 m, 15 Oct 1975, *Sands et al. 2230* (K).

### ﻿10. Lycianthes moszkowskii

Taxon classificationPlantaeSolanalesSolanaceae

(Bitter) Bitter, Abh. Naturwiss. Vereins Bremen 24 [preprint]: 504. 1919, as “ Moszkowskii”.

78C7CFBC-9BC4-5B6B-901E-85C2218BB4F8

Figs 31 , 32

Solanum moszkowskii Bitter, Bot. Jarhb. Syst. 55: 103. 1917, as “*Moszkowskii*”. Type. Indonesia. Papua: “Van Rees, Naumoni” [Van Rees Mtns., Naumoni bivouac], Oct 1910, *M. Moszkowski 368* (holotype: B [destroyed], no duplicates found). Papua New Guinea. Morobe: Aseki road, beside Aseki road, 2,000 m, 27 Jul 1977, *K. Rau*, *W. Moi & Arenaso 73* (neotype, designated here: LAE [acc. # 235844]; isoneotypes: A, K [K001153713], L [L.2899568]).
Solanum acuminatissimum Merr. & L.M.Perry, J. Arn. Arb. 30: 49. 1949. Type. Indonesia. Papua: 15 km SW of Bernhard Camp, Idenburg [Taritaru] River, 1,800 m, Jan 1939, *L.J. Brass 12290* (holotype: A [00077831]; isotypes: L [L0003602], LAE [acc. # 229600]).

#### Type.

Based on *Solanummoszkowskii* Bitter.

**Figure 31.**
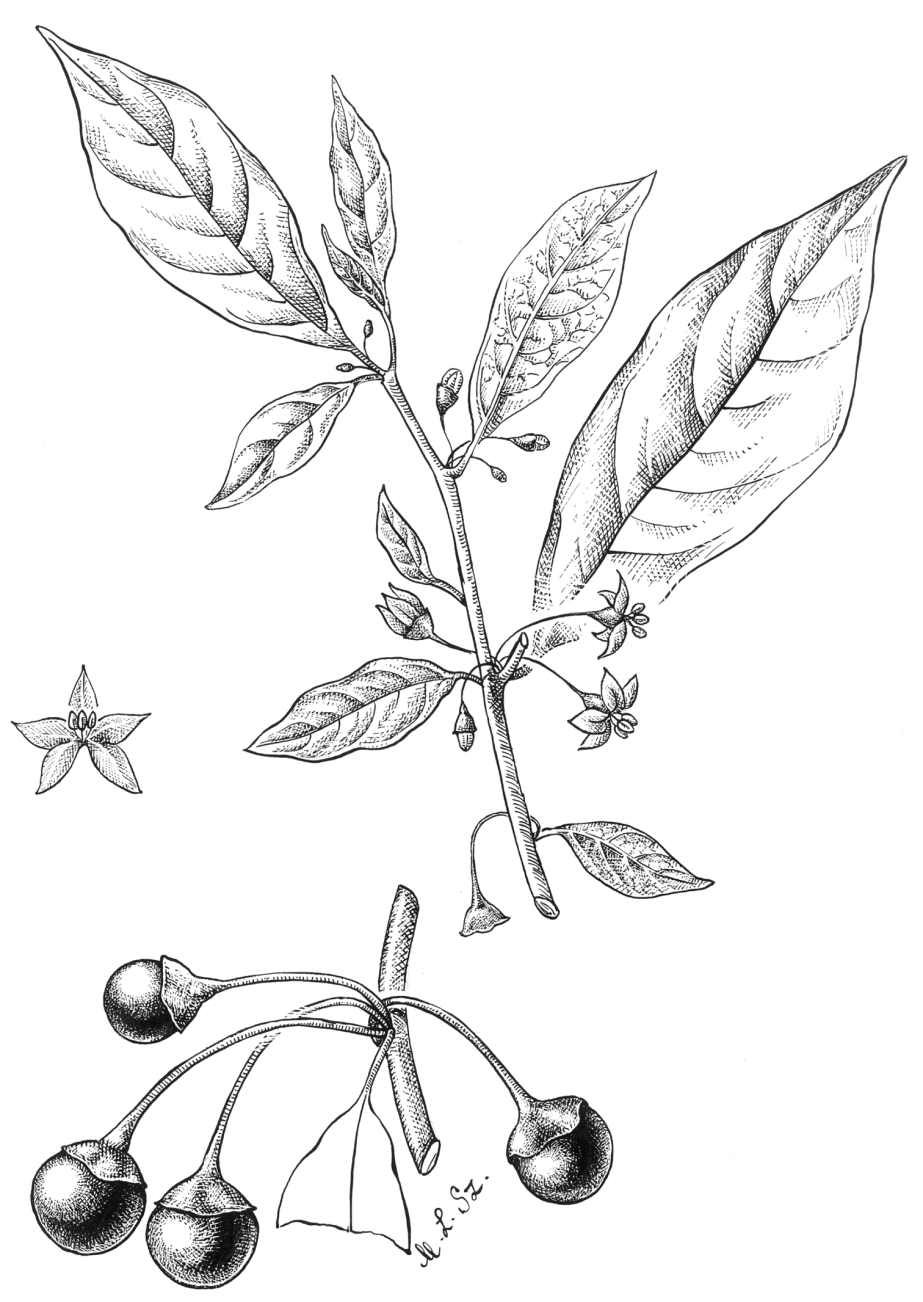
*Lycianthesmoszkowskii* (Bitter) Bitter. Drawing by M.L. Szent-Ivany, first published in Symon (1985: fig. 15, as *S.moszkowskii* Bitter). Courtesy of the Board of the Botanic Gardens and State Herbarium (Adelaide, South Australia), reproduced with permission.

#### Description.

Erect or sprawling (climbing?) shrubs, 0.7–4.5 m tall; stems terete and very lightly ridged with decurrent leaf bases, glabrous or sparsely to densely pubescent with stiff simple uniseriate 2–4-celled trichomes to 1 mm long, these spreading or sometimes antrorse, the bases sometimes enlarged and pustulate; new growth densely pubescent with stiff antrorse simple uniseriate trichomes to 1 mm long, these soon deciduous in nearly glabrous plants; bark of older stems dark brown, glabrescent. Sympodial units difoliate, the leaves geminate, the leaves of a pair differing in size and shape. Leaves simple; blades of major leaves 7–11 cm long, 3–8.5 cm wide, narrowly obovate to obelliptic, widest in the distal half, discolorous, chartaceous or coriaceous, often drying dark brown; adaxial surfaces shiny, glabrous, the more pubescent plants with stiff antrorse trichomes along the somewhat keeled midrib; abaxial surfaces sparsely to moderately and evenly pubescent on veins and lamina with stiff simple uniseriate trichomes like those of the stems, these not markedly antrorse; principal veins 5–6 pairs, impressed above, the midrib slightly keeled; base acute; margins entire, sometimes somewhat ciliate with stiff simple uniseriate trichomes; apex acuminate or abruptly acuminate; petioles 0.5–1 cm long, glabrous or moderately pubescent with stiff simple uniseriate trichomes like those of the stems; blades of minor leaves 2.5–7 cm long, 1–4 cm wide, elliptic, texture and pubescence like that of the major leaves; base acute; margins entire; apex acute to acuminate; petiole absent to 0.5 cm long, glabrous or pubescent like the stems. Inflorescences axillary fascicles of 1–3 flowers, only one open at a time, pubescent with antrorse or spreading stiff uniseriate trichomes like those of the stems; pedicels at anthesis 1.8–2 cm long, ca. 0.5 mm in diameter at the base, ca. 2 mm in diameter at the apex, spreading, thick and apparently fleshy in live plants, green (?), glabrous to sparsely pubescent with antrorse simple uniseriate trichomes like those of the stems, articulated at the base; pedicel scars closely and tightly packed in the leaf axils. Buds globose, later broadly elliptic, the corolla included in the calyx tube until just before anthesis. Flowers 5-merous, heterostylous and apparently unisexual, specimens either have all short-styled flowers or all fruit, the plants possibly dioecious. Calyx tube 3–3.5 mm long, 3.5–5 mm in diameter, cup-shaped, thick and coriaceous, apparently somewhat fleshy in live plants, glabrous to sparsely pubescent with stiff antrorse simple uniseriate trichomes like those of the pedicels, appendages absent, the rim slightly thickened. Corolla 2–2.5 cm in diameter, white or purplish cream, stellate, lobed nearly to the base, interpetalar tissue absent or merely a thin margin on the corolla lobe, the lobes 1–12 mm long, 4–4.5 mm wide, spreading or reflexed, thick and fleshy, glabrous on both surfaces but densely papillate at the tips and margins, the tips cucullate. Stamens equal; filament tube minute; free portion of the filaments 1–2 mm long, glabrous; anthers ca. 3 mm long, ca. 2 mm wide, ellipsoid and slightly tapering, yellow, poricidal at the tips, the pores distally directed, not elongating to slits with age. Ovary conical to globose, glabrous; style in short-styled flowers ca. 1 mm long, in long-styled flowers ca. 5 mm long, straight, glabrous; stigma broadly capitate, the surfaces minutely papillate. Fruit a globose berry, 1.3–1.5 cm in diameter, bright red when ripe, the pericarp glabrous, thin, matte to slightly shiny, opaque; fruiting pedicels (2- immature) 3–3.5 cm long, ca. 1 mm in diameter at the base, 2.5–3 mm in diameter at the apex, spreading or pendent, not markedly woody, green (?); fruiting calyx a spreading saucer beneath the fruit, somewhat corky and rugose (fleshy in live plants?). Seeds 30–40 per berry, 5–6 mm long, 5–6 mm wide, round with a prominent wing 1.5–2 mm wide (total measurement includes the wing), yellowish tan or straw-coloured, the surfaces very shallowly pitted, the testal cells more or less sinuate in outline. Stone cells absent. Chromosome number not known.

**Figure 32.**
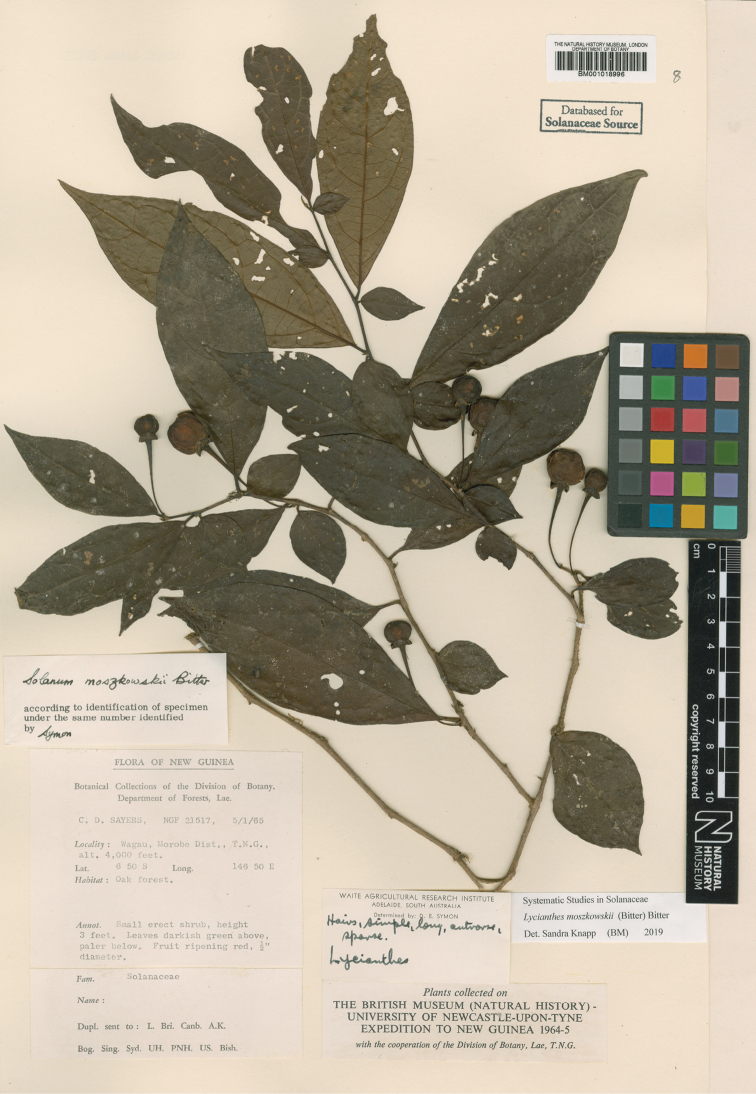
*Lycianthesmoszkowskii* herbarium specimen. Papua New Guinea. Morobe: *Sayers NGF-21517* (BM001018996). Courtesy of the Trustees of the Natural History Museum, London, reproduced with permission.

#### Distribution

**Figure 33.**
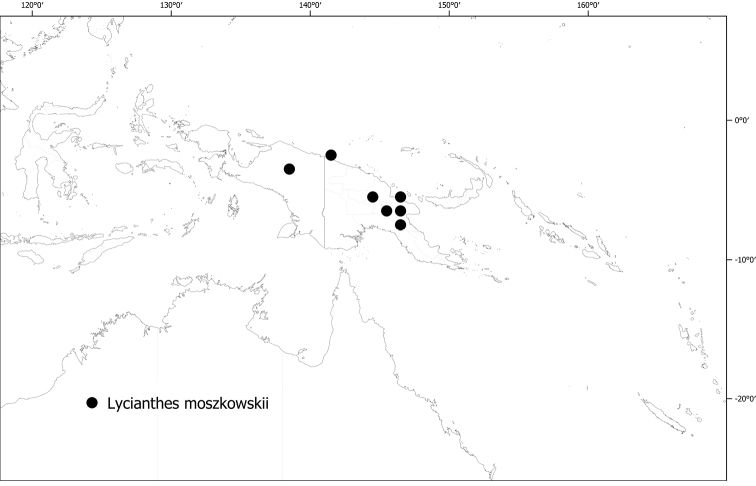
Distribution of *Lycianthesmoszkowskii*.

**(Fig. 33).***Lycianthesmoszkowskii* is endemic to the island of New Guinea; it has been collected in Papua New Guinea (Eastern Highlands, Madang, Morobe, West Sepik, Western Highlands) and Indonesia (Papua).

#### Ecology and habitat.

*Lycianthesmoszkowskii* is a plant of several types of primary rainforests, oak and *Castanopsis* forests, mossy montane forests and mid-montane forests; it occurs from 600 to 2,000 m elevation.

#### Common names.

None recorded.

#### Preliminary conservation assessment

**(IUCN 2020).**EOO (165,342 km^2^ - LC); AOO (80 km^2^ - EN). *Lycianthesmoszkowskii* is known from eight localities, mostly in Papua New Guinea with some collections from protected areas in the Bulolo region (McAdams National Park). I propose a preliminary threat status of Vulnerable (VU [B2a, b(iii,iv)]) for *L.moszkowskii*.

#### Discussion.

*Lycianthesmoszkowskii* is one of the more widely distributed species of *Lycianthes* on the island of New Guinea, only *L.oliveriana* is more widely distributed. The obovoid leaves, large (2–2.5 cm in diameter) flowers, large berries (to 1.5 cm in diameter) on long fruiting pedicels and large seeds (ca. 6 mm in diameter) with a prominent wing are all found only in *L.moszkowskii* and serve to distinguish it from all other species on New Guinea. *Lycianthesmoszkowskii* is very variable in pubescence density; a few specimens are densely and evenly pubescent over the entire abaxial leaf surface, while others have trichomes confined to along the midrib or lack trichomes entirely; branches and leaves are usually glabrescent with age and very lightly ridged from the somewhat decurrent leaf bases. The trichomes (if present) are stiff and usually spreading, rather than strongly antrorse, as is common on other species with stiff trichomes (e.g., *L.peranomala*, *L.rostellata*). The type collection was a branch with immature berries and the protologue of *S.moszkowskii* does not mention any pubescence (Bitter 1917).

Many specimens of *Lycianthesmoszkowskii* are identified as “Lycianthes cf. S.belense” by D.E. Symon in various herbaria, especially at the Naturalis Biodiversity Center (e.g. *Sayers NGF-21517*, L.2859575); care should be taken with early determinations of New Guinea *Lycianthes* on old annotation labels. Symon (1985) suggested the two species were closely related and they certainly are very similar. *Lycianthesbelensis* has similar inflorescences with few, large flowers and calyces with no appendages but differs from *L.moszkowskii* in its soft, curling pubescence (versus stiff spreading trichomes of *L.moszkowskii*) and its calyx with tiny appendages and a rim that is transparent and appears ruffly (versus a truncate calyx with no appendages in *L.moszkowskii*). The flowers of *L.moszkowskii* are more stellate with less interpetalar tissue than those of *L.belensis*. Seeds of *L.belensis* are not known, if they are strongly winged like those of *L.moszkowskii* this would be another shared character.

Symon (1985) suggested that *Lycianthesmoszkowskii* (as *Solanummoszkowskii*) had horticultural potential for its “handsome leaves and large red fruits, looking rather like cherries.” He had not seen long-styled (“female”) flowers, but in common with many other New Guinea *Lycianthes*, they are present on specimens with fruit, but not on specimens with short-styled flowers, suggesting this species is dioecious.

The type of *Solanummoszkowskii* was collected in the Van Rees range in northern Papua (Indonesia), an isolated range north of the main central spine of New Guinea; they are the lowest of the ten outlier mountain ranges along the north coast (Diamond and Bishop 2021) and have been very rarely explored and the area is sparsely populated. The German zoologist Max Moszkowski lost all his first set of collections from the Van Rees Mountains in and accident that occurred while descending the Edi Falls on the Mamberano River (van Steenis-Kruseman 1985). The specimen seen by Bitter (1917) was collected on the second attempt to ascend the river. His collections were all sent to Berlin and no duplicates of the type collection of *S.moszkowskii* have been found, despite intensive searches. In the protologue Bitter (1917) compares *L.moszkowskii* (as *S.moszkowskii*) to *L.impar* (as *S.impar*) and differentiates them based on the sessile, few-flowered inflorescence of *L.moszkowskii*, but he also states that they cannot be distinguished without flowers. In designating a neotype for *S.moszkowskii* that is in accordance with the protologue I have selected a collection with glabrous, slightly ridged stems, narrowly elliptic leaves, and that is in fruit (*Rau et al. 73*, neotype in LAE); it is unfortunate that it is not from nearer the type locality, but selection of *Rau et al. 73* in accordance with the morphology described in the protologue and maintains the circumscription of *L.moszkowskii* as established by Symon (1985). The collection *Streimann NGF-52968* is from nearer the type locality (in the province of Sanduan near the Papua border) and has immature fruit with the distinctive winged seeds of *L.moszkowskii* but is not in accordance with the protologue as the new growth is densely pubescent.

#### Specimens examined.

Indonesia. **Papua**: 15 km. southwest of Bernhard Camp, Idenburg River [=Taritatu River], small clearing in the mossy forest, 1,800 m, Jan 1939, *Brass 12290* (A, L, LAE).

Papua New Guinea. **Eastern Highlands**: Mount Piora, Kainantu subprov., above Habi’ina village on lower slopes of Mt. Piora, 2,125 m, 7 Sep 1995, *Sands et al. 1751* (K). **Madang**: Track between Budemu and Moro villages, S side of Finisterre Range, eastern Madang District., 21 Oct 1964, *Pullen 6011* (BM, LAE); Sewe, Saidor subdistrict, 2,286 m, 10 Aug 1964, *Sayers NGF-19830* (A, K, L, LAE). **Morobe**: Sumanzing, via Heickepe suppl., 1,829 m, 21 Oct 1938, *M.S. Clemens 9071* (B); Manki ridge road, 8 Jun 1977, *Conn & Kairo 444* (A, K); Mount Shungol, about 5 miles S of Wagau, 1,829 m, 12 Dec 1963, *Hartley 12523 a*, (A, K, L, LAE); Bulolo District, Gumi, above Gumi Village, Lower Watut TRP, 1,950 m, 17 Oct 1992, *Höft 29018* (L); Mount Kandi, Wau, 2200 m, 16 Jul 1978, *Kairo 62* (A, K, L, LAE); Manki, Bulolo, 600 m, 17 Jan 1976, *Kairo*, *A. 70* (A, L, LAE), 18 Jan 1976, *Kairo 73* (A, K, L, LAE), 19 Jan 1976, *Kairo 79* (A, K, L, LAE); Wau, north slope of Mt. Kaindi, 2,050 m, 19 Nov 1983, *Kerenga & Dao LAE-56629* (L, LAE); Mount Buruman, Wantoat, 28 May 1980, *Kerenga LAE-77590* (LAE); Aseki, beside Aseki road, 2,000 m, 27 Jul 1977, *Rau et al. 73* (A, K, L, LAE); Wagau, Morobe Dist., T.N.G., 1,219 m, 5 Jan 1965, *Sayers NGF-21517* (BM, L, LAE); Tawa Village near Aseki, Menyamya Subdistrict, 1,700 m, 16 May 1968, *Streimann & Kairo NGF-27634* (L); Piwi-anga, Menyamya-Kaintiba Road, Menyamya Subdistrict, 1,800 m, 11 May 1968, *Streimann & Kairo NGF-35924* (L); Wau, Aseki road from Bulolo, 2,286 m, 29 May 1977, *Symon & Crutwell 10631* (K, L, LAE, MO, US); Aseki, Aseki road, below crest, 1,950 m, 31 May 1984, *Symon & Katik 13828* (LAE, MO), *Symon & Katik 13830* (L, LAE, MO); Mount Missim, lower slopes, 2 Jun 1984, *Symon & Kairo 13846* (L, LAE, MO); Bulolo District, Gumi, Gumi area, 1,750 m, 3 Jun 1984, *Symon & Vinas 13853* (L, L, LAE), *Symon & Vinas 13854* (L, LAE, MO); near Namie Creek, along Edie Creek road, on flanks of Mount Kaindi, above Wau, 1,585–1,615 m, 8 Sep 1968, *Webster & Hildreth 15188* (GH). **Sanduan**: Vanimo hinterland, Vanimo subdistrict, 500 m, 30 Nov 1971, *Streimann NGF-52968* (K, L, LAE). **Western Highlands**: border with Mandang Province, Bismarck range, summit ridge at Camp 2 (Mt. Oibo), 1,830–2,044 m, 10 Oct 1995, *Takeuchi 10663* (A, L, LAE).

### ﻿11. Lycianthes multifolia

Taxon classificationPlantaeSolanalesSolanaceae

(Merr. & L.M.Perry) A.R. Bean, Austrobaileya 6(3): 567. 2003.

E82C7698-B55D-5D79-A7DE-CC97AA5C9341

Figs 34 , 35

Solanum multifolium Merr. & L.M. Perry, J. Arnold Arb. 30: 50. 1949. Type. Indonesia. Papua: 6 km SW of Bernhard Camp, Idenburg River, 1,150 m, Feb 1939, *L.J. Brass 12907* (holotype: A [00077835]: isotypes: BM [BM000778109] BRI [AQ0022668], L [L0003646], LAE [acc. # 6546, acc. # 229594]).

#### Type.

Based on *Solanummultifolium* Merr. & L.M. Perry

**Figure 34.**
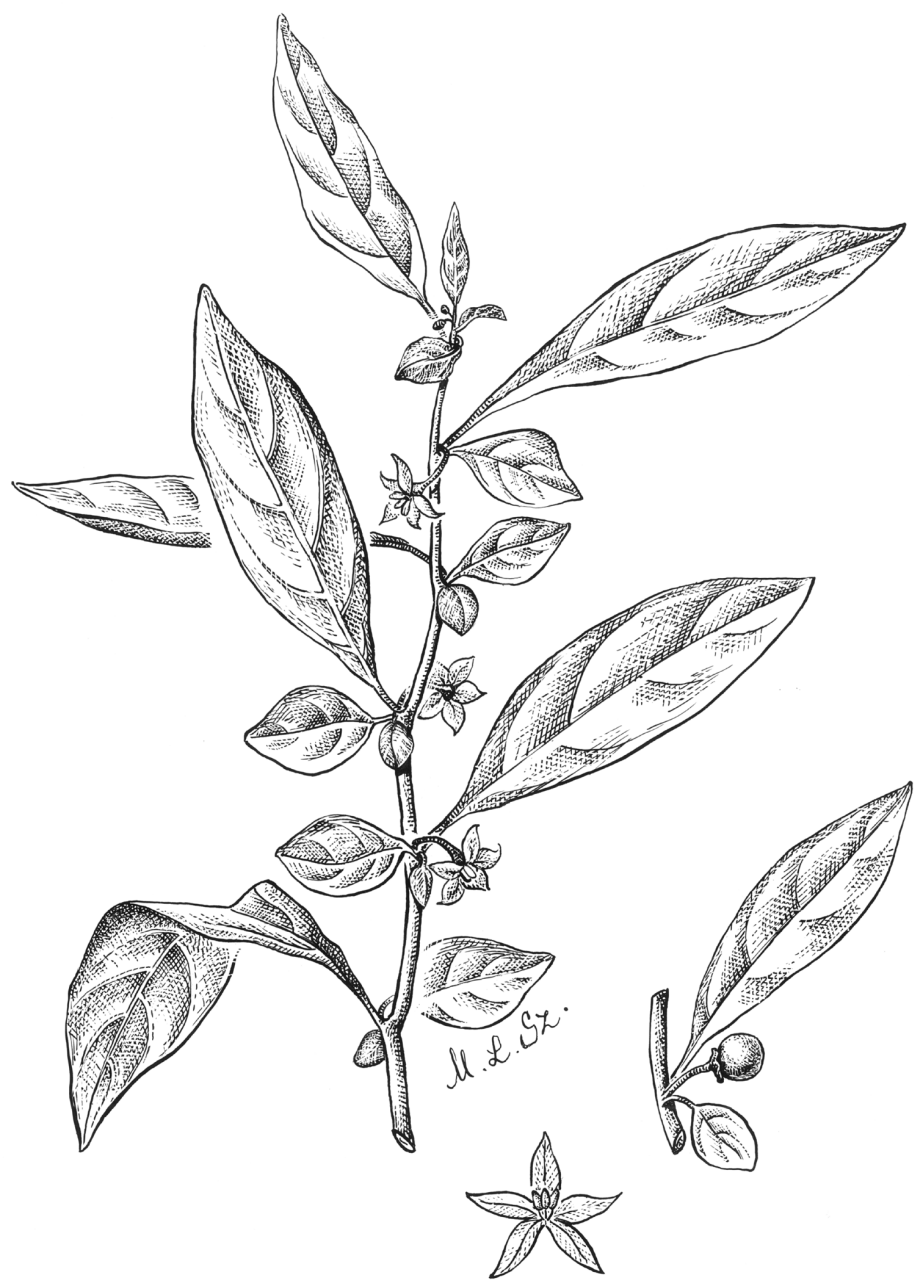
*Lycianthesmultifolia* (Merr. & L.M.Perry) A.R.Bean. Drawing by M.L. Szent-Ivany, first published in Symon (1985: fig. 16, as *S.multifolium* Merr. & L.M.Perry). Courtesy of the Board of the Botanic Gardens and State Herbarium (Adelaide, South Australia), reproduced with permission.

#### Description.

Slender spindly shrubs 0.8–3 m tall, ; stems terete, densely pubescent with stiff, antrorse 1–4-celled simple uniseriate trichomes 0.2–0.7 mm long, these persistent; new growth moderately pubescent with antrorse simple uniseriate trichomes like those of the stems, these denser along the leaf veins; bark of older stems pale tan, not markedly glabrescent. Sympodial units di- or trifoliate, the leaves geminate or with several leaves at a node, the leaves at a node differing in size but not in shape. Leaves simple; blades of major leaves 5.2–9(12) cm long, 1.5–3(5) cm wide, narrowly elliptic or less commonly elliptic, widest at the middle, discolorous, membranous; adaxial surfaces glabrous or with a few antrorse simple uniseriate trichomes along the midrib and scattered stiff 2–3-celled trichomes on the lamina; abaxial surfaces similar; principal veins 6–7 pairs, the midrib keeled above; base acute to attenuate; margins entire; apex acuminate; petiole 0.3–0.6 cm long, sparsely pubescent with trichomes like those of the stems; blades of minor leaves 1–4 cm long, 0.7–2.2 cm wide, similar in shape, texture and pubescence to the major leaves; base attenuate; margins entire; apex acute to acuminate; petiole 0.2–0.5 cm long, sparsely pubescent. Inflorescences axillary fascicles of 3 flowers, only one flower open at a time, pubescent like the stems; pedicels at anthesis 0.7–0.75 cm long, ca. 0.5 mm in diameter at the base, ca. 1 mm in diameter at the apex, slender and nodding, glabrous or with a very few simple uniseriate trichomes near the base, markedly less pubescent than the stems, articulated at the base; pedicel scars tightly packed in the leaf axils. Buds narrowly ellipsoid, the corolla strongly exserted from the calyx tube long before anthesis. Flowers 5-merous, perhaps heterostylous, the one flowering specimen (*Kalkman B.W. 3479*) with all short-styled flowers. Calyx tube ca. 2 mm long, ca. 3 mm wide, open cup-shaped, winged or ridged from the veins, translucent and papery, glabrous except for a few simple uniseriate trichomes ca. 0.2 mm long, with 5 tiny nub-like appendages less than 0.5 mm long arising from very close to or at the rim. Corolla 1–4-1.6 cm in diameter, white, deeply stellate, lobed nearly to the base, interpetalar tissue present, the lobes 6–7 mm long, 1.5–2 mm wide, spreading, membranous, glabrous on both surfaces, but the attenuate tip densely papillate. Stamens equal; filament tube minute; free portion of the filaments 0.75–1 mm long, glabrous; anthers ca. 1 mm long, 1.1–1.25 mm wide, ellipsoid and slightly tapering at the tips, yellow, poricidal at the tips, the pores round, directed distally, not elongating with age. Ovary and style not seen in short-styled flowers. Fruit a globose berry, ca. 0.8 cm in diameter, red when ripe, the pericarp glabrous, thin, shiny, translucent; fruiting pedicels 1.4–1.5 cm long, ca. 0.75 mm in diameter at the base, ca. 1.25 mm in diameter at the apex, not markedly woody, orientation not known; fruiting calyx a spreading saucer at the base of the fruit, not markedly woody or rugose. Seeds 10–20 per berry, 4–4.5 mm long, 2.5–3 mm wide, flattened reniform, reddish brown, the surfaces deeply pitted, the testal cells pentagonal in outline. Stone cells absent. Chromosome number not known.

**Figure 35.**
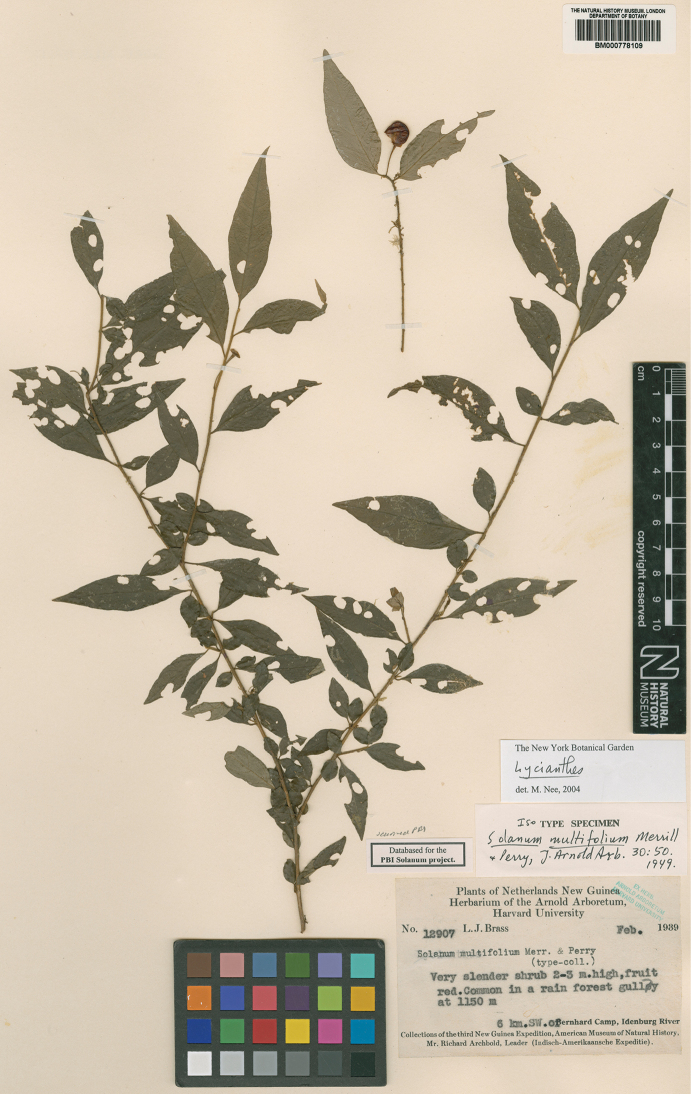
*Lycianthesmultifolia* herbarium specimen. Indonesia. Papua: *Brass 12907* (isotype of *S.multifolium*, BM000778109). Courtesy of the Trustees of the Natural History Museum, London, reproduced with permission.

#### Distribution

**Figure 36.**
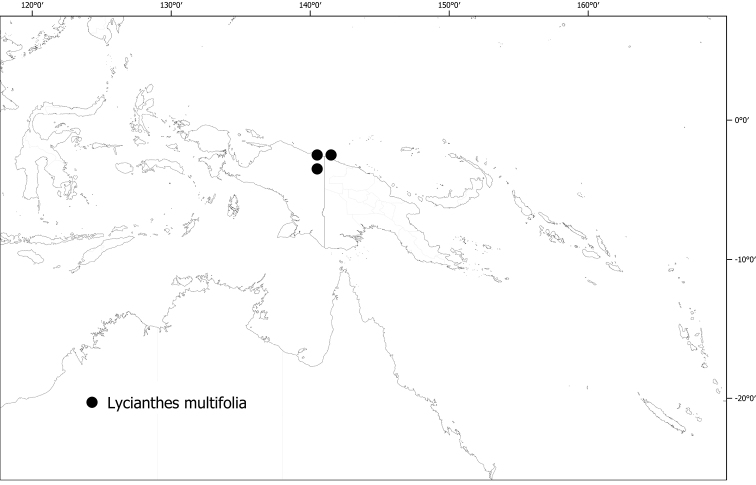
Distribution of *Lycianthesmultifolia*.

**(Fig. 36).***Lycianthesmultifolia* is endemic to the island of New Guinea; found in Papua New Guinea (Sanduan) and Indonesia (Papua).

#### Ecology and habitat.

*Lycianthesmultifolia* grows in rainforests from 50 to 1,150 m elevation.

#### Common names.

None recorded.

#### Preliminary conservation assessment

**(IUCN 2020).**EOO (1,954 km^2^ - EN); AOO (12 km^2^ - EN). *Lycianthesmultifolia* is known from only two widely separated localities; it clearly merits a threat status of Endangered (EN [B1,2a, b(iii, iv)]). The lowland forests where it occurs are within land use concessions (Parsch et al. 2022) in Indonesian Papua.

#### Discussion.

*Lycianthesmultifolia* is similar morphologically to *L.belensis* but has smaller flowers (1.4–1.6 cm versus ca. 2.4 cm in diameter). In addition, the calyx of *L.multifolia* is relatively strongly winged or angled at the veins and has appendages arising from very near the rim, rather than from ca. 0.75 mm below the rim as in *L.belensis*. In general *L.multifolia* is a more delicate plant than many other New Guinea *Lycianthes*. The species name comes from the several leaves at each node seen on the type collection, however, *Kalkman BW-3479* has clearly difoliate geminate sympodial units. It is not clear how consistent leaf number per node is across the range of *L.multifolia* as there are very few collections.

#### Specimens examined.

Indonesia. **Papua**: Res. Hollandia [Jayapura], Nemo, 5 m, 1 Apr 1956, *Kalkman BW-3479* (A, K, L, LAE).

Papua New Guinea. **Sanduan**: near Daunda Bridge, Bewani Highway, subdistrict Vainimo, West Sepik, 120 m, 9 Sep 1977, *Wiakabu & Mamalai LAE-70476* (A, E, K, LAE).

### ﻿12. Lycianthes oliveriana

Taxon classificationPlantaeSolanalesSolanaceae

(Lauterb. & K.Schum.) Bitter, Abh. Naturwiss. Vereins Bremen 24 [preprint]: 504. 1919, as “oliveriana”

1C5C2253-80E2-5217-BCD9-A5D34C6D79DF

Figs 2 , 37 , 38.

Solanum oliverianum Lauterb. & K.Schum., Fl. Schutzgeb. Südsee [Schumann & Lauterbach] 535. 1900 [“1901”], as “Oliverianum”. Type. Papua New Guinea. Sanduan/East Sepik: “Kaiser Wilhelmsland, Augustafluss”, Sep 1887, *M. Hollrung 776* (lectotype, designated by Symon 1985, pg. 56: K [K000759399]; isotypes: HBG [HBG511470], L [L0003651], LE [LE00016994], MEL [MEL104160], P [P00379610]).
Solanum memecylonoides Bitter & Schltr., Bot. Jahrb. Syst. 55: 93. 1917. Type. Papua New Guinea. Sanduan: “Kaiser Wilhelmsland, Torricelli-Geb[irges]”, 800 m, 18 Sep 1909, *F.R.R. Schlechter 20256* (holotype: B [destroyed]; lectotype, designated here: P [P00379576]; isolectotype: BR [BR0000005528844]).
Solanum memecylonoides Bitter & Schltr. var. finisterrae Bitter, Bot. Jahrb. Syst. 55: 94. 1917. Type. Papua New Guinea. Madang: “Kaiser Wilhelmsland, Finisterre-Gebirge”,1,000 m, 3 Jul 1908, *F.R.R. Schlechter 17961* (holotype: B [destroyed]; lectotype, designated here: P [P00379575]; isolectotype: UC [cited by Symon 1985, not seen nor on UC/JEPS database]).
Solanum balanidium Bitter, Bot. Jahrb. Syst. 55: 95. 1917. Type. Papua New Guinea. East Sepik: “Hunsteinspitz” [Mount Hunstein], 1300 m, Feb-Mar 1913, *C.L. Ledermann 11332* (holotype: B [destroyed]). Papua New Guinea. East Sepik: Hunstein range, (Mt. Samsai) at site “Camp 3”on slopes above main streamcourse, 450 m, 17 Jul 1990, *W.N. Takeuchi 6156* (neotype, designated here: LAE [acc. # 293351]; isoneotypes: A [00619947, 00619957], BISH [acc. # 618017], K [K001153745, K000922457, K000922458], L [L.2881432, L.2882048], MO [acc. # 4235181], NSW [NSW825821], NY [01404956, 02286515], US [01253664, acc. # 3723521]).
Solanum ledermannii Bitter, Bot. Jahrb. Syst. 55: 107. 1917, as “Ledermannii”. Type. Papua New Guinea. East Sepik: “Etappenberg” [between Kamelrücken and Bambooberg 142°29E, 4°38S, fide Veldkamp et al. 1988], 850 m, Oct 1912, *C.L. Ledermann 9214* (holotype: B [destroyed]). Papua New Guinea. East Sepik: Amboin, Angoram subdistrict, 90 m, 29 Jul 1967, *A.N. Millar & A.W. Dockrill NGF-35176* (neotype, designated here: LAE [acc. # 89947]; isoneotypes: BRI [n.v.], L [L.2881436]).
Lycianthes balanidium (Bitter) Bitter, Abh. Naturwiss. Vereins Bremen 24 [preprint]: 504. 1919. Type. Based on Solanumbalanidium Bitter.
Lycianthes ledermannii (Bitter) Bitter, Abh. Naturwiss. Vereins Bremen 24 [preprint]: 504. 1919, as “Ledermannii”. Type. Based on Solanumledermannii Bitter.
Lycianthes memecylonoides (Bitter & Schltr.) Bitter, Abh. Naturwiss. Vereins Bremen 24 [preprint]: 504. 1919. Type. Based on Solanummemecylonoides Bitter & Schltr.

#### Type.

Based on *Solanumoliverianum* Lauterb. & K.Schum.

**Figure 37.**
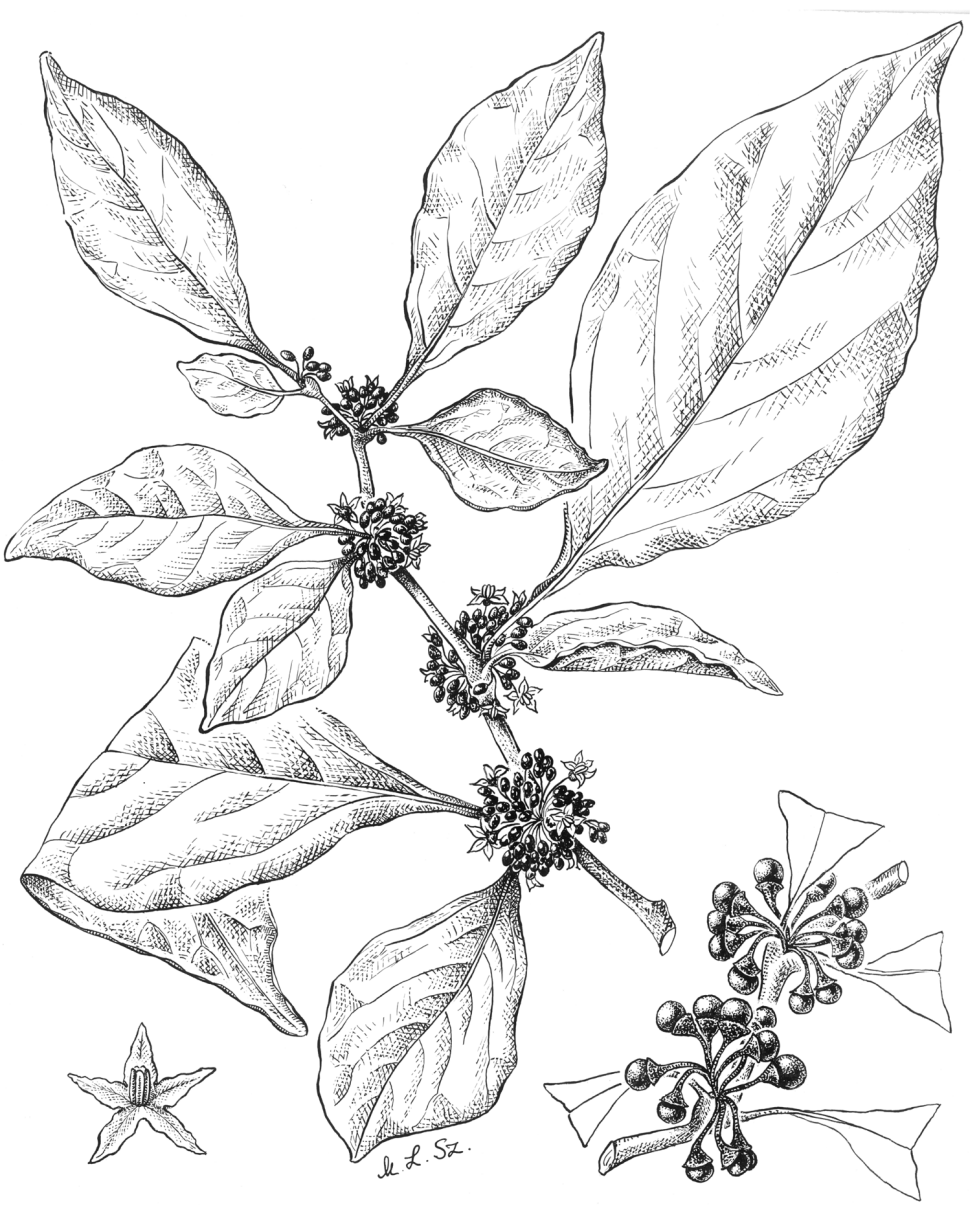
*Lycianthesoliveriana* (Lauterb. & K.Schum.) Bitter. Drawing by M.L. Szent-Ivany, first published in Symon (1985: fig. 17, as *S.oliverianum* Lauterb. & K.Schum.). Courtesy of the Board of the Botanic Gardens and State Herbarium (Adelaide, South Australia), reproduced with permission.

#### Description.

Woody climbers or lianas, sometimes described as shrubs, to 3+ m tall (often described on labels “beautiful” e.g., *van Royen & Sleumer 7716*); stems terete, glabrous; new growth glabrous or minutely papillate with tiny 1–2-celled weak simple uniseriate trichomes less than 0.2 mm long, these soon deciduous; bark of older stems whitish grey, peeling and flaking, somewhat rugose and thick. Sympodial units difoliate, the leaves geminate, the leaves of a pair differing in size but not in shape. Leaves simple; blades of major leaves (6.5)9–25 cm long, (2.8)3–10 cm wide (perhaps larger but not collected), elliptic, slightly discolorous, thick and coriaceous or chartaceous; adaxial surfaces glabrous, somewhat shiny; abaxial surfaces glabrous; principal veins 6–8 pairs, the midrib slightly keeled above, sometimes drying yellowish tan; base acute, often somewhat oblique; margins entire, revolute; apex acute or acuminate with an elongate drip-tip; petioles 1–2.5 cm long, glabrous; blades of minor leaves 4–9 cm long, 2.5–5 cm wide, shape, texture and pubescence like that of the major leaves; base acute; margins entire, revolute; apex acute or acuminate, occasionally rounded; petioles 0.6–1 cm long, glabrous. Inflorescences dense axillary fascicles, occasionally woody and enlarged with what appear to be tiny axes to 0.3 cm long, with 10–20-flowers, several open at the same time, glabrous; pedicels at anthesis 1–1.4 cm long, ca. 0.5 mm in diameter at the base, ca. 1 mm in diameter at the apex, spreading, glabrous, articulated at the base; pedicel scars tightly packed on the woody fascicle base. Buds plumply ellipsoid, the corolla ca. halfway exserted from the calyx tube before anthesis. Flowers 5-merous (4-merous in *Takeuchi 23389*), heterostylous and unisexual, specimens with either all short-styled flowers or long-styled flowers and fruit, the plants probably dioecious. Calyx tube 2.5–3 mm long, 3–3.5 mm in diameter, deeply cup-shaped, usually described as purple or purplish blue, thick and fleshy, densely verrucose/tuberculate, without appendages, the rim somewhat hyaline ca. 0.5 mm wide, sparsely papillate. Corolla 0.8–1.1 cm in diameter, white or purple, stellate, lobed nearly to the base, interpetalar tissue absent, the lobes 2–5 mm long, 1–2 mm wide, spreading or reflexed, thick and fleshy (live plants), appearing woody in dry material, adaxially glabrous to densely papillate with a few weak trichomes distally, abaxially densely papillate somewhat verrucose, the tips and margins densely papillate, the midvein raised especially adaxially, the tips cucullate. Stamens equal; filament tube minute; free portion of the filaments 1–1.5 mm long, glabrous; anthers 2–2.5 mm long, ca. 1 mm wide, plumply ellipsoid or slightly obovoid, creamy white, yellow or purple, poricidal at the tips, the pores round, distally directed, not elongating with age. Ovary conical, glabrous, vestigial in short-styled flowers; styles less than 0.2 mm long and vestigial in short-styled flowers, 5–6 mm long in long-styled flowers, straight, glabrous; stigma slightly bilobed, the surfaces minutely papillate. Fruit a globose berry, 0.7–1 cm in diameter, green and becoming bluish black when ripe, the pericarp glabrous, thick and appearing woody in dry material, matte, opaque; fruiting pedicels 1.1–1.5 cm long, 1–1.5 mm in diameter at the base, 1.5–2 mm in diameter at the apex, spreading or erect (?), woody, corky and markedly verrucose/tuberculate; fruiting calyx a cup surrounding ca. the lower half of the berry (making the fruit look like an acorn), woody (fleshy in live plants) and verrucose/tuberculate both adaxially and abaxially, green flushed with purple (fide *Polak 864*). Seeds 20–40 per berry, 3–3.5 mm long, 2–2.5 mm wide, flattened reniform or slightly tear-drop shape, reddish brown, the surfaces at the margins deeply pitted with pentagonal testal cells, the seed centre only shallowly pitted and the cells not clear. Stone cells absent. Chromosome number not known.

**Figure 38.**
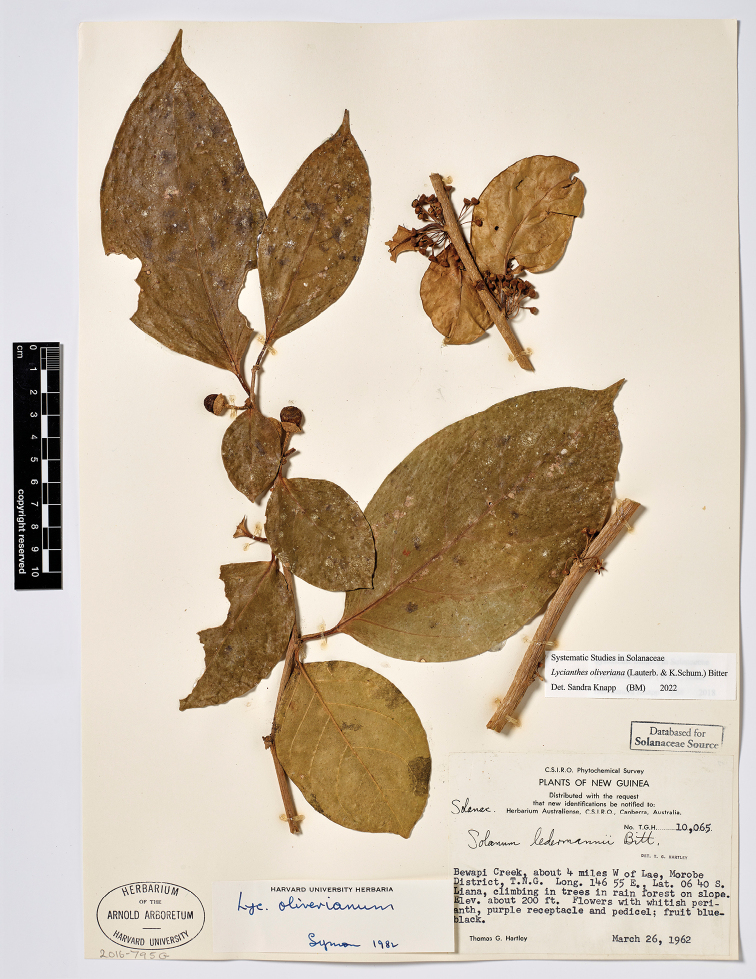
*Lycianthesoliveriana* herbarium specimen. Papua New Guinea. Morobe: *Hartley 10065* (A). Courtesy of the Herbarium of the Arnold Arboretum of Harvard University, reproduced with permission.

#### Distribution

**Figure 39.**
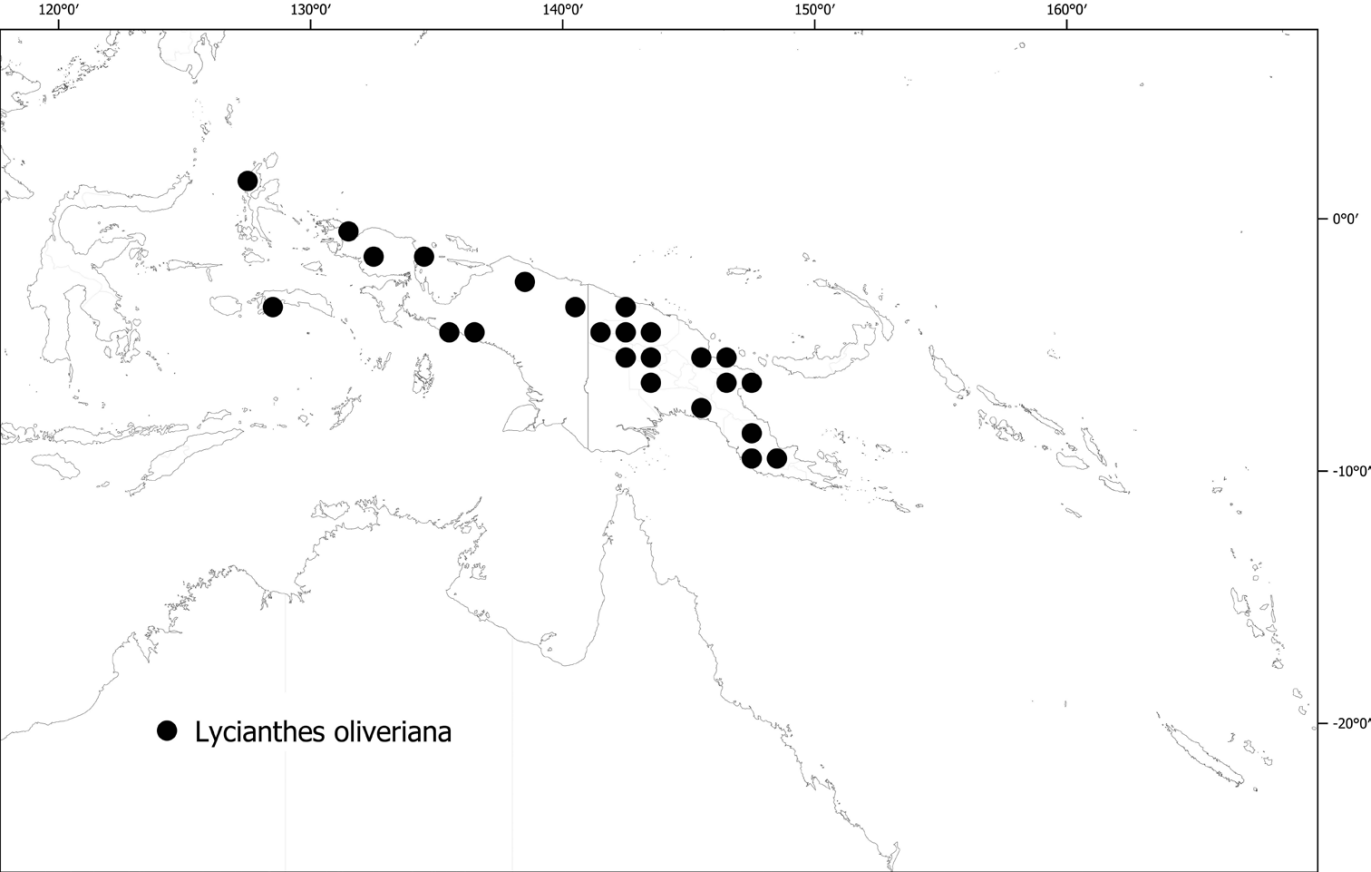
Distribution of *Lycianthesoliveriana*.

(Fig. 39). *Lycianthesoliveriana* is widespread on the island of New Guinea in both Papua New Guinea (Central, East Sepik, Mandang, Morobe, Oro, Southern Highlands, Western) and Indonesia (Papua, Papua Barat); it has also been collected on the nearby Maluku Islands (Seram [Maluku] and Halmahera [Maluku Utara]).

#### Ecology and habitat.

*Lycianthesoliveriana* grows in lowland to montane and premontane rainforests, from almost sea level to 2,300 m elevation. This wide elevational range is accompanied by much variation in leaf size and shape.

#### Common names.

None recorded.

#### Preliminary conservation assessment

**(IUCN 2020).**EOO (1,006,290 km^2^ - LC); AOO (156 km^2^ - EN). *Lycianthesoliveriana* is known from more than 10 localities at a wide variety of elevations. It occurs within protected areas in both Indonesia and Papua New Guinea. This suggests a preliminary threat status of either Least Concern (LC) or Near Threatened (NT).

#### Discussion.

*Lycianthesoliveriana* is, apart from the Pacific *L.vitiensis*, the most commonly collected and widely distributed of the taxa treated in this monograph. It is a large canopy liana that is often described as “beautiful” due to its many flowered inflorescences and large, shiny leaves. Leaf size and shape vary considerably in *L.oliveriana*, plants with smaller, narrower leaves were described as several different taxa (*S.memecylonoides*, S.memecylonoidesvar.finisterrae and *S.balanidium*) by Bitter (1917). Leaf size varies continuously between the extremes, and I cannot discern any environmental factors determining leaf shape and narrowness, although field study or more in-depth environmental analysis might reveal this. All these leaf size variants have the same many-flowered axillary inflorescences, relatively small ca. 1 cm in diameter) flowers with valvate aestivation, thick fleshy corollas, plump, ellipsoid to slightly obovoid anthers and somewhat warty calyces with no appendages.

*Lycianthesoliveriana* is similar to *L.kaernbachii* but differs from it in its glabrous (versus softly pubescent) leaves and strictly axillary (versus cauliflorous) inflorescences. Both species have fleshy calyces without appendages that are often somewhat urceolate and small stellate flowers with fleshy corolla lobes. In low elevation forests *L.oliveriana* broadly co-occurs with *L.impar* that has similar glabrous leaves and difoliate geminate sympodial units. It differs from *L.impar* in having no inflorescence axis, although the woody fascicle can be rather large and lump-like with what are tiny axes, the inflorescence in *L.impar* has a distinct axis with paired pedicel scars along it. Fruits of *L.impar* are imperfectly known, but they appear to be soft and fleshy, while those of *L.oliveriana* have woodier pericarp; this woodiness coupled with the somewhat accrescent calyx tube gives the fruits of *L.oliveriana* the look of tiny acorns (Fig. 38).

Like other species of New Guinea *Lycianthes*, *L.oliveriana* appears to be dioecious, with long- and short-styled flowers on different plants. This needs confirmation in the field, and *L.oliveriana* would be an ideal subject for a reproductive biology study since it is relatively common and widely distributed.

In his discussion of *Solanumbalanidium*, Bitter (1917: 96) suggested that it might be better placed as part of his *S.memecylonoides*, but lack of material held him back. Like *S.memecylonoides*, the collection on which *S.balanidium* is based (*Ledermann 11332*) had narrow leaves and short-styled flowers with no fruit. Almost all of Ledermann’s collections, all of which were distributed from Berlin, have been lost (see Veldkamp et al. 1988; Takeuchi and Golman 2002). I have found no duplicates of Ledermann’s collection from Mount Hunstein, so have selected another gathering (*Takeuchi 6196*) of a short-styled plant from the same mountain range with many duplicates as a neotype (LAE, acc. # 293351).

Bitter (1917) described *Solanumledermannii* from a specimen of short-styled plant with large leaves (“This magnificent species is very similar to *S.Oliverianum*, it differs from it in its more vigorous growth and its larger, coarser, leathery leaves. Unfortunately, the fruits of *S.Ledermannii* are missing from the material, knowledge of which is particularly desirable for comparison with those of *S.Oliverianum*” Bitter 1917: 109, transl. from the original German). The collection was made by C.L. Ledermann (*Ledermann 9214*) during his trip exploring the April River in East Sepik. I have found no duplicates of *Ledermann 9214*; I have therefore selected a collection from East Sepik that matches the protologue and is held in several herbaria as the neotype, although it is from a lower elevation than Ledermann’s collection. The neotype specimen is held at LAE (acc. # 89967) in Papua New Guinea.

#### Specimens examined.

Indonesia. **Maluku**: Seram Island, “Seran, Hatomete”, 4 Dec 1917, *Kornassi 649* (K). **Maluku Utara**: Halmahera, Halmahera, Toliwang, 18 Oct 1951, *Idjan Mochtar 348* (K). **Papua**: “S Nw Guinea” Sg. Aëndosa near Oeta [=Uta], 3 m, 1 Jul 1941, *Aët 395* (A, K, L); Rouffaer River [=Tariku River, Sungai Tariku], 175 m, Aug 1926, *Docters van Leeuwen 9884* (K); Sawia, ‘Nova Guinea neerlandica septemtrionalis’, 100 m, 21 Aug 1911, *Gjellerup 613* (K); Kabupaten Manokwari, Kecamantan Manokwari, Arfak Mountains, Mupi Dessa, trail from Mupi village to G. Humibou, near Sungai Mupi, between Kali Umera (stream) and K. Ngwes, 770 m, 11 Apr 1995, *Sands et al. 6744* (A, K, L); Mount Jaya, PT-Freeport Indonesia Concession Area, new East Levee road about 1 mile from junction of Main Road at Mile 38, 50 m, 9 Apr 1999, *Utteridge et al. 287* (A, K, L); Mount Jaya, PT-Freeport Indonesia Concession Area, Main Road above Mile 38, 230 m, 5 Apr 2000, *Utteridge et al. 295* (A, K, L, LAE, MO). **Papua Barat (West Papua)**: Momi, subdistr. Manokwari, Vogelkop, Momi (S of Manokwari), 18 Aug 1948, *Kostermans 2704* (K); Sorong, near Klamano [Klamano], 20 m, 10 Aug 1948, *Pleyte 623* (A, K); Bird’s Head Peninsula, surroundings of Ayawasi, 14 Jul 1995, *Polak 651* (K); Bird’s Head Peninsula, surroundings of Ayawasi, 500 m, 5 Sep 1995, *Polak 864* (K); Vogelkop Peninsula, Ife River valley, Bamfot village, 850 m, 2 Nov 1961, *Royen & van Sleumer 7621* (K, L, LAE); Vogelkop Peninsula, Ife River valley, central part of Tamray Range, S. slope, path from Sudjak village to Mt. Kusemun, Aiwa River, 840 m, 7 Nov 1961, *Royen & van Sleumer 7716* (K).

Papua New Guinea. “Kaiser Wilhelmsland, am Renegia”, 150 m, 3 Oct 1908, *Schlechter 18319* (P); “Kaiser Wilhelmsland: walden am Renegia”, 150 m, 18 Oct 1908, *Schlechter 18427* (E, LAE, P); Sankwet River, 15 Jun 1977, *Symon 10659* (US). **Central**: Veiya, 12 Mar 1935, *Carr 11670* (BM, K, L, NY); Port Moresby [National Capital District today], *Goldie s.n.* (MEL). **East Sepik**: Waskuk Hills, forest edge near Bangwis stream, 35 m, 2 Jan 2005, *Takeuchi et al. 17743* (A, E, K, LAE, MO, US); Waskuk Hills, along Garuka-Bangwis track, 30 m, 3 Jan 2005, *Takeuchi et al. 17786* (K); Ambunti District, Waskuk Hills, Seringyam near Mt. Musapien bivouac, 360 m, 15 Nov 2007, *Takeuchi & Ama 22112* (A, K, LAE); **Gulf**: Ravikivau, Gulf District, near Ravikivau, Purari delta, 5 m, 18 Feb 1966, *Craven & Schodde848* (K, L, LAE). **Madang**: Mt Wilhelm [border of Chimbu, Jiwaka and Madang], near Plot 1200C, 1,200 m, 6 Nov 2012, *Molino et al. 3060* (LAE, MPU, P); **Morobe**: Sattelberg, 1,006 m, 13 Feb 1936, *Clemens 1821* (A, L); CRA Camp, near Wafi River, east of Tsili Tsili, 200 m, 3 Mar 1985, *Conn et al. 1756* (A, BRI, L, LAE, MO); Bewapi Creek, about 4 miles W of Lae, 60 m, 26 Mar 1962, *Hartley 10065* (A, L, LAE); Burep River NE of Lae, 30 m, 30 Apr 1962, *Hartley 10136* (A, G, K, L, LAE); Tymne-Wago track, 457 m, 18 Mar 1963, *Hartley 11428* (A, K, L, LAE); Oomsis Ridge, 609 m, 22 Feb 1965, *Millar NGF-23858* (A, K, L, LAE); Bewapi Creek, 0.5 mile upstream from main road crossing, subdist. Lae, 7 Jun 1977, *Symon 10655* (K, L, LAE, LAE); above Bupu village on Lae-Bulolo Road, 17 Jun 1984, *Symon 13896* (K); Atzera Range, lowland forest near the 9–11 mile settlement, 300 m, Jan 1994, *Takeuchi 9307* (E, NY, SING). **Oro**: Isuarava, 1,067 m, 4 Mar 1936, *Carr 15948* (B, K); Sibium Mountains, ridge to E of Akupe Camp, Giegari, 1,303 m, 8 Feb 2013, *Damas et al. SAJ-1050* (K, LAE, US); **Sanduan**: Telefomin District, Hak Valley, lower slopes of Deptabip ridge [Sanduan Province], 825 m, 8 Mar 1992, *Frodin et al. 2352* (K); Folongonom, second bush camp below Tamanagabip on track to Busilmin; Telefomin subdist., 2,300 m, 16 May 1975, *Vinas & Wiakabu LAE-59477* (A, E, K, L, LAE). **Southern Highlands**: Deviation Camp (Expedition Bivouac 5), 2,090 m, 8 Apr 2008, *Takeuchi et al. 23895* (A, K, L, LAE, MO, SING, US); Tari subdistrict, Tigibi, 1,570 m, 10 Jun 1966, *Vink 16847* (A, L, LAE). **Western**: Juha North, Bivouac 1, survey track 3, 245 m, 27 Mar 1978, *Takeuchi et al. 23389* (K); Juha North (Bivouac 1) survey track 2, slope immediately east of [coordinates], 350 m, 28 Mar 2008, *Takeuchi et al. 23491* (A). **Western Highlands**: 4 miles from Kopiago on Koroba Rd., Kopiago subdistrict, 1,466 m, 2 Nov 1968, *Womersley et al. NGF-37289* (K, L, LAE).

### ﻿13. Lycianthes peranomala

Taxon classificationPlantaeSolanalesSolanaceae

(Wernham ex. Ridl.) A.R.Bean, Austrobaileya 6(3): 567. 2003.

BD958B41-FA6F-5F05-A23B-09F128B172C3

Figs 40 , 41

Solanum peranomalum Wernham ex Ridl., Hooker’s Icon. Pl. 31 (pt. 3): tab. 3062. 1922. Type. Indonesia. Papua: “Mt. Carstenz [Canoe Camp on Utakwa River drainage]” [Puncak Jaya or Mount Jaya], 45 m, 5 Dec 1912, *C.B. Kloss s.n.* (lectotype, designated by Symon 1985, pg. 58 [as holotype]: BM [BM001014584]).

#### Type.

Based on *Solanumperanomalum* Wernham ex Ridl.

**Figure 40.**
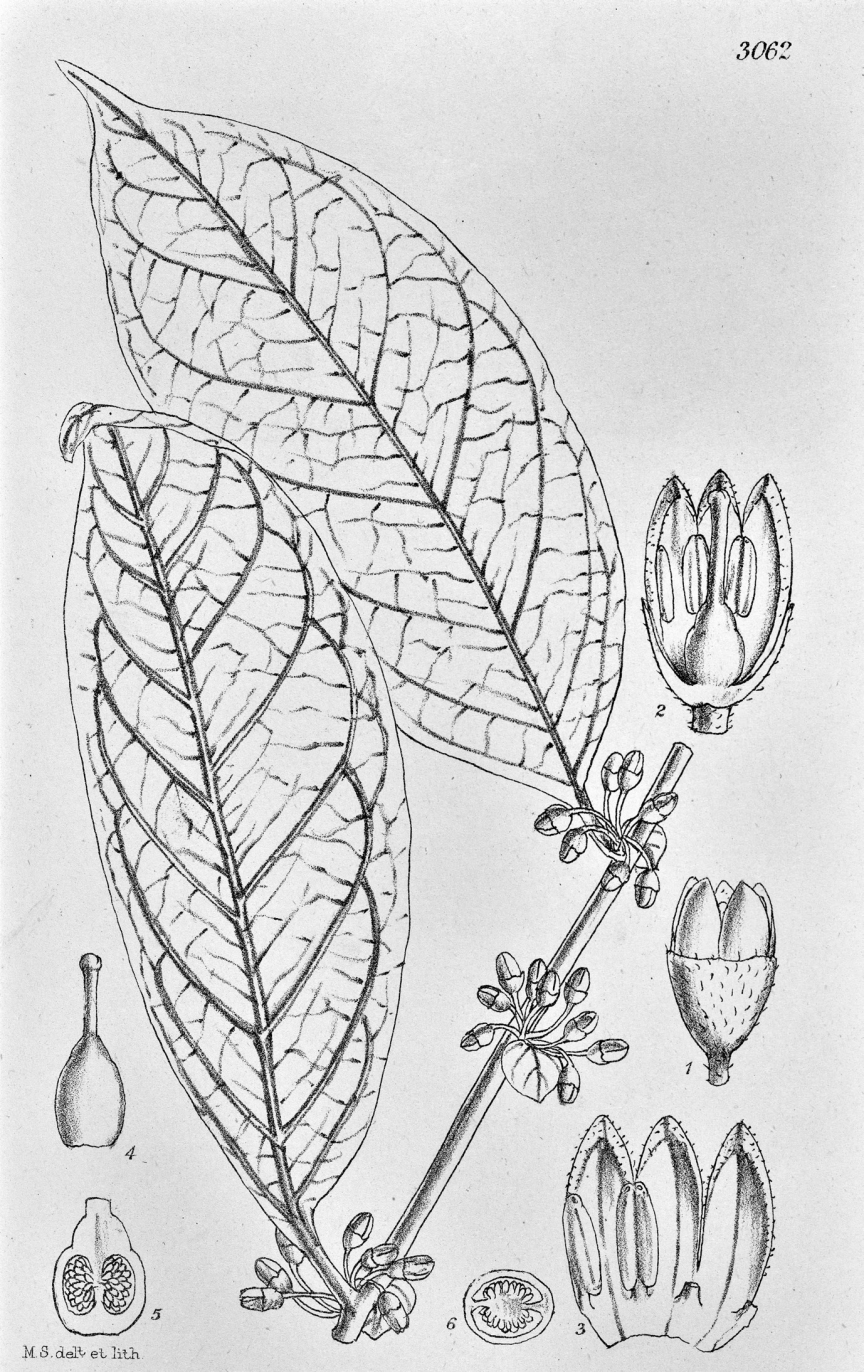
*Lycianthesperanomala* (Wernhamam ex Ridl.) A.R.Bean. Reproduced from Ridley (1922: pl. 3062, as *S.peranomalum* Wernham ex Ridl.). Courtesy of permission of NHM Library and Archives, reproduced with permission.

#### Description.

Shrub to 3 m, or a woody climber with length not recorded; stems terete, sparsely pubescent with appressed stiff antrorse simple uniseriate 1–6-celled trichomes to 1 mm long, the basal cell of each enlarged and sometimes remaining as a pustule or bump; new growth densely stiff-pubescent, the trichomes simple, uniseriate and strongly antrorse; bark of older stems brown, glabrescent, somewhat rugose and corky. Sympodial units unifoliate or difoliate, if difoliate the leaves geminate, the leaves of a pair different in size and sometimes shape. Leaves simple; blades of major leaves 9–16 cm long, 4–7 cm wide, elliptic, widest at the middle, the two sides occasionally uneven in size with the basiscopic half narrower, concolorous or somewhat discolorous, chartaceous or coriaceous; adaxial surfaces bullate (fide *Takeuchi 11204*), glabrous, the midrib keeled; abaxial surfaces glabrous, the veins prominent; principal veins 8–9 pairs, yellowish abaxially; base acute, oblique; margins entire; apex acuminate with an elongate drip-tip; petiole 0.7–1.1 cm long, sparsely pubescent with a few scattered stiff antrorse simple uniseriate trichomes on the adaxial surfaced and near the base; blades of minor leaves 1–2.5(4) cm long, 1–2.2 cm wide, elliptic to orbicular or heart-shaped, often apparently clasping the stem (“retrorsely directed” fide *Takeuchi 11204*), similar in texture and pubescent to the major leaves; base cordate or rounded; margins entire, usually revolute, sometimes markedly so; apex abruptly acute; petioles 0.1–0.2 cm long, glabrous or sometimes with a few simple trichomes like those of the stems. Inflorescences axillary fascicles of 8–10 flowers, several open at once, sparsely pubescent with stiff antrorse trichomes to 0.5 mm long like those of the stems; pedicels at anthesis 0.6–0.7 cm long, ca. 0.5 mm in diameter at the base, ca. 1.5 mm in diameter at the apex, spreading (?), white or pale purple, sparsely pubescent with stiff antrorse simple uniseriate trichomes like those of the stems, ca. 0.5 mm long; pedicel scars clustered in the leaf axils; buds ellipsoid, the corolla ca. halfway exserted from the calyx tube before anthesis. Flowers 5-merous, heterostylous and unisexual, specimens with either short-styled flowers or long-styled flowers and fruit, the plants possibly dioecious. Calyx tube 2.5–3 mm long, 2–2.5 mm wide, urn-shaped, the rim somewhat constricted, thick and woody (dry) or fleshy (live plants?), with no appendages, sparsely pubescent with stiff trichomes like those of the pedicels, these deciduous. Corolla 0.6–0.8 cm in diameter, purple, stellate, lobed nearly to the base, interpetalar tissue absent, the lobes 3–4 mm wide, 1.2–2 mm wide, reflexed, thick and fleshy, adaxially glabrous with a prominent ridged midvein, abaxially glabrous or sparsely papillate, densely papillate on tips and margins. Stamens equal; filament tube minute; free portion of the filaments ca. 1 mm long, glabrous; anthers 1.5–2 mm long, 1–1.5 mm wide, plumply ellipsoid, yellow, poricidal at the tips, the pores distally directed, circular, not elongating to slits with age. Ovary conical, glabrous; style in short-styled flowers ca. 0.5 mm long, in long-styled flowers 4–4.5 mm long, glabrous; stigma capitate or minutely bilobed, the surfaces minutely papillate. Fruit a globose berry, 0.5–0.9 cm in diameter, colour at maturity not known, the pericarp brittle in dry material, glabrous, matte, opaque, fruiting pedicels 0.5–0.8 cm long, ca. 1 mm in diameter at the base, ca. 1.5 mm in diameter at the apex, stiff and somewhat woody and tuberculate, spreading; fruiting calyx a cup at the base of the fruit, covering less than 1/4 of the berry, somewhat tuberculate, with a few stiff trichomes, but these usually deciduous. Seeds 10–20 per berry, ca. 2 mm long, ca. 1.5 mm wide, flattened reniform or slightly tear-shaped, reddish brown, the surfaces deeply pitted especially on the thickened margins, the testal cells pentagonal in outline. Stone cells absent. Chromosome number not known.

**Figure 41.**
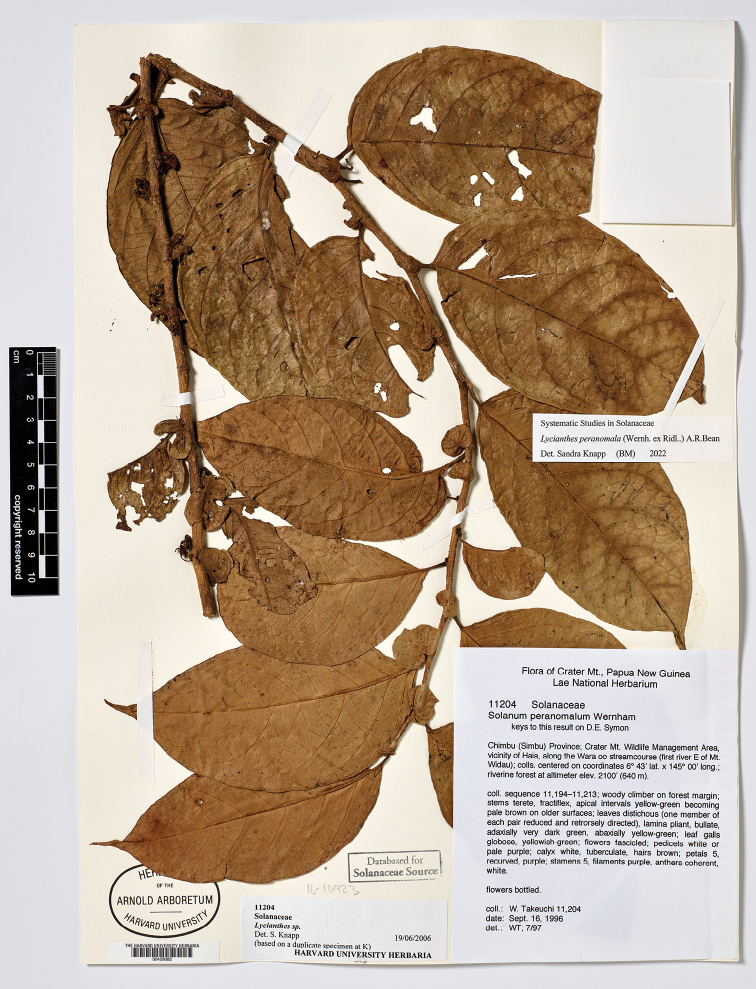
*Lycianthesperanomala* herbarium specimen. Papua New Guinea. Chimbu: *Takeuchi 11204* (A). Courtesy of the Herbarium of the Arnold Arboretum of Harvard University, reproduced with permission.

#### Distribution

**Figure 42.**
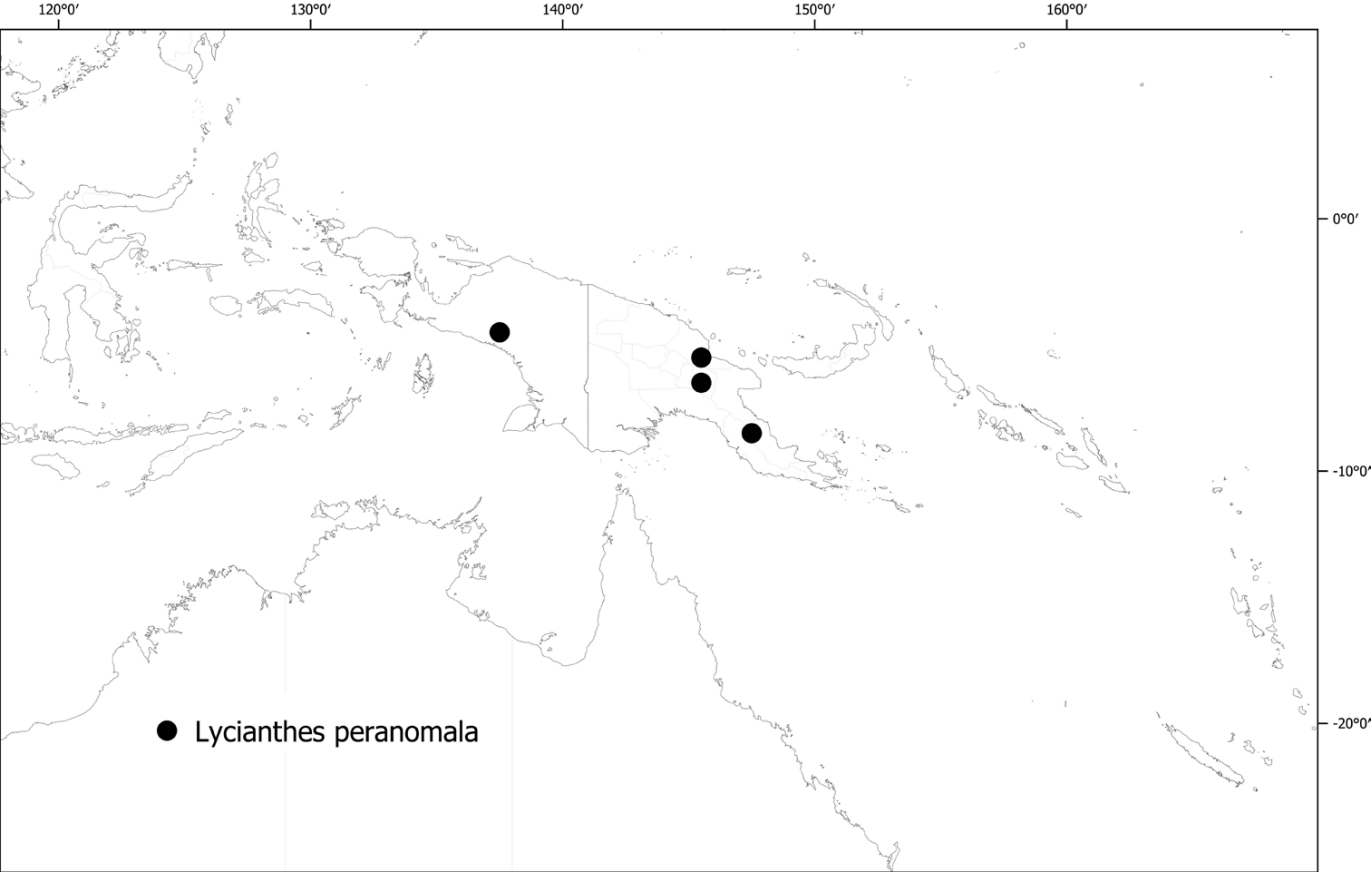
Distribution of *Lycianthesperanomala*.

**(Fig. 42).***Lycianthesperanomala* is endemic to the island of New Guinea; known from Papua New Guinea (Chimbu, Madang, Oro) and Indonesia (Papua).

#### Ecology and habitat.

*Lycianthesperanomala* occurs in lowland rainforest and riverine forests, between 50 and 640 m elevation.

#### Common names.

None recorded.

#### Preliminary conservation assessment

**(IUCN 2020).**EOO (189,160 km^2^ - LC); AOO (16km^2^ - EN). *Lycianthesperanomala* is known from four localities, some of which are within protected areas. Based on EOO alone, it would merit a status of Least Concern, but given the few collections, its forest habitat and general lack of knowledge about the species, I propose a preliminary threat status of Vulnerable (VU [B2a, b(iii,iv)]) for *L.peranomala*.

#### Discussion.

*Lycianthesperanomala* is vegetatively similar to *L.impar* in its orbicular to heart-shaped minor leaves and relatively large, elliptic major leaves with prominent venation. They differ in trichome morphology, *L.peranomala* has sparse stiff, strongly antrorse trichomes on stems, veins near the leaf base and calyces, while those of *L.impar* are soft and curling and found on stems only. Inflorescences of *L.impar* are elongate with paired pedicel scars, while those of *L.peranomala* are strictly axillary.

Like *Lyciantheskaernbachii* and *L.oliveriana* the flowers of *L.peranomala* are usually less than 1 cm in diameter with fleshy corolla lobes lacking any interpetalar tissue and the plants are possibly dioecious with short-styled flowers and long-styled flowers (and fruit) on different plants. *Lycianthesperanomala* can be distinguished from those taxa in its minor leaves that are very dissimilar in shape to the major leaves; both *L.kaernbachii* had *L.oliveriana* have minor leaves that are different in size but not so strongly dissimilar in shape.

The name *Solanumperanomalum* was proposed by Herbert Fuller Wernham, an assistant in the Botany Department of the British Museum, for plants collected by Cecil Boden Kloss on the 1912–1913 expedition led by the ornithologist Arthur Wollaston in a second attempt to reach the high peaks of the “Snow Mountains” (Mount Jaya) in the central range (Ridley 1916). His publication of *S.peranomalum* was superseded by Nicholas Ridley’s publication of the same name, with an illustration (Fig. 40) but a slightly different and less complete description a few months earlier. Ridley perhaps was inpatient for the Transactions to appear or had little regard for Wernham at the Museum, who had a tragic career cut short by mental health issues and alcoholism (Stearn 1981). It is strange that neither of the two other species described by Wernham (1916) were published by Ridley (*S.ridleyanum* and *S.wollastonii*).

#### Specimens examined.

Indonesia. [type only]

Papua New Guinea. **Chimbu**: Crater Mountain Wildlife Management Area, Vicinity of Haia, along the Wara oo streamcourse (first river E of Mt. Widau), 640 m, 16 Sep 1996, *Takeuchi 11204* (A, BM, K, L, LAE, US). **Madang**: Ohu Village, 100 m, 7 Aug 2008, *Ctvrtecka 1644* (US). **Oro**: Kokoda, 365 m, 22 Mar 1936, *Carr 16195* (BM, K, NY).

### ﻿14. Lycianthes rantonnetii

Taxon classificationPlantaeSolanalesSolanaceae

(Carrière) Bitter, Abh. Naturwiss. Vereins Bremen 24 [preprint]: 332. 1919.

1EAE22DC-0C5D-5C69-B739-2F427E2E2A31

Figs 43 , 44

Solanum rantonnetii Carrière, Rev. Hort. [Paris] 32: 135. 1859, as ‘rantonnei’. Type. Cultivated in Paris (lectotype, designated by Dean et al. 2020, pg. 180: [illustration] Carrière, Rev. Hort. [Paris] 32: fig. 32. 1859).
Solanum corniculatum Hiern, Vidensk. Meddel. Naturhist. Foren. Kjøbenhavn 1877–1878: 45. 1877, nom. illeg., not S.corniculatum Huber, 1865. Type. Brazil. Rio de Janeiro: sin. loc., 1867, *A. Glaziou 1078* (lectotype, designated by Dean et al. 2020, pg. 180: C [C10019192]; isolectotypes: BR [BR00000552267, BR00000552234], P [P00325613, P00325614, P00430738]).
Solanum urbanum Morong, Ann. New York Acad. Sci. 7: 177. 1893. Type. Paraguay. Central: streets of Asunción, Nov 1888, *T. Morong 147* (lectotype, designated by Barboza 2013, pg. 29: NY [00172225]; isolectotypes: MO [MO-503602, acc. 2495263], NDG [NDG45160], PH [00030498], US [0027939, acc. # 1324871], WIS [v0004256WIS]).
Solanum muticum N.E.Br., Bull. Misc. Inform. Kew 85: 6. 1894. Type. Uruguay. Montevideo: cultivated in Montevideo, originally from Paraguay, Mar 1858, *E.J. Gibert 56* (lectotype, designated by Barboza 2013, pg. 29: K [K000585755]).
Solanum urbanum Morong var. foliosum Chodat, Bull. Soc. Bot. Genève, ser. 2, 8: 152. 1916. Type. Paraguay. Paraguarí: Paraguary, Cerros de Paraguarí, Sep 1914, *R. Chodat & W. Vischer 60* (lectotype, designated here: G [G00392293]).
Solanum urbanum Morong var. nervosum Chodat, Bull. Soc. Bot. Genève, ser. 2, 8: 152. 1916. Type. Paraguay. Paraguay. Cordillera: “in valle fluminis Y-acá, pr[ope] Valenzuela”, Jan 1900, *É. Hassler 7024* (lectotype, designated by Dean et al. 2020, pg. 180: G [G00390048]; isolectotypes: BM [BM000087583], G [G00392285, G00392288, G00392290], P [P03852955], W [acc. # 1904–804]).
Solanum urbanum Morong var. subtomentosum Chodat, Bull. Soc. Bot. Genève, ser. 2, 8: 152. 1916. Type. Paraguay. Misiones: San Ignacio, Oct 1914, *R. Chodat & W. Vischer 61* (lectotype, designated here: G [G00392295]).

#### Type.

Based on *Solanumrantonnetii* Carrière.

**Figure 43.**
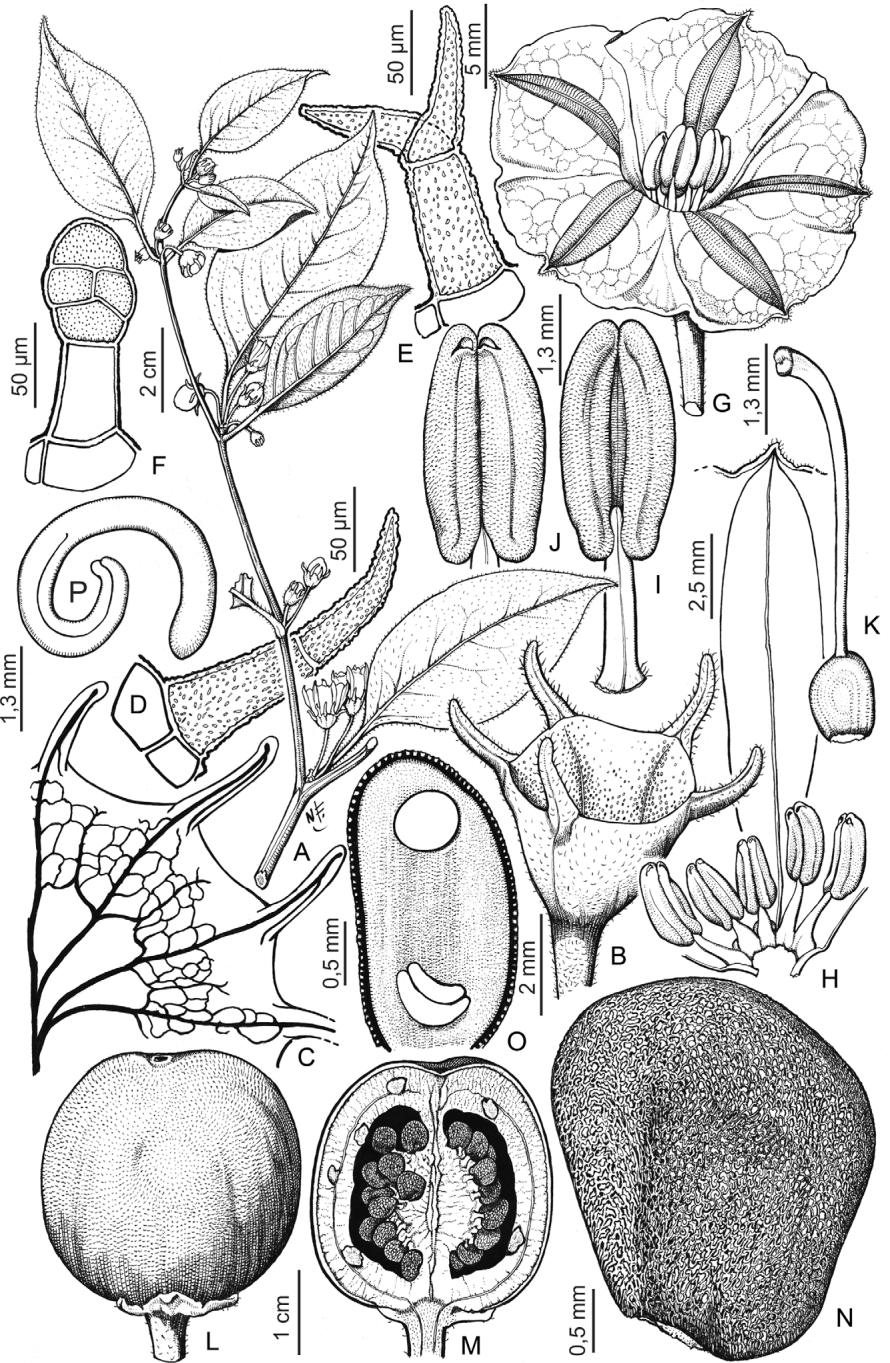
*Lycianthesrantonnetii* (Carrière) Bitter **A** flowering stem **B** five-toothed calyx with appendages near the margins **C** calyx venation **D** simple uniseriate trichome **E** forked trichome **F** glandular papillate trichome **G** rotate corolla with abundant interpetalar tissue **H** androecium showing unequal anther lengths **I** abaxial anther surface **J** adaxial anther surface **K** gynoecium with minutely capitate stigma **L** berry **M** berry cross section showing stone cells embedded in the pericarp **N** seed with minutely pitted surface **O** cross section of seed. Reproduced from Barboza (2013). Courtesy of Flora Argentina, reproduced with permission.

#### Description.

Shrubs 0.5–3 m tall, with multiple stems from the base, these arching and sometimes scandent and sprawling; stems 3–4-angled, the angles yellowish green in live plants and paler than the rest of the stem, sparsely to moderately pubescent with spreading transparent simple uniseriate 1–4-celled trichomes to 0.5 mm long, these occasionally forked or dendritic, glabrescent with age; new growth moderately pubescent with transparent simple uniseriate or occasionally dendritic trichomes like those of the stems; bark of older stems pale greyish brown, prominently angled. Sympodial units unifoliate or more usually difoliate, the leaves usually geminate, if paired the leaves similar in shape and size. Leaves simple; blades of major leaves (1)4–15.5 cm long, (0.5)3.5–7.5 cm wide, ovate, rhombic-elliptic, elliptic or occasionally almost lanceolate, usually broadest at the middle, rarely in the upper half, membranous, concolorous; adaxial surfaces sparsely and evenly pubescent with 1–3-celled simple uniseriate trichomes, these denser along the midrib; abaxial surfaces sparsely to moderately and evenly pubescent with 1–3-celled simple uniseriate trichomes, these denser along the midrib; principal veins 3–7 pairs, more pubescent than the lamina. drying yellowish green abaxially; base attenuate onto the petiole; margins entire or somewhat undulate; apex acute to acuminate; petiole 0.5–2.4(4) cm long, winged from the attenuate leaf base, pubescent with simple uniseriate (or occasionally dendritic) trichomes like those of the stems and leaves; blades of minor leaves similar in size and shape to those of the major leaves, or slightly smaller; petioles 0.5–3 cm long, winged. Inflorescences axillary fascicles with (1)2–7 flowers, pubescent with transparent trichomes like those of the new growth and stems; pedicels 1.2–1.7 cm long, ca. 1 mm in diameter at the base, ca. 2 mm in diameter at the apex, spreading at anthesis, sparsely to moderately and evenly pubescent with transparent simple (occasionally dendritic) uniseriate 1–3-celled trichomes like those of the stems, articulated at the base; pedicels scars tightly packed in the leaf axils. Buds ellipsoid to fusiform with pointed tips, the corolla more than halfway exserted from the calyx tube before anthesis. Flowers 5-merous, all apparently perfect. Calyx with the tube 1.5–4 mm long, 2.5–4.5 mm wide, openly cup-shaped, with (5)10 linear subulate appendages of variable length 0.25–5.2 mm long, arising ca. 0.25–1 mm from the hyaline rim, usually alternating long and short, sparsely to moderately pubescent with simple trichomes like those of the pedicels. Corolla 1.2–2 cm in diameter, violet with the midveins dark purple and the centre yellow, rotate, lobed less than 1/10 of the way to the base, interpetalar tissue abundantly present, the lobes ca. 1 mm long, ca. 1 mm wide and mere acumens from the rotate corolla, glabrous on both surfaces except for the densely papillate, cucullate tips (acumens). Stamens unequal; filament tube minute; free portion of the filaments of two lengths, three long filaments 2–3 mm long, 2 short filaments 0.8–1.5 mm long, glabrous or adaxially pubescent with tangled weak-walled uniseriate simple trichomes; anthers ellipsoid and slightly curved, orange-yellow, poricidal at the tips, the pores round, distally directed, not elongating to slits with age. Ovary conical, glabrous; style 3.5–5.5 mm long, slightly curved in the same direction as the anthers, glabrous; stigma slightly clavate and bilobed, the surface minutely papillate. Fruit a compressed-ellipsoid or compressed globose berry, 2–3 cm long, 1.3–1.5 cm in diameter (usually absent or smaller and seedless in cultivated plants), yellow or yellowish orange when mature, the pericarp glabrous, thin, shiny and translucent; fruiting pedicels 2.5–4 cm long, ca. 1.5 mm in diameter at the base, ca. 3 mm in diameter at the apex, somewhat woody, spreading or hanging from the weight of the berries; fruiting calyx a plate with the appendages somewhat longer than in flower, spreading and often broken off, stiff and woody. Seeds 20–100 per berry (many fewer in cultivated plants), 2–3.5 mm long, 1.5–3.5 mm wide, rounded and compressed, reddish tan, the surfaces minutely pitted, the testal cells with sinuate margins. Stone cells more than 20 per berry, ca. 0.5–1.5 mm in diameter. Chromosome number: 2n=24 (Gerasimenko and Reznikova 1968 [cited in D’Arcy 1974] as *Solanumrantonnetii*; Acosta et al. 2005, as *L.rantonnei*, voucher *Moscone 4260* [CORD]).

**Figure 44.**
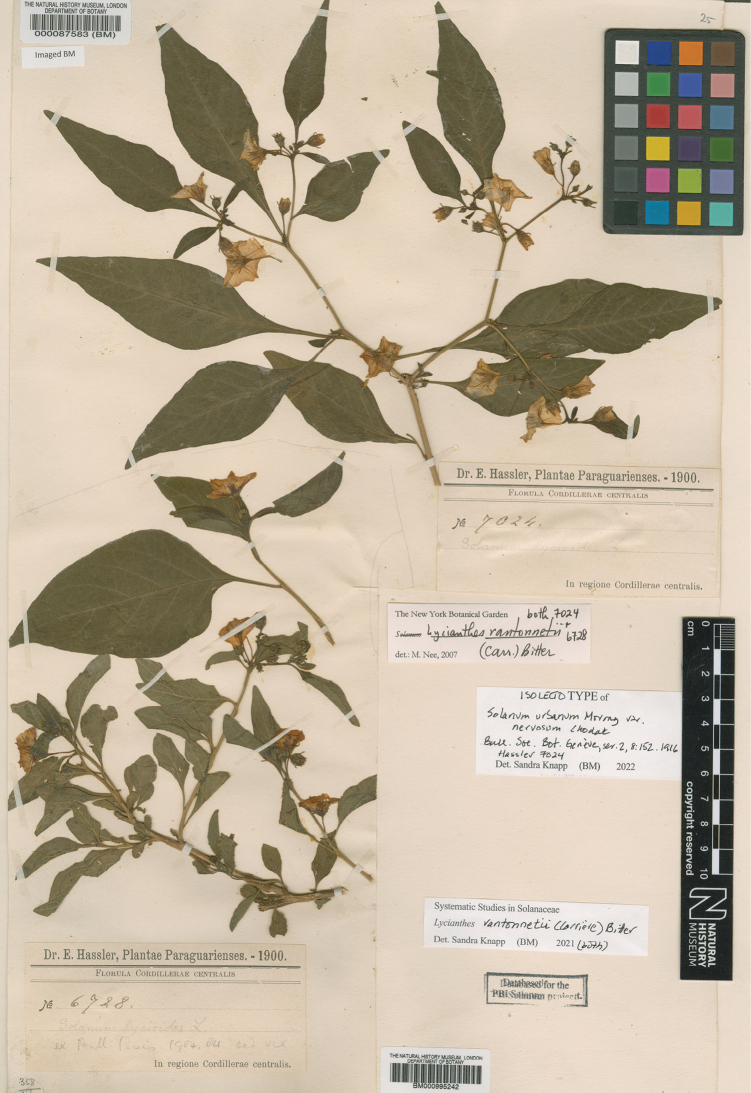
*Lycianthesrantonnetii* herbarium specimen. Paraguay. Cordillera: *Hassler 7024* (isolectotype of S.urbanumvar.nervosum Chodat, BM000087583) and *Hassler 6728* (BM000995242). Courtesy of the Trustees of the Natural History Museum, London, reproduced with permission.

#### Distribution.

*Lycianthesrantonnetii* is widely cultivated in the tropics and subtropics (and even into the temperate zone as a short-lived perennial) worldwide. I have seen no collections from New Guinea, but it has been relatively widely collected in Australia. It is native to southern South America (Argentina, Bolivia, southern Brazil and Paraguay).

#### Ecology and habitat.

In its native range *L.rantonnetii* is a plant of semi-moist, seasonal forests and open areas; from (sea level) 100 to 2,000 m elevation.

#### Common names.

In its native range in Argentina *L.rantonnetii* is called meloncillo del aire (Barboza 2013).

#### Preliminary conservation assessment

**(IUCN 2020).** Not applicable to this species for this region.

#### Discussion.

*Lycianthesrantonnetii* is native to South America (Barboza 2013) but widely cultivated in subtropical and temperate areas worldwide. It is the only species occurring in the region treated here that has stone cells in the berries, but in cultivation it rarely sets fruit. It can easily be distinguished from all native species by its rotate corollas with copious interpetalar tissue, orange-yellow anthers that are slightly curved and angled, and somewhat striped stems. In the area treated here I have only seen specimens of *L.rantonnetii* from Australia, but I would expect it to be in cultivation anywhere in the region.

This species epithet is often seen spelled “rantonnei” but is correctable to “rantonnetii” following Art. 60.9 of the ICN (Turland et al. 2018: Ex. 31), which stipulates that epithets honouring persons where there is an intentional latinisation of the name that involves the omission of a terminal vowel or consonant are not permitted; the epithet in this case honours the French horticulturalist Barthélémy Victor Rantonnet so is correctable to “rantonnetii” even though Carrière (1859) originally spelled in “rantonnei” (using the latinisation Rantonneus).

Dean et al. (2020) cited the type specimens of Solanumurbanumvar.foliosum and var. subtomentosum as “holotype”, but Chodat (1916) did not cite a specific herbarium in any part of the protologue thus these names require lectotypification (see McNeill 2014), even though only a single specimen is preserved in the herbarium at G. I have selected the specimens cited as holotypes by Dean et al. (2020) as the lectotypes in both these cases.

#### Specimens examined.

Australia. **New South Wales**: North Coast, 37.5 km from the Bruxner Highway along Long Gully Road, next to Rocky River., 3 Feb 2014, *Johnstone 3435* (NSW). **Queensland**: Wide Bay, cultivated at Nora Creina, Homestead, 1.5km north-northeast of Didcot., 24 Jan 1999, *Forster 24067* (BRI); Moreton, Brisbane Mt. Coot-Tha Botanic Gardens, 1 Mar 1984, *Whitten s.n.* (BRI). **South Australia**: in creek near Fremont Park, Elizabeth, 10 Feb 1988, *Bates 13861* (AD); Rocky River S of Laura, 12 Jan 1993, *Bates 30884* (AD); in creek NW below entrance to Angove C[onservation] P[ark], (dedicated 2 weeks ago), 15 Jul 1994, *Bates 37051* (AD); Naracoorte, Jul 1940, *Cooper s.n.* (AD); Region 13, Southeastern Drain L., Oct 1989, *Lawson 51* (AD, CANB, MEL, MO); On the roadside at Winding Way, Belair, by vacant block, 18 Feb 1976, *Sparrow s.n.* (AD); Tusmore, Kenneway St. (rear of No. 20 Lynington St.), 14 Dec 1974, *Symon s.n.* (CANB). **Western Australia**: Forrestfield, 1948, *Dawson Harrison Ltd s.n.* (PERTH); Mingenew tip, 30 Apr 1997, *Elson s.n.* (PERTH).

### ﻿15. Lycianthes rostellata

Taxon classificationPlantaeSolanalesSolanaceae

(Merr. & L.M. Perry) A.R.Bean, Austrobaileya 6(3): 568. 2003.

E7E4E523-7600-5D9E-B0EF-6BB853C12170

Figs 45 , 46

Solanum rostellatum Merr. & L.M.Perry, J. Arnold Arb. 30: 51. 1949. Type. Papua New Guinea. Central: East Mt. Tafa, Central Division, 2,100 m, May 1933, *L.J. Brass 4135* (holotype: A [00077837]; isotypes: BRI [BRI-AQ0080376], L [L0003660], LAE [acc. # 229583], NY [00172292]).
Solanum pustulatum Symon, J. Adelaide Bot. Gard. 8: 58. 1985. Type. Papua New Guinea. Eastern Highlands: confluence of Warapuri and Warranaga Rivers, Wahgi-Jimi Divide north of Nondugl, Minj subdistrict, 2,134 m, 5 Sep 1963, *P. van Royen NGF-18229* (holotype: BRI [AQ0080260]; isotypes: CANB [CANB135300], K [K000922492], L [L0403779], LAE [acc. # 58346]).
Lycianthes pustulata (Symon) A.R.Bean, Austrobaileya 6(3): 568. 2003. Type. Based on Solanumpustulatum Symon.

#### Type.

Based on *Solanumrostellatum* Merr. & L.M.Perry.

**Figure 45.**
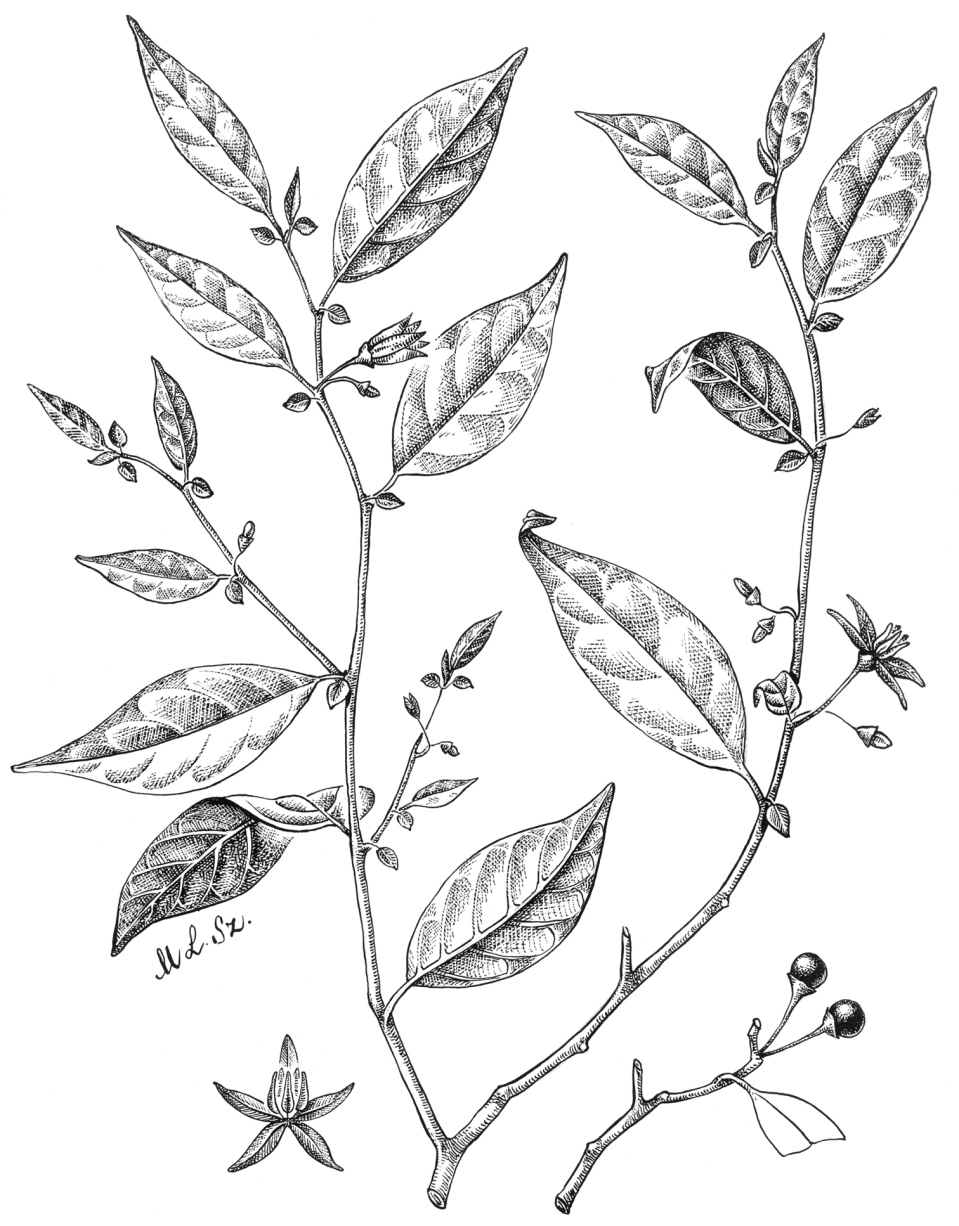
*Lycianthesrostellata* (Merr. & L.M.Perry) A.R.Bean. Drawing by M.L. Szent-Ivany, first published in Symon (1985: fig. 19, as *S.pustulatum* Symon). Courtesy of the Board of the Botanic Gardens and State Herbarium (Adelaide, South Australia), reproduced with permission.

#### Description.

Shrubs or woody vines, 0.3–3 m tall (long); stems terete, densely pubescent with stiff, antrorse simple 4–5-celled uniseriate trichomes to 0.5 mm long, these sometimes from multicellular bases or the bases becoming enlarged with plant growth(i.e., remnants of multicellular bases on old stems but young stems without); new growth sparsely to densely pubescent with stiff simple uniseriate trichomes like those of the stems; bark of older stems brownish grey, glabrescent, corky and warty with persistent multicellular trichome bases. Sympodial units unifoliate (with deciduous minor leaves) or difoliate, the leaves geminate, the leaves of a pair different in size and shape. Leaves simple; blades of major leaves 7–12(17) cm long, 1–5 cm wide, elliptic or more commonly narrowly elliptic, widest at the middle, lower leaves on stems broader than distal ones, discolorous, thick and chartaceous (papery fide *Takeuchi 11804*), slightly bullate; adaxial surfaces shiny, glabrous, the veins deeply impressed; abaxial surfaces glabrous or with a few antrorse simple uniseriate trichomes like those of the stems along the midrib, sometimes purple-tinged (fide *Womersley 4883*); principal veins 4–6 pairs, impressed above, prominent below and occasionally sparsely pubescent; base acute; margins entire, revolute; apex abruptly attenuate to a long drip-tip; petioles 0.3–1.2 cm long, sparsely pubescent with antrorse simple uniseriate trichomes to ca. 0.5 mm long like those of the stems; blades of minor leaves 0.3–0.7 cm long, 0.3–0.7 cm wide, orbicular or somewhat heart-shaped, texture and pubescence like that of the major leaves, often deciduous especially on reproductive stems; base truncate or cordate; margins entire, revolute; apex rounded; petioles less than 0.1 cm long, sparsely pubescent. Inflorescences axillary fascicles of 2–4 flowers, usually only a single flower open at a time, densely pubescent like the stems with stiff antrorse simple uniseriate trichomes; pedicels at anthesis 0.8–1 cm long, ca. 0.5 mm in diameter at the base, 1–1.5 mm in diameter at the apex, erect (?) or spreading, sparsely pubescent with stiff antrorse simple trichomes like those of the stems, articulated at the base; pedicel scars tightly packed in the leaf axils. Buds long-ellipsoid, the corolla strongly exserted from the calyx tube just before anthesis, included in early buds. Flowers 5-merous (6-merous in *Womersley 4883*), apparently all perfect, heterostyly not observed. Calyx tube 2.5–3 mm long, 3–3.5 mm wide, cup-shaped, woody and stiff in dry material, perhaps fleshy in live plants, white or cream-colored, with no appendages, sparsely pubescent with stiff antrorse simple uniseriate trichomes ca. 0.5 mm long. Corolla 1.4–2 cm in diameter, white tinged with purple, purplish blue or purple, deeply stellate, lobed nearly to the base, interpetalar tissue absent, the lobes 8–10 mm long, 1.5–2 mm wide, reflexed or spreading, membranous/fleshy, glabrous on both surfaces, with minutely papillate tips and margins. Stamens equal; filament tube minute; free portion of the filaments 0.75–1 mm long, glabrous or with a few stiff simple uniseriate trichomes abaxially; anthers 5–6 mm long, ca. 1 mm wide, ellipsoid and somewhat tapering at the tips, yellow, poricidal at the tips, the pores round, distally directed, not elongating to slits with age. Ovary conical, glabrous; style 5.5–6 mm long, straight, glabrous (white fide *Takeuchi 11804*); stigma clavate, the surfaces minutely papillate. Fruit a globose berry, often apically umbonate, 0.7–0.8 cm in diameter, green or dark green (immature?), ripening purple-black, the pericarp thick and somewhat woody (fruits immature?), matte, opaque; fruiting pedicels 1.8–2.1 cm long, ca. 1 mm in diameter at the base, ca. 2 mm in diameter at the apex, erect or spreading, somewhat woody, the surfaces thickened and rugose; fruiting calyx a stiff, woody plate beneath the fruit, not enclosing the base. Seeds 60–80 per berry, 2–2.5 mm long, 1.5–2 mm wide, flattened reniform with a deep notch, yellowish golden, the surfaces deeply pitted, especially at the thickened margins, the testal cells pentagonal in outline with slightly sinuate walls. Stone cells absent. Chromosome number not known.

**Figure 46.**
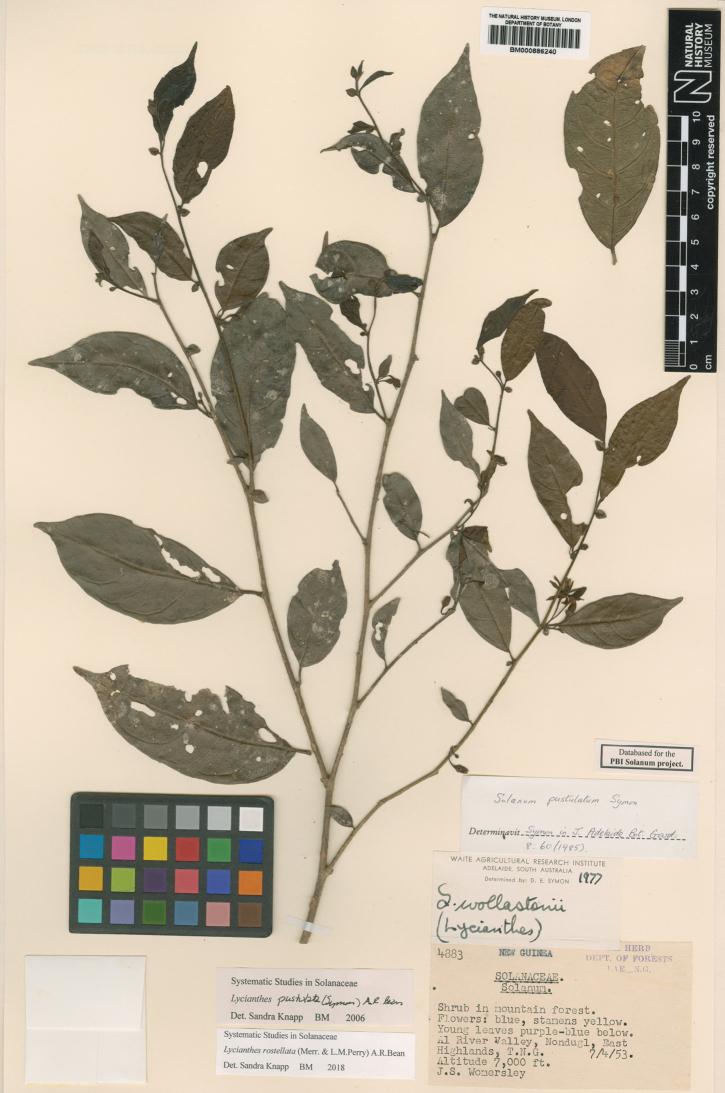
*Lycianthesrostellata* herbarium specimen. Papua New Guinea. Eastern Highlands: *Womersley 4883* (BM000886240). Courtesy of the Trustees of the Natural History Museum, London, reproduced with permission.

#### Distribution

**Figure 47.**
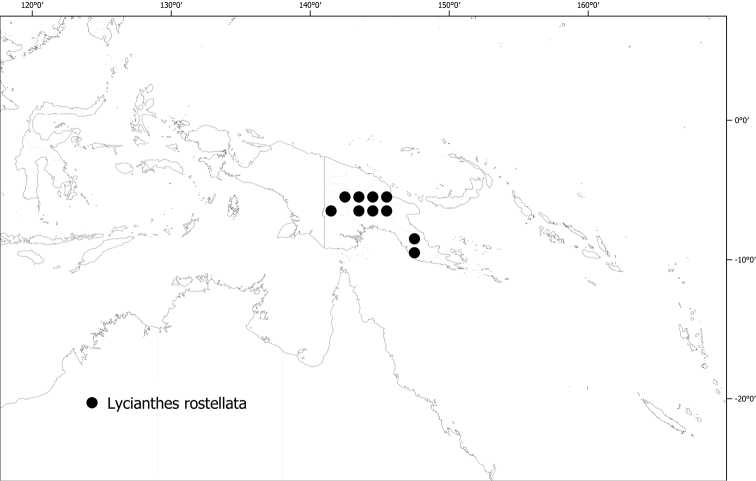
Distribution of *Lycianthesrostellata*.

**(Fig. 47).***Lycianthesrostellata* is endemic to the island of New Guinea and is relatively widely distributed; it has only been collected in Papua New Guinea (Central, Eastern Highlands, Enga, Oro, Western Highlands).

#### Ecology and habitat.

*Lycianthesrostellata* grows in montane and submontane forests, as well as riverine regrowth forests, from 740–2,500 m elevation.

#### Common names.

Papua New Guinea: baga (*Henty et al. NGF-41640*)

#### Preliminary conservation assessment

**(IUCN 2020).**EOO (94,873 km^2^ - LC); AOO (84 km^2^ - EN). *Lycianthesrostellata* is known from more than 10 localities, some of which are within protected areas (e.g., Crater Mountain Wildlife Area). It is currently a single-country endemic. This suggests a preliminary threat status of either Least Concern (LC) or Near Threatened (NT).

#### Discussion.

*Lycianthesrostellata* is one of the larger-flowered species of *Lycianthes* on New Guinea. Like *L.bambusarum*, *L.belensis* and *L.moszkowskii* the corolla is ca. 2 cm in diameter; it can be distinguished from *L.bambusarum* by its pubescent rather than glabrous or minutely papillate new growth, and from *L.belensis* and *L.moszkowskii* by its stiff antrorse pubescence of trichomes with multicellular bases. *Lycianthesbelensis* has soft, curled trichomes on the new growth, and *L.moszkowskii* has antrorse pubescence, but it is sparser than that of *L.rostellata* and the trichomes never have multicellular bases. Flower buds of *L.rostellata* are long-ellipsoid and pointed at the tips (the character upon which the specific epithet is based). *Lycianthesshanesii* of Australia has similar pointed buds, but the corolla lobes are wider relative to their length (2–3 times longer than wide) that are those of *L.rostellata* (4–5 times longer than wide) and the mature berries of *L.shanesii* are bright red, while those of *L.rostellata* are purple-black.

Plants named as *Solanumpustulatum* (Symon 1985) represent an extreme in trichome morphology with prominent multicellular bases that persist after the uniseriate part of the trichome breaks off, but there is much variation in this character, from no to large multicellular bases as well as in the density of these types of trichomes. The specimen illustrated by Symon (1985: fig. 22,) has unusually wide leaves and persistent minor leaves, most collections of *Lycianthesrostellata* as circumscribed here have narrower, lance-elliptic leaves and the minor leaves are often deciduous with the sympodial units apparently unifoliate. Plants not in flower also appear to have wider leaves (*Symon & Katik 10690*, *10677)*, so there may be some change in leaf shape as stems reach reproductive phase (e.g., Roe 1966).

#### Specimens examined.

Papua New Guinea. **Central**: Boridi, 1,524 m, 3 Oct 1935, *Carr 14357* (BM, K, NY); subdsitrict Port Moresby, east slope of Lake Myola No. 1, 1,920 m, 17 Sep 1973, *Croft & Lelean NGF-34800* (E, K, L, LAE, MO, QRS, US); Efogi environs, Owen Stanley range,, 1830 m, 14 Sep 1970, *Schodde 5705* (A, K, L, LAE); Murray Pass, near the summit, 2,650 m, 13 Jun 1984, *Symon 13874* (K, L, LAE, MO); Murray Pass, near crest of pass, 2,800 m, 13 Jun 1984, *Symon 13875* (K, L, LAE, MO); Murray Pass, “Mur Mur Pass”, 2,700 m, 13 Jun 1984, *Symon 13876* (K, L, LAE), *Symon 13877* (K, L, LAE, MO). **Eastern Highlands**: above Fatima River, Marafuga, Sub-dist. Goroka, 2,100 m, 13 Nov 1968, *Millar NGF-40737* (K, L, LAE); Daulo Pass, top of Daulo Pass, 2,320 m, 22 Jun 1977, *Symon & Katik 10677* (K, L, LAE); near summit of Daulo, 22 Jun 1977, *Symon 10678* (MO, US); Crater Mountain Wildlife Area, E of Haia village along Wara Sename, Chimbu (Simbu) province, Crater, 740 m, 16 Mar 1997, *Takeuchi 11804* (K, L, LAE, US); Nondugl, Al River Valley, 2,134 m, 7 Apr 1953, *Womersley 4883* (A, BM, K, L, LAE). **Enga**: between Mount Hagen and Wabog, 42 km from Mount Hagen, 29 km after turnoff to Wabog, 13 km before Waterfall Village, 2,520 m, 23 Jun 1977, *Symon 10687* (K, L, LAE, MO); Wahgi-Sepik Divide, from Banz to Tabibunga, 4 km after crest and 39 km before Tabibunga, 27 Jun 1977, *Symon 10704* (K, L, LAE, MO). **Oro**: Alola, 1,829 m, 4 Dec 1935, *Carr 13611* (BM, K, NY), 1,981 m, 11 Dec 1933, *Carr 13737* (K), 11 Dec 1935, *Carr 13738* (BM, K, NY); eastern side lake Myola No.1 (Northern District), Kokoda subdistrict, 2,000 m, 23 Jul 1974, *Croft et al. LAE-61993* (A, K, LAE). **Sanduan**: Bulindip, W of Oksapmin, Telefomin subdistrict, 1,981 m, 19 Oct 1968, *Henty et al. NGF-41640* (A, K, L, LAE). **Southern Highlands**: Onim Hill, Mt. Gilwe Timber area, Mendi subdistrict, 2,500 m, 19 May 1975, *Argent 8/ 19* (K); Mount Giluwe, track from Onim to SW summit [Eastern Highlands on label], 2,290 m, 19 Jul 1976, *van Royen 11511* (K, L, LAE); between Nol and Mendi, 24 km from Mendi [georef to Mendi], 24 Jun 1977, *Symon & Katik 10690* (K, L, LAE, MO). **Western Highlands**: Wapalepa, Kepaka, Upper Kaugel Valley, Hagen [Mt Hagen?], 2,652 m, 13 Jul 1969, *Bowers 796* (L, LAE, US); Waghi & Jim Divide, Minz subprovince, 10 Aug 1981, *Kerenga & Croft LAE-77644* (K, L, LAE); Kundip, Mt. Hagen subdistrict, 2,134 m, 10 Sep 1963, *Millar & Garay NGF-18664* (GH, LAE); Wabag Road, 1.5 miles from turn-off, Mount Hagen subdistrict, 2,469 m, 30 Sep 1968, *Vandenberg et al. NGF-39883* (A, K, L, LAE).

### ﻿16. Lycianthes shanesii

Taxon classificationPlantaeSolanalesSolanaceae

(F.Muell.) A.R.Bean, Austrobaileya 6(3): 568. 2003.

91E00661-E20C-5B24-B44F-D7E1D2F552FE

Figs 48 , 49

Solanum shanesii F.Muell., Fragm. 6: 144. 1868. Type. Australia. Queensland: Rockhampton, 25 Feb 1868, *P. O’Shanesy #6*, *ser. 1* (lectotype, designated by Symon and Clarkson 1985, pg. 204: MEL [MEL12404]).

#### Type.

Based on *Solanumshanesii* F.Muell.

**Figure 48.**
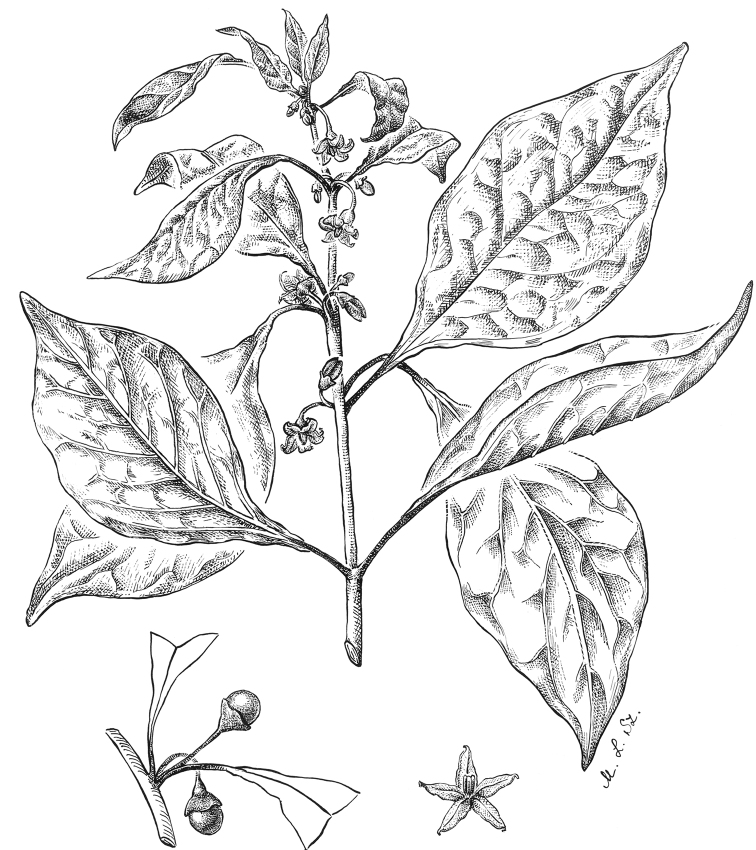
*Lycianthesshanesii* (F.Muell.) A.R.Bean. Drawing) by M.L. Szent-Ivany, first published in Symon and Clarkson (1985, as *S.shanesii* F.Muell.). Courtesy of the Board of the Botanic Gardens and State Herbarium (Adelaide, South Australia), reproduced with permission.

#### Description.

Shrubs or small trees 2–8 m tall, with single trunks to 10 cm diameter at breast height; stems terete, glabrous, with prominent white lenticels; new growth minutely puberulent with simple uniseriate golden 2–4-celled trichomes less than 0.5 mm long; bark of older stems pale greyish brown, blistering and peeling on older stems. Sympodial units difoliate, the leaves geminate, the leaves of a pair similar in shape, differing only somewhat in size. Leaves simple; blades of major leaves 7–12 cm long, 2.5–7.2 cm wide, elliptic to elliptic-obovate, broadest in the upper half or rarely at the middle, membranous, concolorous; adaxial surfaces completely glabrous; abaxial surfaces completely glabrous; principal veins 5–6 pairs, these sometimes purple abaxially (fide *Clarkson 4586*); base attenuate onto the petiole; margins entire (undulate fide *Clarkson 4586*); apex acute to acuminate; petiole 1–2.5 cm long, winged from the attenuate leaf base, with a few golden trichomes less than 0.5 mm long near the base; blades of minor leaves 2.5–7.5 cm long, 1–5.3 cm wide, like the major leaves in shape, texture, and pubescence; petioles 0.5–1.5 cm long. Inflorescences axillary fascicles with (1)2–3 flowers, minutely puberulent with a few golden simple uniseriate trichomes like those of the new growth; pedicels 1.2–1.7 cm long, ca. 1 mm in diameter at the base, ca. 2 mm in diameter at the apex, spreading at anthesis, almost completely glabrous, but often with a few golden simple uniseriate trichomes like those of stems along their length, articulated at the base; pedicels scars tightly packed in the leaf axils. Buds ellipsoid to fusiform with pointed tips, the corolla ca. halfway exserted from the calyx tube before anthesis, in young buds the calyx completely closed. Flowers (4)5-merous, heterostylous, the plants possibly andromonoecious (fide Symon and Clarkson 1985). Calyx with the tube ca. 3 mm long, ca. 4 mm wide, openly cup-shaped, with no appendages, the hyaline calyx rim ca. 0.5 mm wide, often irregularly torn and apparently 4–5-lobed, glabrous or with a few scattered simple trichomes like those of the pedicels. Corolla 1.6–2 cm in diameter, dark purple with the midveins darker than the surrounding tissue, stellate, lobed ca. 3/4 of the way to the base, thin edge of interpetalar tissue present, the lobes 7–7.5 mm long, 2.5–3 mm wide, spreading, glabrous on both surfaces except for the densely papillate, cucullate tips. Stamens equal or slightly unequal (fide Symon and Clarkson 1985); filament tube minute; free portion of the filaments 1–1.2 mm long, glabrous; anthers 4–4.5 mm long, 1–1.25 mm wide, ellipsoid and slightly tapering at the tips, yellow, poricidal at the tips, the pores tear-drop shaped, elongating to slits with age. Ovary conical, glabrous; style in long-styled flowers ca. 6 mm long, straight, glabrous, in short-styled flowers ca. 1.5 mm long, glabrous; stigma minutely capitate, the surface minutely papillate. Fruit a globose berry, 1.3–1.5 cm in diameter, bright red when mature, the pericarp glabrous, thin, shiny and somewhat translucent; fruiting pedicels 1.8–3.5 cm long, 1–2 mm in diameter at the base, 2–4 mm in diameter at the apex, somewhat woody, spreading or slightly hanging from the weight of the berries; fruiting calyx a plate with an undulate margin (appearing “ruffly”) subtending the berry, somewhat stiff and woody, the margins appearing lobed. Seeds 50–100 per berry, 3.5–4.5 mm long, 3–3.5 mm wide, flattened reniform, pale yellowish tan, the margin darker and thickened and the seed looking almost winged, the body of the seed shallowly pitted, the margins with the testal cells deeper, the testal cells with sinuate margins. Stone cells absent. Chromosome number: n=12 (Symon and Clarkson 1985; voucher *Clarkson 4585*).

**Figure 49.**
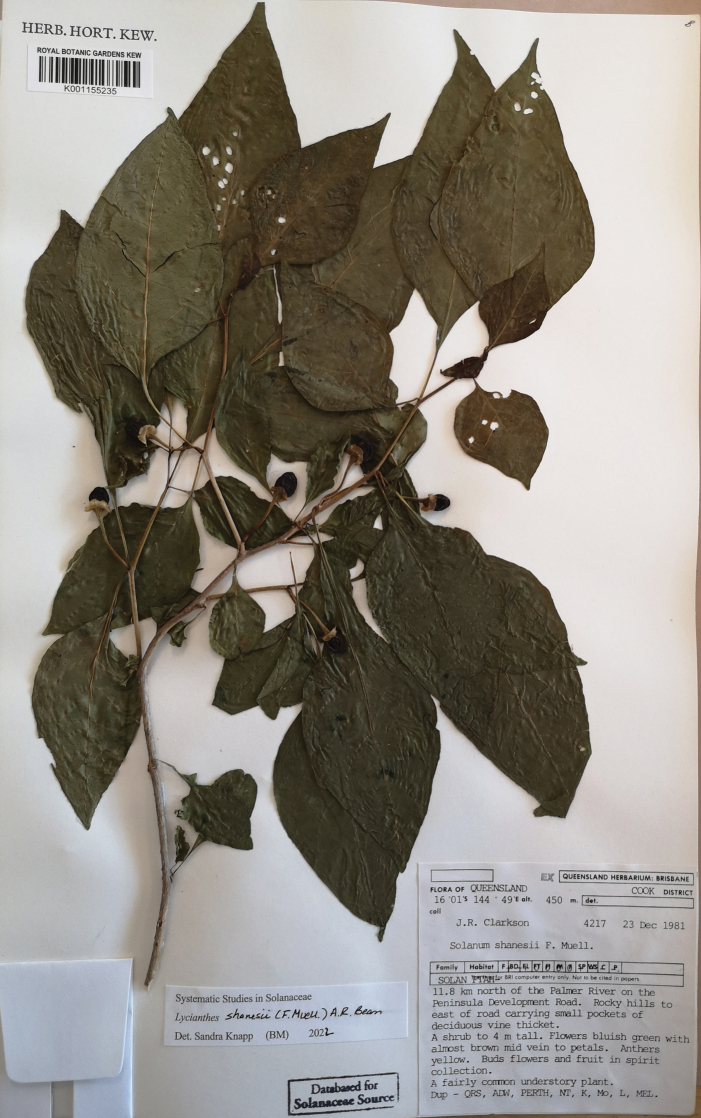
*Lycianthesshanesii* herbarium specimen. Australia. Queensland: *Clarkson 4217* (K001155235). Courtesy of the Trustees of the Royal Botanic Garden, Kew, reproduced with permission.

#### Distribution

**Figure 50.**
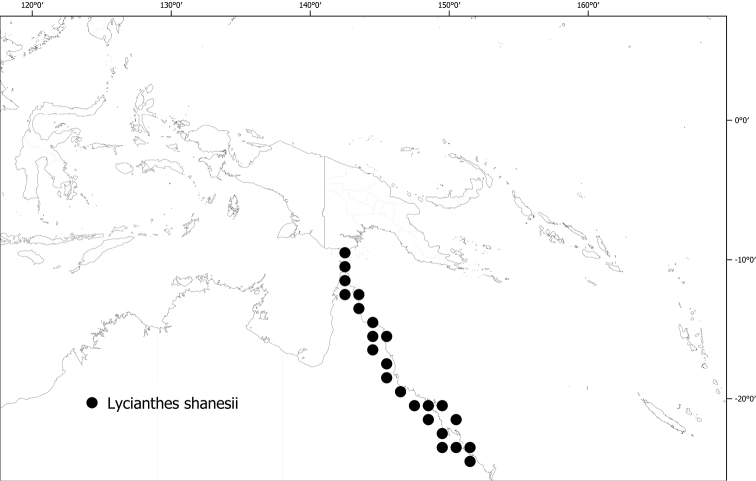
Distribution of *Lycianthesshanesii*.

**(Fig. 50).***Lycianthesshanesii* is endemic to Australia from central eastern Queensland and the York Peninsula.

#### Ecology and habitat.

*Lycianthesshanesii* grows in deciduous and semi-deciduous forests (“closed forest pockets” fide Symon and Clarkson 1985) and monsoon forests and is relatively common where it occurs; from sea level to 450 m elevation.

#### Common names.

None recorded.

#### Preliminary conservation assessment

**(IUCN 2020).**EOO (349,347 km^2^ - LC); AOO (196 km^2^ - EN). *Lycianthesshanesii* is known from more than 10 localities and has quite a wide distribution on the eastern coast of the Cape York Peninsula and on islands in the Torres Strait; populations occur in lands managed by aboriginal peoples for conservation. This suggests a preliminary threat status of Least Concern (LC) but documentation of threats to the dry forests of northern Australia may cause this to be modified.

#### Discussion.

*Lycianthesshanesii* was described based only on fruiting collections and not included in Bitter (1919) nor in Symon (1981a); Bitter, writing between the two World Wars in Europe, would not have had access to the specimens held in Australia, and Symon (1981a) thought it was a species of *Capsicum*. Later, Symon and Clarkson (1985) described it in full (as *S.shanesii*) from Clarkson’s flowering Queensland collections, where the poricidal anthers clearly showed it was not *Capsicum*. Symon and Clarkson (1985) realised it was distinct from the taxa native to nearby New Guinea and suggested it was closely related to *L.synanthera* and *L.heteroclita* (Sendtn.) Bitter of Central America, rather than to other species from southeast Asia or New Guinea. They (Symon and Clarkson 1985) discounted long-distance dispersal for the occurrence of *L.shanesii* (as *S.shanesii*) in Australia.

*Lycianthesshanesii* was included in the Solanaceae-wide phylogeny of Särkinen et al. (2013) and resolved as sister to the clade of *Lycianthes* that included all the Asian species sampled (*L.biflora* and *L.lysimachioides* (Wall.)Bitter) plus *L.synanthera* and other American species. Resolution of the relationship between *Lycianthes* and *Capsicum* has proved difficult (see Spalink et al. 2018), and none of the New Guinea or other southeast Asian taxa have ever been included in a phylogenetic analysis.

Like *Lycianthesvitiensis*, *L.shanesii* is a tree with a single trunk, unlike most of the New Guinea species that are shrubs or woody vines. *Lycianthesshanesii* grows in deciduous or semi-deciduous forests, rather than the wet, mossy forests inhabited by the other taxa treated here. It can be distinguished from *L.vitiensis* by its sparse, golden pubescence on the new growth and young stems; *L.vitiensis* has dense, tangled, reddish brown pubescence on stems and young leaves. The inflorescence of *L.shanesii* is strictly axillary and few-flowered while that of *L.vitiensis* has a short axis and is many-flowered.

The flower buds of *Lycianthesshanesii* resemble those of *L.rostellata* of New Guinea in being long-ellipsoid and somewhat beaked and the anthers are large (4–4.5 mm long in *L.shanesii* and 5–6 mm long in *L.rostellata*) and similarly tapered at the tips. *Lycianthesshanesii* is easily distinguished from *L.rostellata* in its distribution and habitat, and also by the wider corolla lobes (3–4 times longer than wide in *L.shanesii*, 4–5 times longer than wide in *L.rostellata*). *Lycianthesshanesii* also clearly differs in the pubescence of young stems; *L.rostellata* has dense, stiff antrorse pubescence on young stems while *L.shanesii* has sparse, golden trichomes on the young stems.

Symon and Clarkson (1985) cited Symon (1981b) as the designation (“proposal”) of the lectotype for *Solanumshanesii*. The citation of lectotype in Symon (1981b) is ambiguous and could refer to either of the two syntypes (*O’Shanesy 6 ser. 1* or *Dallachy 435*), thus I consider the explicit citation of the O’Shanesy collection as lectotype in Symon and Clarkson (1985) as the effective lectotypification of this name.

#### Specimens examined.

Australia. **Queensland**: Mount Stuart, 9 km S of Townsville, 14 Dec 1991, *Bean 3865* (AD, BRI); South Kennedy, Hazelwood Gorge, 13 km SSW of Eungella, 15 Dec 1992, *Bean 5271* (BRI); Wigton Island, c. 50km NE of Mackay, 28 Jun 2000, *Bean & Champion 16706* (BRI); Cook, 11.8 km N of the Palmer River on the Peninsula Development Road, 450 m, 23 Dec 1981, *Clarkson 4217* (AD, BRI, DNA, K, MBA, MEL, QRS); 11.7 km N of the Palmer River on the Peninsula Development Road, ca. 500 NE of the road, 450 m, 14 Mar 1983, *Clarkson 4585* (AD, BRI, CANB, K, MBA, MEL, MO, NSW, QRS), 14 Mar 1983, *Clarkson 4586* (AD, BRI, K, MBA, QRS), 31 Jan 1984, *Clarkson 5131* (AD, BRI, CANB, K, QRS); 3.4 km N of Spear Creek on the Peninsula Development Road, 11.3 km N of the Palmer River Crossing, 4 Mar 1987, *Clarkson & McDonald 6674* (BRI, QRS); 12 km from the East Normanby River crossing on the Lakeland Downs to Cooktown road, 6 Mar 1987, *Clarkson & McDonald 6768* (BRI, QRS); Dauan Island, Mount Cornwallis, 17 Feb 1993, *Clarkson 7813* (AD, BRI, CANB, DNA, MO, NSW); Mt White, c. 2 km SSW of Coen, 20 Dec 1989, *Clarkson 8205* (AD, BRI, MBA, MEL, QRS); North Kennedy, Granite Ironbark Hill, high range army training area, 31 Dec 1996, *Cumming 15486* (BRI); Mt Fox, 18 Feb 1997, *Cumming 15841* (BRI); Rockhampton, 17 Mar 1863, *Dallachy 345* (MEL, P); Cook, Hannibal Island, near Shelburne Bay, ca. 16 km W of Helby H, 3 Jul 1969, *Done s.n.* (BRI); Browns Peak, 75.4km ENE of Lakefield Homestead, Starcke Pastoral Holding (GR 7868-661813) RF site 49, Cape York Peninsula, 9 May 1993, *Fell et al. 3232* (BRI); Cape Melville National Park NP 4 Eumangin/Temple Creek catchment, 11.5km NW of Barrow Point, 79.2 km NNE of Lakefield Ranger Base, 4 May 1994, *Fell 4314* (BRI); Altanmoui Range, Cape Melville National Park, 1.6 km E of Flat Hill, 62.6 km NE of Lakefield Ranger Base, 4 May 1994, *Fell & McDonald 4354* (BRI); Pulu Islet, off western shore of Mabuiag Island, Torres Strait, 14 Apr 2009, *Fell 10000* (BRI, CNS, DNA); South Kennedy, Mount Bella Vista, 17 Dec 1992, *Fensham 579* (BRI); Leichhardt, ‘Clifton’, northern Boomer Range, 7 Feb 1993, *Fensham 718* (BRI); Back Creek, “Killarney”, Connors Range, 1 Mar 1993, *Fensham 749* (AD, BRI); Leichhardt, ‘Fort Cooper’, 7 Apr 1993, *Fensham 823* (BRI); Cook, NPR166, Black Mountain, Helenvale Road, Site 17, 12 Mar 2001, *Ford & Holmes 2647* (BRI); Port Curtis, Camoo Caves, 11 Jun 1989, *Forster & Tucker . 5108* (BRI); SF 471, Mount Coulston, 4 Oct 1989, *Forster & Bean 5808* (BRI); Pine Mountain, State Forest 79, 21 Apr 1991, *Forster 8012* (BRI, MEL); Cook, Possum Scrub, 22 Jun 1994, *Forster & Tucker 15280* (BRI); Bolt Head, Temple Bay, 26 Jun 1996, *Forster 19385* (BRI); 1.5 mls E of the highway, Byerstown Range, May 1980, *Godwin C-881* (QRS); Kings Plains Station, Barron Range SW of Cooktown, 7 Jun 1983, *Godwin C-2416* (QRS); Maitland Downs Holding, 560 m, 26 Jan 1989, *Gray 4972* (DNA, MO, QRS), *Gray 4974* (QRS); Yam Island, Torres Strait, 8 Feb 1989, *Gray 4981* (QRS); Kum Kum Range, Nychum Station, 6 Apr 1996, *Gray 6706* (QRS); OK Mine Road, 11 km from Burke Developmental Road, 2 Apr 2002, *Gray 8068* (QRS); Cook, Great Barrier Reef, Restoration Rock, near Cape Weymouth, Portland Roads, 24 Jul 1969, *Heatwole s.n.* (BRI); Great Dividing Range, S of Byerstown, 10 Jun 1971, *Hyland 5222* (QRS); Lankelly Creek Road, 6 Apr 1976, *Hyland 8713* (QRS); Mutee Head, 26 May 1981, *Hyland 11071* (QRS); Maitland Downs Holding, Parish of Byerstown, 13 Dec 1988, *Hyland 13764* (QRS), 11 Mar 1993, *Hyland*, *B. 14694* (QRS); Mt White, Coen, 22 Apr 1993, *Hyland 14781* (QRS); Haggerstone Island, 10 May 1994, *Hyland. 15119* (QRS); About 10 km north of the Palmer River Roadhouse, 12 Feb 1997, *Hyland 15534* (BRI, QRS); Mt White, Coen, 20 Jan 2000, *Hyland 16345* (BRI, QRS); Maitland Downs Holding, 29 Sep 1988, *Hyland 25551 RFK*, (QRS), 13 Dec 1988, *Hyland 25640 RFK* (BRI, QRS); Cook, Cooktown Development Road opposite Bonnie Glen Station turnoff, 8 Jun 1996, *Jago 4014* (BRI); Unnamed basalt-capped hill on Springvale Station, adjacent to Plum Tree Creek, 13 Mar 2017, *Kerrigan 1343* (CNS); North Kennedy, Upper slopes of high ridge, ca. 3.5km SW of Earlando, Dryander National Park, 2 Jun 1994, *McDonald & Champion 5862* (BRI); 3.4 km N of Spear Creek on the Peninsula Development Road. 11.3 km N of the Palmer River crossing, 4 Mar 1987, *McDonald 6674* (AD); 12 km from the east Normanby River crossing on the Lakeland Downs to Cooktown road, 6 Mar 1987, *McDonald 6768* (AD); Rocky knob just S of the divide on the Palmer River road, 23 Nov 1972, *Nicholson AFO-4776* (QRS); Port Curtis, Mt Archer, Rockhampton, 12 Mar 2003, *Nicholson NJN 455* (BRI); Rockhampton, 1 Feb 1869, *O’Shanesy s.n.* (MEL); Cook, King’s Plain Stn, Mt Emily, 9 Jan 2014, *Roberts KRM-15160* (BRI); Mt Pinnacle, 4 Mar 1987, *Sankowsky 615* (QRS); Port Curtis, over hanging path to caves, Olsen’s Capricorn Caves, Olsen’s caves Rd, The Caves, 12 Feb 2014, *Shapcott & Howard MGH-40* (BRI); Colosseum Cave, Mt Etna Caves NP, N of Rockhampton, 1 Apr 1993, *Thomas 8951* (BRI); 18 km NNW of Gladstone, 2 Mar 1997, *Thompson & Turpin GLA-22* (BRI); Middle Percy Island, 5 Mar 1906, *Tryon s.n.* (BRI); Olsens’ Caves, 1 Feb 1989, *Vavryn s.n.* (BRI); Cammoo Caves, 1 Jan 1987, *Vavryn 17* (BRI); Cook, Cooktown Developmental Road-Sackleys Hill, 6 Jan 2002, *Wannan & Jago 2328* (BRI, NSW); Seisia Village, Northern Peninsula Area, 17 Jan 1998, *Waterhouse 4806* (BRI, DNA), 12 Mar 1999, *Waterhouse 5114* (CANB), *Waterhouse 5114* (BRI, DNA); tributary of Mossman River, 25 km S of Laura, 8 Mar 2017, *Worboys 1344* (CNS).

### ﻿17. Lycianthes vitiensis

Taxon classificationPlantaeSolanalesSolanaceae

(Seem.) A.R.Bean, Austrobaileya 6(3): 568. 2003.

76D1D95A-E6AD-52EA-821A-15C0B8E578AA

Figs 51 , 52

Solanum vitiense Seem., J. Bot. 1: 206. 1863. Type. Fiji. Ovalau: “Port Kinnaird, July 1860”, *B. Seemann 340* (lectotype, designated here: K [K000759477]; isolectotypes: BM [BM000846686], E [E00279459], G [G00343067], GH [00077854], P [P00315328], W [acc. # 1889-076975]).
Brachistus feddei Reinecke, Bot. Jahrb. Syst. 25: 674. 1895, as “Feddei”. Type. Samoa. Upolu: Mulifanua, Oct 1893, *F. Reinecke 78* (lectotype, designated here: WRSL [WR LB 066181]; isolectotype: BISH [acc. # 181507, BISH1005071]).
Solanum rechingeri Witasek, Repert. Spec. Nov. Regni Veg. 5: 165. 1908, as “Rechingeri”. Type. Solomon Islands. “Shortlands Inseln, Insel Poperang”, 1905, *K. Rechinger & L. Rechinger 4398* (lectotype, designated by Symon 1985, pg. 67 [as holotype]: W [acc.# 0003088]; isolectotypes: LAE [acc. # 231328, acc. # 231327]).
Lycinathes rechingeri (Witasek) Bitter, Abh. Naturwiss. Vereins Bremen 24 [preprint]: 504. 1919, as “Rechingeri”. Type. Based on Solanumrechingeri Witasek.

#### Type.

Based on *Solanumvitiense* Seem.

**Figure 51.**
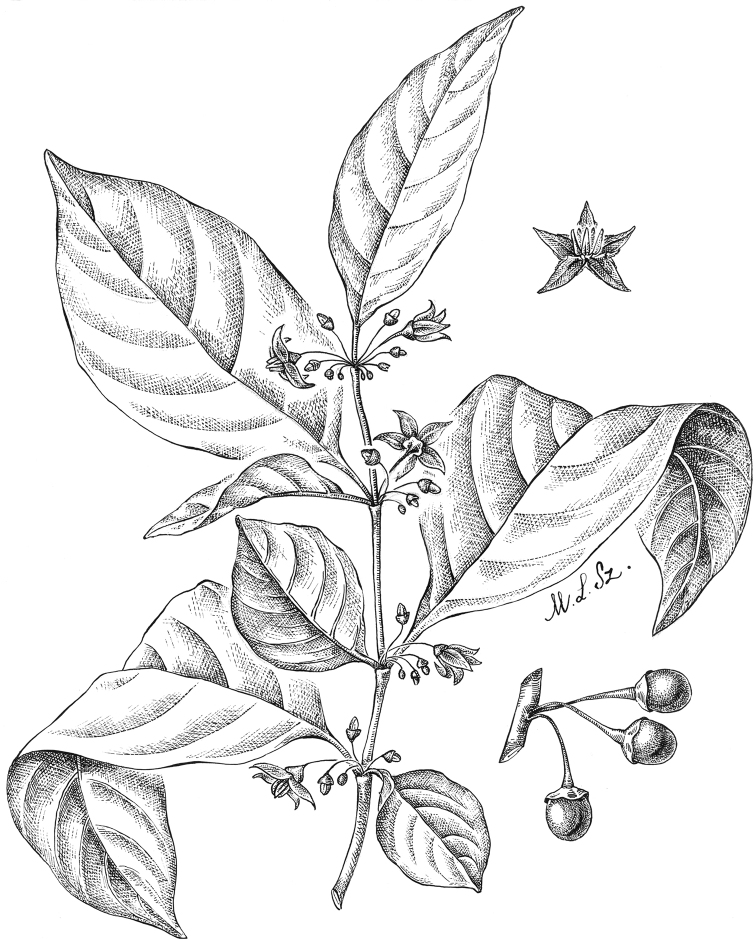
*Lycianthesvitiensis* (Seem.) A.R.Bean. Drawing by M.L. Szent-Ivany, first published in Symon (1985: fig. 24, as *S.vitiense* Seem.). Courtesy of the Board of the Botanic Gardens and State Herbarium (Adelaide, South Australia), reproduced with permission.

#### Description.

Trees to 15 m tall, with single trunks to 30 cm diameter at breast height; stems terete, glabrous or minutely puberulent (especially near the nodes) with weak-walled, 10–15-celled simple uniseriate trichomes to 0.25 mm long, these drying golden reddish brown, soon glabrescent; new growth densely puberulent-papillate with weak-walled 10–15 celled simple uniseriate trichomes like those of the stems near the nodes; bark of older stems pale grey or whitish grey. Sympodial units difoliate, the leaves geminate, the leaves of a pair, differing in size but not in shape. Leaves simple; blades of major leaves 8–28 cm long, (2.5)4–12.5 cm wide, elliptic to broadly elliptic, widest at the middle, somewhat discolorous, membranous to thick and chartaceous (rubbery in live plants?); adaxial surfaces shiny, completely glabrous; abaxial surfaces glabrous; principal veins 7–10 pairs, prominent beneath, drying reddish brown; base acute; margins entire; apex acute to acuminate; petioles 2–4.5 cm long (or perhaps even longer in larger leaves), glabrous; blades of minor leaves 3.8–11.5 cm long, 2.5–6.5 cm wide, shape, texture and pubescence like than of the major leaves; base acute; margins entire; apex acute to acuminate, sometimes rounded; petioles 0.7–1.5 cm long, glabrous. Inflorescences axillary 4–10 flowered fascicles with several flowers usually open at once, sometimes developing a woody axis or axes to 0.6 cm long, pubescent with floccose reddish brown trichomes like those of the new growth; pedicels at anthesis 1.5–2 cm long, 0.5–0.75 mm in diameter at the base, 1.5–2 mm in diameter at the apex, spreading, glabrous, articulated at the base; pedicel scars tightly packed in the leaf axils or along the short inflorescence axes. Buds ellipsoid, the corolla exserted ca. halfway from the calyx tube before anthesis. Flowers 5-merous, heterostylous and unisexual, specimens have only short-styled flowers or long-styled flowers and fruits, the plants probably dioecious (described as androdioecious, with staminate and hermaphroditic flowers, by Reinecke 1898, as *Brachistusfeddei*). Calyx tube 3–3.5 mm long, 3–5 mm wide, openly cup-shaped, chartaceous or somewhat fleshy, often purple or flushed with purple, sparsely puberulent with floccose trichomes like those of the stems, without appendages, the rim hyaline and irregular (“ruffly”). Corolla 1–1.8 cm in diameter, white, sometimes fragrant, stellate, lobed ca. 3/4 of the way to the base, interpetalar tissue present, the lobes 5–8 mm long, 2.5–3 mm wide, spreading to somewhat reflexed, adaxially glabrous, abaxially glabrous or densely papillate, the tips and margins always densely papillate. Stamens equal; filament tube minute; free portion of the filaments 1–1.5 mm long, glabrous; anthers 2.5–4 mm long, 1–1.5 mm wide, ellipsoid and slightly tapering at the tips, white, cream-colored or yellow, poricidal at the tips, the pores tear-drop shaped and markedly splitting to slits with age, some plants apparently with longitudinally dehiscent anthers and no pores (see figure 5 in Smith 1991). Ovary conical, glabrous, vestigial in short-styled flowers; style in short-styled flowers to 1.5 mm long, in long-styled flowers 5.5–6 mm long, straight, glabrous; stigma clavate, occasionally somewhat bilobed, the surfaces minutely papillate. Fruit a globose berry, 0.5–1.5 cm in diameter, green to orange when immature and bright red when ripe (description as “atroviridi” by Reinecke 1898 possibly refers to dry material; blue fide *Kajewski 1863*), the pericarp glabrous, shiny, translucent; fruiting pedicels 2–2.5 cm long, ca. 1.5 mm in diameter at the base, 2–2.5 mm in diameter at the apex, spreading, glabrous, woody, corky and rugose; fruiting calyx a cup with a spreading thinner rim beneath the fruit, the rim thinner than the woody calyx cup, the rim sometimes reflexed. Seeds (10)30–50 per berry, 4–5 mm long, 3.5–4 mm wide, flattened and rounded with a prominent notch, yellowish tan, the surfaces deeply pitted at the thickened margins with pentagonal to rectangular testal cells, the centre of the seed smooth or only shallowly pitted with testal cells pentagonal in outline. Stone cells absent. Chromosome number not known.

**Figure 52.**
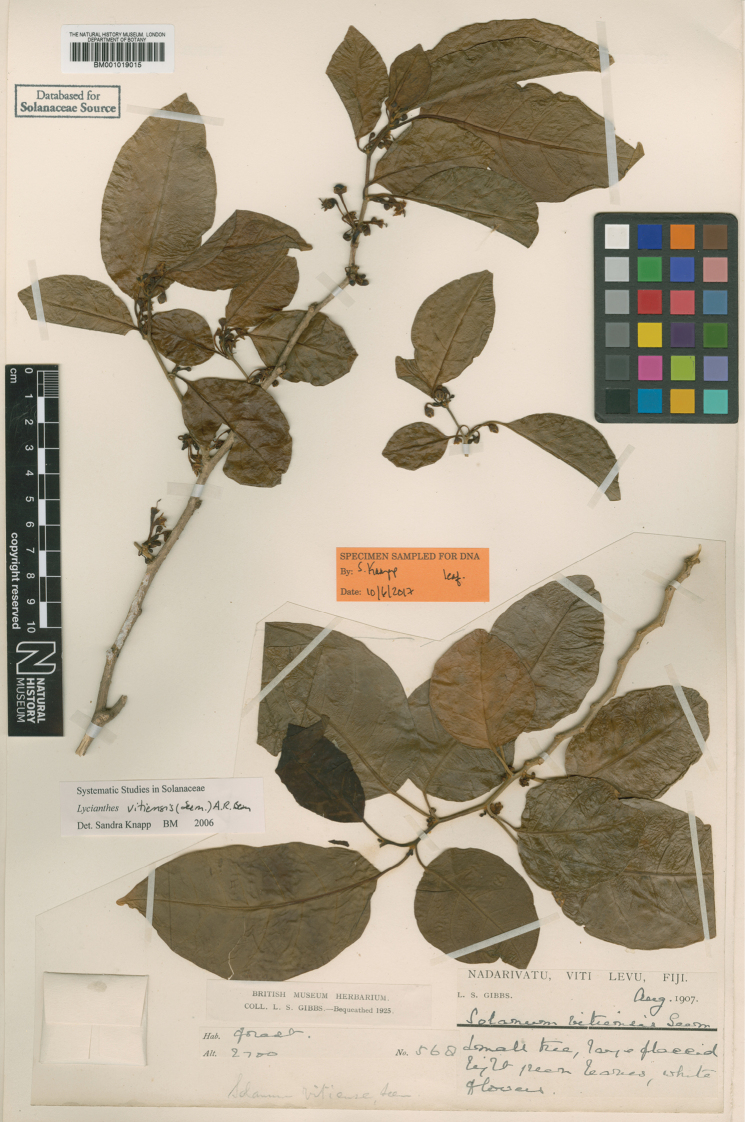
*Lycianthesvitiensis* herbarium specimen. Fiji. Viti Levu: *Gibbs 568* (BM001019015). Courtesy of the Trustees of the Natural History Museum, London, reproduced with permission.

#### Distribution

**Figure 53.**
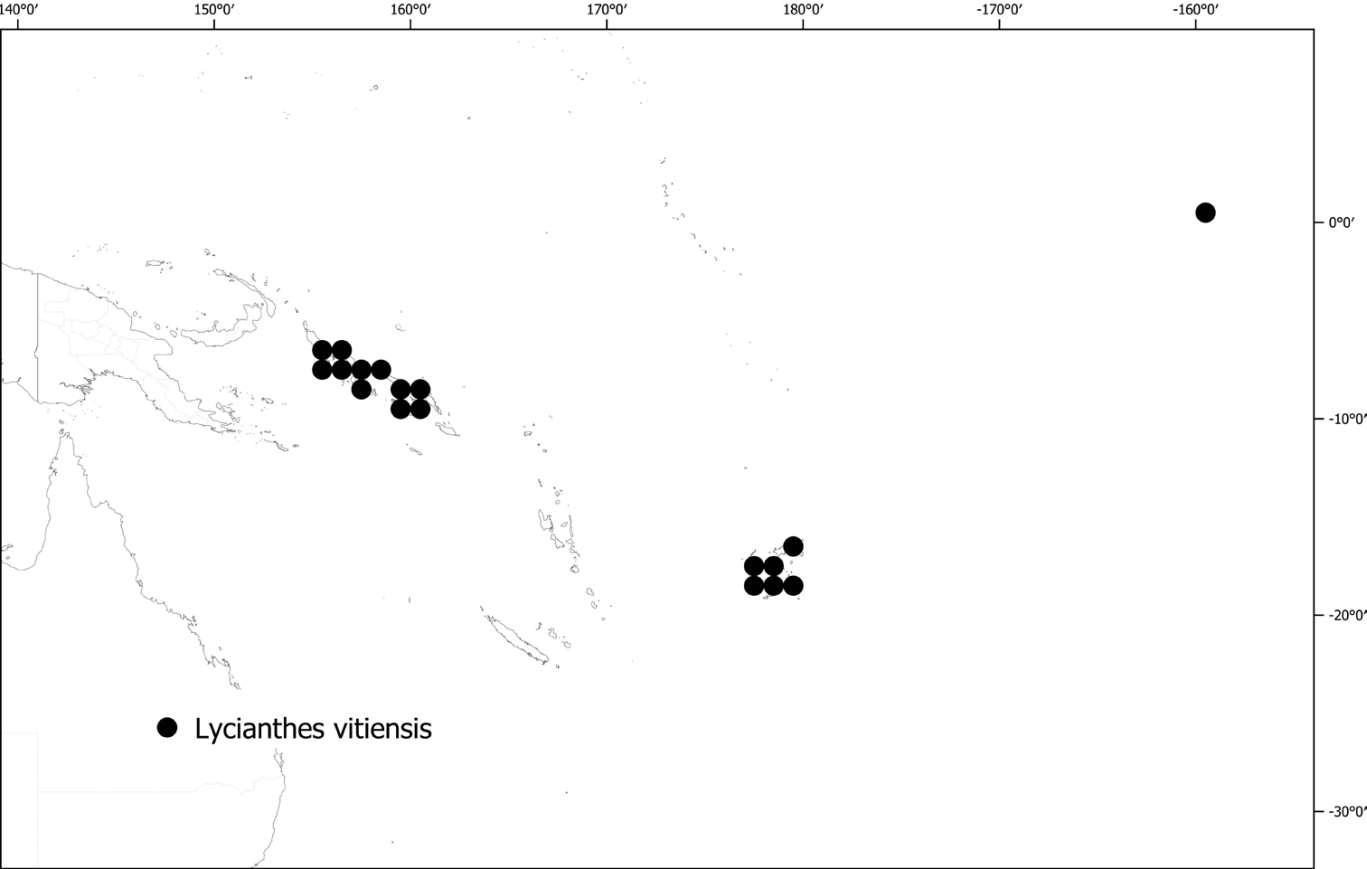
Distribution of *Lycianthesvitiensis*.

**(Fig. 53).***Lycianthesvitiensis* is a mostly Pacific species, known from New Guinea (Bougainville Island), the Solomon Islands, Samoa, Fiji and Tonga. It is very common in Fiji (Smith 1991).

#### Ecology and habitat.

*Lycianthesvitiensis* is a plant of many forest types, and has been collected both in primary and secondary rainforests and along streams; from 30 to 1,200 m elevation.

#### Common names.

There are no standard common names (Smith 1991), but several different names have been recorded across its range. Fiji: malawatha (*Smith 5828*), nandata (*Degener 14832*), nggalinggwa (*Smith 5270*), teleniva (*Smith 5250*), tungasele (*Smith 1244*), vualiku (*Smith 5250*). New Guinea (Bougainville): em-wuye (*Kajewski 1863*), kosiou (*Waterhouse 88*), neketigo (*Kajewski 1800*), nua (*Waterhouse 272B*). Samoa: koriele (*Guppy 160*), uagagi (*Christopherson 2697*), u’agani (*Bristol 12146*)]. No uses have been recorded.

#### Preliminary conservation assessment

**(IUCN 2020).**EOO (1,758,710 km^2^ - LC); AOO (288 km^2^ - EN). *Lycianthesvitiensis* is known from more than 10 localities on many islands in the western Pacific and is often described as a “common tree” on herbarium labels. This suggests it should be assessed as Least Concern (LC), despite its island occurrence.

#### Discussion.

*Lycianthesvitiensis* is a relatively common slender tree; both Seemann (1863) and Reinecke (1898) recognised it as having long- and short-styled flowers. Bitter (1919) did not include this species in his treatment of *Lycianthes*, perhaps because in German herbaria it was often cited as a species of *Brachistus* Miers (see below) due to longitudinal anther dehiscence. Symon (1985) suggested that *L.vitiensis* (as *S.vitiense*) showed intermediate characters between *Lycianthes* (as subgenus Lycianthes) and *Solanum*, citing the short inflorescence axis and calyx that sometimes appears five lobed.

*Lycianthesvitiensis* is not easily confused with any other species of *Lycianthes* treated here. Like *L.shanesii* it is a small tree; it differs from that species in its many-flowered inflorescence with a short axis (versus few-flowered strictly axillary inflorescences in *L.shanesii*) and in the dense, tangled reddish brown pubescence of 10–15-celled trichomes on young stems and inflorescences (versus sparse golden pubescence of 2–3-celled trichomes of *L.shanesii*). *Lycianthesvitiensis* is not sympatric with any other *Lycianthes* in Asia, Australia or the Pacific. It is most similar to *L.banahaensis* (Elmer) Bitter of the Philippines (not treated here) with which it shares floccose reddish brown pubescence of stems and new growth, but differs from that species in its white or violet (versus yellow) flowers and red (versus orange) mature berries.

*Lycianthesvitiensis* is unusual in *Lycianthes* in its anther dehiscence by both apical pores and more commonly by longitudinal slits. Many species of *Solanum* with ellipsoid anthers have tear-drop shaped apical pores that split longitudinally with age and drying (see Särkinen et al. 2018; Knapp et al. 2019). The longitudinally dehiscent anthers of *L.vitiensis*, in contrast, do not appear to be poricidal at the outset (see figure 5 in Smith 1991: 24). This difference does not correlate with the sex expression of individual flowers, and its occurrence merits further study.

Flowers of *Lycianthesvitiensis* are sometimes described as fragrant (e.g., *Mauriasi et al. BISP-13952*), other times as with no smell (e.g., *Mauriasi et al. BISP-8491*); this may be due to time of day or degree of anthesis,

In the protologue of *Solanumvitiense*, Seemann (1863) only cited his collection number “340” but no herbarium. I have selected the specimen at Kew (K000759477) as the lectotype, because it is well-preserved and has a clear label with both the number and the locality. Other specimens at Kew cited as types by Symon (1985) are not clearly duplicates of this number. Smith (1991) cited two sheets at Kew as “holotype”, but this does not constitute effective lectotypification.

In the protologue of *Brachistusfeddei*, Reinecke (1898) cited three of his own Samoan collections: *Reinecke 58* (male), *Reinecke 58a* (male) and *Reinecke 78* (hermaphrodite). Reinecke’s own collections are housed in WRSL. Of the collections located there *Reinecke 78* is the best preserved and has bisexual flowers, therefore the duplicate of this collection at WRSL is here selected as the lectotype. The duplicate of *Reinecke 78* held at BISH has both the locality and date of collection matching the protologue and is and isolectotype (BISH1005071). A note on the specimen from A.C. Smith dated June 1988 indicates the holotype is at B; this was a common mistaken assumption for many names coined by German botanists. Nowhere in Reinecke’s protologue does he indicate material in Berlin; his personal herbarium is held in WRSL (now in Poland). Duplicates of Reinecke’s collections held at K (*Reinecke 58*, K000759467; *Reinecke 58a* K000759466) cited by Symon as types, have different dates than those in the protologue (September in the protologue and October on the Kew specimens) and are thus I do not strictly consider them isolectotypes.

#### Specimens examined.

Fiji. ‘Fiejie Islands’, *US Exploring Expedition under the command of Capt Wilkes s.n.* (NY). **Lau Archipelago**: Kabara, ‘Kambara’, limestone formation, 2 Mar 1934, *Smith 1244* (K, NY, P, US). **Ngau**: hills E of Herald Bay, inland from Sawaieke, 300–450 m, 15 Jun 1953, *Smith 7797* (K, NY, US); **Ovalau**: sin. loc., 15 Oct 1924, *Bryan 608* (US); near summit of main range W of Levuka, 200 m, 26 Jan 1928, *Gillespie 4446* (K, US); hills above Levuka reservoir, 400 m, 30 Jan 1928, *Gillespie 4517* (NY); hills east of Lovoni Valley, 300–500 m, 11 May 1953, *Smith 7343* (K, NY, P, US); slopes of Mt. Koronimiko, vicinity of Thawathi, 250–350 m, 13 Jul 1953, *Smith 8082* (K, NY, P, US); **Vanua Balavu**: ‘Vanua Mbalavu’, northern limestone section, 2 Apr 1934, *Smith 1508* (K, NY, P, US); **Vanua Levu**: Mt. Delaikoro, Macuata, 762–914 m, 21 Aug 1962, *Koroiveibau 12807* (K); Cakaudrove, Navonu, Nov 1964, *Qoro USDA-14091* (K, NY); southern slope of Korotini Range, below Navitho Pass, Thakaundrove, 300–650 m, 21 Nov 1933, *Smith 574* (K, NY, P, US); Yanawai River region, Mount Kasi, Thakaundrove, 300–430 m, 10 May 1934, *Smith 1826* (K, NY, US); **Viti Levu**: Serua District, 152 m, 14 Jun 1961, *Bola 41* (K); vicinity of Nandarivatu, TholoNorth, 700–900 m, 16 Mar 1941, *Degener 14832* (K, NY, US); Vuninatiambua, Navai, Tholo North, 750–900 m, 4 Feb 1941, *Degener 14875* (K, MO, NY, US); Mbulu, near Sovi Bay, Tholo West, 20 Apr 1941, *Degener 15032* (K, MO, NY, P, US); Mt. Victoria [Mount Tomanivi], 29 Dec 1968, *Degener et al. 32083* (E, NY); S of Naboutini, Serua, 274 m, 17 Dec 1963, *Forestry Department 965* (K); Nadarivatu, 823 m, Aug 1907, *Gibbs 568* (BM, K); Tamavua woods 8 miles above Suva, 150 m, 6 Aug 1928, *Gillespie 2016* (K, NY, P, US); above waterfall near Namuamua, Namosi, 400 m, 2 Oct 1927, *Gillespie 3251* (K, NY, US); slopes of Mount Victoria [Mount Tomanivi], 1,000 m, 29 Nov 1927, *Gillespie 4082* (NY); Albuggi Levu, *Graeffe*, *E. s.n.* (K); Mount Evans [= Mount Koroyanitu], Lautoka, 914 m, 3 Oct 1920, *Greenwood 127* (K); Lautoka, Mt. Evans [=Mount Koroyanitu], 750 m, 16 Sep 1945, *Greenwood 1151* (K, US); banks of Samaruna [=Samambula?] River, *Horne 714* (K); Nadroga-Navosa, Nausori Highlands, 579 m, 20 Jul 1964, *Kuruveli 13888* (K); Naboutini, Serua, 9 Sep 1964, *Kuruveli & Qoro 14007* (K); Tailevu, Dakuivuna, 13 Nov 1957, *Ledua 11018* (K); 9 miles above Suva, Jul 1932, *Meebold 17036* (K); prov. Ba, Nadarivatu, 1,067–1,219 m, 24 Oct 1962, *Parham & Koroveibau 13051* (K); Naitsiri, Princes [Princess] Road, 21 Apr 1936, *Raigiso 3141* (K); sin. loc., *Seemann 42* (BM); [Tailevu], 1860, *Seemann 387* (K, P); western and southern slopes of Mount Tomonivi (Mt. Victoria) [Mount Tomanivi], Mba (formerly Tholo North), 850–1,150 m, 7 Jul 1947, *Smith 5250* (K, NY, P, US); western and southern slopes of Mount Tomonivi (Mt. Victoria), Mba (formerly Tholo North), 850–1,150 m, 7 Jul 1947, *Smith 5270* (K, NY, P, US); northern portion of Rairaimatuku Plateau, between Nandrau and Nanga, Nandronga and Navosa (formerly Tholo North), 725–825 m, 4 Aug 1947, *Smith 5501* (K, NY, P, US); hills between Nggaliwana and Nandala Creeks, south of Nauwanga, Mba (formerly Tholo North), 725–850 m, 26 Aug 1947, *Smith 5828* (K, NY, P, US); hills bordering Wainavindrau Creek, in vicinity of Wainimakutu, Namosi, 150–250 m, 17 Sep 1953, *Smith 8894* (K, NY, P, US); hills W of Wainikoroiluva River, near Namuamua, Namosi, 50–200 m, 15 Oct 1953, *Smith 8930* (K, NY, P, US); hills N of Ngaloa, in darinage of Waininggere Creek, Serua, 30–150 m, 19 Nov 1953, *Smith 9171* (K, NY, P, US); Serua, hills west of Waivunu Creek, between Ngaloa and Korovou, 50–150 m, 23 Nov 1953, *Smith 9264* (K, NY, US); Nambavatu [Creek], *Tothill*, *B.H. 556* (K), *Tothill 633* (K); path Kalumbo (Suva), 5 Aug 1926, *Tothill 636* (K); Nandarivatu [= Nadarivatu], 914 m, 1927, *Tothill 638* (K), Feb 1927, *Tothill 639* (K).

Papua New Guinea. **Bougainville (North Solomons)**: Bougainville Island, 22 miles NW of Tonlei Harbour, Buin subdistrict, 91 m, 25 Aug 1969, *Foreman NGF-45698* (A, K, L, LAE); Kugi-maru, Buin., 150 m, 2 Jun 1930, *Kajewski 1800* (BM, G, P); Kugumaru, Buin., 150 m, 12 Jun 1930, *Kajewski 1863* (BM, G, P); vicinity of Aku [Aka] village, ca. 10 miles W of Buin Station, 30 m, 21 Sep 1964, *Schodde & Craven 4094* (A, G, K, L, LAE); “Melanesia, Siwai, Bougainville”, Sep 1932, *Waterhouse 66* (K, NY, US), Sep 1930, *Waterhouse 272*-*B*, (K);

Samoa. sin. loc., *Horne s.n.* (K), *Powell 365* (K), *Whitmee 52* (K), Aug 1875, *Whitmee 185 [a*] (K). **Savai’i**: far inland from Aopo, base line 4 of Division of Forestry 1968 forestry survey, 300 m, 28 Jun 1968, *Bristol 2146* (K, LAE, NY, US); above Salailua, 650 m, 22 May 1924, *Bryan 173* (K, US); Olo, [Mount Olomanu], 700 m, 4 Aug 1931, *Christophersen & Hume 2249* (K, NY, P, US), 9 Aug 1931, *Christophersen & Hume 2320* (K, NY, P, US), 9 Aug 1931, *Christophersen & Hume 2324 [a*] (P, US), 9 Aug 1931, *Christophersen & Hume 2354* (NY), 26 Aug 1931, *Christophersen & Hume 2518* (NY, P), 26 Aug 1931, *Christophersen & Hume 2521* (K), 26 Aug 1931, *Christophersen & Hume 2528* (US); above Salailua, 900 m, 22 Sep 1931, *Christophersen 2697* (K); Lo To, above Salailua, 750 m, 21 Oct 1931, *Christophersen 2895* (K, NY, P);); above Salailua, ca. 1,350 m, 7 Nov 1931, *Christophersen 3116* (P), 1,200 m, 5 Nov 1931, *Christophersen 3123* (NY); ueber Aopo [A’opo], Sep 1894, *Reinecke*, *F. 58 a* (K, WRSL); Olomono, uber Olomono, 600 m, 7 Sep 1905, *Vaupel s.n.* (MO); Forestry Block 25 inland from Asau, 29 Jun 1972, *Whistler 4* (US); above Ologogo, 700 m, 21 Aug 1973, *Whistler 520* (US); above Asau in Block 28, 550 m, 16 Oct 1973, *Whistler 891* (US). **Upolu**: Malololelei, near Malololelei, 550 m, 17 Aug 1929, *Christophersen 326* (NY, P); ‘Ins. Upolu’, *Graeffe s.n.* (BM, NY), Mar 1880, *Graeffe 1354* (K); sin. loc., 1896, *Hufnagel s.n.* (US); Tanumalala, 200 m, 11 Aug 1955, *MacKee 2995* (K); Upalu [Upolu sin. loc.], *von Mueller 13* (BM); Sameaberg [=Samea], Oct 1893, *Reinecke 58* (K, WRSL); Olonono [illegible-Bugrange?] [Cape Olonono], 7 Nov 1905, *Vaupel 104* (K, US); E of the main road near Tiavi, 700 m, 3 Oct 1973, *Whistler 1071* (K, US); just N of Lake Lanoanea, 600 m, 5 Nov 1973, *Whistler 1090* (US); near Mt. Le Pu’e, 750 m, 7 Dec 1973, *Whistler 1253* (US).

Solomon Islands. sin. loc., *Kajewski D s.n.* (BM, P). **Central Province**: Florida Island, Popinisura [=Nggela Islands, Nggela Sule], *Comins 235* (K); Rove Area, Big Nggela [=Nggela Sule], 91 m, 24 Jul 1969, *Gafui & collectors BSIP-16787* (K). **Choiseul Province**: Choiseul Island, East Mbirambira, West Choiseul, valley bottom, 15 m, 17 Jan 1970, *Gafui & collectors BISP-18862* (K). **Guadalcanal Province**: Berande River., 31 Dec 1930, *Kajewski 2388* (A, BM, G, P); NW Guadalcanal, Mt. Austen, W side of the trial plot, 304 m, 27 Nov 1964, *Kere BSIP-4936* (K), 23 Dec 1964, *Kere BSIP-5057* (K); NW Guadalcanal, Mt. Mambulu summit [=Mount Austen], 457 m, 19 Jul 1967, *Nakisi BSIP-8013* (K); Guadalcanal Island, Guadalcanal, west fork Tenau River, 30 Sep 1945, *Riley 45* (A, NY, US); Guadalcanal, Mt Austen near Honiara, 304 m, 13 Mar 1963, *Whitmore BSIP-776* (K); N Guadalcanal, Kukum, 0.5 mile inland for training college, 7 Feb 1964, *Whitmore BSIP-2567* (K). **Isabel Province**: Samusodu, SW Santa Ysabel, 85 m, 2 May 1966, *Beer’s collectors BSIP-7225* (K); Korigole Bay, SW Santa Ysabel, 48 m, 13 Jun 1966, *Beer’s collectors BSIP-7306* (K); Ysabel Island, Kolokofa R, NW Santa Isabel, 250 m, 5 Apr 1966, *Teona BISP-6362* (K, L, LAE, US); Santa Ysabel, near Maringe Lagoon, Molau Village, 670 m, 27 Oct 1963, *Whitmore BSIP-2437* (K). **Malaita Province**: N of Anihonora’a Village, E Malaita, 5 Sep 1969, *Gafui & collectors BSIP-16450* (K). **Western Province**: Shortland Island, May 1884, *Guppy 160* (K); SE Kolombangara, W of Vila River, 30 m, 20 Dec 1967, *Mauriasi & collectors BSIP-8491* (K); Fauro Island, Haliuna River area, 114 m, 16 Apr 1969, *Mauriasi & collectors BISP-13952* (A, K, L, LAE); NW Treasury Island [=Mono Island], Akolea River area, 60 m, 26 Apr 1969, *Mauriasi & collectors BSIP-14089* (K); Palusua, SE Mono, 22 m, 11 May 1969, *Mauriasi & collectors BSIP-14154* (K); New Georgia Group, Baga Island, 26 Jan 1964, *Whitmore’s collectors BSIP-3081* (K).

Tonga. **Vava’u**: above Ha’alaufuli on northeastern side of island, 60 m, 21 May 1953, *Yuncker 16103* (NY, US).

### ﻿18. Lycianthes wollastonii

Taxon classificationPlantaeSolanalesSolanaceae

(Wernham) A.R.Bean, Austrobaileya 6(3): 568. 2003.

4321FC32-1AF7-5BBF-9076-6258BD35B311

Figs 54 , 55

Solanum wollastonii Wernham, Trans. Linn. Soc. London, Bot. 9: 120. 1916. Type. Indonesia. Papua: Mount Carstenz [Puncak Jaya = Mount Jaya] “Camp VIII? To IX, 4,900 to 5,500 ft” [along Bandarong River], Aug 1912, *C.B. Kloss s.n.* (lectotype, designated by Symon 1985, pg. 69 [as holotype]: BM [BM001014583]).

#### Type.

Based on *Solanumwollastonii* Wernham.

**Figure 54.**
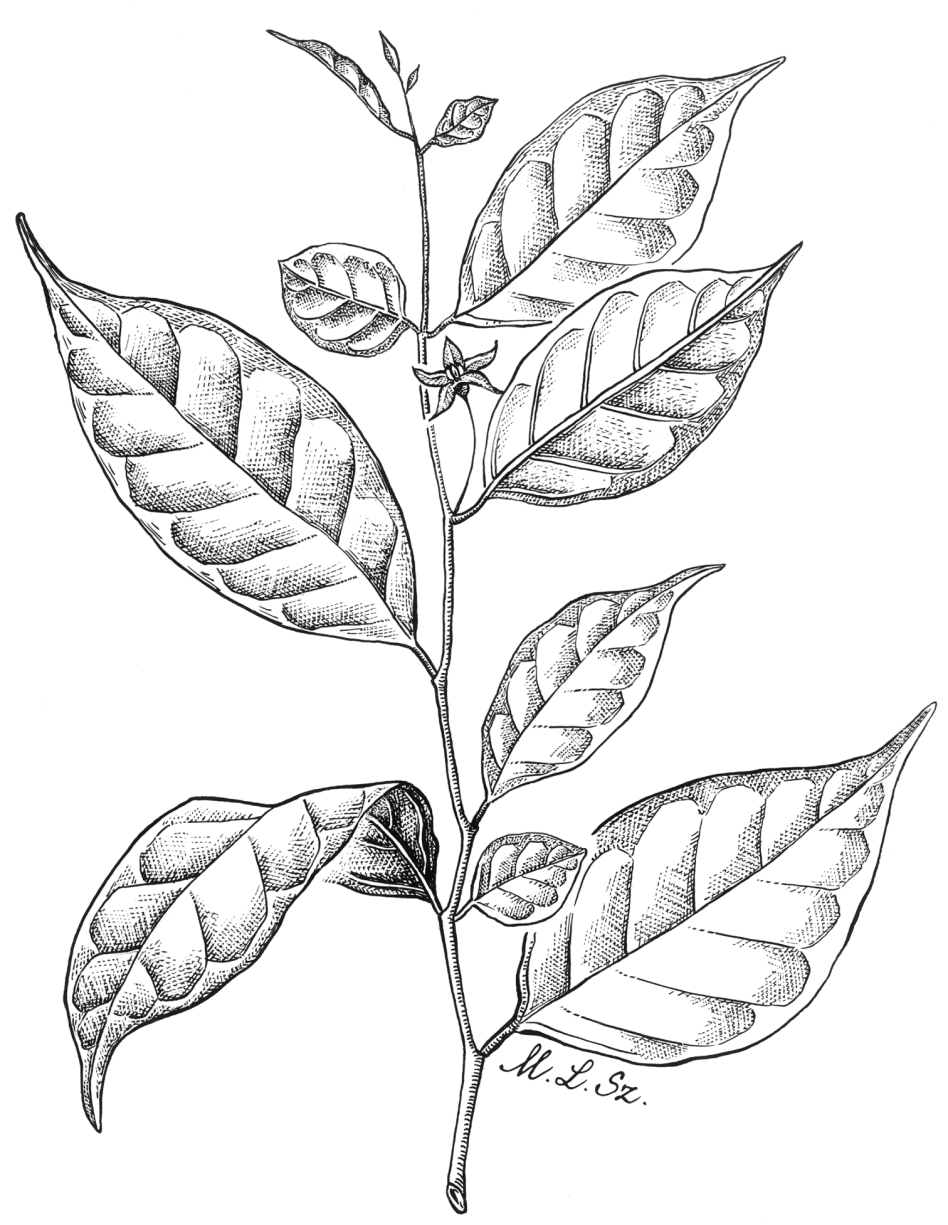
*Lyciantheswollastonii* (Wernham) A.R.Bean. Drawing by M.L. Szent-Ivany, first published in Symon (1985: fig. 25, as *S.wollastonii* Wernham). Courtesy of the Board of the Botanic Gardens and State Herbarium (Adelaide, South Australia), reproduced with permission.

#### Description.

Slender woody climber or epiphyte, to ca. 3 m tall (long); stems terete, glabrous, flushed with purple (fide Mustaqim et al. 2022); new growth minutely papillate, soon glabrescent; bark of older stems pale tan, peeling. Sympodial units difoliate, the leaves geminate, the leaves of a pair differing in size and sometimes in shape. Leaves simple; blade of major leaves 6–11 cm long, 2–4 cm side, elliptic, somewhat oblique, widest at the middle, discolorous, membranous; adaxial surfaces shiny, glabrous, the venation impressed, the midrib keeled; abaxial surfaces paler, glabrous, the veins drying reddish gold; principal veins 4–6 pairs, impressed above, reddish golden below, glabrous; base acute to cuneate; margins entire, somewhat revolute; apex abruptly acuminate with an elongate drip-tip 1–2 cm long; petioles 0.3–0.6 cm long, glabrous; blades of minor leaves 1.9–2.7 cm long, 1.5–2 cm wide, elliptic to narrowly elliptic, texture and pubescence like that of the major leaves; base acute; margins entire; apex acute or rounded; petioles ca. 0.2 cm long, glabrous. Inflorescences axillary 1–3(5) flowered fascicles, with only a single flower open at a time, glabrous; pedicels 1.8–2.2 cm long, ca. 0.5 mm in diameter at the base, ca. 1.5 mm in diameter at the apex, spreading and nodding from weight of flowers, green flushed with purple (fide Mustaqim et al. 2022), glabrous, articulated at the base; pedicel scars tightly packed in the leaf axils. Buds narrowly ellipsoid, the corolla strongly exserted from the calyx tube before anthesis. Flowers 5-merous, apparently perfect (only long-styled flowers seen). Calyx tube 3–3.5 mm long, 4–4.5 mm in diameter, openly cup-shaped, pale purple, glabrous, with 5 triangular appendages 1–2 mm long emerging 0.75–1 mm below the rim and perpendicular to the calyx tube, the rim entire. Corolla 1.6–2 cm in diameter, white, deeply stellate, lobed nearly to the base, interpetalar tissue present, the lobes ca. 10 mm long, ca. 2.5 mm wide, spreading and slightly cupped, glabrous, minutely papillate at the slightly cucullate tips. Stamens equal; filament tube minute; free portion of the filaments ca. 1 mm long, glabrous; anthers 4.5–5 mm long, ellipsoid, yellow, poricidal at the tips, the pores tear-drop shaped, lengthening to slits with age. Ovary conical, glabrous; style ca. 7.5 mm long, straight, glabrous; stigma bilobed to bifid with the lobes ca. 0.2 mm long, the surfaces minutely papillate. Fruit a globose berry (described from Mustaqim et al. 2022), ca. 0.7 cm in diameter, green (immature?), the pericarp glabrous, matte, opaque; fruiting pedicels to 3 cm long, pendent, bright purple (dull violet) especially towards the enlarged apex, glabrous; fruiting calyx a cup subtending the fruit, tightly adhering to the basal part of the berry, the appendages fleshy and somewhat enlarged, slightly backwards pointing. Seeds and stone cells not seen. Chromosome number not known.

**Figure 55.**
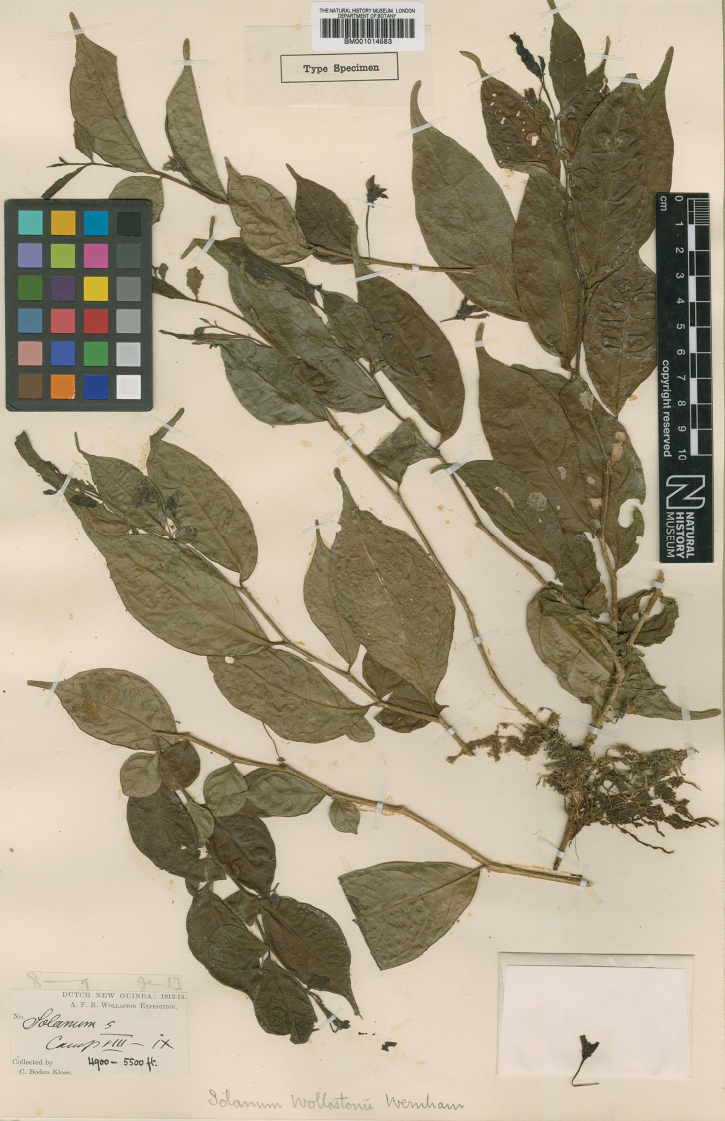
*Lyciantheswollastonii* herbarium specimen. Indonesia. Papua: *Kloss s.n.* (holotype of *S.wollastonii*, BM001014583). Courtesy of the Trustees of the Natural History Museum, London, reproduced with permission.

#### Distribution

**Figure 56.**
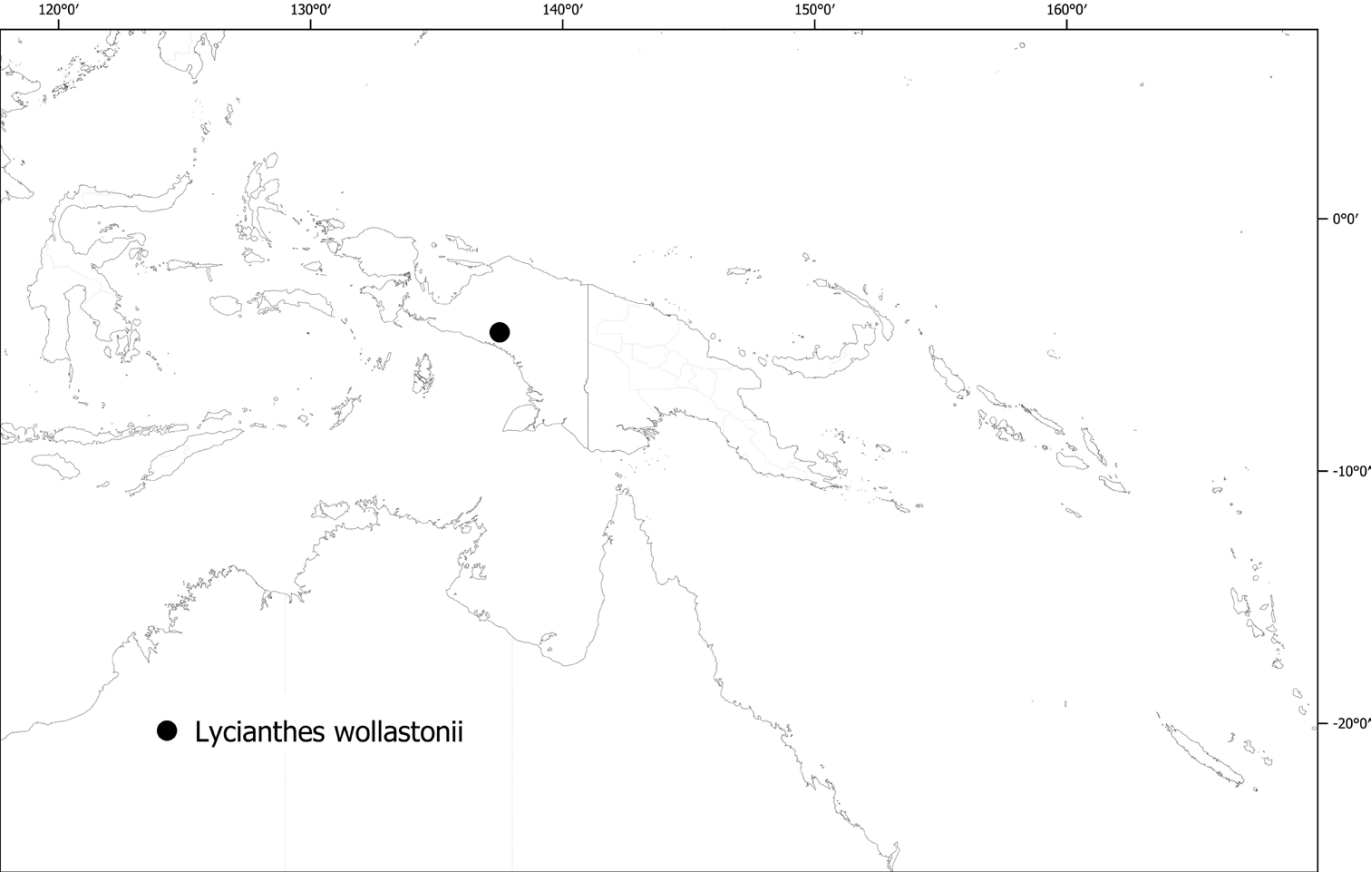
Distribution of *Lyciantheswollastonii*.

**(Fig. 56).***Lyciantheswollastonii* is endemic to the island of New Guinea; it is known only from Puncak Jaya (Mount Carstenz) in Indonesia (Papua).

#### Ecology and habitat.

*Lyciantheswollastonii* is a plant of mossy montane forests, growing between 1,500 and 1,800 m elevation.

#### Common names.

None recorded.

#### Preliminary conservation assessment

**(IUCN 2020).**EOO (0 km^2^ - CR); AOO (8 km^2^ - CR). *Lyciantheswollastonii* is known from a single locality. Mustaqim et al. (2022) assessed *L.wollastonii* as Endangered (EN B2ab(iii)) due to its small range in an area threatened by logging and mining. They did not assign a more threatened status due to the number of populations assumed to be two (their collection and the type), however, although the type locality is somewhat imprecise, it can be pinpointed to a small area using the description of the Wollaston expedition itinerary (Kloss 1916) and is thus distinct from the collection locality cited by Mustaqim et al. (2022). I consider the species should be assigned a threat status of Critically endangered (CR) due to its narrow distribution and the threats for human alteration of the environment. This species merits further collecting and population survey.

#### Discussion.

Until recently (Mustaqim et al. 2022) *Lyciantheswollastonii* was known only from Cecil Boden Kloss’s type collection, made on the expedition led by Arthur Wollaston to “Snow Mountains” (Kloss 1916). Their collection, made in 2018 on the ascent of Mount Jaya, is the first fruiting record for this species and confirms both its distinctness from other New Guinea *Lycianthes* and its rarity and restricted distribution. I have described the fruits and associated characters from Mustaqim et al. (2022); colour of mature fruits is still not known. Wernham (1916) described *L.wollastonii* (as *S.wollastonii*) as an epiphyte, but Mustaqim et al. (2022) described it as a slender shrub; Kloss could have mistaken the habit due to the dense covering of moss on the stems, or *L.wollastonii* may indeed sometimes grow on the branches of other plants in its wet cloud forest habitat as do other Solanaceae (e.g., *S.gonyrhachis* S.Knapp of Bolivia, see Knapp 2002).

*Lyciantheswollastonii* is somewhat like *L.lucens* in its glabrous leaves, triangular calyx appendages held perpendicular to the calyx tube (see figure 1 in Mustaqim et al. 2022) and large flowers. It differs from *L.lucens* in its homostylous (to be confirmed) flowers (heterostylous in *L.lucens*), anthers 4–4.5 mm long (versus 2–2.5 mm long in *L.lucens*) and minor leaves differing significantly in shape from the major leaves (similar in shape, but not in size in *L.lucens*).

The large flowers with narrow corolla lobes of *Lyciantheswollastonii* are somewhat similar to those of *L.rostellata*. The two species can be distinguished by stem pubescence (absent in *L.wollastonii*, of stiff, antrorse trichomes with multicellular bases in *L.rostellata*) and calyx appendage morphology (absent in *L.rostellata*, triangular in *L.wollastonii*).

#### Specimens examined.

Indonesia. **Papua**: Timika Regency, Mount Jaya, Tembagabura, Borobudur, 2,090 m, 21 Nov 2018, *Mustaqim & Manurang 2213* (BO).

##### ﻿Names (designations) not validly published

*Lycianthesamblycarpa* Bitter, not validly published; herbarium name in Bitter’s hand on undated annotation label on *Versteeg 1351* (L.2859655) = *L.impar*.

*Lycianthespliorhachis* Bitter, not validly published herbarium name in Bitter’s hand on undated annotation label on *Versteeg 1137* (L.2881336) = *L.impar*.

*Solanumperanomalum* Wernham, Trans. Linn. Soc. London, Bot. 9(1): 119. 1916, isonym of *S.peranomalum* Wernham ex Ridl. = *L.peranomala*.

SolanumurbanumMorongvar.typicum Chodat, Bull. Soc. Bot. Genève, ser. 2, 8: 151. 1916, not validly published (Art. 24.3, Turland et al. 2018). = *L.rantonnetii*.

## Supplementary Material

XML Treatment for Lycianthes

XML Treatment for Lycianthes bambusarum

XML Treatment for Lycianthes belensis

XML Treatment for Lycianthes biflora

XML Treatment for Lycianthes bitteriana

XML Treatment for Lycianthes cladotrichota

XML Treatment for Lycianthes dendropilosa

XML Treatment for Lycianthes impar

XML Treatment for Lycianthes kaernbachii

XML Treatment for Lycianthes lucens

XML Treatment for Lycianthes moszkowskii

XML Treatment for Lycianthes multifolia

XML Treatment for Lycianthes oliveriana

XML Treatment for Lycianthes peranomala

XML Treatment for Lycianthes rantonnetii

XML Treatment for Lycianthes rostellata

XML Treatment for Lycianthes shanesii

XML Treatment for Lycianthes vitiensis

XML Treatment for Lycianthes wollastonii

## ﻿Index to numbered collections (Australia, New Guinea and the Pacific only)

Only first collectors in collections from the area treated in this revision (Australia, New Guinea and the Pacific) made by two or more collectors are listed here. Collections by anonymous collectors without date or other identifying features are not listed. All collections seen are listed in this format in Suppl. material 1, and full collector strings can be found in the Supplementary Material searchable files (Suppl. material 2, 3). These files are also available on the NHM Data Portal (https://doi.org/10.5519/j1y41fl4).

Aët 395 (oliveriana); 407 (impar).

Andrews, C.W. 181 (biflora).

Argent, G.C.G. 8/19 (rostellata).

Balgooy, M.M.J. van 4916 (biflora).

Bates, R.J. 13861, 30884, 37051 (rantonnetii).

Bean, A.R. 3865, 5271, 16706 (shanesii).

Beer’s collectors BSIP-7225, BSIP-7306 (vitiensis).

Bola, I.S. 41 (vitiensis).

Bowers, N. 796 (rostellata).

Brass, L.J. 4135 (rostellata); 6796 (impar); 11223 (belensis); 12290 (moszkowskii); 12907 (multifolia); 23978 (biflora); 28494 (lucens); 30845 (cladotrichota); 32400 (belensis).

Bristol, M.L. 2146 (vitiensis).

Bryan, E.H. 173, 608 (vitiensis).

Burley, J.S. 2602 (biflora).

Carr, C.E. 11670 (oliveriana); 13611, 13737, 13738, 14357 (rostellata); 14991 (biflora); 15946 (cladotrichota); 15948 (oliveriana); 15965 (biflora); 16109 (cladotrichota); 16195 (peranomala).

Christophersen, E. 326, 2249, 2320, 2324 [a], 2354, 2518, 2521, 2528, 2697, 2895, 3116, 3123 (vitiensis).

Clarkson, J.R. 4217, 4585, 4586, 5131, 6674, 6768, 7813, 8205 (shanesii).

Clemens, J. 1426 (kaernbachii).

Clemens, M.S. 1289 (kaernbachii); 1821 (oliveriana), 9071 (moszkowskii).

Comins, R.B. 235 (vitiensis).

Conn, B.J. 142 (bambusarum); 444 (moszkowskii); 1756 (oliveriana).

Craven, L.A. 848 (oliveriana); 1258 (bambusarum).

Croft, J.R. NGF-34800, LAE-61993 (rostellata); LAE-71421 (lucens).

Cruttwell, N. 2310 (cladotrichota); 2602 (belensis).

Ctvrtecka, R. 1644 (peranomala).

Cumming, R.J. 15486, 15841 (shanesii).

Dallachy, J. 345 (shanesii).

Damas, D. SAJ-1050 (oliveriana).

Degener, O. 14832, 14875, 15032, 32083 (vitiensis).

Docters van Leeuwen, W.M. 9884 (oliveriana); 11108 (impar).

Dransfield, J. 6705 (biflora).

Fallen, M. 374 (bambusarum).

Fell, D.G. 3232, 4314, 4354, 10000 (shanesii).

Fensham, R.J. 579, 718, 749, 823 (shanesii).

Floyd, A.G. 7459, 7509 (biflora).

Forbes, H.O. 882 (biflora).

Ford, A. 2647 (shanesii).

Foreman, D.B. NGF-45698 (vitiensis); NGF-45764 (impar); LAE-60242 (biflora).

Forestry Department 965 (vitiensis).

Forster, P.I. 5108, 5808, 8012, 15280, 19385 (shanesii); 24067 (rantonnetii).

Frodin, D.G. 2352 (oliveriana).

Gafui, I. BSIP-16450, BSIP-16787, BISP-18862 (vitiensis).

Gibbs, L.S. 568 (vitiensis).

Gideon, O. LAE-57196 (lucens).

Gillespie, J.W. 2016, 3251, 4082, (vitiensis); 4446, 4517 (vitiensis).

Gjellerup, K. 613 (oliveriana).

Godwin, M. C-881, C-2416 (shanesii).

Graeffe, E. 1354 (vitiensis).

Gray, B. 4972, 4974, 4981, 6706, 8068 (shanesii).

Greenwood, W. 127, 1151 (vitiensis).

Guppy, H.B. 160 (vitiensis).

Hartley, T.G. 10065, 10136, 11428 (oliveriana); 11434, 11756 (bambusarum); 12523 a (moszkowskii).

Henty, E.E. NGF-29203 (biflora); NGF-41640 (rostellata); NGF-42805 (impar).

Höft, R. 29018 (moszkowskii).

Hollrung, M. 776 (oliveriana).

Hoogland, R.D. 3979 (cladotrichota); 7291 (dendropilosa).

Horne, J. 714 (vitiensis).

Hyland, B. 5222, 8713, 11071, 13764, 14694, 14781, 15119, 15534, 16345, 25551 RFK, 25640 RFK (shanesii).

Idjan 348 (oliveriana).

Isles, S. NGF-33899 (biflora).

Jago, R.L. 4014 (shanesii).

James, S.A. SAJ 1385 (cladotrichota).

Johns, R.J. 8900 (cladotrichota).

Johnstone, R.L. 3435 (rantonnetii).

Kairo, A. 62, 70, 73, 79 (moszkowskii); 10652, NGF-27869 (biflora); NGF-30943, NGF-30983 (kaernbachii).

Kajewski, S.F. 1800, 1863, 2388 (vitiensis).

Kalkman, C. BW-3479 (multifolia).

Kanrtawinana, K. 905 (biflora).

Katik, P. LAE-70928, LAE-70954 (lucens).

Kato, H. C-7273 (biflora).

Kere, F. BSIP-4936, BSIP-5057 (vitiensis).

Kerenga, K. LAE-77590 (moszkowskii); LAE-77644 (rostellata).

Kerrigan, R.A. 1343 (shanesii).

Kessler, P.J.A. 934 (biflora).

Kornassi 649 (oliveriana).

Koroiveibau, D. 12807 (vitiensis).

Kostermans, A.J.G.H. 2704 (oliveriana); 13995 (biflora).

Kuruveli, I.T. 13888, 14007 (vitiensis).

Lawson, E. 51 (rantonnetii).

Ledermann, C.L. 12606 (cladotrichota).

Ledua, M.K. 11018 (vitiensis).

MacKee, H.S. 2995 (vitiensis).

Mananduar, R.K. 6805 (biflora).

Mauriasi, R. BSIP-8491, BISP-13952, BSIP-14089, BSIP-14154 (vitiensis).

McDonald, W.J. 5862 (shanesii).

Mcdonald, W.J.F. 6674, 6768 (shanesii).

Meebold, A. 17036 (vitiensis).

Millar, A.N. NGF-11795 (biflora); NGF-18664 (rostellata); NGF-23260, NGF-23365 (kaernbachii); NGF-23549 (cladotrichota); NGF-23858 (oliveriana); NGF-40737 (rostellata).

Millar, H.N. NGF-11794 (biflora).

Molino, J.-.F. 3060 (oliveriana).

Moll, V.W. BW-9529 (biflora).

Mueller, F.J.H. von 13 (vitiensis).

Nakisi, A. BSIP-8013 (vitiensis).

Nicholson, D.I. AFO-4776 (shanesii).

Nicholson, N.J. NJN 455 (shanesii).

O’Shanesy, P.A. 6 ser. 1 (shanesii).

Parham, J.W. 13051 (vitiensis).

Pleyte, D.R. 623 (oliveriana).

Polak, A.M. 651, 864 (oliveriana).

Powell, D.A. 164 (biflora).

Powell, T. 365 (vitiensis).

Pullen, R. 6011 (moszkowskii).

Puradyatmika, P. 10428 (impar).

Qoro, I. USDA-14091 (vitiensis).

Raigiso, F.C. 3141 (vitiensis).

Ramlanto 869 (biflora).

Rau, K. 73 (moszkowskii), 380 (biflora).

Rechinger, K. 4398 (vitiensis).

Reinecke, F. 58, 58 a, 78 (vitiensis).

Ridley, H.N. 34 (biflora).

Riley, J.C. 45 (vitiensis).

Roberts, L.J. KRM-15160 (shanesii).

Royen, P. van 7621, 7716 (oliveriana); 11511, NGF-18229 (rostellata).

Sands, M.J.S. 1751 (moszkowskii); 1966, 2073, 2230 (lucens); 6431 (biflora); 6744, 6791 (biflora); 7329 (impar).

Sankowsky, G. 615 (shanesii).

Sayers, C.D. NGF-19830, NGF-21517 (moszkowskii).

Schlechter, F.R.R. 13748, 13749 a, 17305 (biflora); 17339 (kaernbachii); 17961, 18319, 18427,, 20256 (oliveriana).

Schodde, R. 4094 (vitiensis); 5705 (rostellata).

Schram, F.A.W. BW-10645, BW-10744 (biflora).

Seemann, B.C. 42, 340, 387 (vitiensis).

Shapcott, A. MGH-40 (shanesii).

Sidiyasa, K. 2124 A (biflora).

Smith, A.C. 574, 1244, 1508, 1826, 5250, 5270, 5501, 5828, 7343, 7797, 8082 8894, 8930, 9171, 9264 (vitiensis).

Sterly, J. 80-469 (belensis).

Stevens, P.F. LAE-54766 (bambusarum); LAE-58668 (biflora).

Streimann, H. 9635 (bambusarum); NGF-25853 (biflora); NGF-25854 (bitteriana); NGF-27634 (moszkowskii); NGF-34091 (cladotrichota); NGF-35924 (moszkowskii); LAE-51786 (impar); LAE-53892 (bambusarum).

Symon, D.E. 10631 (moszkowskii); 10632 (bambusarum); 10651 (bitteriana); 10655 (oliveriana); 10659 (oliveriana); 10676 (cladotrichota); 10677, 10678, 10687, 10690 (rostellata); 10691 (dendropilosa); 10704 (rostellata); 13822 (bambusarum); 13828 (moszkowskii); 13829 (bambusarum); 13830, 13846, 13854 (moszkowskii); 13874, 13875, 13876, 13877 (rostellata); 13896 (oliveriana).

Takeuchi, W.N. 9181 (impar); 9307 (oliveriana); 10663 (moszkowskii); 11204 (peranomala); 11704 (bambusarum); 11804 (rostellata); 12379 (cladotrichota); 12688, 16902 (biflora); 17743, 17786, 22112 (oliveriana); 22892 (cladotrichota); 23389, 23491, 23895 (oliveriana).

Teona, R. BISP-6362 (vitiensis).

Thomas, G. 8951 (shanesii).

Thompson, E.J. GLA-22 (shanesii).

Tothill, B.H. 556, 633, 636, 638, 639 (vitiensis).

Trethewy, A.W. 9 (rantonnetii).

Utteridge, T.M.A. 119 (impar); 287, 295 (oliveriana).

Vandenberg, J. NGF-39883 (rostellata).

Vaupel, F. 104 (vitiensis).

Vavryn, D. 17 (shanesii).

Vinas, A.N. 308 (kaernbachii); LAE-59477 (oliveriana).

Vink, W. 16847 (oliveriana).

Vogel, E.F. de 3565, 3718 (biflora).

Walsh, M.E. 99 (biflora).

Wannan, B.S. 2328 (shanesii).

Warburg, O. 21250 (biflora).

Waterhouse, B.M. 4806, 5114 (shanesii).

Waterhouse, J.H.L. 66, 272 -B (vitiensis).

Webster, G.L. 15188 (moszkowskii).

Wells, J. NGF-7565 (bitteriana); NGF-7569 (biflora).

Whistler, A. 4, 520, 891, 1071, 1090, 1253 (vitiensis).

Whitmee, S.J. 52, 185 [a] (vitiensis).

Whitmore’s collectors BSIP-3081 (vitiensis).

Whitmore, T.C. BSIP-776, BSIP-2437, BSIP-2567 (vitiensis).

Wiakabu, J. LAE-70476 (multifolia).

Womersley, J.S. 4883 (rostellata); NGF-37289 (oliveriana).

Worboys, S.J. 1344 (shanesii).

Yuncker, T.G. 16103 (vitiensis).

## Supplementary materials

Supplementary material 1 Index to all numbered collections in pdf format Data type Collections by collector and number (exsiccatae list) Explanation note An exsiccata index to all numbered collections seen for this mongraph (including those outside Australia, New Guinea and the Pacific); organised by primary collector only. Collections with no collection number are not included in this list. https://binary.pensoft.net/file/746706This dataset is made available under the Open Database License (http://opendatacommons.org/licenses/odbl/1.0/). The Open Database License (ODbL) is a license agreement intended to allow users to freely share, modify, and use this Dataset while maintaining this same freedom for others, provided that the original source and author(s) are credited.Sandra Knapp

Supplementary material 2 Searchable csv file of *Lycianthes* collections from Australia, New Guinea and the Pacific Data type Occurrences Explanation note A searchable csv file of all *Lycianthes* specimens seen for this monograph from Australia, New Guinea and the Pacific only. https://binary.pensoft.net/file/746707This dataset is made available under the Open Database License (http://opendatacommons.org/licenses/odbl/1.0/). The Open Database License (ODbL) is a license agreement intended to allow users to freely share, modify, and use this Dataset while maintaining this same freedom for others, provided that the original source and author(s) are credited.Sandra Knapp

Supplementary material 3 Searchable csv file of all *Lycianthes* collections examined for this revision Data type Occurrences Explanation note A searchable csv file of all *Lycianthes* specimens seen for this monograph including those from outside Australia, New Guinea and the Pacific (the region treated). https://binary.pensoft.net/file/746708This dataset is made available under the Open Database License (http://opendatacommons.org/licenses/odbl/1.0/). The Open Database License (ODbL) is a license agreement intended to allow users to freely share, modify, and use this Dataset while maintaining this same freedom for others, provided that the original source and author(s) are credited.Sandra Knapp

## References

[B1] Acosta MC Bernardello G Guerra M Moscone EA (2005) Karyotype analysis in several South American species of *Solanum* and *Lycianthesrantonnei* (Solanaceae). Taxon 54(3): 713–723. 10.2307/25065428

[B2] Anderson GJ (1979) Dioecious *Solanum* of hermaphroditic origin is an example of broad convergence. Nature 282(5741): 836–838. 10.1038/282836a0

[B3] Anderson GJ Symon DE (1989) Functional dioecy and andromonoecy in *Solanum*. Evolution. International Journal of Organic Evolution 43(1): 204–219. 10.1111/j.1558-5646.1989.tb04218.x 28568500

[B4] Anderson GJ Anderson MKJ Patel N (2015) The ecology, evolution, and biogeography of dioecy in the genus *Solanum*: With paradigms from the strong dioecy in *Solanumpolygamum*, to the unsuspected and cryptic dioecy in *Solanumconocarpum*. American Journal of Botany 102(3): 471–486. 10.3732/ajb.1400486 25784480

[B5] Aubriot X Knapp S (2022) A revision of the “spiny solanums” of tropical Asia (Solanum, the Leptostemonum Clade, Solanaceae). PhytoKeys 198: 1–270. 10.3897/phytokeys.198.79514 PMC9849010 36760991

[B6] Bachmann S Moat J Hill AW de la Torre J Scott B (2011) Supporting Red List assessments with GeoCAT: Geospatial conservation assessment tool. ZooKeys 150: 117–126. 10.3897/zookeys.150.2109 PMC3234434 22207809

[B7] Barboza GE (2013) *Lycianthes*. In: Anton AM Zuloaga FO (Eds) Barboza GE (coord.) Flora Argentina vol. 13, Solanaceae. IOBDA- IMBIV, CONICET: Buenos Aires & Córdoba, Argentina, 25–30.

[B8] Bean AR (2003) New combinations in *Lycianthes* (Dunal) Hassl. (Solanaceae) for New Guinea and Australia. Austrobaileya 6(3): 567–569.

[B9] Bitter G (1911) Steinzellkonkretionen im Fruchtfleisch beerentragender Solanaceen und deren systematische Bedeutung. Botanische Jahrbücher für Systematik, Pflanzengeschichte und Pflanzengeographie 45: 483–507.

[B10] Bitter G (1914) Weitere Untersuchungen über das Vorkommen von Steinzellkonkretionen im Fruchtfleisch beerentragender Solanaceen. Abhandlungen des Naturwissenschaftlichen Vereins zu Bremen 23: 114–163.

[B11] Bitter G (1917) Die papuasischen Arten von *Solanum*. Botanische Jahrbücher für Systematik, Pflanzengeschichte und Pflanzengeographie 55: 59–113.

[B12] Bitter G (1919) Die Gattung *Lycianthes*. Abhandlungen herausgegeben vom Naturwissenschaftlichen Vereins zu Bremen [preprint] 24: 292–520.

[B13] Bridson GDR (2004) BPH-2: Periodicals with Botanical Content. Hunt Institute for Botanical Documentation, Pittsburgh. http://fmhibd.library.cmu.edu/fmi/iwp/cgi?-db=BPH_Online&-loadframes

[B14] Buchmann SL Jones CE Colin LJ (1977) Vibratile pollination of *Solanumdouglasii* and *S.xanti* (Solanaceae) in southern California. Wasmann Journal of Biology 35: 1–25.

[B15] Cámara-Leret R Frodin DC Adema F Anderson C Appelhans MS Argent G Arias Guerrero S Ashton P Baker W Barfod AS Barrington D Borosova R Bramley GLC Briggs M Buerki S Cahen D Callmander MW Cheek M Chen C-W Conn B Coode MJE Darbyshire I Dawson S Dransfield J Drinkell C Duyjfies B Ebihara A Ezedin Z Fu L-F Heatubun CD Hind DJN Hoch P Hovenkamp P Hughes M Jebb M Jennings L Jimbo T Kessler M Kiew R Knapp S Lehnert M Lewis GP Linder HP Lindsay S Low YW Lucas E Mancera JP Monro AK Moore A Middleton DJ Nagamusu H Newman MF Nic Lughadha E Melo PHA Ohlsen D Pannell CM Parris B Pearce L Penneys D Perrie LR Petoe P Poulsen AD Prance GT Quakenbush JP Raes N Rodda M Rogers ZS Schuiteman A Schwartsburd P Scotland RW Simmons MP Simpson DA Stevens PF Sundue M Testo W Trias-Blasi A Turner I Utteridge T Walsingham L Webber BL Wei R Weiblen G Weigend M Weston P de Wilde W Wilkie P Wilmot-Dear CM Wilson HP Wood JRI Zhang L-B van Welzen P (2020) New Guinea has the world’s richest island flora. Nature 584(7822): 579–583. 10.1038/s41586-020-2549-5 32760001

[B16] Carrière E-A (1859) Solanumrantonnei. Revue Horticole 1859: 155–158.

[B17] Chodat RH (1916) II. Solanées. II. B. Géobotanique et étude critique de quelques Solanacées paraguayennes. Bulletin de la Sociedad Botanique de Genève, series 2 8: 142–160.

[B18] Cracraft J (1989) Speciation and its ontogeny: the empirical consequences of alternative species concepts for understanding patterns of differentiation. In: Otte D Endler JA (Eds) Speciation and its consequences.Sinauer and Associates, New York, 28–59.

[B19] Danert S (1958) Die Verzweigung der Solanaceen im reproduktiven Bereich. Abhandlungen der Deutschen Akademie der Wissenschaften zu Berlin, Klasse für Chemie, Geologie und Biologie 1957: 1–183.

[B20] D’Arcy WG (1972) Solanaceae studies II: Typification of subdivisions of *Solanum*. Annals of the Missouri Botanical Garden 59(2): 262–278. 10.2307/2394758

[B21] D’Arcy WG (1974) *Solanum* and its close relatives in Florida. Annals of the Missouri Botanical Garden 61(3): 819–867. 10.2307/2395032

[B22] D’Arcy WG (1986) The calyx in *Lycianthes* and some other genera. Annals of the Missouri Botanical Garden 73(1): 117–127. 10.2307/2399143

[B23] D’Arcy WG Keating RC Buchmann SL (1996) The calcium oxalate package or so-called resorption tissue in some angiosperm anthers. In: D’Arcy WG Keating RC (Eds) The anther: form, function and phylogeny.Cambridge University Press, Cambridge, 159–191.

[B24] Davis JI (1997) Evolution, evidence, and the role of species concepts in phylogenetics. Systematic Botany 22(2): 373–403. 10.2307/2419463

[B25] Davis PH Heywood VH (1963) Principles of angiosperm taxonomy. Van Nostrand, New York.

[B26] Dean EA (2001) The post-anthesis floral biology of *Lycianthes* series *Meizonodontae* (Solanaceae): variation in filament elongation, anther dehiscence, floral movement and corolla growth. In: van den Berg RG Barendse GWM van der Weerden GM Mariani C (Eds) Solanaceae V; advances in taxonomy and utilization.Nijmegen University Press, Nijmegen, 137–151.

[B27] Dean E (2004) A taxonomic revision of *Lycianthes* series *Meizonodontae* (Solanaceae). Botanical Journal of the Linnean Society 145(4): 385–424. 10.1111/j.1095-8339.2004.00296.x

[B28] Dean E Poore J Anguiano-Constante MA Nee MH Kang H Starbuck T Rodrígues A Conner M (2020) The genus *Lycianthes* (Solancaeae, Capsiceae) in Mexico and Guatemala. PhytoKeys 168: 1–333. 10.3897/phytokeys.168.51904 33335445 PMC7718216

[B29] Diamond J Bishop KD (2021) Avifauna of the Van Rees Mountains, New Guinea. Bulletin of the British Ornithologists’ Club 141(4): 446–469. 10.25226/bboc.v141i4.2021.a8

[B30] Dunal M-F (1813) Histoire naturelle, médicale et économique des *Solanum* et des genres qui ont été confundus avec eux. Renaud, Montpellier. 10.5962/bhl.title.164866

[B31] Dunal M-F (1816) Solanorum generumque affinium synopsis. Renaud, Montpellier.

[B32] Dunal M-F (1852) Solanaceae. In: de Candolle AP (Ed.) Prodromus systematis naturalis regni vegetabilis.V. Masson, Paris 13(1), 1–690.

[B33] Endress PK (1996) Diversity and evolutionary trends in angiosperm anthers. In: D’Arcy WG Keating RC (Eds) The anther: form, function and phylogeny.Cambridge University Press, Cambridge, 92–110.

[B34] Hassler E (1917) Solanaceae Austro-Americanae imprimis Paraguariensis. Annuaire du Conservatoire et du Jardin Botaniques de Genève 20: 173–183.

[B35] Hul S Dy Phon P (2014) Solanaceae. In: Aubréville A Leroy J Morat P (Eds) Flore du Cambodge, du Laos et du Vietnam.Muséum National d’Histoire Naturelle & Royal Botanic Garden Edinburgh, Paris & Edinburgh, 1–93.

[B36] Hunziker AT (1979) South American Solanaceae: a synoptic survey. In: Hawkes J Lester RN Skelding AD (Eds) The Biology and Taxonomy of the Solanaceae.The Linnean Society of London, Academic Press, London, 49–85.

[B37] Hunziker AT (2001) Genera Solanacearum: the genera of Solanaceae illustrated, arranged According to a new system. A. R. G. Gantner, Ruggell, Liechtenstein.

[B38] IUCN (2020) Guidelines for Using the IUCN Red List Categories and Criteria. Version 13. Prepared by the Standards and Petitions Subcommittee of the IUCN Species Survival Commission. http://www.iucnredlist.org/documents/RedListGuidelines.pdf

[B39] Kloss CB (1916) Itinerary. In: Ridley HN (Ed.) Report on the botany of the Wollaston expedition to Dutch New Guinea, 1912–1913.Transactions of the Linnean Society of London 9: 2–6. 10.1111/j.1095-8339.1916.tb00009.x

[B40] Knapp S (2001) Is morphology dead in *Solanum* taxonomy? In: van den Berg RG Barendse GWM van der Weerden GM Mariani C (Eds) Solanaceae V: advances in taxonomy and utilization.Nijmegen University Press, Nijmegen, 23–38.

[B41] Knapp S (2002) SolanumsectionGeminata (G. Don) Walpers (Solanaceae). Flora Neotropica 84: 1–405.

[B42] Knapp S (2008) Species concepts and floras: What are species for? Biological Journal of the Linnean Society. Linnean Society of London 95(1): 17–25. 10.1111/j.1095-8312.2008.01090.x

[B43] Knapp S (2013) A revision of the Dulcamaroid clade of *Solanum* L. (Solanaceae). PhytoKeys 22(0): 1–432. 10.3897/phytokeys.22.4041 PMC3689140 23794937

[B44] Knapp S Helgason T (1997) A revision of SolanumsectionPteroidea: Solanaceae. Bulletin of the Natural History Museum. London (Botany) 27: 31–73.

[B45] Knapp S Vorontsova MS (2016) A revision of the “African Non-Spiny” Clade of *Solanum* L. (*Solanum* sections *Afrosolanum* Bitter, *Benderianum* Bitter, *Lemurisolanum* Bitter, *Lyciosolanum* Bitter, *Macronesiotes* Bitter, and *Quadrangulare* Bitter: Solanaceae). PhytoKeys 66: 1–142. 10.3897/phytokeys.66.8457 PMC4957028 27489494

[B46] Knapp S Persson V Blackmore S (1998) Pollen morphology and functional dioecy in *Solanum* (Solanaceae). Plant Systematics and Evolution 210(1–2): 113–139. 10.1007/BF00984731

[B47] Knapp S Sagona E Carbonell AKZ Chiarini F (2017) A revision of the *Solanumelaeagnifolium* clade (Elaeagnifolium clade, subgenus Leptostemonum, Solanaceae). PhytoKeys 84: 1–104. 10.3897/phytokeys.84.12695 PMC5624188 29033654

[B48] Knapp S Barboza GE Bohs L Särkinen T (2019) A revision of the Morelloid Clade of *Solanum* L. (Solanaceae) in the Caribbean and North and Central America. PhytoKeys 123: 1–174. 10.3897/phytokeys.123.31738 31198402 PMC6554266

[B49] Ledermann C (1919) Einiges von der Kaiserin-Augusta-Fluβ-Expedition. Botanische Jahrbücher für Systematik, Pflanzengeschichte und Pflanzengeographie 55: 33–44.

[B50] Lester RN Durrands P (1984) Enzyme treatment as an aid in the study of seed surface structures of *Solanum* species. Annals of Botany 53: 129–131. 10.1093/oxfordjournals.aob.a086662

[B51] Loureiro J de (1790) Flora cochinchinensis. Royal Portuguese Academy of Sciences, Lisbon.

[B52] Luckow M (1995) Species concepts: assumptions, methods and applications. Systematic Botany 20: 589–605. 10.2307/2419812

[B53] Mallet J (1995) A species definition for the modern synthesis. Trends in Ecology & Evolution 10(7): 294–299. 10.1016/0169-5347(95)90031-4 21237047

[B54] Martine CT Anderson GJ Les DH (2009) Gender-bending aubergines: Molecular phylogenetics of cryptically dioecious *Solanum* in Australia. Australian Systematic Botany 22(2): 107–120. 10.1071/SB07039

[B55] Mayr E (1982) The growth of biological thought. Harvard University Press, Cambridge.

[B56] McDonnell AJ Wetreich HB Cantley JT Jobson P Martine CT (2019) *Solanumplastisexum*, and enigmatic new bush tomato from the Australian Monsson Tropics exhibiting breeding system fluidity. PhytoKeys 124: 39–55. 10.3897/phytokeys.124.33526 31258372 PMC6592974

[B57] McNeill J (2014) Holotype specimens and type citations: General issues. Taxon 63(5): 1112–1113. 10.12705/635.7

[B58] Merrill ED (1935) Loureiro and his botanical work. Proceedings of the American Philosophical Society 72(4): 229–239.

[B59] Merrill ED Perry LM (1939) PlantaePapuanaeArchboldianae I. Journal of the Arnold Arboretum 20(3): 324–345. 10.5962/p.325783

[B60] Merrill ED Perry LM (1949) PlantaePapuanaeArchboldianae XVIII. Journal of the Arnold Arboretum 30(1): 39–63. 10.5962/p.185601

[B61] Morton C (1976) A Revision of the Argentine Species of *Solanum*. Academia Nacional de Ciencias, Córdoba, Argentina.

[B62] Mustaqim WA Puradyatmika P Heatubun CD (2022) Solanaceae of New Guinea: Recollection and conservation status assessments of two endemic and poorly known species including update taxonomic descriptions. Rheedea 32(1): 46–54. 10.22244/rheedea.2022.32.01.04

[B63] Orejuela A (2021) Evolution of epiphytism in Solanaceae. PhD thesis, University of Edinburgh/Royal Botanic Garden Edinburgh, Edinburgh, United Kingdom.

[B64] Parsch C Wagner B Pangau-Adam M Nitschke C Kreft H Schrader J (2022) Papua at the crossroads: A plea for systematic conservation planning in one of the largest remaining areas of tropical rainforest. Frontiers in Forests and Global Change 5: 763131. 10.3389/ffgc.2022.763131

[B65] Peralta IE Spooner DM Knapp S (2008) Taxonomy of wild tomatoes and their relatives (Solanumsect.Lycopersicoides, sect. Juglandifolia, sect. Lycopersicon; Solanaceae). Systeamtic Botany Monographs 84: 1–186.

[B66] Plowman TC (1998) A revision of the South American species of *Brunfelsia* (Solanaceae). Fieldiana, Botany n.s. 39: 1–135.

[B67] Prado J Hirai RY Moran RC (2015) (046–048) Proposals concerning inadvertent lectotypifications (and neotypifications). Taxon 64(3): 651. 10.12705/643.29

[B68] Reinecke F (1898) Die Flora der Samoa-Inseln. Botanische Jahrbücher für Systematik, Pflanzengeschichte und Pflanzengeographie 25: 578–708.

[B69] Ridley HN (1916) Report on the botany of the Wollaston expedition to Dutch New Guinea, 1912–1913. Transactions of the Linnean Society of London 9, ser. 2: 1–269. 10.1111/j.1095-8339.1916.tb00009.x

[B70] Ridley HN (1922) *Solanumperanomalum*. Hooker’s Icones Plantarum 31(pt. 3): tab. 3062.

[B71] Roe KE (1966) Juvenile forms in *Solanummitlense* and *S.blodgettii* (Solanaceae) and their importance in taxonomy. Sida 2: 381–385.

[B72] Särkinen T Olmstead RG Bohs L Knapp S (2013) A phylogenetic framework for evolutionary study of the nightshades (Solanaceae): A dated 1000-tip tree. BMC Evolutionary Biology 13(1): e214. 10.1186/1471-2148-13-214 PMC3850475 24283922

[B73] Särkinen T Poczai P Barboza GE van der Weerden GM Baden M Knapp S (2018) A revision of the Old World black nightshades (Morelloid clade of *Solanum* L., Solanaceae). PhytoKeys 106: 1–223. 10.3897/phytokeys.106.21991 PMC6070582 30072843

[B74] Schumann K Lauterbach K (1900 [1901]) Die Flora der Deutschen Schutzgebeite in der Südsee. Gebrüder Borntraeger, Leipzig. [volume dated 1901, published November 1900]

[B75] Seemann B (1863) The Solana of tropical Polynesia. Journal of Botany 1: 206–211.

[B76] Seemann B (1865–1873) Flora Vitiensis. Vols. 1–10. L. Reeve and Company, London.

[B77] Seithe A (1962) Die Haararten der Gattung *Solanum* L. und ihre taxonomische Verwertung. Botanische Jahrbücher für Systematik, Pflanzengeschichte und Pflanzengeographie 81: 261–336.

[B78] Seithe A (1979) Hair types as taxonomic characters in *Solanum*. In: Hawkes JG, Lester RN, Skelding AD (Eds) The biology and taxonomy of the Solanaceae, Academic Press, London, 307–319.

[B79] Smith AC (1991) Solancaeae. In: Flora Vitensis Nova: a new flora of Fiji. Pacific Tropical Botanica Garden, Lawaii, 4–40.

[B80] Souèges R (1907) Développement et structure de tégument seminal chez les Solanacées. Annales des Sciences Naturelles, Botanique, séries 9, 6: 1–124.

[B81] Spalink D Stoffel K Walden GK Hulse-Kemp AM Hill TA Van Deynze A Bohs L (2018) Comparative transcriptomics and genomic patterns of discordance in Capsiceae (Solanaceae). Molecular Phylogenetics and Evolution 126: 293–302. 10.1016/j.ympev.2018.04.030 29702214

[B82] Stearn WT (1981) Herbert Fuller Wernham (1879–1941), a centenary commemoration. Taxon 30(1): 1–6. 10.2307/1219381

[B83] Symon DE (1970) Dioecious *Solanums*. Taxon 19(6): 909–910. 10.2307/1218308

[B84] Symon DE (1979) Sex forms in *Solanum* (Solanaceae) and the role of pollen collecting insects. In: Hawkes JD Lester RN Skelding AD (Eds) The biology and taxonomy of the Solanaceae.Academic Press, London, 385–397.

[B85] Symon DE (1981a) A revision of *Solanum* in Australia. Journal of the Adelaide Botanic Gardens 4: 1–367.

[B86] Symon DE (1981b) The Solanaceous genera, *Browallia*, *Capsicum*, *Cestrum*, *Cyphomandra*, *Hyoscyamus*, *Lycopersicon*, *Nierembergia*, *Physalis*, *Petunia*, *Salpichroa* and *Withania*, naturalised in Australia. Journal of the Adelaide Botanic Gardens 3: 133–166.

[B87] Symon DE (1984a) 313. Lycianthessubg.Cypellocalyx (Bitt.) Bitt. In: van Balgooy MMJ (Ed.) Pacific Plant Areas 4.Rijksherbarium, Leiden, 246–247.

[B88] Symon DE (1984b) 314. Lycianthessubg.Polymerissect.Asiomelanesia Bitt. In: van Balgooy MMJ (Ed.) Pacific Plant Areas 4.Rijksherbarium, Leiden, 248–249.

[B89] Symon DE (1985) The Solanaceae of New Guinea. Journal of the Adelaide Botanic Gardens 8: 1–171.

[B90] Symon DE (1986) The phytogeography of New Guinea *Solanum* (Solanaceae). Blumea 31: 319–328.

[B91] Symon DE (1994) Kangaroo apples; Solanumsect.Archaeosolanum. Published by the author, Adelaide.

[B92] Symon DE Clarkson JR (1985) The reinstatement of *Solanumshanesii* F. Muell. section Lycianthes (Solanaceae) with a discussion of its significance. Journal of the Adelaide Botanic Gardens 7(2): 201–206.

[B93] Takeuchi W Golman M (2002) The present status of Ledermann’s April River localities in Papua New Guinea. Sida 20: 55–70.

[B94] Turland NJ Wiersema JH Barrie FR Greuter W Hawksworth DL Herendeen PS Knapp S Kusber W-H Li D-Z Marhold K May TW McNeill J Monro AM Prado J Price MJ Smith GF (2018) International Code of Nomenclature for algae, fungi, and plants (Shenzhen Code). Regnum Vegetabile 159. Koeltz Botanical Books, Glashütten. 10.12705/Code.2018

[B95] Vallejo-Marín M Da Silva EM Sargent RD Barrett SCH (2010) Trait correlates and functional significance of heteranthery in flowering plants. The New Phytologist 188(2): 418–425. 10.1111/j.1469-8137.2010.03430.x 20819173

[B96] van Steenis-Kruseman MJ (1985) Malaysian plant collectors and collections, being a cyclopedia of botanical exploration in Malaysia. [originally published 1950 in Djakarta]. Martinius Nijhoff (Kluwer Academic Publishers), Boston, Dordrecht, Lancaster.

[B97] Veldkamp JF Vink W Frodin DG (1988) XI. Ledermann’s and some other German localities in Papua New Guinea. Flora Malesiana Bulletin 10: 32–38.

[B98] Vorontsova MS Knapp S (2010) Lost Berlin (B) types of *Solanum* (Solanaceae) – found in Göttingen (GOET). Taxon 59(5): 1585–1601. 10.1002/tax.595024

[B99] Vorontsova MS Knapp S (2016) A revision of the “spiny solanums,” SolanumsubgenusLeptostemonum (Solanaceae), in Africa and Madagascar. Systematic Botany Monographs 99: 1–428. 10.1007/BF03027161

[B100] Warburg O (1891) Beiträge zur Kenntnis der papuanischen Flora. Botanische Jahrbücher fur Systematik, Pflanzengeschichte und Pflanzengeographie 13: 230–455.

[B101] Wernham HF (1916) Solanaceae. In: Ridley HN (Ed.) Report on the botany of the Wollaston expedition to Dutch New Guinea, 1912–1913.Transactions of the Linnean Society of London 9: 119–120.

[B102] Wight R (1850) Icones plantarum Indiae orientalis; or Figures of Indian plants. Vol. IV. Frank and Co., Madras. [Chennai]

[B103] Witasek J (1908) Solani generis species et varietates novae. Repertorium Specierum Novarum Regni Vegetabilis 5(7–12): 163–166. 10.1002/fedr.19080050726

