# Supplementary material for: ﻿A revision of Lycianthes (Solanaceae) in Australia, New Guinea, and the Pacific
Source: PhytoKeys. 2022 Sep 23;209:1–134. doi: 10.3897/phytokeys.209.87681 (PMC9848948; doi:10.3897/phytokeys.209.87681)
Supplement: Supplementary material 1 — Index to all numbered collections in pdf format [file phytokeys-209-001_article-87681__-s001.pdf]

Supplementary Material 1: Index to all numbered collections

Aban Gibot SAN-66896 (biflora); SAN-81013 (biflora); SAN-94332 (biflora).
Aët 395 (oliveriana); 407 (impar).
Afriastini, J.J. 479 (biflora); 755 (biflora); 2859 (biflora).
Alston, A.H.G. 12898 (biflora); 14180 (biflora); 15100 (biflora); 15692 (biflora); 16218 (biflora).
Amano, T. 6961 (biflora).
Amin, A. SAN-123497 (biflora).
Amin, G. SAN-93928 (biflora).
Andau, D. 1118 (biflora).
Anderson, T. 303 (biflora); 364 (biflora); 365 (biflora); 1019 (biflora).
Anderson, W.R. 9139 (rantonnetii); 9186 (rantonnetii).
Andrews, C.W. 181 (biflora).
Andrews, L.M. 137 (rantonnetii).
Argent, G.C.G. 8/ 19 (rostellata).
Armstrong, K. 1036 (biflora); 1105 (biflora); 2187 (biflora); 2966 (biflora).
Ashton, P.S. S. 19162 (biflora).
Aung, M.M. 92543 (biflora); 92584 (biflora).
Averyanov, L. CBL-547 (biflora); VR-981 (biflora).
Backer, C.A. 3654 (biflora); 4845 (biflora).
Bakia, A. 612 (biflora).
Balgooy, M.M.J. van 215 (biflora); 3556 (biflora); 3594 (biflora); 4916 (biflora).
Barboza, G.E. 472 (rantonnetii); 486 (rantonnetii); 825 (rantonnetii); 1033 (rantonnetii); 1697 (rantonnetii); 2098 (rantonnetii).
Bartlett, H.H. 8215 (biflora); 8566 (biflora); 8567 (biflora); 8624 (biflora).
Bates, R.J. 13861 (rantonnetii); 30884 (rantonnetii); 37051 (rantonnetii).
Beaman, J.H. 7195 (biflora); 8636 (biflora); 9102 (biflora); 9202 (biflora); 9554 (biflora); 11175 (biflora); 11321 (biflora).
Bean, A.R. 3865 (shanesii); 5271 (shanesii); 16706 (shanesii).
Beccari, O. 119 (biflora); 120 (biflora).
Beddome, R.H. 5479 (biflora); 5484 (biflora); 5486 (biflora).
Beer's collectors BSIP-7225 (vitiensis); BSIP-7306 (vitiensis).
Berendsohn, W. WB 31 (rantonnetii).
Berhaman, A. 98 (biflora).
Beusekom, C.F. van 1360 (biflora); 3773 (biflora).
Biswas, K. 4578 (biflora).
Bodinier, E.M. 485 (biflora); 799 (biflora).

Boehmer, L. 112 (biflora).

Bola, I.S. 41 (vitiensis).

Borthwick, H.A. 1172 (rantonnetii).

Bourell, M. 2317 (biflora).

Bowers, N. 796 (rostellata).

Brass, L.J. 4135 (rostellata); 6796 (impar); 11223 (belensis); 12290 (moszkowskii); 12907 (multifolia); 23978 (biflora); 28494 (lucens); 30845 (cladotrichota); 32400 (belensis).

Bristol, M.L. 2146 (vitiensis).

Brooke, W.M.A. 10701 (biflora).

Bryan, E.H. 173 (vitiensis); 608 (vitiensis).

Búgola Silva, F. 8024 (rantonnetii).

Bunnemeyer, H.A.B. 4738 (biflora).

Burkill, I.H. 37463 (biflora).

Burley, J.S. 2602 (biflora); 3611 (biflora); 3628 (biflora).

Canton Christian College 1652 (biflora); 1882 (biflora); 3131 (biflora); 8268 (biflora); 12648 (biflora).

Carr, C.E. 11670 (oliveriana); 13611 (rostellata); 13737 (rostellata); 13738 (rostellata); 14357 (rostellata); 14991 (biflora); 15946 (cladotrichota); 15948 (oliveriana); 15965 (biflora); 16109 (cladotrichota); 16195 (peranomala).

Cavalerie, P.J. 1360 (biflora).

Chagas, F. 1391 (rantonnetii).

Chai, P. S. 37360 (biflora).

Chamchumroon, V. VC-5544 (biflora).

Chantaranothai, P. 90/69 (biflora).

Chatterjee's collector 118 (biflora).

Chatterjee, D. 118 (biflora).

Chew, W.L. RSNB-126 (biflora); 637 (biflora); RSNB-1247 (biflora); 1278 (biflora); RSNB-4025 (biflora); RSNB-4707 (biflora).

Chiarini, F. 876 (rantonnetii); 918 (rantonnetii).

Ching, R.C. 1948 (biflora); 5317 (biflora); 6684 (biflora).

Chodat, R.H. 60 (rantonnetii); 61 (rantonnetii).

Chow, C.L. 6772 (biflora).

Chow, K.S. 79100 (biflora); 80151 (biflora).

Christensen, H. 71 (biflora).

Christophersen, E. 326 (vitiensis); 2249 (vitiensis); 2320 (vitiensis); 2324 [a] (vitiensis); 2354 (vitiensis); 2518 (vitiensis); 2521 (vitiensis); 2528 (vitiensis); 2697 (vitiensis); 2895 (vitiensis); 3116 (vitiensis); 3123 (vitiensis).

Chun, N.K. 41918 (biflora); 42333 (biflora).

Chun, W.Y. 6764 (biflora); 6764 [b] (biflora).

Chung, H.H. 6743 (biflora); 8025 (biflora).

Clarke, C.B. 8830 B (biflora); 10956 (biflora); 12123 (biflora); 12198 (biflora); 15780 A (biflora); 18911 (biflora); 18934 (biflora); 26473 A (biflora); 26728 D (biflora); 35334 A (biflora); 43843 (biflora); 44136 E (biflora).

Clarkson, J.R. 4217 (shanesii); 4585 (shanesii); 4586 (shanesii); 5131 (shanesii); 6674 (shanesii); 6768 (shanesii); 7813 (shanesii); 8205 (shanesii).

Clemens, J. 1426 (kaernbachii); 20171 (biflora); 20624 (biflora); 22188 (biflora); 26049 (biflora); 26119 (biflora); 26139 (biflora); 28470 (biflora); 28479 (biflora); 31879 (biflora); 32965 (biflora); 40167 (biflora); 40315 (biflora); 50070 (biflora).

Clemens, M.S. 1289 (kaernbachii); 1821 (oliveriana); 16471 (biflora).

Comins, R.B. 235 (vitiensis).

Conn, B.J. 142 (bambusarum); 444 (moszkowskii); 1756 (oliveriana).

Cooper, R.E. 467 (biflora); 4466 (biflora); 4710 (biflora); 5941 A (biflora); 6130 (biflora).

Covile, D.P.M. 6729 (biflora).

Craib, W.G. 66 (biflora).

Craven, L.A. 848 (oliveriana); 1258 (bambusarum).

Creech, J.L. 599 (biflora).

Croft, J.R. NGF-34800 (rostellata); LAE-61993 (rostellata); LAE-71421 (lucens).

Cruttwell, N. 2310 (cladotrichota); 2602 (belensis).

Ctvrtecka, R. 1644 (peranomala).

Cumming, R.J. 15486 (shanesii); 15841 (shanesii).

Cuong, N.M. 47 (biflora); 662 (biflora).

Curran, H.M. 16203 (biflora).

D'Arcy, W.G. 19272 (biflora).

Dallachy, J. 345 (shanesii).

Damas, D. SAJ-1050 (oliveriana).

Dastur, R.H. 40 (rantonnetii).

Davis, P.H. 69213 (biflora).

Dean, E. 250 (rantonnetii); 400 (rantonnetii).

Deanna, R. 195 (rantonnetii).

Deb, D.B. 746 (biflora); 859 (biflora); 26139 (biflora); 26406 (biflora); 27174 (biflora).

Degener, O. 14832 (vitiensis); 14875 (vitiensis); 15032 (vitiensis); 32083 (vitiensis).

Docters van Leeuwen, W.M. 9884 (oliveriana); 11108 (impar).

Dransfield, J. 6329 (biflora); 6705 (biflora).

Du, H.B. HNK-2950 (biflora).

Du, N.V. HNK-2798 (biflora).

Dulong Jiang Investigation Team 230 (biflora); 387 (biflora).

Dunn, S.T. 3344 (biflora).

Duyag, A. 76666 (biflora).

East India Company 5903 (biflora).

Edaño, G.E. 716 (biflora); 852 (biflora).

Elbert, J. 2956 (biflora).

Elmer, A.D.E. 13828 (biflora); 13881 (biflora); 17592 (biflora); 20597 (biflora).

Erwin, ? S. 27459 (biflora).

Esquirol, J.H. 176 (biflora); 523 (biflora); 544 (biflora); 3662 (biflora).

Everett, B. FRI-13774 (biflora).

Expeditio Biologica Sino-Rossica 1955 1236 (biflora).

Fallen, M. 374 (bambusarum).

Fang, W.P. 2569 (biflora); 15748 (biflora); 17850 (biflora).

Faurie, U. 318 (biflora); 321 (biflora); 324 (biflora); 641 (biflora); 918 [a] (biflora); 1192 (biflora); 1481 (biflora); 4104 (biflora).

Fell, D.G. 3232 (shanesii); 4314 (shanesii); 4354 (shanesii); 10000 (shanesii).

Feng, K.M. 3061 (biflora); 11392 (biflora); 12923 (biflora).

Fénix, E. 3838 (biflora).

Fensham, R.J. 579 (shanesii); 718 (shanesii); 749 (shanesii); 823 (shanesii).

Field, W.D. 21 y (biflora); 42 (biflora).

Fischer, C.E.C. 417 (biflora); 2741 (biflora).

Floto, F. 7615 (biflora).

Floyd, A.G. 7459 (biflora); 7509 (biflora).

Forbes, H.O. 507 (biflora); 794 (biflora); 882 (biflora); 949 (biflora); 974 a (biflora); 995 (biflora); 1001 C (biflora); 3464 (biflora).

Ford, A. 2647 (shanesii).

Ford, C.A. 124 (biflora).

Foreman, D.B. NGF-45698 (vitiensis); NGF-45764 (impar); LAE-60242 (biflora).

Forestry Department 965 (vitiensis).

Forrest, G. 7898 (biflora); 7972 (biflora); 8125 (biflora); 8650 (biflora); 10590 (biflora); 11035 (biflora); 18237 (biflora); 24674 (biflora).

Forster, P.I. 5108 (shanesii); 5808 (shanesii); 8012 (shanesii); 15280 (shanesii); 19385 (shanesii); 24067 (rantonnetii).

Fosberg, F.R. 37656 (biflora); 37890 (biflora).

Frodin, D.G. 2352 (oliveriana).

Fujikawa, K. 53378 (biflora); 89302 (biflora); 89469 (biflora); 89521 (biflora); 94109 (biflora); 94293 (biflora); 95404 (biflora).

Funakoshi, H. 85366 (biflora).

Furuse, M. 39791 (biflora); 44780 (biflora).

Gaerlan, F. PPI-137 (biflora).

Gafui, I. BSIP-16450 (vitiensis); BSIP-16787 (vitiensis); BISP-18862 (vitiensis).

Gallatly, G. 189 (biflora); 386 (biflora).

Gamble, J.S. 741 C (biflora); 3396 (biflora); 18034 (biflora).

Gaoligong Shan Expedition 8709 (yunnanensis); 8727 (biflora); 8829 (biflora); 9728 (biflora); 9758 (biflora).

Gaoligong Shan Biodiversity Survey 15688 (biflora); 15708 (biflora); 15819 (biflora); 16605 (biflora); 18958 (yunnanensis); 20731 (biflora); 20786 (biflora); 22271 (biflora); 22292 (biflora); 26301 (biflora); 27554 (biflora); 28983 (biflora); 32396 (biflora); 32454 (biflora); 33091 (yunnanensis); 33355 (biflora); 33476 (biflora); 33492 (biflora); 33526 (yunnanensis).

Garrett, H.B.G. 1136 (biflora); 1265 (biflora).

Gaudichaud, C. 95 (biflora).

Gibbs, L.S. 568 (vitiensis).

Gibert, E.J. 56 (rantonnetii).

Gideon, O. LAE-57196 (lucens).

Giking, M. 236 (biflora).

Gillespie, J.W. 2016 (vitiensis); 3251 (vitiensis); 4082 (vitiensis); 4446 (vitiensis); 4517 (vitiensis).

Ging, T.S. 5699 (biflora); 7094 (biflora); 16101 (biflora).

Girmansyah, D. Deden-937 (biflora).

Gjellerup, K. 613 (oliveriana).

Glaziou, A.F.M. 1078 (rantonnetii).

Godefroy, A. 139 (biflora).

Godwin, M. C-881 (shanesii); C-2416 (shanesii).

Gonzáles, P. 562 (rantonnetii).

Graeffe, E. 1354 (vitiensis).

Gray, B. 4972 (shanesii); 4974 (shanesii); 4981 (shanesii); 6706 (shanesii); 8068 (shanesii).

Greenwood, W. 127 (vitiensis); 1151 (vitiensis).

Gressitt, J.L. 51 (biflora); 269 (biflora); 272 (biflora).

Grierson, A.J.C. 2290 (biflora).

Griffith, W. 2331 (biflora); 5903 [a] (biflora); 5903 [b] (biflora).

Griswold, J.A. 68 (biflora).

Guppy, H.B. 160 (vitiensis).

Hallier, H. 24 (biflora); 4476 [a] (biflora).

Hamel, C. 496 (biflora); 539 (biflora); 668 (biflora); 679 (biflora); 1242 (biflora).

Hance, H.F. 2128 (biflora).

Hanoi-UBC-Logan-Longwood-Kew Expedition to Vietnam NHE-210 (biflora).

Hara, H. 63-03555 (biflora).

Harder, D.K. 1783 (biflora); 1788 (biflora).

Hartley, T.G. 10065 (oliveriana); 10136 (oliveriana); 11428 (oliveriana); 11434 (bambusarum); 11756 (bambusarum); 12523 a (moszkowskii).

Hassler, É. 6728 (rantonnetii); 7024 (rantonnetii); 8614 (rantonnetii); 11727 (rantonnetii).

Hatschbach, G.G. 23160 (rantonnetii).

Haviland, G.D. 17217 (biflora).

Hayakawa's collector N-415 (biflora).

Henderson, M.R. 18286 (biflora); SF-22323 (biflora); 22378 (biflora); 22709 (biflora); SF-23365 (biflora).

Heng, L. 8727 (biflora); 8829 (biflora); 9728 (biflora); 9758 (biflora); 11476 (biflora).

Hennipman, E. 6039 (biflora).

Henry, A. 307 A (biflora); 307 B (biflora); 307 (biflora); 758 (biflora); 1750 (biflora); 4304 (biflora); 9160 A (biflora); 9160 (biflora); 9218 B (biflora); 9218 C (biflora); 9218 (biflora); 12009 B (biflora); 12009 A (biflora); 12009 (biflora); 12273 (biflora); 12911 (biflora); 13652 (biflora).

Henty, E.E. NGF-29203 (biflora); NGF-41640 (rostellata); NGF-42805 (impar).

Herb Univ Hull Flora of India 281 (biflora); 282 (biflora); 283 (biflora); 284 (biflora).

Herb. Griffith 5903 (biflora).

Herb. Harland 2128 (biflora).

Herb. Sampson 441 A (biflora).

Herbal Community PT-783 (biflora).

Hochreutiner, B.P.G. 825 (biflora); 2041 (biflora).

Höft, R. 29018 (moszkowskii).

Hollrung, M. 776 (oliveriana).

Holstvoogd, C. 496 (biflora).

Hoogland, R.D. 3979 (cladotrichota); 7291 (dendropilosa).

Hoover, W.S. Deden 300 (biflora); 809 (biflora); 30388 (biflora); 30390 (biflora); 30403 (biflora); 31106 (biflora); 31784 (biflora); 32477 (biflora); 32632 (biflora).

Hore, D.K. ANC-7553 (biflora).

Horne, J. 714 (vitiensis).

Horsfield, T. Sol. 10 (biflora); Sol. 13 (biflora).

How, F.C. 70743 (biflora); 72611 (biflora); 73399 (biflora); 73538 (biflora); 73538 (biflora).

Hsiao, S.-.C. 1240 (biflora).

Hu, S.Y. 5523 (biflora); 9277 (biflora); 23888 (biflora).

Hu, W.K. 8921 (biflora).

Huang, S.C. 4486 (biflora).

Huang, T.C. 10675 (biflora); 15179 (biflora).

Hyland, B. 5222 (shanesii); 8713 (shanesii); 11071 (shanesii); 13764 (shanesii); 14694 (shanesii); 14781 (shanesii); 15119 (shanesii); 15534 (shanesii); 16345 (shanesii); 25551 RFK (shanesii); 25640 RFK (shanesii).

Idjan 348 (oliveriana).

Iltis, H.H. G 103 (rantonnetii).

Isles, S. NGF-33899 (biflora).

Jacobs, M. 4805 (biflora).

Jago, R.L. 4014 (shanesii).

James, S.A. SAJ 1385 (cladotrichota).

Jawa, R. S. 70069 (biflora).

Jehen, A. 7747 b (rantonnetii).

Jiménez Chimil, M. JDA-30398 (rantonnetii).

Johansson, J.T. 143 (biflora).

Johns, R.J. 8900 (cladotrichota).

Johnstone, R.L. 3435 (rantonnetii).

Jones, K.D. 1828 (rantonnetii).

Joseph, J. EC-40453 (biflora); 48294 (biflora).

Kairo, A. 62 (moszkowskii); 70 (moszkowskii); 73 (moszkowskii); 79 (moszkowskii); 10652 (biflora); NGF-27869 (biflora); NGF-30943 (kaernbachii); NGF-30983 (kaernbachii).

Kajewski, S.F. 1800 (vitiensis); 1863 (vitiensis); 2388 (vitiensis).

Kalkman, C. BW-3479 (multifolia).

Kamarudin, S. FRI-33752 (biflora).

Kanrtawinana, K. 905 (biflora).

Kao, M.T. 6711 (biflora); 7926 (biflora).

Kao, Y.-.C. 387 (biflora); 560 (biflora).

Katik, P. LAE-70928 (lucens); LAE-70954 (lucens).

Kato, H. C-7273 (biflora).

Kaudern, W.A. 450 (biflora).

Keenan, J. 809 (biflora); 1124 (biflora); 1521 (biflora).

Kere, F. BSIP-4936 (vitiensis); BSIP-5057 (vitiensis).

Kerenga, K. LAE-77590 (moszkowskii); LAE-77644 (rostellata).

Kerr, A.F.G. 3510 (biflora); 5030 (biflora); 5550 (biflora); 6742 (biflora); 7069 (biflora); 13293 (biflora).

Kerrigan, R.A. 1343 (shanesii).

Kessler, P.J.A. 934 (biflora); 3014 (biflora); 3038 (biflora).

King's collector 10930 (biflora).

King, G. 995 (biflora).

Kingdon-Ward, F. 13884 (biflora); 22249 (biflora).

Kloss, C.B. SF-18984 (biflora).

Knapp, S. 10106 (biflora).

Ko, S.P. 55822 (biflora).

Kobayashi, S. 2811 (biflora).

Kochummen, K.M. FRI-16186 (biflora).

Koelz, W. 8780 (rantonnetii); 11211 (biflora).

Konta, F. T-29815 (biflora).

Koorders, S.H. 18038 (biflora); 27963 B (biflora); 37847 B (biflora).

Kornassi 649 (oliveriana).

Koroiveibau, D. 12807 (vitiensis).

Kostermans, A.J.G.H. 1024 (biflora); 2704 (oliveriana); 13995 (biflora).

Koyama, H. T-30451 (biflora); T-31410 (biflora); T-61226 (biflora).

Krispinus, F. SAN-120254 (biflora).

Kuntze, C.E.O. 6007 (biflora).

Kuo, C.M. 8880 (biflora).

Kuroiwa, N. 51266 (biflora); 51333 (biflora).

Kuruveli, I.T. 13888 (vitiensis); 14007 (vitiensis).

Kurz, S. 1502 (biflora); 1775 (biflora); 1777 (biflora); 1803 (biflora).

Lai, S.T. S. 72457 (biflora).

Lammers, T.G. 8506 (biflora).

Lamont, J. 493 (biflora).

Larsen, K. 9007 (biflora); 30658 (biflora).

Lau, S.K. 4303 (biflora); 4768 (biflora); 20223 (biflora); 25593 (biflora); 28538 (biflora).

Lau, S.Y. 20223 (biflora).

Lawson, E. 51 (rantonnetii).

Ledermann, C.L. 12606 (cladotrichota).

Ledua, M.K. 11018 (vitiensis).

Leslie, J.E. 180 (biflora).

Leu, W.-.P. 823 (biflora); 886 (biflora).

Levine, C.O. 106 (biflora); 234 (biflora); 1652 (biflora); 1882 (biflora); 3131 (biflora); 3458 (biflora).

Liang, H.Y. 64881 (biflora).

Liao, C.-.C. 411 (biflora).

Licehong 96062 (biflora).

Lin, C.-.H. 869 (biflora).
Lingnan University Herbarium 12648 (biflora).
Liou, T.N. 834 (biflora).
Liu, J.H. 442 (biflora).
Long, D.G. 1057 (biflora).
Lorence, D.H. 7766 (rantonnetii).
Lörzing, J.A. 11366 (biflora); 13440 (biflora).
Lovato, M.C. 153 (rantonnetii).
Lugas, L. 2152 (biflora).
Lütjeharmes, W.J. 5406 (biflora).
MacGregor, R.W. 1302 (biflora).
MacKee, H.S. 2995 (vitiensis).
Madani, L. SAN-111183 (biflora).
Mamit, J.D. S. 33474 (biflora).
Man, L.S. 55051 (biflora); 55348 (biflora); 87558 (biflora); 88162 (biflora); 96155 (biflora).
Mananduar, R.K. 6805 (biflora).
Mandal, N.R. BSHC-11037 (biflora); BSHC-11132 (biflora); BSHC-11823 (biflora); BSHC-14672 (biflora).
Mann, G. 136 (biflora).
Marcan, A. 1156 (biflora); 1398 (biflora).
Matthew, K.M. RHT-23335 (denticulatum); RHT-42486 (biflora).
Mauriasi, R. BSIP-8491 (vitiensis); BISP-13952 (vitiensis); BSIP-14089 (vitiensis); BSIP-14154 (vitiensis).
Maxwell, J.F. 93-127 (biflora); 09-262 (biflora); 05-456 (biflora); 97-476 (biflora); 06-534 (biflora); 91-759 (biflora); 94-866 (biflora).
McCaskill, J. 353 (rantonnetii); 612 (rantonnetii).
McClure, F.A. 9699 (biflora).
McDonald, W.J. 5862 (shanesii).
Mcdonald, W.J.F. 6674 (shanesii); 6768 (shanesii).
Meares, K. 33 (rantonnetii).
Meebold, A. 6906 (biflora); 17036 (vitiensis); 17068 (biflora).
Merrill, E.D. 1719 (biflora); 6588 (biflora); 9525 (biflora).
Mgadiman SF-36779 (biflora).
Middleton, D.J. 139 (biflora); 3452 (biflora).
Millar, A.N. NGF-11795 (biflora); NGF-18664 (rostellata); NGF-23260 (kaernbachii); NGF-23365 (kaernbachii); NGF-23549 (cladotrichota); NGF-23858 (oliveriana); NGF-40737 (rostellata).
Millar, H.N. NGF-11794 (biflora).

Miyazaki, T. 508073 1/2 (biflora).

Molina R, A. 24520 (rantonnetii).

Molino, J.-.F. 3060 (oliveriana).

Moll, V.W. BW-9529 (biflora).

Monro, A.K. 6455 (biflora); 6498 (biflora).

Moran, R. 28811 (rantonnetii).

Morong, T. 147 (rantonnetii).

Mueller, F.J.H. von 13 (vitiensis).

Murata, G. T-15042 (biflora); T-16856 (biflora); T-17789 (biflora); T-17790 (biflora); T-37073 (biflora).

Murata, J. 24682 (biflora).

Muroi, H. 2513 (biflora).

Nakisi, A. BSIP-8013 (vitiensis).

Nanakorn, W. 1090 (biflora).

Native Collector 2127 (biflora).

Nee, M. 18736 (rantonnetii); 22986 (rantonnetii).

Nguyen, V.D. HNK-1507 (biflora).

Nicholson, D.I. AFO-4776 (shanesii).

Nicholson, N.J. NJN 455 (shanesii).

Nitta, A. 15090 (biflora).

Nuraliev, M.S. NUR-101 n (biflora); NUR-182 a (biflora).

O'Shanesy, P.A. 6 ser. 1 (shanesii).

Ohashi, H. 77 5702 (biflora).

Okada, H. 3284 (biflora).

Oldham, R. 337 (biflora).

Ou, C.H. 9297 (biflora).

Palee, P. 844 (biflora); 995 (biflora).

Panigrahi, G. 14531 (biflora).

Parham, J.W. 13051 (vitiensis).

Patsipun S. 79958 (biflora); S. 82141 (biflora).

Peng, C. 8359 (biflora).

Pereira, J.T. 558 (biflora).

Pételot, A. 2207 (biflora); 3110 (biflora).

Phuong, V.X. HNK-228 (biflora).

Pierre, L. 635 (biflora).

Pleyte, D.R. 623 (oliveriana).

Po, U.S. 12053 (biflora).

Poilane, E. 17706 (biflora).

Polak, A.M. 651 (oliveriana); 864 (oliveriana).

Powell, D.A. 164 (biflora).

Powell, T. 365 (vitiensis).

Premanath, R.K. ANC-8464 (biflora).

Price, W.R. 299 (biflora).

Pullen, R. 6011 (moszkowskii).

Puradyatmika, P. 10428 (impar).

Put Phraisurind 156 (biflora).

Qoro, I. USDA-14091 (vitiensis).

Rai, S.K. BSHC-21852 (biflora); BSHC-23988 (biflora); BSHC-25852 (biflora); BSHC-38370 (biflora); BSHC-38371 (biflora).

Raigiso, F.C. 3141 (vitiensis).

Ramesh, S.R. 3938 (biflora).

Ramlanto, ? 869 (biflora).

Ramos, M. 4648 (biflora); 23438 (biflora); 30519 (biflora); 40268 (biflora); 40336 (biflora); 40439 (biflora); 45017 (biflora); 48403 (biflora).

Rao, A.S. 48039 (biflora).

Rao, R.S. 10400 (biflora).

Rao, T.A. 826 (biflora).

Rau, K. 73 (moszkowskii), 380 (biflora).

Raynal, A. 18863 (biflora); 18882 (biflora).

Rechinger, K. 4398 (vitiensis).

Reinecke, F. 58 (vitiensis); 58 a (vitiensis); 78 (vitiensis).

Reporter on Economic Products to the Govt. of India 11536 (biflora).

Reynoso, ? PPI-3639 (biflora).

Richards, P.W. 2622 (biflora).

Ridley, H.N. 34 (biflora).

Riley, J.C. 45 (vitiensis).

Roberts, L.J. KRM-15160 (shanesii).

Robinson, H.C. 5 (biflora); 61 (biflora).

Rock, J.F. 1706 (biflora).

Rodríguez, D. 1470 (rantonnetii).

Rosales, J.M. 1253 (rantonnetii).

Royen, P. van 7621 (oliveriana); 7716 (oliveriana); 11511 (rostellata); NGF-18229 (rostellata).

Ruse, L.F. 141 (biflora).

Russell, P.T. 2166 (biflora); 2219 (biflora).

Ryan, G.M. 1495 (biflora).

Sá, K.L.V.R. 513 (rantonnetii).

Saigol, P. SAN-93078 (biflora).

Saito, S. 7294 (biflora).

Sampson, T. 441 (biflora).

Sandoval, E. ES 1575 (rantonnetii).

Sands, M.J.S. 1751 (moszkowskii); 1966 (lucens); 2073 (lucens); 2230 (lucens); 6431 (biflora); 6744 (oliveriana); 6791 (biflora); 7329 (oliveriana).

Sankowsky, G. 615 (shanesii).

Sapiin 2473 (biflora).

Saulière, A. 3 (biflora); 72 (biflora); 73 (biflora).

Sayers, C.D. NGF-19830 (moszkowskii); NGF-21517 (moszkowskii).

Schiffner, V. 2512 (biflora).

Schlechter, F.R.R. 13748 (biflora); 13749 a (biflora); 17305 (biflora); 17339 (kaernbachii); 17961 (oliveriana); 18319 (oliveriana); 18427 (oliveriana); 20256 (oliveriana).

Schodde, R. 4094 (vitiensis); 5705 (rostellata).

Schram, F.A.W. BW-10645 (biflora); BW-10744 (biflora).

Seemann, B.C. 42 (vitiensis); 340 (vitiensis); 387 (vitiensis).

Sengupta, G. 354 (biflora).

Shah, M. 695 (biflora).

Shapcott, A. MGH-40 (shanesii).

Shin Ying Hu 5523 (biflora).

Shukla, B.K. BSHC-21022 (biflora).

Si Boeea, R. 6619 (biflora); 8649 (biflora); 8765 (biflora); 9034 (biflora); 10182 (biflora); 10232 (biflora); 10242 (biflora); 10323 (biflora); 10418 (biflora); 10780 (biflora); 10971 (biflora).

Sidiyasa, K. 2124 A (biflora).

Sinclair, J. 9761 (biflora).

Siti Munirah, M.Y. FRI-76680 (biflora).

Smith, A.C. 574 (vitiensis); 1244 (vitiensis); 1508 (vitiensis); 1826 (vitiensis); 5250 (vitiensis); 5270 (vitiensis); 5501 (vitiensis); 5828 (vitiensis); 7343 (vitiensis); 7797 (vitiensis); 8082 (vitiensis); 8894 (vitiensis); 8930 (vitiensis); 9171 (vitiensis); 9264 (vitiensis).

Soewarta 143 (biflora).

Soibeh, D. 789 (biflora).

Solomon, J.C. 20620 (biflora).

Sorenson, T. 3730 (biflora).

Srivastava, R.C. BSHC-10301 (biflora).

Steenis, C.G.G.J. van 6212 (biflora); 7310 (biflora); 9354 (biflora); 10816 (biflora).

Sterly, J. 80-469 (belensis).

Stevens, P.F. LAE-54766 (bambusarum); LAE-58668 (biflora).

Steward, A.N. 605 (biflora).

Streimann, H. 9635 (bambusarum); NGF-25853 (biflora); NGF-25854 (bitteriana); NGF-27634 (moszkowskii); NGF-34091 (cladotrichota); NGF-35924 (moszkowskii); LAE-51786 (impar); LAE-53892 (bambusarum).

Subils, R. 4260 (rantonnetii); 4560 (rantonnetii); 4561 (rantonnetii); 4651 (rantonnetii); 4707 (rantonnetii).

Sugau, J.B. SAN-147090 (biflora).

Sulit, M.D. 16964 (biflora).

Sundaling, P. SAN-71284 (biflora).

Surunda, Y. 100 (biflora).

Sykes, W.R. Ch-63 (biflora).

Symon, D.E. 10631 (moszkowskii); 10632 (bambusarum); 10651 (bitteriana); 10655 (oliveriana); 10659 (oliveriana); 10676 (cladotrichota); 10677 (rostellata); 10678 (rostellata); 10687 (rostellata); 10690 (rostellata); 10691 (dendropilosa); 10704 (rostellata); 13822 (bambusarum); 13828 (moszkowskii); 13829 (bambusarum); 13830 (moszkowskii); 13846 (moszkowskii); 13854 (moszkowskii); 13874 (rostellata); 13875 (rostellata); 13876 (rostellata); 13877 (rostellata); 13896 (oliveriana).

Taam, Y.W. 2503 (biflora).

Tadong, D. 506 (biflora).

Tai, L.Y. T-453 (biflora).

Takahashi, H. T-62628 (biflora); T-63382 (biflora).

Takeuchi, W.N. 9181 (impar); 9307 (oliveriana); 10663 (moszkowskii); 11204 (peranomala); 11704 (bambusarum); 11804 (rostellata); 12379 (cladotrichota); 12688 (biflora); 16902 (biflora); 17743 (oliveriana); 17786 (oliveriana); 22112 (oliveriana); 22892 (cladotrichota); 23389 (oliveriana); 23491 (oliveriana); 23895 (oliveriana).

Talbot, W.A. 3007 (biflora).

Tanaka, N. MY 338 (biflora); 23001 (biflora); 23235 (biflora); 23525 (biflora); 30626 (biflora); 81320 (biflora).

Tanaka, T. 341 (biflora); 17877 (biflora).

Templeton, B.C. 9 (rantonnetii).

Teng, S.W. 90828 (biflora).

Teona, R. BISP-6362 (vitiensis).

Thomas, G. 8951 (shanesii).

Thompson, E.J. GLA-22 (shanesii).

Thorel, C. 2087 (biflora).

Tibet-MacArthur 2111 (biflora).

Tothill, B.H. 556 (vitiensis); 633 (vitiensis); 636 (vitiensis); 638 (vitiensis); 639 (vitiensis).

Trethewy, A.W. 9 (rantonnetii).

Tsai, H.T. 52494 (biflora); 54160 (biflora); 54273 (biflora); 58534 (biflora); 61720 (biflora).

Tsang, W.T. 355 (biflora); 540 (biflora); 21464 (biflora); 28144 (biflora).

Tsiang, Y. 419 (biflora); 4621 (biflora); 9256 (biflora); 9505 (biflora); 12428 (biflora).

Tsui, T.M. 411 (biflora); 422 (biflora); 827 (biflora).

Uji, T. 2750 (biflora).

Utteridge, T.M.A. 119 (impar); 287 (oliveriana); 295 (oliveriana).

Vandenberg, J. NGF-39883 (rostellata).

Vaupel, F. 104 (vitiensis).

Vavryn, D. 17 (shanesii).

Vera Santos, J. 4207 (biflora).

Vieira, A.O.S. 112 (rantonnetii).

Vinas, A. LAE-59477 (oliveriana).

Vinas, A.N. 308 (kaernbachii).

Vink, W. 16847 (oliveriana).

Vogel, E.F. de 1326 (biflora); 3565 (biflora); 3718 (biflora).

Voogd, C.N.A. de 972 (biflora).

Walker, E.H. 5876 (biflora); 7608 (biflora); 8336 (biflora).

Walker, G.W. 236 (biflora).

Wallich, N. Cat. 2614 (biflora); Cat. 2621 (biflora); Cat. 2625 b (biflora); Cat. 2625 (biflora).

Walsh, M.E. 99 (biflora).

Wan, P.P. 79100 (biflora).

Wang, C. 32543 (biflora); 33638 (biflora); 36950 (biflora); 40596 (biflora); 40894 (biflora); 42589 (biflora).

Wang, C.-.C. 544 (biflora).

Wang, C.W. 72647 (biflora); 76510 (biflora); 76822 (biflora); 78209 (biflora).

Wang, J.-.C. 2693 (biflora); 2781 (biflora).

Wang, W.H. 8113 (biflora).

Wannan, B.S. 2328 (shanesii).

Warburg, O. 4186 (biflora); 15067 (biflora); 21250 (biflora).

Waterhouse, B.M. 4806 (shanesii); 5114 (shanesii); 5114 (shanesii).

Waterhouse, J.H.L. 66 (vitiensis); 272 -B (vitiensis).

Watt, G. 5647 (biflora).

Webster, G.L. 15188 (moszkowskii); 32022 (rantonnetii).

Wells, J. NGF-7565 (bitteriana); NGF-7569 (biflora).

Wen, J. 7373 (biflora); 7374 (biflora); 10282 (biflora).

Whistler, A. 4 (vitiensis); 520 (vitiensis); 891 (vitiensis); 1071 (vitiensis); 1090 (vitiensis); 1253 (vitiensis).

Whitmee, S.J. 52 (vitiensis); 185 [a] (vitiensis).

Whitmore's collectors BSIP-3081 (vitiensis).

Whitmore, T.C. BSIP-776 (vitiensis); BSIP-2437 (vitiensis); BSIP-2567 (vitiensis).

Wiakabu, J. LAE-70476 (multifolia).

Widjaja, E.A. 8917 (biflora); 9734 (biflora).

Wight, R. 691 (biflora); 1569 [2417] (biflora); 1569 -103 (biflora); 1569 -127 (biflora); 1569 -105 (biflora); 1569 -101 (biflora); 2025 (biflora).

Wilde, W.J.J.O. de 18335 (biflora).

Williams, R.S. 1334 (biflora).

Wilson, E.H. 4202 (biflora); 5096 (biflora).

Without Collector 40 (biflora); 85 (biflora); 436 (biflora); 649 (biflora); 1448 (biflora); 3007 (biflora); 5487 (biflora).

Womersley, J.S. 4883 (rostellata); NGF-37289 (oliveriana).

Worboys, S.J. 1344 (shanesii).

Wray, L. 1460 (biflora).

Wright, C. 197 (biflora).

Wu, M.J. 1331 (biflora).

Wu, S.-.H. 1053 (biflora).

Yahara, T. 7431 (biflora).

Yamamoto, Y. 1150 (biflora).

Yates, H.S. 2864 (biflora); 3038 (biflora).

Yen, H.F. 8400 (biflora).

Yii S. 70379 (biflora).

Ying, T. 419 (biflora).

Yonekura, K. 11397 (biflora).

Yu, D. 16455 (biflora).

Yu, T.T. 16404 (biflora); 20377 (biflora).

Yuncker, T.G. 16103 (vitiensis).

Zhang, T. 10CS-2040 (biflora).

Zollinger, H. 723 (biflora); 1799 (biflora); 1981 (biflora).

Zuloaga, F.O. 6713 (rantonnetii); 6727 (rantonnetii).

Zwickey, A.L. 163 (biflora);
